# Investigation on predominant *Leptospira* serovars and its distribution in humans and livestock in Thailand, 2010-2015

**DOI:** 10.1371/journal.pntd.0005228

**Published:** 2017-02-09

**Authors:** Sudarat Chadsuthi, Dominique J. Bicout, Anuwat Wiratsudakul, Duangjai Suwancharoen, Wimol Petkanchanapong, Charin Modchang, Wannapong Triampo, Parntep Ratanakorn, Karine Chalvet-Monfray

**Affiliations:** 1 Department of Physics, Faculty of Science, Naresuan University, Phitsanulok, Thailand; 2 Biomathematics & Epidemiology, EPHP–TIMC Lab, UMR 5525 CNRS Univ Grenoble Alpes, VetAgro Sup, 69280 Marcy l’Etoile, France; 3 Department of Clinical Sciences and Public Health, and the Monitoring and Surveillance Center for Zoonotic Diseases in Wildlife and Exotic Animals, Faculty of Veterinary Science, Mahidol University, Nakhon Pathom, Thailand; 4 National Institute of Animal Health, Department of Livestock Development, Bangkok, Thailand; 5 National Institute of Health, Department of Medical Sciences, Ministry of Public Health, Nontaburi, Thailand; 6 Biophysics Group, Department of Physics, Faculty of Science, Mahidol University, Bangkok, Thailand; 7 Centre of Excellence in Mathematics, CHE, 328, Si Ayutthaya Road, Bangkok, Thailand; 8 Department of Clinical Science and Public Health, Faculty of Veterinary Science, Mahidol University, Nakhon Pathom, Thailand; 9 UMR EPIA, INRA, VetAgro Sup, Univ Lyon, 69280 Marcy l’Etoile, France; Mahidol University, THAILAND

## Abstract

**Background:**

Leptospirosis is a worldwide zoonotic bacterial disease caused by infection with leptospires. Leptospirosis in humans and livestock is an endemic and epidemic disease in Thailand. Livestock may act as reservoirs for leptospires and source for human infection.

**Methodology/Principal findings:**

Data on leptospirosis infection in humans and livestock (Buffaloes, Cattle, and Pigs) species during 2010 to 2015 were analyzed. Serum samples were examined using Microscopic Agglutination Test (MAT) to identify antibodies against *Leptospira* serovars using a cut-off titer ≥ 1:100. The seroprevalence was 23.7% in humans, 24.8% in buffaloes, 28.1% in cattle, and 11.3% in pigs. Region specific prevalence among humans and livestock was found in a wide range. The most predominant serovars were Shermani, followed by Bratislava, Panama, and Sejroe in human, Shermani, Ranarum, and Tarassovi in buffaloes, and Shermani and Ranarum in cattle and pigs. Equally highest MAT titers against multiple serovars per one sample were found mainly in buffaloes and cattle showing equally titers against Ranarum and Shermani. The correlations of distribution of serovars across Thailand’s regions were found to be similar in pattern for cattle but not for buffaloes. In humans, the serovar distribution in the south differed from other regions. By logistic regression, the results indicated that livestock is more susceptible to infection by serovar Shermani when compared to humans.

**Conclusions/Significance:**

This study gives a detailed picture of the predominance of *Leptospira* serovars in relation to region, humans and typical livestock. The broad spatial distribution of seroprevalence was analyzed across and within species as well as regions in Thailand. Our finding may guide public health policy makers to implement appropriate control measures and help to reduce the impact of leptospirosis in Thailand.

## Author summary

Leptospirosis is an important worldwide zoonotic disease, particularly in tropical and subtropical countries. The infection in humans is caused by either direct contact with products of infected animals, mainly urine, or by indirect contact via a contaminated environment. The animal hosts are thus considered reservoirs for human infection as livestock in Thailand usually live in close contact with householders in rural areas. However, the links of *Leptospira* serovars in humans and livestock in Thailand are poorly understood. Therefore, we illustrate the circulation of *Leptospira* serovars in humans and livestock during the past six years. The cross-correlations of the seroprevalence distribution were investigated to assess similarity between serovars across and within both, species and regions. The results suggest that livestock could be a potential source for human infection as sample analysis revealed a predominance of the same serovars. This information will increase public health awareness and may benefit especially high risk groups such as abattoir workers, livestock owners, farmers and other animal handling personal.

## Introduction

Leptospirosis is a worldwide zoonotic bacterial disease particularly in tropical and subtropical countries [1]. Pathogenic *Leptospira* species are causative agents of the disease, specifically *Leptospira interrogans* sensu lato. There are approximately more than 250 recognized pathogenic serovars and 24 antigenically related serogroups [2,3]. Serovars, that are antigenically related, can be grouped into serogroups. The serogroups of *L*. *interrogans* can have some common serovars [4]. The infection in humans is caused by direct contact with products of infected animals, mainly urine, and also by indirect contact as the organisms can be transmitted to humans through cut skin or mucous membranes via a contaminated environment [5].

The continuing epidemic of human leptospirosis in Thailand produces an annual incidence rate of about 5.9 cases per 100,000 population each year during the last ten years [6]. From March 2003 to November 2004, most confirmed cases occurred in the north and northeast regions of the country [7]. The number of reported cases was highest during the rainy seasons. Farmers and other agricultural workers make up the main occupational risk groups, which are likely to be exposed with contaminated wet soil and water during their daily activities [8–10], for example during rice cultivation, fish capture and animal farming. In the environment, leptospires may survive from a few weeks to almost a year in wet soil on dry days or in surface waters on rainy days [11]. The animal hosts (e.g., cattle, buffaloes, pigs, dogs, and wildlife) are considered as common reservoirs of leptospires and may act as a source for human infection [1]. Domesticated livestock in Thailand usually dwell in close proximity to their owners in rural areas, which poses a certain risk of interspecies transmission.

In a previous study conducted in Thailand during January to August 2001, it was found that the most commonly detected *Leptospira* serovars in cattle were *Leptospira* serovars Ranarum, Sejroe, and Mini, whereas Mini, Sejroe, and Bratislava were mostly detected in buffaloes, Ranarum, Pomona, and Bratislava in pigs, and Mini, Shermani, and Ranarum in sheep and goats, respectively [12]. The sera of patients in Bangkok Thailand were found to have the highest reactions to *Leptospira* serovars Shermani and Bratislava [13]. Undoubtedly, animals hosting and shedding leptospires pose a certain risk to public health as even vaccinated livestock are reported to shed *Leptospira* into urine [14]. The host reservoirs may be infected asymptomatically while infected humans with exactly the same serovars may develop serious illnesses [15,16].

Preventive measures by public health sections were typically focused on the increase of public awareness on the exposure risks when dealing with animals [17]. However, understanding of the diversity of *Leptospira* serovars in animals and humans should also be taken into account in order to identify associations of animal reservoirs with human infection.

We established a large-scale dataset gathered from passive surveillance over a six year period, which may represent the largest and longest study of leptospirosis among buffaloes, cattle, pigs and humans in Thailand so far. This basic knowledge of serovars and their maintenance hosts is critical to provide more understanding of the epidemiology of leptospirosis. The aims of this study were i)–to determine predominant *Leptospira* serovars circulating between humans and livestock in the epidemiological context of Thailand, ii)–to identify spatial distributions of *Leptospira* serovar seroprevalence in humans and livestock in each of the 5 regions Thailand, iii)–to assess the similarity between the distribution of predominant serovars across and within both species and regions in Thailand using cross-correlation analysis, and iv) to provide a detailed investigation of *Leptospira* serovar seropositivity according to species and/or regions based on statistical analysis. This study also gives an update on leptospirosis in Thailand and information about host and human serovar association to support public health management.

## Materials and methods

### 2.1 Data sources

A total of 7,218 livestock serum samples derived from 432 buffaloes, 3,648 cattle, and 3,138 pigs were submitted to National Institute of Animal Health, the Department of Livestock Development, Ministry of Agriculture, Thailand from January 2010 to December 2015 under the passive surveillance program of leptospiral seropositivity, which is a part of the passive surveillance program. Most of samples in this study were collected from rural areas. This study was not basically designed for research proposal; an animal ethic protocol was not required. The samples were sent by different reasons, e.g., routine diagnosis, health check, and leptospirosis investigation. Most animals were not vaccinated against leptospirosis (6,934, of 7,218 samples, 96.07%) and were collected from rural areas.

The 1,990 human serum samples under suspicion of leptospirosis, which have some clinical symptoms of leptospirosis such as high fever, headache, muscle aches, Jaundice, and diarrhea, were sent to National Institute of Health, Department of Medical Sciences, Ministry of Public Health, Thailand during January 2010 to December 2015. Data collection was performed as a part of routine clinical examination procedures for which results were previously transmitted to patient and consent was thus not required by the Ethics Committee. Data contained in the patient’s records, without any patient information, except location and time, was de-identified prior to an anonymous analysis.

### 2.2 Serovar identification

All serum samples were examined for the presence of *Leptospira* serovar antibodies by the microscopic agglutination test (MAT). The MAT is the most widely used method in identifying leptospiral positive samples [3]. Serological tests and leptospira culture protocol in this study were based on the standard methodology [18,19] using a panel of 23 reference serovars [12]. The panel of antigens included *L*. *interrogans* serovars Bratislava (BRA) (serogroup Australis), Autumnalis (AUT), Ballum (BAL), Bataviae (BAT), Canicola (CAN), Celledoni (CEL), Cynopteri (CYN), Djasiman (DJA), Grippotyphosa (GRI), Hebdomadis (HEB), Icterohaemorrhagiae (ICT), Javanica (JAV), Louisiana (LOU), Manhao (MAN), Mini (MIN), Panama (PAN), Pomona (POM), Pyrogenes (PYR), Ranarum (RAN), Sarmin (SAR), Sejroe (SEJ), Shermani (SHE), and Tarassovi (TAR).

Based on practical approaches, the MAT titer ≥ 1:100 is recommended cut-off and was used to determine seropositivity [15,20]. However, previously, the MAT showed high sensitivity and specificity of 95% and 89% at a cut-off titer 1:50, respectively [21]. A dilution of 1:100 sera was screened as positive sera [22]. This cut-off titer was also used in previous studies [23,24]. This study, the occurrence of humans and livestock leptospirosis was determined by serological test. The cut-off titer was chosen to ≥ 1:100 to increase the previously specificity. Cross-reactivity between different serogroups may occur in MAT due to the detection of both IgM and IgG antibodies [5]. In this case, the highest MAT titer criteria were used to identify the predominant serovar(s). Infection by multiple serovars was assumed in case of equally highest MAT titers against two or more serogroups. The distribution of leptospiral serovar seroprevalence was measured within and across both species and regions in Thailand. The seroprevalence was calculated from the proportion of leptospiral culture positive hosts and all tested samples.

### 2.3 Statistical analysis

The spearman correlation test was employed to test the correlation coefficient between pairwise comparisons of seropositive frequency distribution within and across both species and regions. According to Thai Meteorological Department, Thailand can be divided into 5 regions, i.e., northern, northeastern, central, eastern, and southern regions by using climate pattern and meteorology [25]. In northern region, most area is hilly and mountainous, which have lowest average temperature. Northeastern region is naturally a high level plain with slightly higher average temperature than northern region, while central region is a large lower plain with high average temperature. Eastern region is the part of adjacent of the Gulf of Thailand, which have high average temperature as well as in central region. The topography of southern region is the peninsula in Andaman Sea, which has the highest average annual rainy day comparing to the other 4 regions. The season of southern region is divided into two major seasons which are the rainy (June-February) and the summer (March-May) seasons, while the other 4 regions have 3 seasons, i.e., a rainy season (mid-May to mid-October), a winter season (mid-October to mid-February), and a summer season (mid-February to mid-May). All calculations were performed using R software [26]. The Bonferroni correction was used to adjust the P-value and control type I error rates in multiple comparisons. All pairwise comparisons with adjusted a P-value < 0.05 were considered significant.

To investigate the relation of serovars according to species and/or regions, a logistic regression model (Generalized Linear Model (GLM) with binomial function) [27] was performed using R software [26]. A total of 9,208 serum samples were analyzed. The presence or absence (Yes/No) of antibodies against serovars was analyzed. Five serovars were selected based on the highest seroprevalence, i.e., Bratislava, Ranarum, Sejroe, Shermani, and Tarassovi (S1 Table). The best univariable or multivariable model was selected using a stepwise forward approach based on the Akaike Information Criterion (AIC). The models were compared in regard to deviance and degree of freedom at a significance level of P-value < 0.05 under chi-squared distribution. The interaction between all effects was also investigated. The regression diagnostic model was performed by Cook's distance and leverage methods.

## Results

### 3.1 Overall prevalence

The *Leptospira* seroprevalence in 4 species (buffaloes, cattle, pigs, and humans) and 5 regions (northern, northeastern, central, eastern, and southern) as determined by MAT using a 23 serovar panel during the period 2010–2015 is summarized in Table 1. A total of 7,218 animals were tested with 1,489 (20.6%, 95% CI [19.7–21.6%]) animals found positive by MAT. The seroprevalence was 24.8% [20.8–29.1%] in buffaloes, 28.1% [26.7–29.6%] in cattle, and 11.3% [10.3–12.5%] in pigs. For humans, a total of 1,990 samples were tested and 471 (23.7% [21.8–25.6%]) were found seropositive. Seropositive samples reacting with two or more serovars was highest in cattle measuring 43.3% [40.2–46.4%] and lowest in pigs counting 16.9% [13.1–21.1%]. In humans, seropositivity against multiple serovars occurred in 15.3% [12.1–18.9%] of tested samples.

**Table 1:** The leptospiral prevalence by species and region.

	Total samples	No. of positive samples (%)	Single serovar seropositivity^a^ (%)	Multiple serovar seropositivity^b^ (%)
Species				
- Buffaloes	432	107 (24.8%)	70(65.4%)	37(34.6%)
- Cattle	3,648	1,026 (28.1%)	582(56.7%)	444(43.3%)
- Pigs	3,138	356 (11.3%)	296(83.2%)	60(16.9%)
- Humans	1,990	471(23.8%)	399(84.7%)	72(15.3%)
Regions				
- Northern	883	116(13.1%)	80(69.0%)	36(31.0%)
- Northeastern	1,937	513(26.5%)	360(70.2%)	153(29.8%)
- Central	4,914	917(18.7%)	652(71.1%)	265(28.9%)
- Eastern	878	203(23.1%)	107(52.7%)	96(47.3%)
- Southern	596	211(35.4%)	148(70.1%)	63(29.9%)

^a^The highest MAT titer reacting with one serovar.

^b^Two or more serovars with equally highest MAT titer.

MAT titer at threshold ≥1:100.

To assess the spatial seroprevalence, Thailand was divided into 5 regions: northern, northeastern, central, eastern, and southern according to climate variations [25]. Animal locations were based on the owner’s address. The highest overall prevalence (35.4% [31.6–39.4%]) (pooled data of 4 species) was found in the southern region and the lowest overall prevalence was identified in the north (13.1% [11.0–15.5%]). The highest prevalence reacting with multiple serovars by region was found in the eastern region (47.3% [40.2–54.5%]), while the lowest was found in the central region (28.9% [26.0–32.0%]).

**Fig 1:**
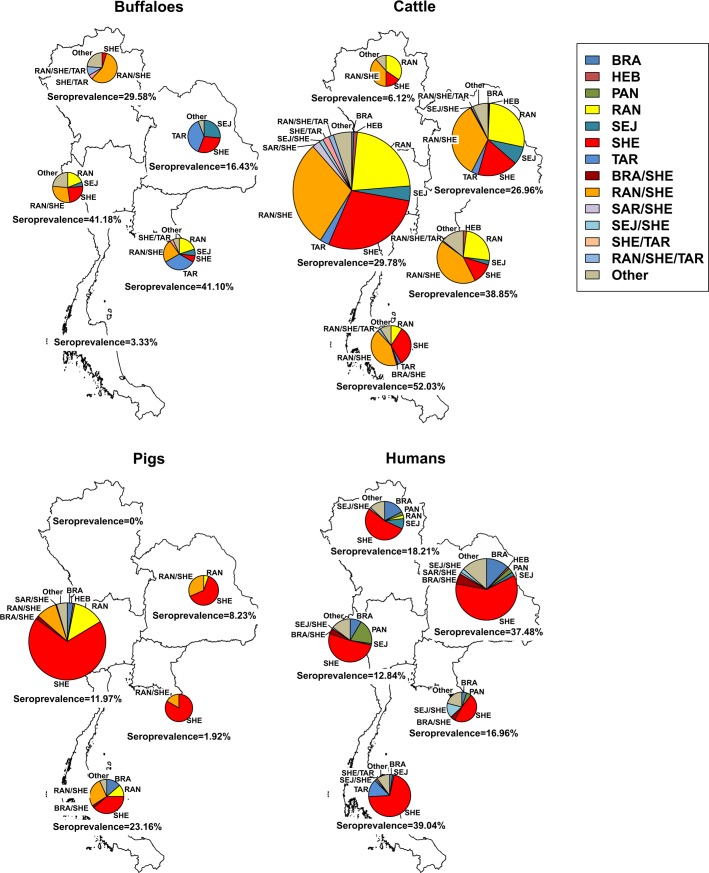
Regional distributions of most prevalent serovars and serovar associations in buffaloes, cattle, pigs, and humans in 5 regions of Thailand. By using the highest MAT titer criteria: BRA = Bratislava, HEB = Hebdomadis, PAN = Panama, RAN = Ranarum, SEJ = Sejroe, SHE = Shermani, TAR = Tarassovi, BRA/SHE = Bratislava and Shermani, RAN/SHE = Ranarum and Shermani, SAR/SHE = Sarmin and Shermani, SEJ/SHE = Sejroe and Shermani, SHE/TAR = Shermani and Tarassovi, and RAN/SHE/TAR = Ranarum, Shermani and Tarassovi, and the other group. The sizes of pie charts refer to the size of positive MAT samples. The seroprevalence was calculated relative to the number of samples for each species and in each region.

Region specific prevalence in buffaloes ranged from 16.4% [11.7–22.2%] in the northeastern region to 41.2% [27.6–55.8%] in the central region (one positive sample (3.33%) in the south) (Fig 1). In cattle, findings ranged from 6.1% [4.0–8.8%] in the northern region to 52.0% [44.7–60.3%] in southern region. The spatial seroprevalence in pigs ranged from 1.9% [0.7–4.1%] in the eastern region to 23.2% [31.1–50.2%] in the southern region with no positive sample in the north. In humans, the value ranged from 12.8% [10.5–15.5%] in the central region to 39.0% [32.7–45.7%] in the southern region.

### 3.2 *Leptospira* serovar predominance

The predominance of serovars in humans and livestock was presented in Fig 1. High titer seropositivity against single serovars was observed for 7 serovars and against multiple serovars for 6 serovars. Less prevalent serovars were grouped into “Other”. In buffaloes and in the northern and central regions, multiple serovar association was identified showing equally titers against serovars Ranarum and Shermani (57.1% and 28.6%, respectively), whereas in the northeastern and eastern regions the predominant serovar was serovar Tarassovi (38.2% and 33.3%, respectively). In cattle, the most common MAT reaction was Ranarum/Shermani for samples from all regions (38.6% in northern, 34.5% in northeastern, 29.6% in central, 42.6% in eastern and 42.9% in southern regions respectively). The most common serovar in all regions (except northern, where no positive sample was observed) in pigs was serovar Shermani (63.2% in the northeast, 68.6% in central, 83.3% in the east, and 38.6% in the southern regions, respectively), which coincides with the human samples (53.6% in northern, 60.3% in northeastern, 51.6% in central, 47.4% in eastern, and 70.8% in southern regions, respectively).

By considering each serovar separately, regardless of species and regions, seropositive MAT titers of the first 5 predominant serovars were Shermani (67.9%), Ranarum (38.9%), Sejroe (6.4%), Bratislava (5.9%), and Tarassovi (4.5%), while titers against Canicola and Celledoni were not observed (S1 Table). Seropositivity against three serovars (Ballum, Cynopteri, and Panama) was observed only in humans, whereas seropositive samples against serovars Manhao and Pyrogenes were observed only in livestock. In general, the MAT titer was higher in humans than in livestock. The most common serovars in all species was serovar Shermani, while serovar Ranarum was found mainly in livestock.

**Fig 2:**
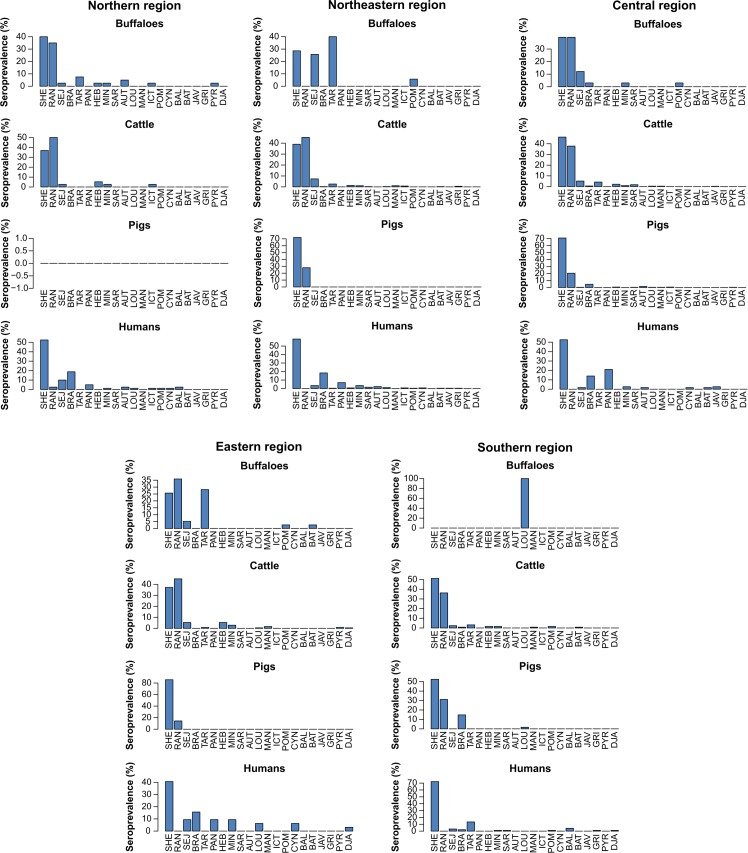
The pattern distribution of serovars for seropositive samples in buffaloes, cattle, pigs, and humans in 5 regions of Thailand. The highest MAT titer reactive against each serovar of each positive sample is described. Samples are ordered according to seroprevalence: Shermani (SHE), Ranarum (RAN), Sejroe (SEJ), Bratislava (BRA), Tarassovi (TAR), Panama (PAN), Hebdomadis (HEB), Mini (MIN), Sarmin (SAR), Autumnalis (AUT), Louisiana (LOU), Manhao (MAN), Icterohaemorrhagiae (ICT), Pomona (POM), Cynopteri (CYN), Ballum (BAL), Bataviae (BAT), Javanica (JAV), Grippotyphosa (GRI), Pyrogenes (PYR), and Djasiman (DJA).

We extracted the information of the predominant serovar that is specific to species and region from S1 Table and illustrated the results in Fig 2. In buffaloes, the MAT titers against the primary serovar were different in each region; high titers were found against serovars Shermani and Ranarum in northern and central regions, against Tarassovi, Shermani, and Sejroe in the northeast, against Ranarum, Tarassovi, and Shermani in the east, and against Louisiana in the south (with only one positive sample). In cattle, the most common serovar titers were against Ranarum and Shermani from all regions, whereas the primary infecting serovar in pigs and humans was serovar Shermani.

### 3.3 Seroprevalence distributions

The distribution of the *Leptospira* serovar seroprevalence in buffaloes differed in composition across regions whereas similar prevalence was found in cattle for all regions (Fig 2). In pigs, the serovar distribution in the central region differed to other regions showing highest serovar diversity (present 11 serovars). It shall be noted that the central region had the highest sample size and seroprevalence in pigs. In the southern region, the serovar distribution in humans differed in comparison to other regions by higher proportions of serovars Shermani and Tarassovi.

### 3.4 Cross-correlation of seroprevalence distribution

**Fig 3:**
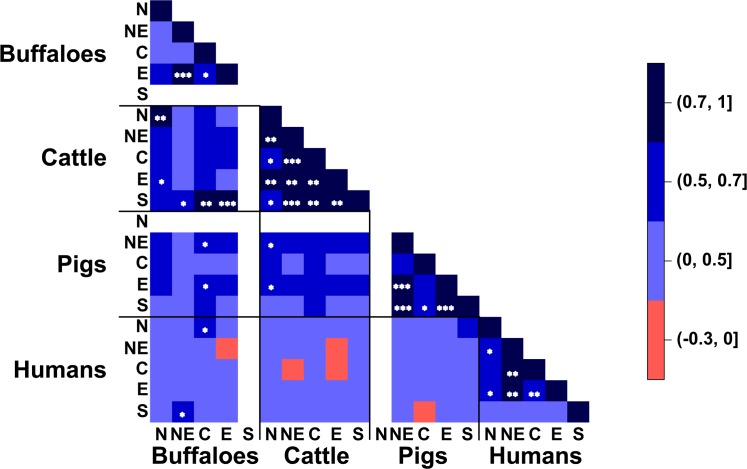
Spearman correlation of the pattern distributions of serovars between buffaloes, cattle, pigs and humans in northern (N), northeastern (NE), central (C), eastern (E), and southern (S) regions. * represents p-value < 0.05, ** represents p-value < 0.01, *** represents p-value < 0.001, and the white area represents correlation not available.

The pairwise correlation in distribution of serovar prevalence (data in Fig 2) across and within both species and regions was determined using the Spearman method with Bonferroni adjustment of P-value (Fig 3). Strongly positive correlations across regions within species were found in buffaloes between northeastern and eastern regions (Spearman’s correlation (cor.) 0.75, 95% confidence interval [0.40–1.00]), in cattle between all regions ranging from cor. 0.70 [0.28–0.90] to cor. 0.82 [0.52–0.97] (except between northern and central, and between northern and southern), in pigs between northeastern and eastern (cor. 0.99 [0.99–1.00]), northeastern and southern (cor. 0.74 [0.49–1.00]), and eastern and southern (cor. 0.74 [05.0–1.00]), and in humans between northeastern and central (cor. 0.70, CI 0.31–0.90), and northeastern and eastern (cor. 0.70 [0.29–0.92]) (all P-values <0.05).

Strongly positive correlations across both regions and species were observed only between buffaloes and cattle and measured 0.71 [0.38–0.99] between both species in the northern region, 0.75 [0.42–0.94] in the central region for buffaloes and in the southern region for cattle and 0.80 [0.51–0.95] for buffaloes in the eastern region and for cattle in southern region. For correlation between livestock and humans, there was a medium positive correlation between buffaloes in the northeastern region and humans in the southern (cor. 0.65 [0.33–0.86]), and buffaloes in the central region and humans in the northern (cor. 0.64 [0.32–0.85]) (all P-values <0.05). A negative correlation was observed between humans and livestock, however being statistically significant.

### 3.5 Logistic regression

We fitted data from all serum samples using the logistic regression model to investigate seropositivity with regard to species and regions. The selection of the best model used a forward stepwise approach based on the Akaike Information Criterion. The best logistic regression model was the univariate model for the serovars Bratislava, Ranarum, and Sejroe (Table 2) and the multivariate model for the serovars Tarassovi and Shermani, and MAT appearance (Table 3). The effect of the region was analyzed in reference to the central region and the effect of the species in reference to humans. The central region was chosen as it is the region for which has the highest number of sample. Thus it is the most powerful disposition to study contrast between regions in reference to the central. For species effect, we would like to highlight the species with higher seroprevalence compared to human.

**Table 2:** The best univariable logistic regression model (with binomial function) for seropositivity of serovars Bratislava (BRA), Ranarum (RAN), and Sejroe (SEJ).

Variable	BRA	RAN	SEJ
	OR (95% CI)	OR (95% CI)	OR (95% CI)
Humans	1.00	-	-
Buffaloes	0.10 (0.03–0.43) ***	-	-
Cattle	0.05 (0.02–0.10) ***	-	-
Pigs	0.17 (0.11–0.27) ***	-	-
Northern	2.22 (1.21–4.05) *	0.50 (0.35–0.71) ***	1.16 (0.59–2.30)
Northeastern	3.26 (2.12–5.01) ***	1.22 (1.02–1.47) *	2.47 (1.64–3.71)***
Central	1.00	1.00	1.00
Eastern	0.73 (0.29–1.87)	2.24 (1.82–2.76) ***	2.61 (1.56–4.34)***
Southern	2.86 (1.52–5.40) **	1.63 (1.25–2.13) ***	1.2 (0.54–2.67)

CI, Confidence Interval.

* significant (P-value < 0.05).

** very significant (P-value < 0.01).

*** highly significant (P-value < 0.001).

**Table 3:** The best multivariable logistic regression model (with binomial function) for seropositivity of serovars Tarassovi (TAR) and Shermani (SHE), and MAT appearance.

Variable	TAR	SHE	MAT
	OR (95% CI)	OR (95% CI)	OR (95% CI)
Humans	1.00	1.00	1.00
Buffaloes	10.76 (5.47–21.17)***	4.72 (2.45–9.11) ***	4.75 (2.61–8.64)***
Cattle	2.26 (1.23–4.14) ***	2.95 (2.21–3.93) ***	2.88 (2.27–3.66) ***
Pigs	n.a.	1.22 (0.91–1.64)	0.92 (0.72–1.18)
Northern	0.23 (0.07–0.76)*	1.41 (0.93–2.14)	1.51 (1.08–2.12)*
Northeastern	0.83 (0.48–1.44)	4.42 (3.19–6.12) ***	4.07 (3.08–5.37) ***
Central	1.00	1.00	1.00
Eastern	1.53 (0.81–2.87)	1.49 (0.79–2.81)	1.39 (0.81–2.38)
Southern	3.57 (1.93–6.62)***	5.02 (3.41–7.39) ***	4.35 (3.09–6.12) ***
Buffaloes:Northern	-	0.49 (0.20–1.24)	0.4 (0.17–0.91) *
Cattle:Northern	-	0.09 (0.05–0.18) ***	0.10 (0.06–0.17) ***
Pigs:Northern	-	n.a.	n.a.
Buffaloes:Northeastern	-	0.03 (0.01–0.08) ***	0.07 (0.03–0.14) ***
Cattle:Northeastern	-	0.16 (0.11–0.24) ***	0.21 (0.15–0.30) ***
Pigs:Northeastern	-	0.18 (0.10–0.32) ***	0.16 (0.09–0.28) ***
Buffaloes:Eastern	-	0.32 (0.11–0.93) *	0.72 (0.29–1.78)
Cattle:Eastern	-	0.95 (0.48–1.88)	1.08 (0.60–1.94)
Pigs:Eastern	-	0.12 (0.04–0.35) ***	0.10 (0.04–0.28) ***
Buffaloes:Southern	-	n.a.	0.01 (0–0.09) ***
Cattle:Southern	-	0.65 (0.39–1.09)	0.59 (0.36–0.95)*
Pigs:Southern	-	0.39 (0.22–0.68) ***	0.51 (0.31–0.84)**

n.a., not available; CI, Confidence Interval.

* significant (P-value < 0.05).

** very significant (P-value < 0.01).

*** highly significant (P-value < 0.001).

The seroprevalence of serovar Bratislava alone in buffaloes (odds ratio (OR) 0.1), cattle (OR 0.05), and pigs (OR 0.17) was significantly (P-value <0.001) lower than in humans. When investigating the seroprevalence according to regions, a higher chance of seropositivity was associated with the northern (OR 2.22, P-value <0.05), northeastern (OR 3.26, P-value <0.001) and southern regions (OR 2.86, P-value <0.01) when compared to the central region. There was no satisfying univariable model for seropositivity against serovars Ranarum and Sejroe associated with species. Seropositivity against serovar Ranarum was more frequent in the northeastern region (OR 1.22, P-value <0.05), eastern (OR 2.24, P-value <0.001), and southern regions (OR 1.63, P-value <0.001) when compared to the northern region (OR 0.5, P-value <0.001). Seropositive samples against serovar Sejroe were significantly (P-value <0.001) more frequent in the northeast (OR 2.47) and the east (OR 2.61).

The results of the multivariable logistic model suggest a significant influence of the type of species on the seroprevalence of serovar Tarassovi. The risk of infection was significantly higher (P-value <0.001) in buffaloes (OR 10.76) and cattle (OR 2.26) compared to humans. Analysis according to region showed a higher risk of infection (OR 3.57, P-value <0.001) in the southern region and a lower risk in the north (OR 0.23, P-value <0.05).

Multivariable logistic model with interaction effects were found to be significant for serovar Shermani and MAT positivity. In general, seropositive samples would be identified for serovar Shermani that are significantly (P-value <0.001) associated with buffaloes (OR 4.72) and cattle (OR 2.95) and also with the northeastern (OR 4.42) and southern regions (OR 5.02). However, a significant interaction effect must be considered. For example, to compare the risk in buffalo in northeastern to the reference (in humans in central), three terms must be multiplied, i.e., OR for buffalo (4.72), OR in northeastern (4.42), and OR for buffalo in northeastern (0.03). So the result of interaction OR equaled 0.64. The overall MAT appearance indicated higher seroprevalence in buffaloes (OR 4.75) and cattle (OR 2.88) as the species effect, and in higher seroprevalence in the northern (OR 1.51), northeastern (OR 4.07) and southern region (OR 4.35) as the region effect. Moreover, the interaction terms must also be considered.

## Discussion

This study provides basic knowledge on serological examination based on a large data set that was gathered by passive surveillance during 2010–2015 among livestock (buffaloes, cattle, and pigs) and humans in Thailand. Anti-*Leptospira* antibodies are prevalent in all 5 regions as well as in livestock and humans. This study is the first to investigate the link between livestock and humans on *Leptospira* serovars endemic in Thailand. This study constitutes an important epidemiological approach and the results may increase comprehension of leptospiral serovar distribution at the regional level. Previous studies suggested that livestock could play an important role as source of human leptospirosis infection [12,28]. High seroprevalence in buffaloes and cattle was also observed, which may increase the exposure level and thus a high risk of infection in humans [12,29].

In this study, we used the MAT to determine seropositivity against a panel of 23 reference serovars including the local ones. As there is no absolute congruency about MAT cut-off titers, we used the recommended cut-off titer ≥ 1:100 [15,20]. MAT is the decade-long gold standard and most commonly used serological test in routine leptospirosis laboratories [30]. However, the test may yield false-negative results and be flawed by cross-reactions [4]. It is also a reported poor predictor of the infecting serovar [31]. However, the MAT provides information about *Leptospira* serogroups circulating in respective species and the immune response of the host.

In livestock, the highest seroprevalence was found in cattle, whereas lowest prevalence occurred in pigs regardless the region. This finding could be explained by the living conditions of pigs leading to a low exposure level to leptospires in the environment due to being caged and/or fenced. In contrast, most cattle are free to move in their environment. Another factor could be the feeding of antibiotics to pigs to prevent leptospirosis infection. In general, pigs also have a shorter life span when compared to cattle and buffaloes leading to shorter exposure time. Regardless of species, the highest seroprevalence was found in southern Thailand and the least was found in the north. This may be attributed to higher rainfall in the south leading to higher exposure to contaminated water and soil [32] even though there are more agricultural areas cultivating rice in northern and northeastern regions.

This study indicates a diversity of *Leptospira* serovars occurring in humans as well as in livestock in all 5 regions of Thailand. In humans, serovar Shermani has the highest seroprevalence, followed by serovars Bratislava, Panama, and Sejroe. Shermani prevalence was also highest in all regions. The high prevalence of serovar Shermani corresponds well with a previous report detecting mainly serovar Shermani in humans [13]. However, the previous study was limited to sample collection in Bangkok. Previously, the patients with a clinical diagnosis of leptospirosis from March 2003 to November 2004 in Thailand were found to positive cultured Leptospira, i.e., *L*. *interrogans* serovar Autumnalis (7), *L*. *interrogans* serovar Bataviae (2), *L*. *interrogans* serovar Pyrogenes (2), *L*. *borgpetersenii* serovar Javanica (1), *L*. *interrogans* serovar Hebdomadis (1), *L*. *interrogans* serovar Grippotyphosa (1), and an unidentified serovar (1) [7]. However, those serovars were not consistence with this study. This may be attributed to the time lag, land use, and environmental factors [10]. The infection by serovar Shermani in humans could be attributed to a high contact rate with livestock or leptospira contaminated environmental sources. Livestock is known to be a host reservoir for leptospires and is potential source of infection for humans [33,34]. Our statistical analysis indicated that livestock were more frequently seropositive for the infecting serovar Shermani than were humans.

We observed low prevalence of antibodies against serovars Bratislava and Sejroe in human samples for all regions. Historically, Bratislava [35–38] and Sejroe [38] were the main infecting serovars in humans across different areas in Thailand. The logistic modeling results with respect to serovar Bratislava prevalence indicated a lower occurrence in livestock when compared to humans. The species effect for serovar Sejroe was found to be insignificant. Sejroe infection could also be attributed to the interaction between rodents, as they are the natural carriers of this serovar in Thailand [39].

Seropositive samples against serovar Panama were detected in humans, particularly in the central region, but not in livestock. High MAT titers against this serovar were also frequently found in the province of Khammouane, Lao PDR [40], in the province of Tien Giang (Mekong delta), Viet Nam [41]. However, associations may need further investigation.

In buffaloes, the most common infecting serovars were Shermani, Ranarum, and Tarassovi. The identification of serovar Ranarum infections corresponds well with a previous study [12], and the seropositivity against serovars Shermani and Tarassovi corresponds to previous positive identifications made in Sakon Nakhon Province, Thailand [42]. The present study suggests that buffaloes could be the maintenance host of the serovars Shermani, Ranarum, and Tarassovi. It shall be noted that a single positive sample was identified in the south. A smaller data set was available for that region.

Serum samples from cattle showed highest MAT titers against serovars Shermani and Ranarum in all regions. Similar to buffaloes, cattle might be a host for those two serovars. The transmission of these serovars between cattle and buffaloes is likely due to shared pastures and water sources. A similar observation of seropositive samples with the other serovars amongst cattle and buffaloes was made in Katavi-Rukwa, Tanzania [33] and in Turkey [43].

Seropositivity against multiple serovars was most common for serovars Ranarum and Shermani in samples from cattle and buffaloes. As a consequence of singlet tests, we were not able to distinguish between cross-reaction and co-infection (sensu stricto or sensu lato). In the case of co-infection sensu stricto, animals may have been infected by different serogroups during the same period [14]. In the case of co-infection sensu lato, animals may have been exposed to a previous serogroup and subsequently exposed to another serogroup resulting in samples positive against multiple serogroups [44]. The later infection may then elicit an immune-response of cross-reactive polyclonal anti-leptospiral antibodies [13,33]. It shall be noted that this is the first study to report seropositive samples against RAN/SHE in Thailand.

In pigs, the predominant infecting serovar was serovar Shermani followed by serovar Ranarum. This study also suggests that pigs can be maintenance hosts of serovar Shermani beside buffaloes and cattle. A great difference in prevalence between serovars Shermani and Ranarum was observed in pigs but not in buffaloes and cattle. We explain this observation with different feeding and living behavior as described above. Serovar Shermani may have a broader distribution with respect to region, while serovar Ranarum occurrence may be limited to grazing grounds and wet lands, which are not the natural habitat of pigs.

Our results highlight that the most abundant infecting serovar is Shermani across humans, buffaloes, cattle, pigs and regions. This suggests a possible transmission pathway between humans and livestock. However, a previous study based on active surveillance [12] found less frequent seropositive samples against serovar Shermani when compared with this study, in particular for livestock. When we compared ratio of serovar Shermani relative to serovar Ranarum between previous study and current one, we obtained an increase of 3.33-fold, 3.62-fold, and 153-fold in buffaloes, cattle, and pigs respectively. Possible reasons are the time lag between the two studies, landscape ecology variations, differences in land use and environmental factors, such as humidity, climate, and animal behavior [10]. Differences in data from livestock between the studies may arise from the individual decision making of farmers to send samples as well as recognition of the pathological conditions of the animals.

The correlation of distributions of *Leptospira* serovar proportions across regions showed a similar pattern for cattle but differed for buffaloes. Cattle have greater commercial relevancy for bush meat production than buffaloes. As a consequence, greater movement of cattle between regions may occurs, which then may lead to similar serovar patterns and higher serovar diversity than in buffaloes. In humans, our results suggested that the infecting serovar distribution in the southern region (high prevalence with 39%) was different to all other regions showing a high proportion of serovars Shermani and Tarassovi. The correlations of titers against the 23 serovars across regions in humans compared to livestock also differed, measuring greater serovar diversity in humans. Some seropositive samples would only be found in humans (serovar Ballum, Cynopteri, and Panama) and some were absent (serovars Manhao and Pyrogenes). The exclusivity of some serovars to human samples could be explained by several other sources of infection, for example the transmission path via rodents.

Indeed, the present study was based on data derived from passive surveillance system. One must interpret the results with caution. Thus, an active surveillance with well sample collection design is also suggested to confirm our passive surveillance to perform to better understand the disease transmission in the field. The size of samples in each species and region could not be controlled and depended on farmers decision making and public health campaigning. The samples in this study were not exclusively collected for leptospiral surveillance. However, this sample size was large enough to illustrate the whole picture of leptospiral infection in livestock in Thailand. Additionally, the samples were collected from different scales of livestock operations. The risk of leptospiral infection was, therefore, different. However, the present study focused on holistic picture of leptospirosis occurrence in livestock at national level regardless types and sizes of the farms. A further study on comparison of leptospirosis in different livestock settings is suggested to elaborate this point.

Logically, animal density should affect epidemiology of leptospirosis. The regions with higher density may pose a higher risk of leptospiral infection. However, this aspect was not focused in our study as the reliable data on animal census in each region of Thailand, have not yet existed. The animal identification system has still been developing. Once the system is fully set up, the study on animal density and risk of leptospiral infection in different regions of Thailand is strongly recommended. Animal movement is also an important factor that may contribute to the exchange of leptospiral serovars among different regions in the country. Nonetheless, animal movement data in Thailand is not publicly accessible. The data is officially hosted by the Department of Livestock Development. The joint research with this institute to visualize the dynamic network of livestock movement is suggested. Subsequently, the study on animal movement and leptospiral distribution along the movement network should be initiated.

Other animal such as wild animal and rodents were not included in this study because we focused on livestock. Wild animals may results in seroprevalence in humans and livestock. Wild animal can be identified as carries of leptospires, which can transfer to humans and livestock [45]. Rodent has been found as reservoirs for human leptospirosis in Thailand especially for *L*. *interrogans* and *L*. *borgpetersenii* species [46]. Rats can be an environmental risk factor by infestation in slums residents in Brazil [47], whereas other mammals can be major reservoir for human leptospirosis, that highlights an importance of leptospiral surveillance beyond rodent species [48]. Other factors such as environmental effects and climate change were also not included. The temporal aspects were not included, this can influence control measures as leptospirosis pattern peak in rainy season and flood events [28]. In fact, the results were analysis based on the interpretation of seropositivity, which indicates a previous exposure to leptospires in the past. The interpretation of occurred infection should be concerned. The age of animals was not recorded in this study. Older animals have more opportunity for leptospires exposure, resulting in an increase of opportunities for seropositivity against multiple serovars [44].

However, leptospiral serovar diversity was observed in humans and livestock. We identified the current most abundant Thailand-endemic leptospiral serovars, foremost serovar Shermani, using MAT to test human and livestock serum samples. Serovar Shermani could be considered a potential public health risk as an emerging serovar occurring at high frequency in humans as well as livestock. The risk of human infection via livestock may be caused directly by contact with an infected animal or indirectly via animal products, mostly contaminated urine [34]. The infection in animal may continue for several months to a year [14]. The public health sector should increase awareness of high-risk groups, in particular abattoir workers, livestock keepers, farmers, and other such individuals with close contact to host and carrier animals. The finding of same serovar distributions may support public health officials in setting up effective intervention and control measures. However, further studies employing molecular typing or real-time PCR are recommended to identify leptospires and confirm the interspecies transmission. A cross-sectional survey should be conducted, especially in abattoirs and animal farms.

## Supporting information

S1 Fig The serovar association distribution in buffaloes and pigs in 5 regions. (TIF)

S2 Fig The serovar association distribution in cattle and humans in 5 regions. (TIF)

S1 Table Distribution of buffaloes, cattle, pigs, and humans exhibiting different MAT titers against *Leptospira* serovarsa. (DOCX)

S2 Table Seroprevalence of serovar association by species and region. (DOCX)

S3 Table Seropositivity by species and region. (PDF)
