# Supplementary figures and images for: Investigation on predominant Leptospira serovars and its distribution in humans and livestock in Thailand, 2010-2015
Source: PLoS Negl Trop Dis. 2017 Feb 9;11(2):e0005228. doi: 10.1371/journal.pntd.0005228 (PMC5325611; doi:10.1371/journal.pntd.0005228)

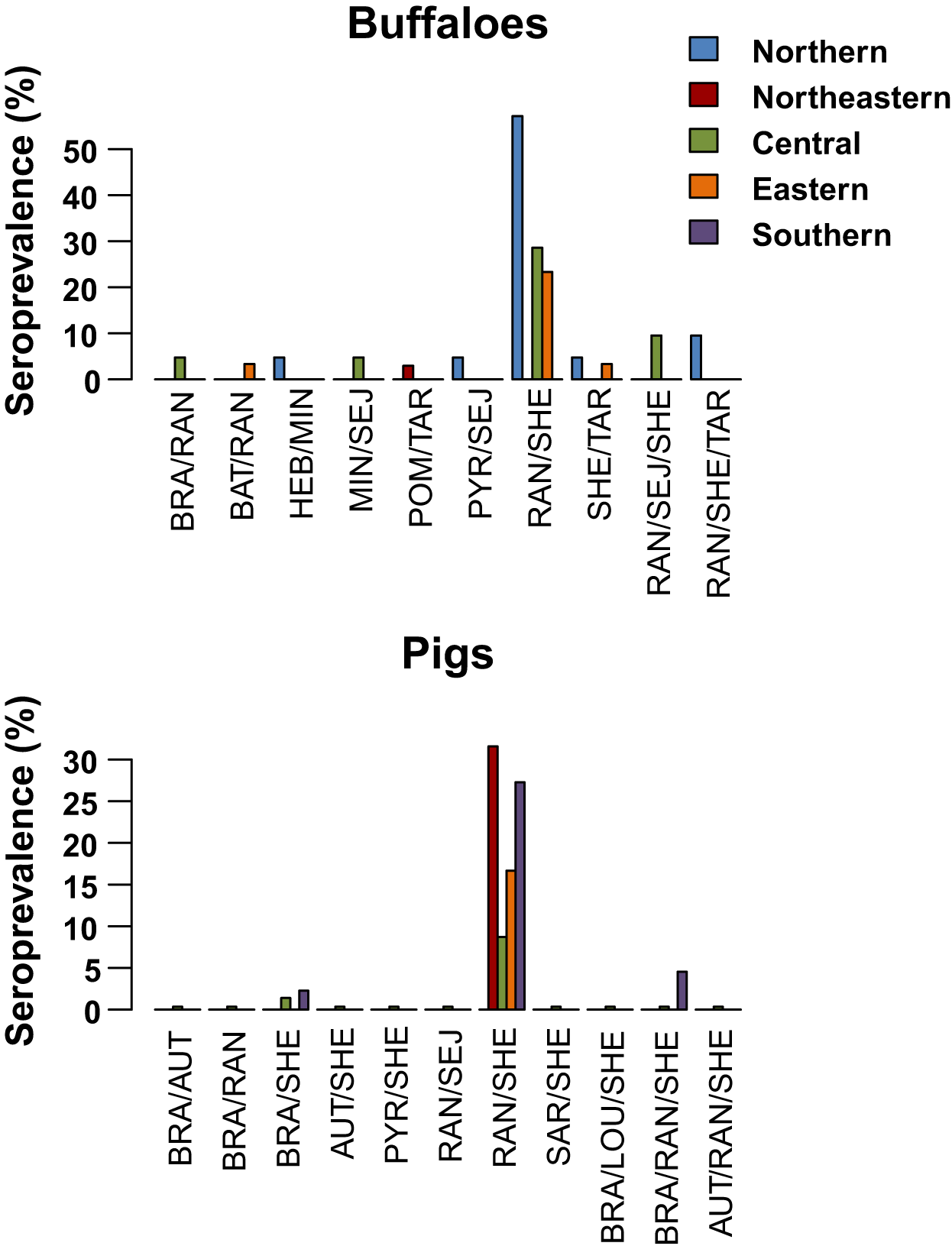

Supplement: S1 Fig — (TIF) [file pntd.0005228.s001.tif]

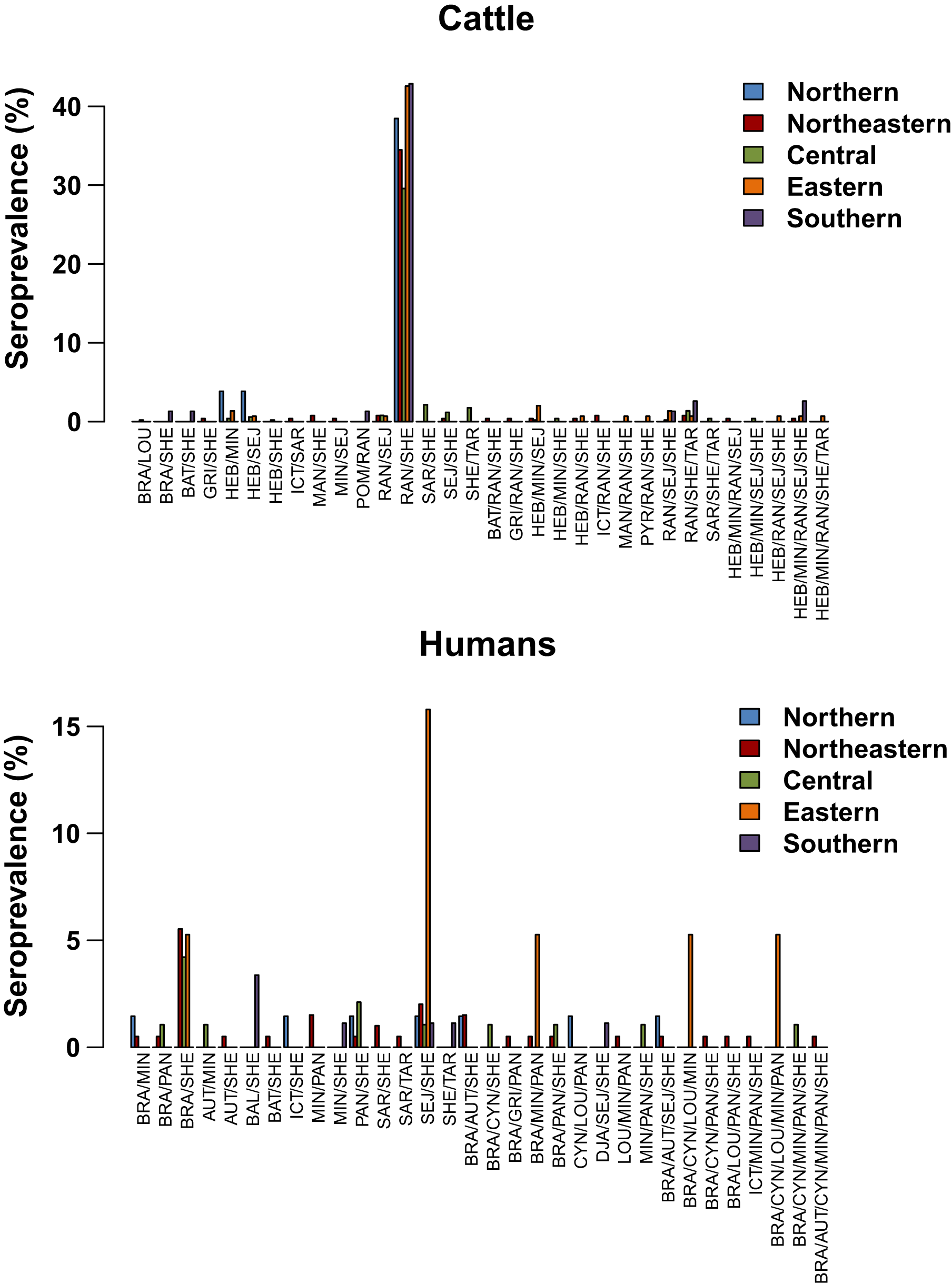

Supplement: S2 Fig — (TIF) [file pntd.0005228.s002.tif]
