# Supplementary material for: Investigation on predominant Leptospira serovars and its distribution in humans and livestock in Thailand, 2010-2015
Source: PLoS Negl Trop Dis. 2017 Feb 9;11(2):e0005228. doi: 10.1371/journal.pntd.0005228 (PMC5325611; doi:10.1371/journal.pntd.0005228)
Supplement: S1 Table — (DOCX) [file pntd.0005228.s003.docx]

**Table S1** Distribution of buffaloes, cattle, pigs, and humans exhibiting different MAT titers against *Leptospira* serovars^a^

| Serovars | Number of samples exhibit MAT titer in (buffaloes, cattle, pigs, human)^b,c^ | | | | | | | | Total number |
| --- | --- | --- | --- | --- | --- | --- | --- | --- | --- |
|  | 100 | 200 | 400 | 800 | 1600 | 3200 | 6400 | 12800 |  |
| Bratislava (BRA) | (0, 5, 20, 8) | (1, 0, 3, 15) | (0, 0, 1, 6) | (0, 1, 0, 13) | (0, 0, 0, 7) | (0, 0, 0, 9) | (0, 0, 0, 25) | (0, 0, 0, 2) | (1, 6, 24, 85) |
| Autumnalis (AUT) | (2, 0, 5, 2) | (0, 0, 0, 1) | (0, 0, 0, 1) | (0, 0, 0, 1) | (0, 0, 0, 1) | (0, 0, 0, 1) | (0, 0, 0, 3) | (0, 0, 0, 0) | (2, 0, 5, 10) |
| Ballum (BAL) | (0, 0, 0, 1) | (0, 0, 0, 0) | (0, 0, 0, 1) | (0, 0, 0, 1) | (0, 0, 0, 1) | (0, 0, 0, 1) | (0, 0, 0, 1) | (0, 0, 0, 0) | (0, 0, 0, 6) |
| Bataviae (BAT) | (1, 1, 0, 1) | (0, 1, 0, 0) | (0, 0, 0, 0) | (0, 0, 0, 0) | (0, 0, 0, 0) | (0, 0, 0, 1) | (0, 0, 0, 1) | (0, 0, 0, 0) | (1, 2, 0, 3) |
| Cynopteri (CYN) | (0, 0, 0, 0) | (0, 0, 0, 0) | (0, 0, 0, 0) | (0, 0, 0, 1) | (0, 0, 0, 0) | (0, 0, 0, 1) | (0, 0, 0, 4) | (0, 0, 0, 1) | (0, 0, 0, 7) |
| Djasiman (DJA) | (0, 1, 0, 0) | (0, 0, 0, 0) | (0, 0, 0, 0) | (0, 0, 0, 1) | (0, 0, 0, 0) | (0, 0, 0, 1) | (0, 0, 0, 0) | (0, 0, 0, 0) | (0, 1, 0, 2) |
| Grippotyphosa (GRI) | (0, 2, 0, 1) | (0, 0, 0, 0) | (0, 0, 0, 0) | (0, 0, 0, 0) | (0, 0, 0, 0) | (0, 0, 0, 0) | (0, 0, 0, 0) | (0, 0, 0, 1) | (0, 2, 0, 2) |
| Hebdomadis (HEB) | (1, 29, 2, 0) | (0, 3, 0, 0) | (0, 5, 0, 0) | (0, 1, 0, 1) | (0, 0, 0, 0) | (0, 0, 0, 0) | (0, 0, 0, 1) | (0, 0, 0, 0) | (1, 38, 2, 2) |
| Icterohaemorrhagiae (ICT) | (0, 4, 1, 0) | (1, 0, 1, 2) | (0, 0, 0, 0) | (0, 0, 0, 0) | (0, 0, 0, 0) | (0, 0, 0, 0) | (0, 0, 0, 1) | (0, 0, 0, 0) | (1, 4, 2, 3) |
| Javanica (JAV) | (0, 2, 0, 1) | (0, 0, 0, 2) | (0, 0, 0, 0) | (0, 0, 0, 1) | (0, 0, 0, 0) | (0, 0, 0, 0) | (0, 0, 0, 0) | (0, 0, 0, 0) | (0, 2, 0, 4) |
| Louisiana (LOU) | (0, 1, 1, 0) | (1, 0, 0, 1) | (0, 1, 1, 0) | (0, 1, 0, 0) | (0, 2, 0, 1) | (0, 0, 0, 1) | (0, 0, 0, 2) | (0, 0, 0, 1) | (1, 5, 2, 6) |
| Manhao (MAN) | (0, 10, 1, 0) | (0, 2, 0, 0) | (0, 0, 0, 0) | (0, 0, 0, 0) | (0, 0, 0, 0) | (0, 0, 0, 0) | (0, 0, 0, 0) | (0, 0, 0, 0) | (0, 12, 1, 0) |
| Mini (MIN) | (2, 17, 0, 1) | (0, 2, 0, 3) | (0, 2, 0, 1) | (0, 0, 0, 1) | (0, 0, 0, 2) | (0, 0, 0, 3) | (0, 0, 0, 5) | (0, 0, 0, 1) | (2, 21, 0, 17) |
| Panama (PAN) | (0, 0, 0, 3) | (0, 0, 0, 4) | (0, 0, 0, 5) | (0, 0, 0, 3) | (0, 0, 0, 6) | (0, 0, 0, 4) | (0, 0, 0, 21) | (0, 0, 0, 3) | (0, 0, 0, 49) |
| Pomona (POM) | (2, 2, 0, 0) | (2, 0, 0, 1) | (0, 0, 0, 0) | (0, 0, 0, 1) | (0, 0, 0, 1) | (0, 0, 0, 0) | (0, 0, 0, 0) | (0, 0, 0, 0) | (4, 2, 0, 3) |
| Pyrogenes (PYR) | (1, 0, 1, 0) | (0, 2, 0, 0) | (0, 0, 0, 0) | (0, 0, 0, 0) | (0, 0, 0, 0) | (0, 0, 0, 0) | (0, 0, 0, 0) | (0, 0, 0, 0) | (1, 2, 1, 0) |
| Ranarum (RAN) | (40, 609, 91, 2) | (1, 16, 3, 0) | (0, 1, 0, 0) | (0, 0, 0, 0) | (0, 0, 0, 0) | (0, 0, 0, 0) | (0, 0, 0, 0) | (0, 0, 0, 0) | (41, 626, 94, 2) |
| Sarmin (SAR) | (0, 14, 1, 1) | (0, 0, 0, 0) | (0, 0, 0, 1) | (0, 0, 0, 1) | (0, 0, 0, 1) | (0, 0, 0, 0) | (0, 0, 0, 1) | (0, 0, 0, 0) | (0, 14, 1, 5) |
| Sejroe (SEJ) | (8, 64, 1, 4) | (6, 10, 0, 8) | (1, 5, 0, 0) | (1, 4, 0, 5) | (0, 0, 0, 3) | (0, 0, 0, 0) | (0, 0, 0, 3) | (0, 0, 0, 2) | (16, 83, 1, 25) |
| Shermani (SHE) | (49, 628, 264, 38) | (0, 26, 22, 53) | (0, 5, 2, 25) | (0, 0, 0, 53) | (0, 0, 0, 32) | (0, 0, 0, 48) | (0, 0, 0, 78) | (0, 0, 0, 7) | (49, 659, 288, 334) |
| Tarassovi (TAR) | (18, 31, 0, 5) | (9, 11, 0, 5) | (1, 3, 0, 3) | (0, 2, 0, 0) | (0, 0, 0, 0) | (0, 0, 0, 0) | (0, 0, 0, 1) | (0, 0, 0, 0) | (28, 47, 0, 14) |

^a^ Negative seroprevalence are not listed in table.

^b^ Highest MAT titer reactive serovars of each positive sample are described.

^c^A total of 432 buffaloes, 3,648 cattle, 3,138 pigs, and 1,990 humans were tested.
