# Supplementary material for: Investigation on predominant Leptospira serovars and its distribution in humans and livestock in Thailand, 2010-2015
Source: PLoS Negl Trop Dis. 2017 Feb 9;11(2):e0005228. doi: 10.1371/journal.pntd.0005228 (PMC5325611; doi:10.1371/journal.pntd.0005228)
Supplement: S2 Table — (DOCX) [file pntd.0005228.s004.docx]

**Table S2** Seroprevalence of serovar association by species and region.

| **Serovar association** |  | **Buffaloes** | | | | | **Cattle** | | | | | **Pigs** | | | | | **Humans** | | | | |
| --- | --- | --- | --- | --- | --- | --- | --- | --- | --- | --- | --- | --- | --- | --- | --- | --- | --- | --- | --- | --- | --- |
|  | **No.Serovars** | **Northern** | **Northeastern** | **Central** | **Eastern** | **Southern** | **Northern** | **Northeastern** | **Central** | **Eastern** | **Southern** | **Northern** | **Northeastern** | **Central** | **Eastern** | **Southern** | **Northern** | **Northeastern** | **Central** | **Eastern** | **Southern** |
| BRA/AUT | 2 | 0 | 0 | 0 | 0 | 0 | 0 | 0 | 0 | 0 | 0 | 0 | 0 | 0.35 | 0 | 0 | 0 | 0 | 0 | 0 | 0 |
| BRA/LOU | 2 | 0 | 0 | 0 | 0 | 0 | 0 | 0 | 0.19 | 0 | 0 | 0 | 0 | 0 | 0 | 0 | 0 | 0 | 0 | 0 | 0 |
| BRA/MIN | 2 | 0 | 0 | 0 | 0 | 0 | 0 | 0 | 0 | 0 | 0 | 0 | 0 | 0 | 0 | 0 | 1.45 | 0.50 | 0 | 0 | 0 |
| BRA/PAN | 2 | 0 | 0 | 0 | 0 | 0 | 0 | 0 | 0 | 0 | 0 | 0 | 0 | 0 | 0 | 0 | 0 | 0.50 | 1.05 | 0 | 0 |
| BRA/RAN | 2 | 0 | 0 | 4.76 | 0 | 0 | 0 | 0 | 0 | 0 | 0 | 0 | 0 | 0.35 | 0 | 0 | 0 | 0 | 0 | 0 | 0 |
| BRA/SHE | 2 | 0 | 0 | 0 | 0 | 0 | 0 | 0 | 0 | 0 | 1.30 | 0 | 0 | 1.39 | 0 | 2.27 | 0 | 5.53 | 4.21 | 5.26 | 0 |
| AUT/MIN | 2 | 0 | 0 | 0 | 0 | 0 | 0 | 0 | 0 | 0 | 0 | 0 | 0 | 0 | 0 | 0 | 0 | 0 | 1.05 | 0 | 0 |
| AUT/SHE | 2 | 0 | 0 | 0 | 0 | 0 | 0 | 0 | 0 | 0 | 0 | 0 | 0 | 0.35 | 0 | 0 | 0 | 0.50 | 0 | 0 | 0 |
| BAL/SHE | 2 | 0 | 0 | 0 | 0 | 0 | 0 | 0 | 0 | 0 | 0 | 0 | 0 | 0 | 0 | 0 | 0 | 0 | 0 | 0 | 3.37 |
| BAT/RAN | 2 | 0 | 0 | 0 | 3.33 | 0 | 0 | 0 | 0 | 0 | 0 | 0 | 0 | 0 | 0 | 0 | 0 | 0 | 0 | 0 | 0 |
| BAT/SHE | 2 | 0 | 0 | 0 | 0 | 0 | 0 | 0 | 0 | 0 | 1.30 | 0 | 0 | 0 | 0 | 0 | 0 | 0.50 | 0 | 0 | 0 |
| GRI/SHE | 2 | 0 | 0 | 0 | 0 | 0 | 0 | 0.38 | 0 | 0 | 0 | 0 | 0 | 0 | 0 | 0 | 0 | 0 | 0 | 0 | 0 |
| HEB/MIN | 2 | 4.76 | 0 | 0 | 0 | 0 | 3.85 | 0 | 0.39 | 1.35 | 0 | 0 | 0 | 0 | 0 | 0 | 0 | 0 | 0 | 0 | 0 |
| HEB/SEJ | 2 | 0 | 0 | 0 | 0 | 0 | 3.85 | 0 | 0.58 | 0.68 | 0 | 0 | 0 | 0 | 0 | 0 | 0 | 0 | 0 | 0 | 0 |
| HEB/SHE | 2 | 0 | 0 | 0 | 0 | 0 | 0 | 0 | 0.19 | 0 | 0 | 0 | 0 | 0 | 0 | 0 | 0 | 0 | 0 | 0 | 0 |
| ICT/SAR | 2 | 0 | 0 | 0 | 0 | 0 | 0 | 0.38 | 0 | 0 | 0 | 0 | 0 | 0 | 0 | 0 | 0 | 0 | 0 | 0 | 0 |
| ICT/SHE | 2 | 0 | 0 | 0 | 0 | 0 | 0 | 0 | 0 | 0 | 0 | 0 | 0 | 0 | 0 | 0 | 1.45 | 0 | 0 | 0 | 0 |
| MAN/SHE | 2 | 0 | 0 | 0 | 0 | 0 | 0 | 0.77 | 0 | 0 | 0 | 0 | 0 | 0 | 0 | 0 | 0 | 0 | 0 | 0 | 0 |
| MIN/PAN | 2 | 0 | 0 | 0 | 0 | 0 | 0 | 0 | 0 | 0 | 0 | 0 | 0 | 0 | 0 | 0 | 0 | 1.51 | 0 | 0 | 0 |
| MIN/SEJ | 2 | 0 | 0 | 4.76 | 0 | 0 | 0 | 0.38 | 0 | 0 | 0 | 0 | 0 | 0 | 0 | 0 | 0 | 0 | 0 | 0 | 0 |
| MIN/SHE | 2 | 0 | 0 | 0 | 0 | 0 | 0 | 0 | 0 | 0 | 0 | 0 | 0 | 0 | 0 | 0 | 0 | 0 | 0 | 0 | 1.12 |
| PAN/SHE | 2 | 0 | 0 | 0 | 0 | 0 | 0 | 0 | 0 | 0 | 0 | 0 | 0 | 0 | 0 | 0 | 1.45 | 0.50 | 2.11 | 0 | 0 |
| POM/RAN | 2 | 0 | 0 | 0 | 0 | 0 | 0 | 0 | 0 | 0 | 1.30 | 0 | 0 | 0 | 0 | 0 | 0 | 0 | 0 | 0 | 0 |
| POM/TAR | 2 | 0 | 2.94 | 0 | 0 | 0 | 0 | 0 | 0 | 0 | 0 | 0 | 0 | 0 | 0 | 0 | 0 | 0 | 0 | 0 | 0 |
| PYR/SEJ | 2 | 4.76 | 0 | 0 | 0 | 0 | 0 | 0 | 0 | 0 | 0 | 0 | 0 | 0 | 0 | 0 | 0 | 0 | 0 | 0 | 0 |
| PYR/SHE | 2 | 0 | 0 | 0 | 0 | 0 | 0 | 0 | 0 | 0 | 0 | 0 | 0 | 0.35 | 0 | 0 | 0 | 0 | 0 | 0 | 0 |
| RAN/SEJ | 2 | 0 | 0 | 0 | 0 | 0 | 0 | 0.77 | 0.78 | 0.68 | 0 | 0 | 0 | 0.35 | 0 | 0 | 0 | 0 | 0 | 0 | 0 |
| RAN/SHE | 2 | 57.14 | 0 | 28.57 | 23.33 | 0 | 38.46 | 34.48 | 29.57 | 42.57 | 42.86 | 0 | 31.58 | 8.71 | 16.67 | 27.27 | 0 | 0 | 0 | 0 | 0 |
| SAR/SHE | 2 | 0 | 0 | 0 | 0 | 0 | 0 | 0 | 2.14 | 0 | 0 | 0 | 0 | 0.35 | 0 | 0 | 0 | 1.01 | 0 | 0 | 0 |
| SAR/TAR | 2 | 0 | 0 | 0 | 0 | 0 | 0 | 0 | 0 | 0 | 0 | 0 | 0 | 0 | 0 | 0 | 0 | 0.50 | 0 | 0 | 0 |
| SEJ/SHE | 2 | 0 | 0 | 0 | 0 | 0 | 0 | 0.38 | 1.17 | 0 | 0 | 0 | 0 | 0 | 0 | 0 | 1.45 | 2.01 | 1.05 | 15.79 | 1.12 |
| SHE/TAR | 2 | 4.76 | 0 | 0 | 3.33 | 0 | 0 | 0 | 1.75 | 0 | 0 | 0 | 0 | 0 | 0 | 0 | 0 | 0 | 0 | 0 | 1.12 |
| BRA/AUT/SHE | 3 | 0 | 0 | 0 | 0 | 0 | 0 | 0 | 0 | 0 | 0 | 0 | 0 | 0 | 0 | 0 | 1.45 | 1.51 | 0 | 0 | 0 |
| BRA/CYN/SHE | 3 | 0 | 0 | 0 | 0 | 0 | 0 | 0 | 0 | 0 | 0 | 0 | 0 | 0 | 0 | 0 | 0 | 0 | 1.05 | 0 | 0 |
| BRA/GRI/PAN | 3 | 0 | 0 | 0 | 0 | 0 | 0 | 0 | 0 | 0 | 0 | 0 | 0 | 0 | 0 | 0 | 0 | 0.50 | 0 | 0 | 0 |
| BRA/LOU/SHE | 3 | 0 | 0 | 0 | 0 | 0 | 0 | 0 | 0 | 0 | 0 | 0 | 0 | 0.35 | 0 | 0 | 0 | 0 | 0 | 0 | 0 |
| BRA/MIN/PAN | 3 | 0 | 0 | 0 | 0 | 0 | 0 | 0 | 0 | 0 | 0 | 0 | 0 | 0 | 0 | 0 | 0 | 0.50 | 0 | 5.26 | 0 |
| BRA/PAN/SHE | 3 | 0 | 0 | 0 | 0 | 0 | 0 | 0 | 0 | 0 | 0 | 0 | 0 | 0 | 0 | 0 | 0 | 0.50 | 1.05 | 0 | 0 |
| BRA/RAN/SHE | 3 | 0 | 0 | 0 | 0 | 0 | 0 | 0 | 0 | 0 | 0 | 0 | 0 | 0.35 | 0 | 4.55 | 0 | 0 | 0 | 0 | 0 |
| AUT/RAN/SHE | 3 | 0 | 0 | 0 | 0 | 0 | 0 | 0 | 0 | 0 | 0 | 0 | 0 | 0.35 | 0 | 0 | 0 | 0 | 0 | 0 | 0 |
| BAT/RAN/SHE | 3 | 0 | 0 | 0 | 0 | 0 | 0 | 0.38 | 0 | 0 | 0 | 0 | 0 | 0 | 0 | 0 | 0 | 0 | 0 | 0 | 0 |
| CYN/LOU/PAN | 3 | 0 | 0 | 0 | 0 | 0 | 0 | 0 | 0 | 0 | 0 | 0 | 0 | 0 | 0 | 0 | 1.45 | 0 | 0 | 0 | 0 |
| DJA/SEJ/SHE | 3 | 0 | 0 | 0 | 0 | 0 | 0 | 0 | 0 | 0 | 0 | 0 | 0 | 0 | 0 | 0 | 0 | 0 | 0 | 0 | 1.12 |
| GRI/RAN/SHE | 3 | 0 | 0 | 0 | 0 | 0 | 0 | 0.38 | 0 | 0 | 0 | 0 | 0 | 0 | 0 | 0 | 0 | 0 | 0 | 0 | 0 |
| HEB/MIN/SEJ | 3 | 0 | 0 | 0 | 0 | 0 | 0 | 0.38 | 0.19 | 2.03 | 0 | 0 | 0 | 0 | 0 | 0 | 0 | 0 | 0 | 0 | 0 |
| HEB/MIN/SHE | 3 | 0 | 0 | 0 | 0 | 0 | 0 | 0 | 0.39 | 0 | 0 | 0 | 0 | 0 | 0 | 0 | 0 | 0 | 0 | 0 | 0 |
| HEB/RAN/SHE | 3 | 0 | 0 | 0 | 0 | 0 | 0 | 0.38 | 0 | 0.68 | 0 | 0 | 0 | 0 | 0 | 0 | 0 | 0 | 0 | 0 | 0 |
| ICT/RAN/SHE | 3 | 0 | 0 | 0 | 0 | 0 | 0 | 0.77 | 0 | 0 | 0 | 0 | 0 | 0 | 0 | 0 | 0 | 0 | 0 | 0 | 0 |
| LOU/MIN/PAN | 3 | 0 | 0 | 0 | 0 | 0 | 0 | 0 | 0 | 0 | 0 | 0 | 0 | 0 | 0 | 0 | 0 | 0.50 | 0 | 0 | 0 |
| MAN/RAN/SHE | 3 | 0 | 0 | 0 | 0 | 0 | 0 | 0 | 0 | 0.68 | 0 | 0 | 0 | 0 | 0 | 0 | 0 | 0 | 0 | 0 | 0 |
| MIN/PAN/SHE | 3 | 0 | 0 | 0 | 0 | 0 | 0 | 0 | 0 | 0 | 0 | 0 | 0 | 0 | 0 | 0 | 0 | 0 | 1.05 | 0 | 0 |
| PYR/RAN/SHE | 3 | 0 | 0 | 0 | 0 | 0 | 0 | 0 | 0 | 0.68 | 0 | 0 | 0 | 0 | 0 | 0 | 0 | 0 | 0 | 0 | 0 |
| RAN/SEJ/SHE | 3 | 0 | 0 | 9.52 | 0 | 0 | 0 | 0 | 0.19 | 1.35 | 1.30 | 0 | 0 | 0 | 0 | 0 | 0 | 0 | 0 | 0 | 0 |
| RAN/SHE/TAR | 3 | 9.52 | 0 | 0 | 0 | 0 | 0 | 0.77 | 1.36 | 0.68 | 2.60 | 0 | 0 | 0 | 0 | 0 | 0 | 0 | 0 | 0 | 0 |
| SAR/SHE/TAR | 3 | 0 | 0 | 0 | 0 | 0 | 0 | 0 | 0.39 | 0 | 0 | 0 | 0 | 0 | 0 | 0 | 0 | 0 | 0 | 0 | 0 |
| BRA/AUT/SEJ/SHE | 4 | 0 | 0 | 0 | 0 | 0 | 0 | 0 | 0 | 0 | 0 | 0 | 0 | 0 | 0 | 0 | 1.45 | 0.50 | 0 | 0 | 0 |
| BRA/CYN/LOU/MIN | 4 | 0 | 0 | 0 | 0 | 0 | 0 | 0 | 0 | 0 | 0 | 0 | 0 | 0 | 0 | 0 | 0 | 0 | 0 | 5.26 | 0 |
| BRA/CYN/PAN/SHE | 4 | 0 | 0 | 0 | 0 | 0 | 0 | 0 | 0 | 0 | 0 | 0 | 0 | 0 | 0 | 0 | 0 | 0.50 | 0 | 0 | 0 |
| BRA/LOU/PAN/SHE | 4 | 0 | 0 | 0 | 0 | 0 | 0 | 0 | 0 | 0 | 0 | 0 | 0 | 0 | 0 | 0 | 0 | 0.50 | 0 | 0 | 0 |
| HEB/MIN/RAN/SEJ | 4 | 0 | 0 | 0 | 0 | 0 | 0 | 0.38 | 0 | 0 | 0 | 0 | 0 | 0 | 0 | 0 | 0 | 0 | 0 | 0 | 0 |
| HEB/MIN/SEJ/SHE | 4 | 0 | 0 | 0 | 0 | 0 | 0 | 0 | 0.39 | 0 | 0 | 0 | 0 | 0 | 0 | 0 | 0 | 0 | 0 | 0 | 0 |
| HEB/RAN/SEJ/SHE | 4 | 0 | 0 | 0 | 0 | 0 | 0 | 0 | 0 | 0.68 | 0 | 0 | 0 | 0 | 0 | 0 | 0 | 0 | 0 | 0 | 0 |
| ICT/MIN/PAN/SHE | 4 | 0 | 0 | 0 | 0 | 0 | 0 | 0 | 0 | 0 | 0 | 0 | 0 | 0 | 0 | 0 | 0 | 0.50 | 0 | 0 | 0 |
| BRA/CYN/LOU/MIN/PAN | 5 | 0 | 0 | 0 | 0 | 0 | 0 | 0 | 0 | 0 | 0 | 0 | 0 | 0 | 0 | 0 | 0 | 0 | 0 | 5.26 | 0 |
| BRA/CYN/MIN/PAN/SHE | 5 | 0 | 0 | 0 | 0 | 0 | 0 | 0 | 0 | 0 | 0 | 0 | 0 | 0 | 0 | 0 | 0 | 0 | 1.05 | 0 | 0 |
| HEB/MIN/RAN/SEJ/SHE | 5 | 0 | 0 | 0 | 0 | 0 | 0 | 0.38 | 0 | 0.68 | 2.60 | 0 | 0 | 0 | 0 | 0 | 0 | 0 | 0 | 0 | 0 |
| HEB/MIN/RAN/SHE/TAR | 5 | 0 | 0 | 0 | 0 | 0 | 0 | 0 | 0 | 0.68 | 0 | 0 | 0 | 0 | 0 | 0 | 0 | 0 | 0 | 0 | 0 |
| BRA/AUT/CYN/MIN/PAN/SHE | 6 | 0 | 0 | 0 | 0 | 0 | 0 | 0 | 0 | 0 | 0 | 0 | 0 | 0 | 0 | 0 | 0 | 0.50 | 0 | 0 | 0 |
