# Supplementary material for: Investigation on predominant Leptospira serovars and its distribution in humans and livestock in Thailand, 2010-2015
Source: PLoS Negl Trop Dis. 2017 Feb 9;11(2):e0005228. doi: 10.1371/journal.pntd.0005228 (PMC5325611; doi:10.1371/journal.pntd.0005228)
Supplement: S3 Table — (PDF) [file pntd.0005228.s005.pdf]

Label	SPECIES	Region	Bratislava	Sejroe	Ranarum	Shermani	Tarassovi	MAT
1	Human	Eastern	0	0	0	0	0	0
2	Human	Eastern	0	0	0	0	0	0
3	Human	Central	1	0	0	0	0	1
4	Human	Eastern	1	0	0	0	0	1
5	Human	Southern	0	0	0	0	0	0
6	Human	Southern	0	0	0	0	0	0
7	Human	Southern	0	1	0	0	0	1
8	Human	Southern	0	0	0	0	0	0
9	Human	Southern	0	0	0	0	0	0
10	Human	Central	0	0	0	0	0	0
11	Human	Central	0	0	0	1	0	1
12	Human	Central	0	0	0	0	0	0
13	Human	Central	0	0	0	0	0	0
14	Human	Central	0	0	0	0	0	0
15	Human	Central	0	0	0	0	0	0
16	Human	Northeastern	0	0	0	0	0	0
17	Human	Northeastern	0	0	0	0	0	0
18	Human	Eastern	0	0	0	0	0	0
19	Human	Central	0	0	0	0	0	0
20	Human	Central	0	0	0	0	0	0
21	Human	Central	0	0	0	0	0	0
22	Human	Central	0	0	0	0	0	0
23	Human	Northeastern	0	0	0	1	0	1
24	Human	Northeastern	0	0	0	1	0	1
25	Human	Northeastern	1	0	0	0	0	1
26	Human	Northeastern	0	0	0	0	0	0
27	Human	Northeastern	0	0	0	1	0	1
28	Human	Northeastern	0	0	0	0	0	0
29	Human	Northeastern	0	0	0	1	0	1
30	Human	Northeastern	0	0	0	1	0	1
31	Human	Northeastern	1	0	0	1	0	1
32	Human	Northeastern	0	0	0	0	0	0
33	Human	Northeastern	0	1	0	0	0	1
34	Human	Northeastern	0	0	0	1	0	1
35	Human	Northeastern	0	0	0	0	1	1
36	Human	Northeastern	0	0	0	0	0	0
37	Human	Northeastern	0	0	0	0	0	1
38	Human	Northeastern	0	0	0	1	0	1
39	Human	Northeastern	1	0	0	0	0	1
40	Human	Northeastern	1	0	0	0	0	1
41	Human	Northeastern	1	0	0	1	0	1
42	Human	Northeastern	0	0	0	1	0	1
43	Human	Central	0	0	0	1	0	1
44	Human	Central	0	0	0	0	0	0
45	Human	Central	0	0	0	0	0	0
46	Human	Central	0	0	0	0	0	0
47	Human	Central	0	0	0	0	0	0

48	Human	Northern	0	0	0	0	0	0
49	Human	Northern	0	0	0	0	0	0
50	Human	Northern	0	0	0	1	0	1
51	Human	Northern	0	0	0	0	0	0
52	Human	Northern	0	0	0	0	0	0
53	Human	Northern	0	0	0	0	0	0
54	Human	Northern	0	0	0	0	0	0
55	Human	Northern	0	0	0	0	0	0
56	Human	Northern	0	0	0	0	0	0
57	Human	Northern	0	0	0	0	0	0
58	Human	Northern	0	0	0	0	0	0
59	Human	Northern	0	0	0	0	0	0
60	Human	Northern	0	0	0	0	0	0
61	Human	Northern	0	0	0	0	0	0
62	Human	Northern	0	0	0	0	0	0
63	Human	Northern	0	0	0	0	0	0
64	Human	Northern	0	0	0	0	0	0
65	Human	Northern	0	0	0	0	0	0
66	Human	Northern	0	0	0	0	0	0
67	Human	Northern	0	0	0	1	0	1
68	Human	Northern	0	0	0	1	0	1
69	Human	Northern	0	0	0	0	0	0
70	Human	Northern	0	0	0	1	0	1
71	Human	Northern	0	0	0	0	0	0
72	Human	Northern	0	0	0	0	0	0
73	Human	Northern	0	0	0	0	0	0
74	Human	Northern	0	0	0	0	0	0
75	Human	Northern	0	0	0	1	0	1
76	Human	Northern	0	1	0	0	0	1
77	Human	Northern	0	0	0	0	0	0
78	Human	Northern	0	0	0	0	0	0
79	Human	Northern	0	0	0	0	0	0
80	Human	Northern	0	0	0	0	0	0
81	Human	Northern	0	0	0	0	0	0
82	Human	Northern	0	0	0	0	0	0
83	Human	Northern	0	0	0	0	0	0
84	Human	Northern	0	0	0	0	0	0
85	Human	Northern	0	1	0	1	0	1
86	Human	Northern	0	0	0	0	0	0
87	Human	Northern	0	0	0	0	0	0
88	Human	Northern	0	0	0	0	0	0
89	Human	Northern	0	0	0	0	0	0
90	Human	Northern	0	0	0	0	0	0
91	Human	Central	0	0	0	0	0	0
92	Human	Northeastern	0	0	0	0	0	0
93	Human	Northeastern	0	0	0	0	0	0
94	Human	Northeastern	0	0	0	0	0	0
95	Human	Northeastern	0	0	0	0	0	0

96	Human	Northeastern	1	0	0	1	0	1
97	Human	Northeastern	0	0	0	0	0	0
98	Human	Northeastern	0	0	0	0	0	0
99	Human	Northeastern	0	0	0	0	0	0
100	Human	Northeastern	0	0	0	0	0	0
101	Human	Northeastern	0	0	0	0	0	0
102	Human	Northeastern	0	0	0	0	0	0
103	Human	Northeastern	0	0	0	0	0	0
104	Human	Northeastern	0	0	0	0	0	0
105	Human	Northeastern	0	0	0	0	0	0
106	Human	Northeastern	0	0	0	0	0	0
107	Human	Northeastern	1	0	0	0	0	1
108	Human	Northeastern	1	0	0	0	0	1
109	Human	Northeastern	0	0	0	0	0	0
110	Human	Northeastern	0	0	0	0	0	0
111	Human	Northeastern	0	0	0	0	0	0
112	Human	Northeastern	0	0	0	0	0	0
113	Human	Northeastern	0	0	0	0	0	0
114	Human	Northeastern	0	0	0	0	0	0
115	Human	Northeastern	0	0	0	0	0	0
116	Human	Northeastern	0	0	0	0	0	1
117	Human	Northeastern	0	0	0	0	0	0
118	Human	Northeastern	0	0	0	0	0	0
119	Human	Northeastern	0	0	0	0	0	0
120	Human	Northeastern	0	0	0	0	0	0
121	Human	Northeastern	0	0	0	0	0	0
122	Human	Northeastern	0	0	0	0	0	0
123	Human	Northeastern	0	0	0	0	0	0
124	Human	Northeastern	0	0	0	0	0	0
125	Human	Northeastern	0	0	0	0	0	0
126	Human	Northeastern	0	0	0	0	0	0
127	Human	Northeastern	0	0	0	0	0	0
128	Human	Northeastern	0	0	0	0	0	0
129	Human	Northeastern	0	0	0	0	0	0
130	Human	Northeastern	0	0	0	0	0	0
131	Human	Northeastern	0	0	0	0	0	0
132	Human	Northeastern	1	0	0	1	0	1
133	Human	Northeastern	0	0	0	0	0	0
134	Human	Northeastern	0	0	0	0	0	0
135	Human	Northeastern	0	0	0	0	0	0
136	Human	Northeastern	0	0	0	1	0	1
137	Human	Northeastern	0	0	0	1	0	1
138	Human	Northeastern	0	0	0	0	0	0
139	Human	Northeastern	0	0	0	0	0	0
140	Human	Northeastern	0	0	0	0	0	0
141	Human	Northeastern	1	0	0	1	0	1
142	Human	Northeastern	0	0	0	0	0	0
143	Human	Northeastern	0	0	0	0	0	0

144	Human	Northeastern	0	0	0	0	0	0
145	Human	Northeastern	0	0	0	0	0	0
146	Human	Northeastern	0	0	0	0	0	0
147	Human	Northeastern	0	0	0	1	0	1
148	Human	Southern	0	0	0	0	0	0
149	Human	Central	0	0	0	0	0	0
150	Human	Central	0	0	0	0	0	0
151	Human	Central	0	0	0	0	0	1
152	Human	Central	0	0	0	0	0	0
153	Human	Eastern	0	0	0	0	0	0
154	Human	Central	0	0	0	0	0	0
155	Human	Central	0	0	0	0	0	0
156	Human	Central	0	0	0	0	0	0
157	Human	Central	0	0	0	0	0	0
158	Human	Eastern	0	0	0	1	0	1
159	Human	Southern	0	0	0	0	0	0
160	Human	Central	0	0	0	0	0	0
161	Human	Central	0	0	0	0	0	0
162	Human	Northeastern	0	0	0	0	0	0
163	Human	Northeastern	0	0	0	0	0	0
164	Human	Northeastern	0	0	0	0	0	0
165	Human	Northeastern	0	0	0	1	0	1
166	Human	Northeastern	0	0	0	1	0	1
167	Human	Northeastern	0	0	0	0	0	0
168	Human	Northeastern	0	0	0	0	0	0
169	Human	Northeastern	0	0	0	0	0	0
170	Human	Northeastern	0	0	0	1	0	1
171	Human	Northeastern	0	0	0	0	0	0
172	Human	Northeastern	0	0	0	1	0	1
173	Human	Northeastern	0	0	0	0	0	0
174	Human	Northeastern	0	0	0	0	0	0
175	Human	Northeastern	0	0	0	0	0	0
176	Human	Northeastern	0	0	0	0	0	0
177	Human	Northeastern	0	0	0	0	0	0
178	Human	Northeastern	0	0	0	0	0	0
179	Human	Northeastern	0	0	0	0	0	0
180	Human	Northeastern	0	0	0	0	0	0
181	Human	Northeastern	0	0	0	1	0	1
182	Human	Northeastern	0	0	0	1	0	1
183	Human	Northeastern	0	0	0	0	0	0
184	Human	Northeastern	0	0	0	0	0	0
185	Human	Northeastern	0	0	0	0	0	0
186	Human	Northeastern	0	0	0	0	0	0
187	Human	Northeastern	0	1	0	1	0	1
188	Human	Northeastern	0	0	0	1	0	1
189	Human	Northeastern	0	0	0	0	0	0
190	Human	Northeastern	0	0	0	0	0	0
191	Human	Northeastern	0	0	0	1	0	1

192	Human	Northeastern	0	0	0	1	0	1
193	Human	Northeastern	0	0	0	1	0	1
194	Human	Northeastern	0	0	0	1	0	1
195	Human	Northeastern	0	0	0	0	0	0
196	Human	Northeastern	0	0	0	0	0	0
197	Human	Northeastern	0	0	0	0	0	0
198	Human	Northeastern	0	0	0	0	0	0
199	Human	Northeastern	0	0	0	0	0	0
200	Human	Northeastern	0	0	0	0	0	0
201	Human	Northeastern	0	0	0	0	0	0
202	Human	Northeastern	0	0	0	0	0	0
203	Human	Northeastern	0	0	0	0	0	0
204	Human	Northeastern	0	0	0	0	0	0
205	Human	Northeastern	1	0	0	0	0	1
206	Human	Northeastern	1	0	0	0	0	1
207	Human	Northeastern	1	0	0	1	0	1
208	Human	Northeastern	0	0	0	1	0	1
209	Human	Northeastern	0	0	0	0	0	0
210	Human	Northeastern	0	0	0	0	0	0
211	Human	Northeastern	0	0	0	0	0	0
212	Human	Northeastern	0	0	0	1	0	1
213	Human	Northeastern	0	0	0	1	0	1
214	Human	Northeastern	0	0	0	0	0	0
215	Human	Northeastern	0	0	0	0	0	0
216	Human	Northeastern	0	0	0	0	0	0
217	Human	Northeastern	0	0	0	0	0	0
218	Human	Northeastern	0	0	0	0	0	0
219	Human	Northeastern	0	0	0	0	0	0
220	Human	Northeastern	0	0	0	0	0	0
221	Human	Northeastern	0	0	0	0	0	0
222	Human	Northeastern	1	0	0	1	0	1
223	Human	Northeastern	0	0	0	1	0	1
224	Human	Central	0	0	0	0	0	0
225	Human	Central	0	0	0	0	0	0
226	Human	Central	0	0	0	0	0	0
227	Human	Central	0	0	0	0	0	0
228	Human	Southern	0	0	0	1	0	1
229	Human	Southern	0	0	0	1	0	1
230	Human	Southern	0	0	0	1	0	1
231	Human	Southern	0	0	0	0	0	1
232	Human	Southern	0	0	0	1	0	1
233	Human	Central	0	0	0	0	0	0
234	Human	Central	0	0	0	1	0	1
235	Human	Central	0	0	0	0	0	0
236	Human	Central	0	0	0	0	0	0
237	Human	Central	0	0	0	0	0	0
238	Human	Central	0	0	0	0	0	0
239	Human	Central	0	0	0	0	0	0

240	Human	Central	0	0	0	0	0	0
241	Human	Central	0	0	0	0	0	0
242	Human	Central	0	0	0	0	0	0
243	Human	Central	0	0	0	0	0	0
244	Human	Central	0	0	0	0	0	0
245	Human	Central	0	0	0	0	0	0
246	Human	Central	0	0	0	1	0	1
247	Human	Central	0	0	0	1	0	1
248	Human	Central	1	0	0	0	0	1
249	Human	Central	0	0	0	1	0	1
250	Human	Central	0	0	0	1	0	1
251	Human	Central	0	0	0	1	0	1
252	Human	Southern	0	0	0	1	0	1
253	Human	Southern	0	0	0	1	0	1
254	Human	Southern	0	0	0	1	0	1
255	Human	Southern	0	0	0	1	0	1
256	Human	Southern	0	0	0	1	0	1
257	Human	Southern	0	0	0	1	0	1
258	Human	Southern	0	0	0	1	0	1
259	Human	Southern	0	0	0	1	0	1
260	Human	Southern	0	0	0	1	0	1
261	Human	Southern	0	0	0	1	0	1
262	Human	Southern	0	0	0	1	0	1
263	Human	Southern	0	0	0	1	0	1
264	Human	Southern	0	0	0	1	0	1
265	Human	Southern	0	0	0	1	0	1
266	Human	Southern	0	0	0	1	0	1
267	Human	Southern	0	0	0	1	0	1
268	Human	Southern	0	0	0	1	0	1
269	Human	Southern	0	0	0	1	0	1
270	Human	Southern	0	0	0	1	0	1
271	Human	Southern	0	0	0	1	0	1
272	Human	Southern	0	0	0	1	0	1
273	Human	Southern	0	0	0	1	0	1
274	Human	Southern	0	0	0	1	0	1
275	Human	Southern	0	0	0	1	0	1
276	Human	Southern	0	0	0	1	0	1
277	Human	Southern	0	0	0	1	0	1
278	Human	Southern	0	0	0	1	0	1
279	Human	Southern	0	0	0	1	0	1
280	Human	Southern	0	0	0	1	0	1
281	Human	Southern	0	0	0	1	0	1
282	Human	Southern	0	0	0	1	0	1
283	Human	Southern	0	0	0	1	0	1
284	Human	Southern	0	0	0	1	0	1
285	Human	Southern	0	0	0	1	0	1
286	Human	Southern	0	0	0	1	0	1
287	Human	Southern	0	0	0	1	0	1

288	Human	Southern	0	0	0	1	0	1
289	Human	Southern	0	0	0	1	0	1
290	Human	Southern	0	0	0	1	0	1
291	Human	Southern	0	0	0	1	0	1
292	Human	Southern	0	0	0	1	0	1
293	Human	Southern	0	0	0	1	0	1
294	Human	Southern	0	0	0	1	0	1
295	Human	Southern	0	0	0	1	0	1
296	Human	Southern	0	0	0	1	0	1
297	Human	Northeastern	0	0	0	1	0	1
298	Human	Northeastern	0	0	0	1	0	1
299	Human	Northeastern	0	0	0	0	0	0
300	Human	Northeastern	0	0	0	1	0	1
301	Human	Northeastern	0	0	0	0	0	0
302	Human	Northeastern	0	0	0	0	0	1
303	Human	Northeastern	0	0	0	0	0	0
304	Human	Northeastern	0	1	0	0	0	1
305	Human	Northeastern	0	0	0	1	0	1
306	Human	Northeastern	0	0	0	0	0	0
307	Human	Northeastern	0	0	0	1	0	1
308	Human	Northeastern	0	0	0	1	0	1
309	Human	Northeastern	0	0	0	1	0	1
310	Human	Northeastern	0	0	0	1	0	1
311	Human	Northeastern	0	0	0	1	0	1
312	Human	Northeastern	0	0	0	1	0	1
313	Human	Northeastern	0	0	0	1	0	1
314	Human	Northeastern	0	0	0	1	0	1
315	Human	Northeastern	1	0	0	0	0	1
316	Human	Northeastern	0	0	0	1	0	1
317	Human	Northeastern	1	0	0	0	0	1
318	Human	Northeastern	0	0	0	0	0	0
319	Human	Northeastern	0	0	0	1	0	1
320	Human	Northeastern	0	0	0	1	0	1
321	Human	Northeastern	0	0	0	0	0	0
322	Human	Northeastern	0	0	0	1	0	1
323	Human	Northeastern	0	0	0	1	0	1
324	Human	Northeastern	0	0	0	1	0	1
325	Human	Northeastern	0	0	0	0	0	0
326	Human	Northeastern	1	0	0	1	0	1
327	Human	Northeastern	0	0	0	1	0	1
328	Human	Northeastern	0	0	0	1	0	1
329	Human	Northeastern	0	0	0	0	0	0
330	Human	Northeastern	0	0	0	1	0	1
331	Human	Northeastern	0	0	0	1	0	1
332	Human	Northeastern	0	0	0	1	0	1
333	Human	Northeastern	0	0	0	1	0	1
334	Human	Northeastern	1	0	0	0	0	1
335	Human	Northeastern	0	0	0	0	0	0

336	Human	Northeastern	0	0	0	1	0	1
337	Human	Northeastern	0	0	0	0	0	0
338	Human	Northeastern	0	0	0	0	0	0
339	Human	Northeastern	0	0	0	1	0	1
340	Human	Northeastern	0	0	0	1	0	1
341	Human	Northeastern	0	0	0	0	0	0
342	Human	Northeastern	1	0	0	0	0	1
343	Human	Northeastern	0	0	0	0	0	0
344	Human	Northeastern	0	0	0	1	0	1
345	Human	Northeastern	0	0	0	0	0	0
346	Human	Northeastern	0	0	0	0	0	1
347	Human	Northeastern	0	0	0	0	0	0
348	Human	Northeastern	0	1	0	0	0	1
349	Human	Northeastern	0	0	0	0	0	0
350	Human	Northeastern	0	0	0	1	0	1
351	Human	Central	0	0	0	0	0	0
352	Human	Central	0	0	0	0	0	0
353	Human	Central	0	0	0	0	0	0
354	Human	Central	0	0	0	0	0	0
355	Human	Central	1	0	0	0	0	1
356	Human	Central	0	0	0	0	0	0
357	Human	Central	1	0	0	0	0	1
358	Human	Central	0	0	0	0	0	0
359	Human	Central	0	0	0	0	0	0
360	Human	Northern	0	0	0	1	0	1
361	Human	Northern	0	0	0	1	0	1
362	Human	Northern	0	0	0	0	0	0
363	Human	Northern	0	0	0	0	0	0
364	Human	Northern	0	0	0	0	0	0
365	Human	Northern	0	0	0	0	0	0
366	Human	Northern	0	0	0	0	0	0
367	Human	Northern	0	0	0	0	0	0
368	Human	Northern	0	0	0	0	0	0
369	Human	Northern	0	0	0	0	0	0
370	Human	Northern	0	0	0	0	0	0
371	Human	Northern	0	0	0	1	0	1
372	Human	Northern	0	0	0	0	0	0
373	Human	Northern	0	0	0	0	0	0
374	Human	Northern	0	0	0	0	0	0
375	Human	Northern	0	0	0	0	0	0
376	Human	Northern	0	0	0	0	0	0
377	Human	Northern	0	0	0	0	0	0
378	Human	Northern	0	0	0	0	0	0
379	Human	Northern	0	0	0	0	0	0
380	Human	Northern	0	0	0	0	0	0
381	Human	Northern	0	0	0	0	0	0
382	Human	Northern	0	0	0	0	0	0
383	Human	Northern	0	0	0	0	0	0

384	Human	Northern	0	0	0	0	0	0
385	Human	Northern	0	0	0	0	0	0
386	Human	Northern	0	0	0	0	0	0
387	Human	Northern	0	0	0	0	0	0
388	Human	Northern	0	0	0	0	0	0
389	Human	Northern	0	0	0	0	0	0
390	Human	Northern	0	0	0	0	0	0
391	Human	Northern	0	0	0	0	0	0
392	Human	Northern	0	0	0	0	0	0
393	Human	Northern	0	0	0	0	0	0
394	Human	Northern	0	0	0	0	0	0
395	Human	Northern	0	0	0	0	0	0
396	Human	Northern	0	0	0	0	0	0
397	Human	Northern	1	1	0	1	0	1
398	Human	Northern	0	0	0	0	0	0
399	Human	Northern	0	0	0	0	0	0
400	Human	Northern	0	0	0	0	0	0
401	Human	Northern	0	0	0	0	0	0
402	Human	Northern	0	0	0	0	0	0
403	Human	Northern	0	0	0	0	0	0
404	Human	Northern	0	0	0	0	0	0
405	Human	Northern	0	0	0	0	0	0
406	Human	Northern	0	0	0	0	0	0
407	Human	Northern	0	0	0	0	0	0
408	Human	Northern	0	0	0	0	0	0
409	Human	Northern	0	0	0	1	0	1
410	Human	Northern	0	0	0	0	0	0
411	Human	Northern	0	0	0	0	0	0
412	Human	Northern	0	0	0	0	0	0
413	Human	Northern	0	0	0	0	0	0
414	Human	Northern	0	0	0	0	0	0
415	Human	Northern	0	0	0	0	0	0
416	Human	Northern	0	0	0	0	0	0
417	Human	Northern	0	0	0	0	0	0
418	Human	Northern	0	0	0	0	0	0
419	Human	Northern	0	0	0	0	0	0
420	Human	Northern	0	0	0	0	0	0
421	Human	Northern	0	0	0	0	0	0
422	Human	Northern	0	0	0	0	0	0
423	Human	Northern	0	0	0	0	0	0
424	Human	Northern	0	0	0	1	0	1
425	Human	Northern	0	0	0	1	0	1
426	Human	Northern	0	0	0	0	0	0
427	Human	Northern	0	0	0	0	0	0
428	Human	Northern	0	0	0	0	0	0
429	Human	Northern	0	0	0	0	0	0
430	Human	Northern	0	0	0	0	0	0
431	Human	Northern	0	0	0	0	0	0

432	Human	Northern	0	0	0	0	0	0
433	Human	Northern	0	0	0	0	0	0
434	Human	Northern	0	0	0	0	0	0
435	Human	Northern	0	0	0	0	0	0
436	Human	Northern	0	0	0	0	0	0
437	Human	Northern	1	0	0	0	0	1
438	Human	Northern	0	0	0	0	0	0
439	Human	Northern	0	0	0	0	0	0
440	Human	Northern	0	0	0	0	0	0
441	Human	Northern	0	0	0	0	0	0
442	Human	Northern	0	0	0	0	0	0
443	Human	Northern	0	0	0	1	0	1
444	Human	Northern	0	0	0	0	0	0
445	Human	Northern	0	0	0	1	0	1
446	Human	Northern	0	0	0	0	0	0
447	Human	Northern	0	0	0	0	0	0
448	Human	Northern	0	0	0	0	0	0
449	Human	Northern	0	0	0	0	0	0
450	Human	Northern	0	0	0	0	0	0
451	Human	Northern	0	0	0	0	0	0
452	Human	Northern	0	0	0	0	0	0
453	Human	Northern	0	0	0	0	0	0
454	Human	Northern	0	0	0	0	0	0
455	Human	Northern	0	0	0	0	0	0
456	Human	Northern	0	0	0	0	0	0
457	Human	Northern	0	0	0	0	0	0
458	Human	Northern	0	0	0	0	0	0
459	Human	Northern	0	0	0	0	0	0
460	Human	Northern	0	0	0	0	0	0
461	Human	Northern	0	0	0	0	0	0
462	Human	Northern	0	0	0	0	0	0
463	Human	Northern	0	0	0	0	0	1
464	Human	Northern	0	0	0	0	0	1
465	Human	Northern	0	0	0	0	0	0
466	Human	Northern	0	0	0	0	0	0
467	Human	Northern	0	0	0	0	0	0
468	Human	Northern	0	0	0	0	0	0
469	Human	Northern	0	0	0	0	0	0
470	Human	Northern	0	0	0	0	0	0
471	Human	Northern	0	0	0	0	0	0
472	Human	Northern	0	0	0	0	0	0
473	Human	Northern	0	0	0	0	0	0
474	Human	Northern	0	0	0	0	0	0
475	Human	Northern	0	0	0	0	0	0
476	Human	Northern	0	0	0	0	0	0
477	Human	Northern	0	0	0	0	0	0
478	Human	Northern	0	0	0	0	0	0
479	Human	Northern	0	0	0	0	0	0

480	Human	Northern	0	0	0	0	0	0
481	Human	Northern	0	0	0	0	0	0
482	Human	Northern	0	0	0	0	0	0
483	Human	Northern	0	0	0	0	0	0
484	Human	Northern	0	0	0	0	0	0
485	Human	Northern	0	0	0	0	0	0
486	Human	Northern	0	0	0	0	0	0
487	Human	Northern	0	0	0	0	0	0
488	Human	Northern	0	0	0	0	0	0
489	Human	Northern	0	0	0	0	0	0
490	Human	Northern	0	0	0	0	0	0
491	Human	Northern	0	0	0	0	0	0
492	Human	Northern	0	0	0	0	0	0
493	Human	Northern	0	0	0	1	0	1
494	Human	Northern	0	0	0	0	0	0
495	Human	Northern	0	0	0	0	0	0
496	Human	Northern	0	0	0	0	0	0
497	Human	Northern	0	0	0	0	0	0
498	Human	Northern	0	0	0	0	0	0
499	Human	Northern	0	0	0	0	0	0
500	Human	Northern	0	0	0	0	0	0
501	Human	Northern	0	0	0	0	0	0
502	Human	Northern	0	0	0	0	0	0
503	Human	Northern	0	0	0	0	0	0
504	Human	Northern	0	0	0	0	0	0
505	Human	Central	0	0	0	0	0	0
506	Human	Central	0	0	0	0	0	0
507	Human	Northern	0	0	0	1	0	1
508	Human	Central	0	0	0	0	0	0
509	Human	Central	0	0	0	0	0	0
510	Human	Central	0	0	0	0	0	0
511	Human	Central	0	0	0	0	0	0
512	Human	Central	0	0	0	0	0	0
513	Human	Eastern	0	0	0	0	0	0
514	Human	Central	0	0	0	0	0	0
515	Human	Central	0	0	0	0	0	0
516	Human	Northern	0	0	0	0	0	0
517	Human	Eastern	0	0	0	0	0	0
518	Human	Central	0	0	0	0	0	0
519	Human	Central	0	0	0	0	0	1
520	Human	Central	0	0	0	0	0	0
521	Human	Central	0	0	0	0	0	1
522	Human	Northeastern	1	1	0	1	0	1
523	Human	Northeastern	1	0	0	0	0	1
524	Human	Northeastern	0	0	0	0	0	0
525	Human	Northeastern	0	0	0	0	0	0
526	Human	Northeastern	0	0	0	0	0	0
527	Human	Northeastern	0	0	0	0	0	0

528	Human	Northeastern	0	0	0	0	0	0
529	Human	Northeastern	1	0	0	0	0	1
530	Human	Eastern	0	0	0	0	0	0
531	Human	Central	0	0	0	0	0	0
532	Human	Central	0	0	0	0	0	0
533	Human	Northern	0	0	0	0	0	0
534	Human	Northern	0	0	0	0	0	0
535	Human	Eastern	0	0	0	0	0	0
536	Human	Central	0	0	0	0	0	0
537	Human	Central	0	0	0	0	0	0
538	Human	Northern	0	0	0	0	0	0
539	Human	Northern	0	0	0	0	0	1
540	Human	Eastern	0	0	0	0	0	0
541	Human	Central	0	0	0	0	0	0
542	Human	Eastern	0	0	0	0	0	0
543	Human	Northern	1	0	0	0	0	1
544	Human	Northern	0	0	0	1	0	1
545	Human	Eastern	0	0	0	0	0	0
546	Human	Northeastern	0	0	0	1	0	1
547	Human	Northeastern	0	0	0	0	0	0
548	Human	Northeastern	0	0	0	0	0	1
549	Human	Northeastern	0	0	0	0	0	1
550	Human	Northeastern	0	0	0	1	0	1
551	Human	Northeastern	1	0	0	1	0	1
552	Human	Central	0	0	0	0	0	0
553	Human	Northern	0	0	0	0	0	0
554	Human	Northeastern	0	0	0	0	0	0
555	Human	Central	0	0	0	0	0	0
556	Human	Northeastern	0	0	0	0	0	0
557	Human	Northeastern	1	0	0	0	0	1
558	Human	Northeastern	0	0	0	0	0	0
559	Human	Northeastern	0	0	0	0	0	0
560	Human	Northeastern	0	0	0	0	0	0
561	Human	Northeastern	0	0	0	0	0	0
562	Human	Northeastern	0	0	0	0	0	0
563	Human	Northeastern	0	0	0	1	0	1
564	Human	Northeastern	0	0	0	0	0	0
565	Human	Northeastern	0	0	0	1	0	1
566	Human	Northeastern	0	0	0	0	0	0
567	Human	Northeastern	0	0	0	0	0	0
568	Human	Northeastern	0	0	0	0	0	0
569	Human	Northeastern	0	0	0	0	0	0
570	Human	Northeastern	1	0	0	0	0	1
571	Human	Northeastern	0	0	0	0	0	1
572	Human	Northeastern	1	0	0	0	0	1
573	Human	Northern	0	0	0	0	0	0
574	Human	Central	0	0	0	0	0	0
575	Human	Central	0	0	0	0	0	0

576	Human	Northern	0	0	0	0	0	0
577	Human	Central	0	0	0	0	0	0
578	Human	Northeastern	0	0	0	0	0	0
579	Human	Northern	0	0	0	0	0	0
580	Human	Central	0	0	0	0	0	0
581	Human	Central	0	0	0	0	0	0
582	Human	Eastern	0	0	0	1	0	1
583	Human	Southern	0	0	0	0	0	0
584	Human	Central	0	0	0	0	0	0
585	Human	Northern	0	0	0	1	0	1
586	Human	Northern	1	0	0	0	0	1
587	Human	Northern	1	0	0	0	0	1
588	Human	Northern	0	0	0	0	0	0
589	Human	Northern	0	0	0	0	0	0
590	Human	Northern	0	0	0	0	0	0
591	Human	Northern	0	0	0	0	0	0
592	Human	Northern	0	0	0	1	0	1
593	Human	Northern	0	0	0	1	0	1
594	Human	Northern	0	0	0	0	0	0
595	Human	Northern	0	0	0	0	0	0
596	Human	Northern	0	1	0	0	0	1
597	Human	Northern	0	0	0	0	0	0
598	Human	Central	1	0	0	1	0	1
599	Human	Central	0	0	0	0	0	0
600	Human	Central	0	0	0	0	0	0
601	Human	Northern	0	0	0	0	0	0
602	Human	Northern	0	0	0	0	0	0
603	Human	Northern	0	0	0	0	0	0
604	Human	Northern	0	0	0	1	0	1
605	Human	Northern	0	0	0	1	0	1
606	Human	Northern	0	0	0	0	0	0
607	Human	Northern	0	0	0	0	0	0
608	Human	Northern	1	0	0	0	0	1
609	Human	Northern	0	0	0	0	0	0
610	Human	Northern	0	0	0	0	0	0
611	Human	Northern	0	0	0	0	0	0
612	Human	Northern	0	0	0	0	0	0
613	Human	Northern	0	0	0	0	0	0
614	Human	Northern	0	0	0	0	0	0
615	Human	Northern	0	0	0	0	0	0
616	Human	Northern	0	0	0	0	0	0
617	Human	Northern	0	0	0	0	0	0
618	Human	Northern	0	0	0	0	0	0
619	Human	Northern	0	0	0	0	0	0
620	Human	Northern	0	0	0	0	0	0
621	Human	Northern	0	0	0	0	0	0
622	Human	Southern	0	0	0	1	0	1
623	Human	Northern	0	0	0	0	0	0

624	Human	Northern	1	0	0	0	0	1
625	Human	Northern	0	0	0	0	0	0
626	Human	Northern	0	0	0	1	0	1
627	Human	Southern	0	0	0	0	0	0
628	Human	Central	0	0	0	0	0	0
629	Human	Central	0	0	0	0	0	0
630	Human	Central	0	0	0	0	0	0
631	Human	Southern	0	0	0	0	0	0
632	Human	Northeastern	0	0	0	1	0	1
633	Human	Northeastern	0	0	0	0	0	0
634	Human	Northeastern	0	1	0	1	0	1
635	Human	Northeastern	0	0	0	0	0	0
636	Human	Northeastern	0	0	0	0	0	1
637	Human	Northeastern	0	0	0	0	0	0
638	Human	Northeastern	0	0	0	0	0	0
639	Human	Northeastern	0	0	0	0	0	0
640	Human	Eastern	0	0	0	0	0	0
641	Human	Central	0	0	0	0	0	0
642	Human	Central	0	0	0	0	0	0
643	Human	Southern	0	0	0	0	0	0
644	Human	Northern	0	0	0	0	0	0
645	Human	Northern	0	0	0	1	0	1
646	Human	Northern	1	0	0	0	0	1
647	Human	Eastern	0	0	0	0	0	0
648	Human	Southern	0	0	0	0	1	1
649	Human	Southern	0	0	0	0	1	1
650	Human	Southern	0	0	0	0	1	1
651	Human	Southern	0	0	0	0	1	1
652	Human	Southern	0	0	0	0	0	1
653	Human	Southern	0	0	0	0	1	1
654	Human	Southern	0	0	0	0	1	1
655	Human	Southern	0	0	0	0	1	1
656	Human	Southern	0	0	0	1	1	1
657	Human	Southern	0	0	0	0	1	1
658	Human	Southern	0	0	0	0	0	1
659	Human	Southern	0	0	0	0	0	1
660	Human	Southern	0	0	0	0	0	0
661	Human	Southern	0	0	0	0	1	1
662	Human	Southern	0	0	0	0	1	1
663	Human	Southern	0	0	0	0	1	1
664	Human	Southern	0	0	0	0	1	1
665	Human	Southern	0	0	0	0	0	0
666	Human	Eastern	0	0	0	0	0	0
667	Human	Northeastern	0	0	0	0	0	0
668	Human	Southern	0	0	0	0	0	0
669	Human	Southern	0	0	0	0	0	0
670	Human	Eastern	0	0	0	0	0	0
671	Human	Eastern	0	0	0	0	0	0

672	Human	Eastern	0	0	0	0	0	0
673	Human	Central	0	0	0	0	0	0
674	Human	Northeastern	0	0	0	1	0	1
675	Human	Northeastern	0	0	0	0	0	0
676	Human	Eastern	0	0	0	0	0	0
677	Human	Eastern	0	0	0	0	0	0
678	Human	Eastern	0	0	0	0	0	0
679	Human	Northeastern	0	0	0	0	0	0
680	Human	Northeastern	1	0	0	0	0	1
681	Human	Northeastern	0	0	0	0	0	0
682	Human	Northeastern	0	0	0	0	0	0
683	Human	Southern	0	0	0	0	0	0
684	Human	Northeastern	0	0	0	0	0	0
685	Human	Eastern	0	0	0	0	0	0
686	Human	Eastern	0	0	0	0	0	0
687	Human	Eastern	0	0	0	0	0	0
688	Human	Southern	0	0	0	0	0	0
689	Human	Northeastern	1	0	0	0	0	1
690	Human	Northeastern	0	0	0	1	0	1
691	Human	Southern	0	0	0	0	0	0
692	Human	Southern	0	0	0	1	0	1
693	Human	Southern	0	0	0	1	0	1
694	Human	Southern	0	0	0	1	0	1
695	Human	Southern	0	0	0	0	0	0
696	Human	Central	0	0	0	0	0	0
697	Human	Northeastern	0	0	0	1	0	1
698	Human	Eastern	0	0	0	1	0	1
699	Human	Southern	0	0	0	1	0	1
700	Human	Central	0	0	0	0	0	0
701	Human	Central	0	0	0	0	0	0
702	Human	Central	0	0	0	0	0	0
703	Human	Southern	0	0	0	0	0	0
704	Human	Southern	0	0	0	0	0	0
705	Human	Southern	0	0	0	0	0	0
706	Human	Southern	0	0	0	0	0	0
707	Human	Southern	1	0	0	0	0	1
708	Human	Southern	0	0	0	0	0	0
709	Human	Central	0	0	0	0	0	0
710	Human	Southern	0	0	0	0	0	0
711	Human	Southern	0	0	0	0	0	0
712	Human	Southern	0	0	0	1	0	1
713	Human	Southern	0	0	0	1	0	1
714	Human	Southern	0	0	0	0	0	0
715	Human	Central	0	0	0	0	0	0
716	Human	Northeastern	0	0	0	0	0	0
717	Human	Northeastern	0	0	0	0	0	0
718	Human	Northeastern	0	0	0	0	0	0
719	Human	Northeastern	0	0	0	0	0	0

720	Human	Eastern	0	0	0	0	0	0
721	Human	Eastern	0	0	0	0	0	0
722	Human	Central	0	0	0	1	0	1
723	Human	Northeastern	0	0	0	0	0	0
724	Human	Eastern	0	0	0	0	0	0
725	Human	Eastern	0	0	0	0	0	0
726	Human	Central	0	0	0	1	0	1
727	Human	Central	0	0	0	1	0	1
728	Human	Central	0	0	0	1	0	1
729	Human	Central	0	0	0	1	0	1
730	Human	Central	0	0	0	0	0	0
731	Human	Central	0	0	0	0	0	0
732	Human	Southern	0	0	0	0	0	0
733	Human	Southern	0	0	0	0	0	0
734	Human	Southern	0	0	0	0	0	0
735	Human	Southern	0	0	0	0	0	0
736	Human	Southern	0	0	0	1	0	1
737	Human	Southern	0	0	0	0	0	0
738	Human	Southern	0	0	0	0	0	0
739	Human	Southern	0	0	0	0	0	0
740	Human	Eastern	0	0	0	0	0	0
741	Human	Eastern	0	0	0	0	0	0
742	Human	Northeastern	0	0	0	1	0	1
743	Human	Northeastern	0	0	0	1	0	1
744	Human	Northeastern	0	0	0	0	0	0
745	Human	Northeastern	0	0	0	0	0	0
746	Human	Northeastern	0	0	0	1	0	1
747	Human	Northeastern	1	0	0	0	0	1
748	Human	Northern	0	0	0	0	0	0
749	Human	Northern	0	0	0	0	0	0
750	Human	Northern	0	0	0	0	0	0
751	Human	Northern	0	0	0	0	0	0
752	Human	Northern	0	0	0	0	0	0
753	Human	Northern	0	0	0	0	0	0
754	Human	Northern	0	0	0	0	0	0
755	Human	Northern	0	0	0	0	0	0
756	Human	Northern	0	0	0	0	0	0
757	Human	Northern	0	0	0	0	0	0
758	Human	Northern	0	0	0	0	0	0
759	Human	Northern	0	0	0	0	0	0
760	Human	Northern	0	0	0	0	0	0
761	Human	Northern	0	0	0	0	0	0
762	Human	Northern	0	0	0	0	0	0
763	Human	Northern	0	0	0	0	0	0
764	Human	Northern	0	0	0	0	0	0
765	Human	Northern	0	0	0	0	0	0
766	Human	Northern	0	0	0	0	0	0
767	Human	Northern	0	0	0	0	0	0

768	Human	Northern	0	0	0	0	0	0
769	Human	Northern	0	0	0	0	0	0
770	Human	Northern	0	0	0	0	0	0
771	Human	Central	0	0	0	0	0	0
772	Human	Eastern	0	0	0	0	0	0
773	Human	Central	0	0	0	0	0	0
774	Human	Southern	0	0	0	0	0	0
775	Human	Southern	0	0	0	0	0	0
776	Human	Central	0	0	0	0	0	0
777	Human	Eastern	0	0	0	0	0	0
778	Human	Central	0	0	0	0	0	0
779	Human	Central	0	0	0	0	0	0
780	Human	Northeastern	0	0	0	0	0	0
781	Human	Northeastern	0	0	0	0	0	0
782	Human	Northeastern	0	0	0	0	0	0
783	Human	Northeastern	0	0	0	1	0	1
784	Human	Northeastern	0	0	0	0	0	0
785	Human	Central	0	0	0	0	0	0
786	Human	Southern	0	0	0	0	0	0
787	Human	Northeastern	0	0	0	0	0	0
788	Human	Northeastern	0	0	0	0	0	0
789	Human	Northeastern	0	0	0	0	0	0
790	Human	Central	0	0	0	0	0	0
791	Human	Central	0	0	0	1	0	1
792	Human	Central	0	0	0	1	0	1
793	Human	Central	0	0	0	0	0	0
794	Human	Northeastern	0	0	0	0	0	0
795	Human	Northeastern	0	0	0	0	0	0
796	Human	Northeastern	0	0	0	0	0	0
797	Human	Central	0	0	0	0	0	0
798	Human	Central	0	0	0	0	0	0
799	Human	Central	0	0	0	0	0	0
800	Human	Southern	0	0	0	0	0	0
801	Human	Southern	0	0	0	0	0	0
802	Human	Eastern	0	0	0	0	0	0
803	Human	Central	0	0	0	0	0	0
804	Human	Central	0	0	0	0	0	0
805	Human	Southern	0	0	0	0	0	0
806	Human	Central	0	0	0	0	0	0
807	Human	Central	0	0	0	0	0	0
808	Human	Central	1	0	0	0	0	1
809	Human	Central	0	0	0	0	0	0
810	Human	Central	0	0	0	0	0	0
811	Human	Central	0	0	0	0	0	1
812	Human	Central	1	0	0	0	0	1
813	Human	Central	0	0	0	0	0	0
814	Human	Central	1	0	0	0	0	1
815	Human	Central	0	0	0	0	0	0

816	Human	Central	0	0	0	0	0	0
817	Human	Central	1	0	0	1	0	1
818	Human	Central	0	0	0	0	0	1
819	Human	Central	0	0	0	0	0	0
820	Human	Central	0	0	0	0	0	0
821	Human	Central	0	0	0	0	0	0
822	Human	Central	0	0	0	0	0	1
823	Human	Central	0	0	0	0	0	0
824	Human	Central	0	0	0	0	0	1
825	Human	Central	0	0	0	0	0	1
826	Human	Central	0	0	0	1	0	1
827	Human	Central	0	0	0	1	0	1
828	Human	Central	0	0	0	1	0	1
829	Human	Central	0	0	0	0	0	0
830	Human	Central	1	0	0	1	0	1
831	Human	Central	0	0	0	0	0	0
832	Human	Central	0	0	0	0	0	0
833	Human	Central	0	0	0	0	0	0
834	Human	Central	0	0	0	0	0	1
835	Human	Central	0	0	0	0	0	0
836	Human	Central	0	0	0	0	0	0
837	Human	Central	0	0	0	0	0	0
838	Human	Central	1	0	0	0	0	1
839	Human	Central	0	0	0	0	0	0
840	Human	Central	0	0	0	0	0	1
841	Human	Central	0	0	0	1	0	1
842	Human	Central	0	0	0	1	0	1
843	Human	Central	0	0	0	0	0	0
844	Human	Central	0	0	0	0	0	1
845	Human	Central	0	0	0	0	0	1
846	Human	Central	0	0	0	0	0	1
847	Human	Northeastern	0	0	0	1	0	1
848	Human	Northeastern	1	0	0	0	0	1
849	Human	Northeastern	0	0	0	0	0	0
850	Human	Northeastern	0	0	0	1	0	1
851	Human	Northeastern	0	0	0	0	0	0
852	Human	Northeastern	0	0	0	0	0	0
853	Human	Northeastern	0	0	0	0	0	0
854	Human	Northeastern	0	0	0	0	0	0
855	Human	Northeastern	0	0	0	0	0	0
856	Human	Northeastern	0	0	0	0	0	0
857	Human	Southern	0	0	0	0	0	0
858	Human	Central	0	0	0	0	0	0
859	Human	Central	0	0	0	0	0	0
860	Human	Central	0	0	0	0	0	0
861	Human	Central	0	0	0	0	0	0
862	Human	Central	0	0	0	0	0	0
863	Human	Central	0	0	0	0	0	0

864	Human	Central	0	0	0	0	0	0
865	Human	Central	0	0	0	0	0	0
866	Human	Central	0	0	0	1	0	1
867	Human	Central	0	0	0	0	0	1
868	Human	Central	0	0	0	0	0	1
869	Human	Central	0	0	0	0	0	0
870	Human	Central	0	0	0	0	0	0
871	Human	Central	0	0	0	0	0	0
872	Human	Central	0	0	0	0	0	0
873	Human	Central	0	0	0	0	0	0
874	Human	Central	0	0	0	1	0	1
875	Human	Central	0	0	0	0	0	0
876	Human	Central	0	0	0	0	0	0
877	Human	Central	0	0	0	0	0	0
878	Human	Central	0	0	0	0	0	0
879	Human	Central	0	0	0	0	0	0
880	Human	Central	0	0	0	0	0	0
881	Human	Central	0	0	0	0	0	0
882	Human	Central	0	0	0	0	0	0
883	Human	Central	0	0	0	0	0	0
884	Human	Central	0	0	0	0	0	0
885	Human	Central	0	0	0	0	0	0
886	Human	Central	0	0	0	0	0	0
887	Human	Central	0	0	0	0	0	0
888	Human	Central	0	0	0	0	0	0
889	Human	Central	0	0	0	1	0	1
890	Human	Central	0	0	0	1	0	1
891	Human	Central	0	0	0	1	0	1
892	Human	Central	0	0	0	1	0	1
893	Human	Central	0	0	0	0	0	0
894	Human	Central	0	0	0	0	0	0
895	Human	Central	0	0	0	0	0	0
896	Human	Central	0	0	0	0	0	0
897	Human	Central	0	0	0	0	0	0
898	Human	Central	0	0	0	1	0	1
899	Human	Central	0	0	0	0	0	0
900	Human	Central	0	0	0	0	0	0
901	Human	Central	0	0	0	0	0	0
902	Human	Central	0	0	0	0	0	0
903	Human	Central	0	0	0	0	0	0
904	Human	Central	0	0	0	0	0	0
905	Human	Central	0	0	0	0	0	0
906	Human	Central	0	0	0	0	0	0
907	Human	Central	0	0	0	0	0	0
908	Human	Central	0	0	0	0	0	0
909	Human	Central	0	0	0	0	0	0
910	Human	Central	0	0	0	0	0	0
911	Human	Central	0	0	0	0	0	0

912	Human	Central	0	0	0	1	0	1
913	Human	Central	0	0	0	0	0	0
914	Human	Central	0	0	0	0	0	0
915	Human	Central	0	0	0	0	0	0
916	Human	Central	0	0	0	0	0	0
917	Human	Central	0	0	0	1	0	1
918	Human	Central	0	0	0	1	0	1
919	Human	Central	0	0	0	0	0	0
920	Human	Central	0	0	0	1	0	1
921	Human	Central	0	0	0	0	0	0
922	Human	Central	0	0	0	0	0	1
923	Human	Central	0	0	0	0	0	0
924	Human	Central	0	0	0	0	0	1
925	Human	Central	0	0	0	0	0	0
926	Human	Central	0	0	0	1	0	1
927	Human	Central	0	0	0	1	0	1
928	Human	Central	0	0	0	0	0	1
929	Human	Central	0	0	0	1	0	1
930	Human	Central	0	0	0	1	0	1
931	Human	Central	0	0	0	0	0	0
932	Human	Central	0	0	0	0	0	0
933	Human	Central	0	0	0	0	0	1
934	Human	Central	0	0	0	0	0	1
935	Human	Central	0	0	0	1	0	1
936	Human	Central	0	0	0	1	0	1
937	Human	Central	0	0	0	0	0	0
938	Human	Central	0	0	0	0	0	1
939	Human	Central	0	0	0	0	0	0
940	Human	Central	0	0	0	1	0	1
941	Human	Central	0	1	0	0	0	1
942	Human	Central	0	1	0	1	0	1
943	Human	Central	0	0	0	0	0	0
944	Human	Central	0	0	0	0	0	0
945	Human	Central	0	0	0	0	0	0
946	Human	Central	0	0	0	0	0	0
947	Human	Central	0	0	0	0	0	0
948	Human	Central	0	0	0	0	0	0
949	Human	Central	0	0	0	0	0	0
950	Human	Central	0	0	0	0	0	0
951	Human	Central	0	0	0	0	0	0
952	Human	Central	0	0	0	0	0	0
953	Human	Central	0	0	0	0	0	0
954	Human	Central	0	0	0	0	0	0
955	Human	Central	0	0	0	0	0	0
956	Human	Central	0	0	0	0	0	0
957	Human	Central	0	0	0	0	0	0
958	Human	Central	0	0	0	1	0	1
959	Human	Central	0	0	0	0	0	0

960	Human	Central	0	0	0	0	0	0
961	Human	Central	0	0	0	0	0	0
962	Human	Central	0	0	0	1	0	1
963	Human	Central	1	0	0	1	0	1
964	Human	Central	1	0	0	1	0	1
965	Human	Central	0	0	0	0	0	0
966	Human	Central	0	0	0	0	0	0
967	Human	Central	0	0	0	1	0	1
968	Human	Central	0	0	0	1	0	1
969	Human	Central	0	0	0	0	0	0
970	Human	Central	0	0	0	0	0	0
971	Human	Central	0	0	0	0	0	0
972	Human	Central	0	0	0	0	0	0
973	Human	Central	0	0	0	0	0	0
974	Human	Central	0	0	0	1	0	1
975	Human	Central	0	0	0	0	0	0
976	Human	Central	0	0	0	0	0	0
977	Human	Central	0	0	0	0	0	0
978	Human	Central	0	0	0	0	0	0
979	Human	Central	0	0	0	0	0	0
980	Human	Central	0	0	0	0	0	0
981	Human	Central	0	0	0	0	0	0
982	Human	Central	1	0	0	0	0	1
983	Human	Central	0	0	0	0	0	0
984	Human	Central	0	0	0	0	0	0
985	Human	Central	0	0	0	0	0	0
986	Human	Central	0	0	0	0	0	1
987	Human	Central	0	0	0	0	0	0
988	Human	Central	0	0	0	0	0	0
989	Human	Central	0	0	0	1	0	1
990	Human	Central	0	0	0	1	0	1
991	Human	Central	0	0	0	0	0	0
992	Human	Central	0	0	0	0	0	0
993	Human	Central	0	0	0	0	0	0
994	Human	Central	1	0	0	1	0	1
995	Human	Central	1	0	0	1	0	1
996	Human	Central	0	0	0	0	0	1
997	Human	Central	0	0	0	0	0	0
998	Human	Central	0	0	0	0	0	0
999	Human	Central	0	0	0	0	0	0
1000	Human	Central	0	0	0	0	0	0
1001	Human	Central	0	0	0	0	0	0
1002	Human	Central	0	0	0	0	0	0
1003	Human	Central	0	0	0	1	0	1
1004	Human	Central	0	0	0	1	0	1
1005	Human	Central	0	0	0	0	0	0
1006	Human	Central	0	0	0	1	0	1
1007	Human	Northeastern	0	0	0	0	0	0

1008	Human	Central	0	0	0	0	0	0
1009	Human	Central	0	0	0	0	0	0
1010	Human	Central	0	0	0	0	0	0
1011	Human	Northeastern	0	0	0	0	0	0
1012	Human	Northeastern	0	0	0	0	0	0
1013	Human	Northeastern	0	0	0	1	0	1
1014	Human	Central	0	0	0	0	0	0
1015	Human	Northern	1	0	0	0	0	1
1016	Human	Northern	0	0	0	0	0	0
1017	Human	Northern	0	0	0	0	0	0
1018	Human	Northern	0	0	0	0	0	0
1019	Human	Northern	0	0	0	0	0	0
1020	Human	Northern	0	0	0	0	0	0
1021	Human	Northern	0	0	0	0	0	0
1022	Human	Northern	0	0	0	0	0	0
1023	Human	Northern	0	0	0	0	0	0
1024	Human	Northern	0	0	0	0	0	0
1025	Human	Northern	0	0	0	0	0	0
1026	Human	Central	0	0	0	0	0	0
1027	Human	Central	0	0	0	0	0	0
1028	Human	Southern	0	0	0	0	0	0
1029	Human	Southern	0	0	0	0	0	0
1030	Human	Southern	0	0	0	0	0	0
1031	Human	Northeastern	0	0	0	0	0	0
1032	Human	Southern	0	0	0	0	0	0
1033	Human	Southern	0	0	0	0	0	0
1034	Human	Central	0	0	0	0	0	0
1035	Human	Southern	0	0	0	0	0	0
1036	Human	Southern	0	0	0	0	0	0
1037	Human	Southern	0	0	0	0	0	0
1038	Human	Southern	0	0	0	0	0	0
1039	Human	Southern	0	0	0	0	0	0
1040	Human	Southern	0	0	0	0	0	0
1041	Human	Southern	0	0	0	0	0	0
1042	Human	Southern	0	0	0	0	0	0
1043	Human	Southern	0	0	0	0	0	0
1044	Human	Southern	0	0	0	0	0	0
1045	Human	Southern	0	0	0	0	0	0
1046	Human	Southern	0	0	0	0	0	0
1047	Human	Southern	0	0	0	0	0	0
1048	Human	Southern	0	0	0	0	0	0
1049	Human	Southern	0	0	0	0	0	0
1050	Human	Southern	0	0	0	0	0	0
1051	Human	Southern	0	0	0	1	0	1
1052	Human	Southern	0	0	0	1	0	1
1053	Human	Southern	0	0	0	0	0	0
1054	Human	Southern	0	0	0	0	0	0
1055	Human	Southern	0	0	0	0	0	0

1056	Human	Southern	0	0	0	0	0	0
1057	Human	Southern	0	0	0	1	0	1
1058	Human	Southern	0	0	0	0	0	0
1059	Human	Central	0	0	0	0	0	0
1060	Human	Central	0	0	0	0	0	0
1061	Human	Southern	0	0	0	0	0	0
1062	Human	Northeastern	0	0	0	0	0	0
1063	Human	Northeastern	0	0	0	0	0	0
1064	Human	Northeastern	0	0	0	0	0	0
1065	Human	Northeastern	0	0	0	1	0	1
1066	Human	Northeastern	0	0	0	0	0	0
1067	Human	Northeastern	0	0	0	0	0	0
1068	Human	Northeastern	0	0	0	0	0	0
1069	Human	Central	0	0	0	0	0	0
1070	Human	Southern	0	0	0	0	0	0
1071	Human	Northeastern	0	0	0	0	0	0
1072	Human	Northern	0	0	0	0	0	0
1073	Human	Northern	0	0	0	0	0	1
1074	Human	Northern	0	0	0	0	0	0
1075	Human	Northern	0	0	0	0	0	0
1076	Human	Northern	0	0	0	0	0	0
1077	Human	Northern	0	0	0	0	0	0
1078	Human	Northeastern	0	0	0	0	0	0
1079	Human	Northeastern	0	0	0	0	0	1
1080	Human	Northeastern	0	0	0	0	0	0
1081	Human	Northeastern	0	0	0	0	0	0
1082	Human	Northern	0	0	0	0	0	0
1083	Human	Northern	0	0	0	0	0	0
1084	Human	Northern	0	0	0	0	0	0
1085	Human	Northern	0	0	0	0	0	0
1086	Human	Northern	0	0	0	0	0	0
1087	Human	Northern	0	0	0	0	0	0
1088	Human	Northern	0	0	0	0	0	0
1089	Human	Northern	0	0	0	0	0	0
1090	Human	Northern	0	0	0	0	0	0
1091	Human	Northern	0	0	0	0	0	0
1092	Human	Northern	0	0	0	0	0	0
1093	Human	Northern	0	0	0	0	0	0
1094	Human	Northern	0	0	0	0	0	0
1095	Human	Northern	0	0	0	0	0	0
1096	Human	Northern	0	0	0	0	0	0
1097	Human	Northern	0	0	0	0	0	0
1098	Human	Northern	0	0	0	0	0	0
1099	Human	Northern	0	0	0	0	0	0
1100	Human	Northeastern	1	0	0	0	0	1
1101	Human	Northeastern	1	0	0	0	0	1
1102	Human	Northeastern	0	0	0	0	0	0
1103	Human	Northeastern	0	0	0	0	0	0

1104	Human	Northeastern	0	0	0	0	0	0
1105	Human	Northeastern	0	0	0	0	0	0
1106	Human	Northeastern	0	0	0	0	0	0
1107	Human	Northeastern	0	0	0	0	0	0
1108	Human	Northeastern	0	0	0	0	0	0
1109	Human	Northeastern	0	0	0	0	0	0
1110	Human	Northeastern	0	0	0	0	0	0
1111	Human	Northeastern	0	0	0	0	0	0
1112	Human	Northeastern	0	0	0	0	0	0
1113	Human	Northeastern	0	0	0	0	0	0
1114	Human	Northeastern	0	0	0	0	0	0
1115	Human	Northeastern	0	0	0	1	0	1
1116	Human	Northeastern	0	0	0	0	0	0
1117	Human	Northeastern	0	0	0	0	0	0
1118	Human	Northern	0	0	0	0	0	0
1119	Human	Northern	0	0	0	0	0	0
1120	Human	Northern	0	0	0	0	0	0
1121	Human	Northern	0	0	0	0	0	0
1122	Human	Northern	0	0	0	0	0	0
1123	Human	Northern	0	0	0	0	0	0
1124	Human	Northern	0	0	0	0	0	0
1125	Human	Northern	0	0	0	0	0	0
1126	Human	Northern	0	0	0	0	0	0
1127	Human	Northern	0	0	0	0	0	0
1128	Human	Southern	0	0	0	0	0	0
1129	Human	Central	0	0	0	0	0	0
1130	Human	Eastern	0	0	0	0	0	0
1131	Human	Central	0	0	0	0	0	0
1132	Human	Southern	0	0	0	0	0	0
1133	Human	Southern	0	0	0	0	0	0
1134	Human	Southern	0	0	0	0	0	0
1135	Human	Northern	0	0	0	0	0	0
1136	Human	Central	0	0	0	0	0	0
1137	Human	Central	0	0	0	0	0	0
1138	Human	Central	0	0	0	0	0	0
1139	Human	Northeastern	0	0	0	0	0	0
1140	Human	Northeastern	0	0	0	1	0	1
1141	Human	Northeastern	0	0	0	0	0	0
1142	Human	Northeastern	0	0	0	0	0	0
1143	Human	Northeastern	0	0	0	0	0	0
1144	Human	Northeastern	0	0	0	0	0	0
1145	Human	Northeastern	0	0	0	0	0	0
1146	Human	Northeastern	0	0	0	1	0	1
1147	Human	Northeastern	0	0	0	0	0	0
1148	Human	Central	0	0	0	0	0	0
1149	Human	Central	0	0	0	0	0	0
1150	Human	Central	0	0	0	0	0	0
1151	Human	Central	0	0	0	0	0	0

1152	Human	Central	0	0	0	0	0	0
1153	Human	Eastern	0	0	0	0	0	0
1154	Human	Southern	0	1	0	1	0	1
1155	Human	Southern	0	0	0	0	0	0
1156	Human	Central	0	0	0	0	0	0
1157	Human	Southern	0	0	0	0	0	0
1158	Human	Southern	0	0	0	0	0	0
1159	Human	Central	0	0	0	0	0	0
1160	Human	Eastern	0	0	0	0	0	0
1161	Human	Southern	0	0	0	0	0	0
1162	Human	Northeastern	0	0	0	0	0	0
1163	Human	Central	0	0	0	0	0	0
1164	Human	Northeastern	0	0	0	0	0	0
1165	Human	Northeastern	0	0	0	0	0	0
1166	Human	Northeastern	0	0	0	0	0	0
1167	Human	Northeastern	0	0	0	0	0	0
1168	Human	Northeastern	0	0	0	0	0	0
1169	Human	Northeastern	0	0	0	0	0	0
1170	Human	Northeastern	0	0	0	0	0	0
1171	Human	Northeastern	0	0	0	0	0	0
1172	Human	Northeastern	0	0	0	1	0	1
1173	Human	Northeastern	0	0	0	0	0	0
1174	Human	Northeastern	0	0	0	0	0	0
1175	Human	Eastern	0	0	0	0	0	0
1176	Human	Eastern	0	1	0	1	0	1
1177	Human	Eastern	0	0	0	1	0	1
1178	Human	Eastern	0	0	0	0	0	1
1179	Human	Eastern	0	0	0	0	0	0
1180	Human	Eastern	0	0	0	1	0	1
1181	Human	Eastern	1	0	0	0	0	1
1182	Human	Eastern	1	0	0	0	0	1
1183	Human	Eastern	0	0	0	0	0	1
1184	Human	Eastern	1	0	0	0	0	1
1185	Human	Eastern	0	1	0	1	0	1
1186	Human	Eastern	0	1	0	1	0	1
1187	Human	Northern	0	1	0	0	0	1
1188	Human	Northern	0	1	0	0	0	1
1189	Human	Northern	0	0	0	0	0	0
1190	Human	Northern	0	0	0	1	0	1
1191	Human	Northern	0	0	0	0	0	0
1192	Human	Northern	0	0	0	1	0	1
1193	Human	Northern	0	0	0	0	0	0
1194	Human	Northern	0	1	0	0	0	1
1195	Human	Northern	1	0	0	0	0	1
1196	Human	Northern	1	0	0	0	0	1
1197	Human	Northern	0	1	0	0	0	1
1198	Human	Northern	0	0	0	0	0	1
1199	Human	Northern	0	0	0	0	0	0

1200	Human	Northern	0	0	0	1	0	1
1201	Human	Northern	0	0	0	0	0	0
1202	Human	Northern	0	0	0	1	0	1
1203	Human	Northern	0	0	0	1	0	1
1204	Human	Northern	0	0	0	1	0	1
1205	Human	Northern	0	0	0	1	0	1
1206	Human	Northern	0	0	0	1	0	1
1207	Human	Northern	0	0	0	0	0	0
1208	Human	Northern	0	0	0	0	0	0
1209	Human	Northern	0	0	0	1	0	1
1210	Human	Northern	1	0	0	0	0	1
1211	Human	Northern	0	0	1	0	0	1
1212	Human	Northern	0	0	1	0	0	1
1213	Human	Northern	0	0	0	0	0	0
1214	Human	Northern	0	0	0	0	0	0
1215	Human	Southern	0	0	0	0	0	0
1216	Human	Southern	0	0	0	0	0	0
1217	Human	Southern	0	0	0	0	0	0
1218	Human	Southern	0	0	0	0	0	0
1219	Human	Southern	0	0	0	0	0	0
1220	Human	Southern	0	0	0	0	0	0
1221	Human	Southern	0	0	0	0	0	0
1222	Human	Southern	0	0	0	0	0	0
1223	Human	Southern	0	0	0	0	0	0
1224	Human	Southern	0	0	0	0	0	0
1225	Human	Southern	0	0	0	0	0	0
1226	Human	Southern	0	0	0	0	0	0
1227	Human	Southern	0	0	0	0	0	0
1228	Human	Southern	0	0	0	0	0	0
1229	Human	Southern	0	0	0	0	0	0
1230	Human	Southern	0	0	0	0	0	0
1231	Human	Southern	0	0	0	0	0	0
1232	Human	Southern	0	0	0	1	0	1
1233	Human	Southern	0	0	0	0	0	0
1234	Human	Southern	0	0	0	0	0	0
1235	Human	Southern	0	0	0	0	0	0
1236	Human	Southern	0	0	0	1	0	1
1237	Human	Northeastern	0	0	0	0	0	0
1238	Human	Northeastern	0	0	0	0	0	0
1239	Human	Northeastern	0	0	0	0	0	0
1240	Human	Northeastern	0	0	0	0	0	0
1241	Human	Northeastern	0	0	0	0	0	0
1242	Human	Northeastern	0	0	0	0	0	0
1243	Human	Northeastern	0	0	0	0	0	0
1244	Human	Northeastern	0	0	0	0	0	0
1245	Human	Northeastern	0	0	0	0	0	0
1246	Human	Northeastern	0	0	0	0	0	0
1247	Human	Northeastern	0	0	0	0	0	0

1248	Human	Northeastern	1	0	0	0	0	1
1249	Human	Northeastern	0	0	0	0	0	0
1250	Human	Northeastern	0	0	0	1	0	1
1251	Human	Northeastern	0	0	0	0	0	0
1252	Human	Northeastern	0	0	0	0	0	0
1253	Human	Northeastern	0	0	0	0	0	0
1254	Human	Northeastern	0	0	0	0	0	0
1255	Human	Northeastern	0	0	0	0	0	0
1256	Human	Central	0	0	0	0	0	0
1257	Human	Southern	0	0	0	0	0	0
1258	Human	Northeastern	0	0	0	0	0	0
1259	Human	Central	0	0	0	0	0	0
1260	Human	Central	0	0	0	0	0	1
1261	Human	Central	0	0	0	0	0	0
1262	Human	Central	0	0	0	0	0	1
1263	Human	Central	0	0	0	0	0	0
1264	Human	Central	0	0	0	0	0	0
1265	Human	Northern	0	0	0	0	0	0
1266	Human	Northeastern	0	0	0	1	0	1
1267	Human	Northeastern	0	0	0	0	0	0
1268	Human	Northeastern	0	0	0	0	0	0
1269	Human	Northeastern	0	0	0	1	0	1
1270	Human	Northeastern	0	0	0	0	0	0
1271	Human	Northeastern	0	0	0	0	0	0
1272	Human	Northeastern	0	0	0	0	0	0
1273	Human	Central	0	0	0	0	0	0
1274	Human	Central	0	0	0	0	0	0
1275	Human	Central	0	0	0	0	0	0
1276	Human	Central	0	0	0	0	0	0
1277	Human	Southern	0	0	0	0	0	0
1278	Human	Southern	0	0	0	0	0	0
1279	Human	Southern	0	0	0	0	0	0
1280	Human	Southern	1	0	0	0	0	1
1281	Human	Southern	0	0	0	0	0	0
1282	Human	Southern	0	0	0	1	0	1
1283	Human	Southern	0	0	0	0	0	0
1284	Human	Southern	0	0	0	0	0	0
1285	Human	Central	0	0	0	0	0	0
1286	Human	Central	0	0	0	0	0	0
1287	Human	Central	0	0	0	0	0	0
1288	Human	Northeastern	0	0	0	0	0	0
1289	Human	Northeastern	0	0	0	0	0	0
1290	Human	Northeastern	0	0	0	0	0	0
1291	Human	Central	0	0	0	0	0	0
1292	Human	Central	0	0	0	0	0	0
1293	Human	Central	0	0	0	0	0	0
1294	Human	Central	0	0	0	0	0	0
1295	Human	Central	0	0	0	0	0	0

1296	Human	Central	0	0	0	0	0	0
1297	Human	Central	0	0	0	0	0	0
1298	Human	Central	0	0	0	0	0	0
1299	Human	Central	0	0	0	0	0	0
1300	Human	Central	0	0	0	0	0	0
1301	Human	Central	0	0	0	0	0	0
1302	Human	Northeastern	0	0	0	0	0	0
1303	Human	Southern	0	0	0	0	0	0
1304	Human	Central	0	0	0	0	0	0
1305	Human	Central	0	0	0	0	0	0
1306	Human	Central	0	0	0	0	0	0
1307	Human	Central	0	0	0	0	0	0
1308	Human	Central	0	0	0	0	0	0
1309	Human	Central	0	0	0	0	0	0
1310	Human	Central	0	0	0	0	0	0
1311	Human	Central	0	0	0	0	0	0
1312	Human	Central	0	0	0	0	0	0
1313	Human	Central	0	0	0	0	0	0
1314	Human	Central	0	0	0	0	0	0
1315	Human	Central	0	0	0	0	0	0
1316	Human	Central	0	0	0	0	0	0
1317	Human	Central	0	0	0	0	0	0
1318	Human	Central	0	0	0	0	0	0
1319	Human	Central	0	0	0	0	0	0
1320	Human	Northern	0	0	0	0	0	0
1321	Human	Northern	0	0	0	0	0	0
1322	Human	Northern	0	0	0	0	0	0
1323	Human	Northern	1	0	0	0	0	1
1324	Human	Northern	0	0	0	0	0	0
1325	Human	Northern	0	0	0	1	0	1
1326	Human	Northern	0	0	0	0	0	0
1327	Human	Northern	0	0	0	1	0	1
1328	Human	Northern	0	0	0	0	0	0
1329	Human	Northern	0	0	0	0	0	0
1330	Human	Northern	0	0	0	0	0	0
1331	Human	Northern	0	0	0	0	0	1
1332	Human	Northern	0	0	0	0	0	0
1333	Human	Northern	0	0	0	0	0	0
1334	Human	Northern	0	0	0	0	0	0
1335	Human	Northern	0	0	0	1	0	1
1336	Human	Northeastern	0	0	0	0	0	0
1337	Human	Northeastern	0	0	0	1	0	1
1338	Human	Northeastern	0	0	0	0	0	0
1339	Human	Northeastern	0	0	0	1	0	1
1340	Human	Northeastern	0	0	0	0	0	0
1341	Human	Eastern	0	0	0	0	0	0
1342	Human	Eastern	0	0	0	1	0	1
1343	Human	Eastern	0	0	0	0	0	0

1344	Human	Eastern	0	0	0	0	0	0
1345	Human	Eastern	0	0	0	0	0	0
1346	Human	Eastern	0	0	0	0	0	0
1347	Human	Eastern	0	0	0	1	0	1
1348	Human	Eastern	0	0	0	1	0	1
1349	Human	Central	0	0	0	0	0	0
1350	Human	Central	0	0	0	0	0	0
1351	Human	Northern	0	0	0	0	0	0
1352	Human	Northern	0	0	0	0	0	0
1353	Human	Northern	0	0	0	0	0	0
1354	Human	Northern	1	0	0	0	0	1
1355	Human	Northern	0	0	0	0	0	0
1356	Human	Northern	0	0	0	0	0	0
1357	Human	Northern	0	0	0	1	0	1
1358	Human	Northern	1	0	0	1	0	1
1359	Human	Northern	0	0	0	0	0	0
1360	Human	Northern	0	0	0	1	0	1
1361	Human	Northern	0	0	0	0	0	0
1362	Human	Northern	0	0	0	0	0	0
1363	Human	Northern	0	0	0	1	0	1
1364	Human	Northern	0	0	0	1	0	1
1365	Human	Northern	0	0	0	0	0	0
1366	Human	Northern	0	0	0	0	0	0
1367	Human	Southern	0	0	0	0	0	0
1368	Human	Southern	0	0	0	0	0	0
1369	Human	Southern	0	0	0	0	0	0
1370	Human	Southern	0	0	0	1	0	1
1371	Human	Southern	0	0	0	0	0	0
1372	Human	Southern	0	0	0	1	0	1
1373	Human	Southern	0	0	0	0	0	0
1374	Human	Southern	0	1	0	1	0	1
1375	Human	Southern	0	0	0	0	0	0
1376	Human	Southern	0	0	0	0	0	0
1377	Human	Southern	0	0	0	0	0	0
1378	Human	Southern	0	0	0	0	0	0
1379	Human	Southern	0	0	0	0	0	0
1380	Human	Southern	0	0	0	0	0	0
1381	Human	Southern	0	0	0	0	0	0
1382	Human	Northeastern	0	0	0	0	0	0
1383	Human	Northeastern	0	0	0	1	0	1
1384	Human	Northeastern	0	0	0	1	0	1
1385	Human	Northeastern	0	0	0	1	0	1
1386	Human	Northeastern	0	0	0	0	0	0
1387	Human	Northeastern	0	0	0	0	0	0
1388	Human	Northeastern	0	0	0	0	0	0
1389	Human	Northeastern	0	0	0	0	0	0
1390	Human	Northeastern	0	0	0	0	0	0
1391	Human	Northeastern	0	0	0	0	0	0

1392	Human	Northeastern	0	0	0	0	0	0
1393	Human	Northeastern	0	0	0	0	0	1
1394	Human	Northeastern	0	0	0	0	0	0
1395	Human	Northeastern	0	0	0	0	0	0
1396	Human	Northeastern	0	0	0	0	0	0
1397	Human	Northeastern	0	0	0	0	0	0
1398	Human	Northeastern	0	0	0	0	0	0
1399	Human	Northeastern	0	0	0	0	0	0
1400	Human	Northeastern	0	0	0	0	0	0
1401	Human	Northeastern	0	0	0	0	0	0
1402	Human	Northeastern	0	0	0	0	0	0
1403	Human	Northeastern	0	0	0	0	0	0
1404	Human	Northeastern	0	0	0	0	0	0
1405	Human	Northeastern	0	0	0	0	0	0
1406	Human	Northeastern	0	0	0	0	0	0
1407	Human	Northeastern	0	0	0	0	0	0
1408	Human	Northeastern	0	0	0	1	0	1
1409	Human	Northeastern	0	0	0	0	0	1
1410	Human	Northeastern	0	0	0	0	0	0
1411	Human	Northeastern	0	0	0	1	0	1
1412	Human	Northeastern	0	0	0	0	0	0
1413	Human	Eastern	0	0	0	0	0	0
1414	Human	Northeastern	0	0	0	0	0	0
1415	Human	Northeastern	0	0	0	0	0	0
1416	Human	Northeastern	0	0	0	0	0	0
1417	Human	Northeastern	0	0	0	1	0	1
1418	Human	Northeastern	0	0	0	1	0	1
1419	Human	Northeastern	0	0	0	0	0	0
1420	Human	Northeastern	0	0	0	1	0	1
1421	Human	Northeastern	0	0	0	1	0	1
1422	Human	Central	0	0	0	0	0	0
1423	Human	Southern	0	0	0	0	0	0
1424	Human	Central	0	0	0	0	0	0
1425	Human	Central	0	0	0	0	0	0
1426	Human	Central	0	0	0	0	0	0
1427	Human	Central	0	0	0	0	0	0
1428	Human	Central	0	0	0	0	0	0
1429	Human	Central	0	0	0	0	0	0
1430	Human	Central	0	0	0	0	0	0
1431	Human	Central	0	0	0	0	0	0
1432	Human	Central	0	0	0	0	0	0
1433	Human	Central	0	0	0	0	0	0
1434	Human	Central	0	0	0	0	0	0
1435	Human	Central	0	0	0	0	0	0
1436	Human	Central	0	0	0	0	0	0
1437	Human	Central	0	0	0	0	0	0
1438	Human	Central	0	0	0	0	0	0
1439	Human	Central	0	0	0	0	0	0

1440	Human	Central	0	0	0	0	0	0
1441	Human	Central	0	0	0	0	0	0
1442	Human	Central	0	0	0	0	0	0
1443	Human	Central	0	0	0	0	0	0
1444	Human	Central	0	0	0	0	0	0
1445	Human	Central	0	0	0	1	0	1
1446	Human	Northeastern	0	0	0	0	0	0
1447	Human	Northeastern	0	0	0	0	0	0
1448	Human	Northeastern	0	0	0	0	0	0
1449	Human	Central	0	0	0	0	0	0
1450	Human	Central	0	0	0	0	0	0
1451	Human	Northern	0	0	0	0	0	0
1452	Human	Central	0	0	0	0	0	0
1453	Human	Central	0	0	0	0	0	0
1454	Human	Eastern	0	0	0	0	0	0
1455	Human	Southern	0	0	0	0	0	0
1456	Human	Southern	0	0	0	0	0	0
1457	Human	Central	0	0	0	0	0	0
1458	Human	Central	0	0	0	0	0	0
1459	Human	Northeastern	0	1	0	1	0	1
1460	Human	Northeastern	0	0	0	0	0	0
1461	Human	Northeastern	0	0	0	0	0	0
1462	Human	Northeastern	1	0	0	0	0	1
1463	Human	Northeastern	0	0	0	0	0	0
1464	Human	Central	0	0	0	0	0	0
1465	Human	Northeastern	0	0	0	1	0	1
1466	Human	Northeastern	0	0	0	0	0	0
1467	Human	Northeastern	1	0	0	0	0	1
1468	Human	Northeastern	1	0	0	1	0	1
1469	Human	Northeastern	1	0	0	1	0	1
1470	Human	Northeastern	1	0	0	0	0	1
1471	Human	Northeastern	0	0	0	0	0	1
1472	Human	Northeastern	0	0	0	1	0	1
1473	Human	Northeastern	0	0	0	0	0	1
1474	Human	Northeastern	1	0	0	1	0	1
1475	Human	Northeastern	0	0	0	1	0	1
1476	Human	Northeastern	0	0	0	0	0	0
1477	Human	Northeastern	0	0	0	1	0	1
1478	Human	Northeastern	0	0	0	0	0	0
1479	Human	Northeastern	0	0	0	0	0	0
1480	Human	Northeastern	0	0	0	1	0	1
1481	Human	Northeastern	0	0	0	0	0	0
1482	Human	Northeastern	0	0	0	1	0	1
1483	Human	Northeastern	0	0	0	1	0	1
1484	Human	Northeastern	1	0	0	1	0	1
1485	Human	Northeastern	1	0	0	1	0	1
1486	Human	Northeastern	0	0	0	1	0	1
1487	Human	Northeastern	1	0	0	0	0	1

1488	Human	Northeastern	0	0	0	0	0	0
1489	Human	Northeastern	0	0	0	1	0	1
1490	Human	Northeastern	0	0	0	1	0	1
1491	Human	Northeastern	0	0	0	1	0	1
1492	Human	Northeastern	0	0	0	1	0	1
1493	Human	Northeastern	0	0	0	1	0	1
1494	Human	Northeastern	0	0	0	1	0	1
1495	Human	Northeastern	0	0	0	1	0	1
1496	Human	Northeastern	0	0	0	0	0	1
1497	Human	Northeastern	0	0	0	1	0	1
1498	Human	Northeastern	0	0	0	1	0	1
1499	Human	Northeastern	0	0	0	1	0	1
1500	Human	Northeastern	0	0	0	1	0	1
1501	Human	Northeastern	0	0	0	1	0	1
1502	Human	Northeastern	0	0	0	1	0	1
1503	Human	Northeastern	1	0	0	1	0	1
1504	Human	Northeastern	0	0	0	1	0	1
1505	Human	Northeastern	0	0	0	1	0	1
1506	Human	Northeastern	0	0	0	1	0	1
1507	Human	Northeastern	0	0	0	1	0	1
1508	Human	Northeastern	1	0	0	1	0	1
1509	Human	Northeastern	0	0	0	1	0	1
1510	Human	Northeastern	1	0	0	1	0	1
1511	Human	Northeastern	0	0	0	0	0	1
1512	Human	Northeastern	0	0	0	0	0	0
1513	Human	Northeastern	0	0	0	0	0	0
1514	Human	Northeastern	0	0	0	1	0	1
1515	Human	Northeastern	0	0	0	0	0	0
1516	Human	Northeastern	0	0	0	1	0	1
1517	Human	Northeastern	0	0	0	1	0	1
1518	Human	Northeastern	1	0	0	1	0	1
1519	Human	Northeastern	0	0	0	0	0	0
1520	Human	Northeastern	0	0	0	1	0	1
1521	Human	Northeastern	0	0	0	1	0	1
1522	Human	Northeastern	0	0	0	1	0	1
1523	Human	Northeastern	0	0	0	0	0	0
1524	Human	Northeastern	0	0	0	1	0	1
1525	Human	Northeastern	0	1	0	1	0	1
1526	Human	Northeastern	0	0	0	0	0	0
1527	Human	Northeastern	0	1	0	0	0	1
1528	Human	Northeastern	0	0	0	1	0	1
1529	Human	Northeastern	0	0	0	0	0	0
1530	Human	Central	0	0	0	0	0	0
1531	Human	Eastern	0	0	0	0	0	0
1532	Human	Central	0	0	0	0	0	0
1533	Human	Central	0	0	0	0	0	0
1534	Human	Central	0	0	0	0	0	0
1535	Human	Central	0	0	0	0	0	0

1536	Human	Central	0	0	0	0	0	0
1537	Human	Central	0	0	0	0	0	0
1538	Human	Central	0	0	0	0	0	0
1539	Human	Northern	0	0	0	0	0	0
1540	Human	Southern	0	0	0	0	0	0
1541	Human	Southern	0	0	0	0	0	0
1542	Human	Southern	0	0	0	0	0	0
1543	Human	Central	0	0	0	0	0	0
1544	Human	Central	0	0	0	0	0	0
1545	Human	Central	0	0	0	0	0	0
1546	Human	Central	0	0	0	0	0	0
1547	Human	Central	0	0	0	0	0	0
1548	Human	Central	0	0	0	0	0	0
1549	Human	Northeastern	0	0	0	0	0	0
1550	Human	Central	0	0	0	0	0	0
1551	Human	Central	0	0	0	0	0	0
1552	Human	Central	0	0	0	0	0	0
1553	Human	Central	0	0	0	0	0	0
1554	Human	Central	0	0	0	0	0	0
1555	Human	Eastern	0	0	0	0	0	0
1556	Human	Central	0	0	0	0	0	0
1557	Human	Central	0	0	0	0	0	0
1558	Human	Northeastern	0	0	0	0	0	0
1559	Human	Northeastern	0	0	0	0	0	0
1560	Human	Northeastern	0	0	0	0	0	0
1561	Human	Northeastern	0	0	0	0	0	0
1562	Human	Northeastern	0	0	0	0	0	0
1563	Human	Northeastern	0	0	0	0	0	0
1564	Human	Eastern	0	0	0	0	0	0
1565	Human	Central	0	0	0	0	0	0
1566	Human	Central	0	0	0	0	0	0
1567	Human	Eastern	0	0	0	0	0	0
1568	Human	Central	0	0	0	0	0	0
1569	Human	Central	0	0	0	0	0	0
1570	Human	Central	0	0	0	0	0	0
1571	Human	Central	0	0	0	0	0	0
1572	Human	Central	0	0	0	0	0	0
1573	Human	Central	0	0	0	0	0	0
1574	Human	Central	0	0	0	0	0	0
1575	Human	Central	0	0	0	0	0	0
1576	Human	Central	0	0	0	0	0	0
1577	Human	Central	0	0	0	0	0	0
1578	Human	Central	0	0	0	0	0	0
1579	Human	Central	0	0	0	0	0	0
1580	Human	Central	0	0	0	0	0	0
1581	Human	Central	0	0	0	0	0	0
1582	Human	Northeastern	0	0	0	0	0	0
1583	Human	Northeastern	0	0	0	0	0	0

1584	Human	Central	0	0	0	0	0	0
1585	Human	Central	0	0	0	0	0	0
1586	Human	Southern	0	0	0	0	0	0
1587	Human	Central	0	0	0	0	0	0
1588	Human	Central	0	0	0	0	0	0
1589	Human	Central	0	0	0	0	0	0
1590	Human	Central	0	0	0	0	0	0
1591	Human	Northeastern	0	0	0	0	0	0
1592	Human	Northeastern	0	0	0	0	0	1
1593	Human	Northeastern	0	0	0	0	0	0
1594	Human	Northeastern	0	0	0	0	0	0
1595	Human	Northeastern	0	0	0	0	0	1
1596	Human	Central	0	0	0	0	0	0
1597	Human	Central	0	0	0	0	0	0
1598	Human	Central	0	0	0	0	0	0
1599	Human	Central	0	0	0	0	0	0
1600	Human	Eastern	0	0	0	0	0	0
1601	Human	Central	0	0	0	0	0	0
1602	Human	Northern	0	0	0	0	0	0
1603	Human	Central	0	0	0	0	0	0
1604	Human	Northeastern	0	0	0	0	0	0
1605	Human	Northeastern	0	0	0	0	0	0
1606	Human	Northeastern	0	0	0	0	0	0
1607	Human	Northeastern	0	0	0	0	0	0
1608	Human	Northeastern	0	0	0	0	0	0
1609	Human	Northern	0	0	0	0	0	0
1610	Human	Northern	0	0	0	0	0	0
1611	Human	Central	0	0	0	0	0	0
1612	Human	Central	0	0	0	0	0	0
1613	Human	Central	0	0	0	0	0	0
1614	Human	Central	0	0	0	0	0	0
1615	Human	Northeastern	0	0	0	0	0	0
1616	Human	Central	0	0	0	0	0	0
1617	Human	Central	0	0	0	0	0	0
1618	Human	Northeastern	0	0	0	0	0	0
1619	Human	Eastern	0	0	0	0	0	0
1620	Human	Central	0	0	0	0	0	0
1621	Human	Central	0	0	0	0	0	0
1622	Human	Central	0	0	0	0	0	0
1623	Human	Eastern	0	0	0	0	0	0
1624	Human	Eastern	0	0	0	0	0	0
1625	Human	Central	0	0	0	0	0	0
1626	Human	Eastern	0	0	0	0	0	0
1627	Human	Central	0	0	0	0	0	0
1628	Human	Central	0	0	0	0	0	0
1629	Human	Central	0	0	0	0	0	0
1630	Human	Central	0	0	0	0	0	0
1631	Human	Southern	0	0	0	0	0	0

1632	Human	Southern	0	0	0	0	0	0
1633	Human	Central	0	0	0	0	0	0
1634	Human	Central	0	0	0	0	0	0
1635	Human	Central	0	0	0	0	0	0
1636	Human	Central	0	0	0	0	0	0
1637	Human	Central	0	0	0	0	0	0
1638	Human	Central	0	0	0	0	0	0
1639	Human	Central	0	0	0	0	0	0
1640	Human	Central	0	0	0	0	0	0
1641	Human	Eastern	0	0	0	0	0	0
1642	Human	Central	0	0	0	0	0	0
1643	Human	Eastern	0	0	0	0	0	0
1644	Human	Central	0	0	0	0	0	0
1645	Human	Southern	0	0	0	1	0	1
1646	Human	Central	0	0	0	0	0	0
1647	Human	Central	0	0	0	0	0	0
1648	Human	Central	0	0	0	0	0	0
1649	Human	Eastern	0	0	0	0	0	0
1650	Human	Central	0	0	0	0	0	0
1651	Human	Central	0	0	0	0	0	0
1652	Human	Central	0	0	0	0	0	0
1653	Human	Eastern	0	0	0	0	0	0
1654	Human	Central	0	0	0	0	0	0
1655	Human	Eastern	0	0	0	0	0	0
1656	Human	Southern	0	0	0	0	0	0
1657	Human	Southern	0	0	0	0	0	0
1658	Human	Southern	0	0	0	0	0	0
1659	Human	Central	0	0	0	0	0	0
1660	Human	Central	0	0	0	0	0	0
1661	Human	Central	0	0	0	0	0	0
1662	Human	Northeastern	0	0	0	0	0	0
1663	Human	Northeastern	0	0	0	1	0	1
1664	Human	Northeastern	0	0	0	1	0	1
1665	Human	Northeastern	1	0	0	0	0	1
1666	Human	Northeastern	0	0	0	0	0	0
1667	Human	Northeastern	0	0	0	1	0	1
1668	Human	Central	0	0	0	0	0	0
1669	Human	Central	0	0	0	0	0	0
1670	Human	Central	0	0	0	0	0	0
1671	Human	Central	0	0	0	0	0	0
1672	Human	Central	0	0	0	0	0	0
1673	Human	Central	0	0	0	0	0	0
1674	Human	Central	0	0	0	0	0	0
1675	Human	Eastern	0	0	0	0	0	0
1676	Human	Central	0	0	0	0	0	0
1677	Human	Central	0	0	0	0	0	0
1678	Human	Northeastern	0	0	0	0	0	0
1679	Human	Central	0	0	0	0	0	0

1680	Human	Northeastern	0	0	0	0	0	0
1681	Human	Central	0	0	0	0	0	0
1682	Human	Central	0	0	0	0	0	0
1683	Human	Central	0	0	0	0	0	0
1684	Human	Central	0	0	0	0	0	0
1685	Human	Central	0	0	0	0	0	0
1686	Human	Northeastern	0	0	0	0	0	0
1687	Human	Central	0	0	0	1	0	1
1688	Human	Central	0	0	0	0	0	0
1689	Human	Central	0	0	0	0	0	0
1690	Human	Northeastern	0	0	0	0	0	0
1691	Human	Central	0	0	0	0	0	0
1692	Human	Central	0	0	0	0	0	0
1693	Human	Central	0	0	0	0	0	0
1694	Human	Northeastern	0	0	0	0	0	0
1695	Human	Central	0	0	0	0	0	0
1696	Human	Central	0	0	0	0	0	0
1697	Human	Central	0	0	0	0	0	0
1698	Human	Central	0	0	0	0	0	0
1699	Human	Central	0	0	0	0	0	0
1700	Human	Central	0	0	0	0	0	0
1701	Human	Central	0	0	0	0	0	0
1702	Human	Central	0	0	0	0	0	0
1703	Human	Central	0	0	0	0	0	0
1704	Human	Central	0	0	0	0	0	0
1705	Human	Northeastern	0	0	0	0	0	0
1706	Human	Central	0	0	0	0	0	0
1707	Human	Central	0	0	0	0	0	0
1708	Human	Central	0	0	0	0	0	0
1709	Human	Central	0	0	0	0	0	0
1710	Human	Central	0	0	0	0	0	0
1711	Human	Central	0	0	0	0	0	0
1712	Human	Central	0	0	0	0	0	0
1713	Human	Central	0	0	0	0	0	0
1714	Human	Eastern	0	0	0	0	0	0
1715	Human	Central	0	0	0	0	0	0
1716	Human	Central	0	0	0	0	0	0
1717	Human	Eastern	0	0	0	0	0	0
1718	Human	Eastern	0	0	0	0	0	0
1719	Human	Central	0	0	0	0	0	0
1720	Human	Central	0	0	0	0	0	0
1721	Human	Northeastern	0	0	0	0	0	0
1722	Human	Central	0	0	0	0	0	0
1723	Human	Eastern	0	0	0	0	0	0
1724	Human	Central	0	0	0	0	0	0
1725	Human	Central	0	0	0	0	0	0
1726	Human	Central	0	0	0	0	0	0
1727	Human	Central	0	0	0	0	0	0

1728	Human	Central	0	0	0	0	0	0
1729	Human	Northeastern	0	0	0	0	0	0
1730	Human	Northeastern	0	0	0	0	0	0
1731	Human	Northeastern	0	0	0	0	0	0
1732	Human	Northeastern	0	0	0	0	0	0
1733	Human	Central	0	0	0	0	0	0
1734	Human	Northeastern	0	0	0	0	0	0
1735	Human	Central	0	0	0	0	0	0
1736	Human	Southern	0	0	0	0	0	0
1737	Human	Central	0	0	0	0	0	0
1738	Human	Central	0	0	0	0	0	0
1739	Human	Central	0	0	0	0	0	0
1740	Human	Central	0	0	0	0	0	0
1741	Human	Central	0	0	0	0	0	0
1742	Human	Central	0	0	0	0	0	0
1743	Human	Central	0	0	0	0	0	0
1744	Human	Central	0	0	0	0	0	0
1745	Human	Eastern	0	0	0	0	0	0
1746	Human	Central	0	0	0	0	0	0
1747	Human	Central	0	0	0	0	0	0
1748	Human	Central	0	0	0	0	0	0
1749	Human	Northeastern	0	0	0	0	0	0
1750	Human	Central	0	0	0	0	0	0
1751	Human	Eastern	0	0	0	0	0	0
1752	Human	Central	0	0	0	0	0	0
1753	Human	Central	0	0	0	0	0	0
1754	Human	Eastern	0	0	0	0	0	0
1755	Human	Central	0	0	0	0	0	0
1756	Human	Central	0	0	0	0	0	0
1757	Human	Central	0	0	0	0	0	0
1758	Human	Central	0	0	0	0	0	0
1759	Human	Central	0	0	0	0	0	0
1760	Human	Central	0	0	0	0	0	0
1761	Human	Central	0	0	0	0	0	0
1762	Human	Central	0	0	0	0	0	0
1763	Human	Central	0	0	0	0	0	0
1764	Human	Central	0	0	0	0	0	0
1765	Human	Central	0	0	0	0	0	0
1766	Human	Northeastern	0	0	0	0	0	0
1767	Human	Central	0	0	0	0	0	0
1768	Human	Central	0	0	0	0	0	0
1769	Human	Central	0	0	0	0	0	0
1770	Human	Central	0	0	0	0	0	0
1771	Human	Central	0	0	0	0	0	0
1772	Human	Central	0	0	0	0	0	0
1773	Human	Central	0	0	0	0	0	0
1774	Human	Central	0	0	0	1	0	1
1775	Human	Central	0	0	0	0	0	0

1776	Human	Central	0	0	0	0	0	0
1777	Human	Central	0	0	0	0	0	0
1778	Human	Central	0	0	0	0	0	0
1779	Human	Central	0	0	0	0	0	0
1780	Human	Southern	0	0	0	0	0	0
1781	Human	Central	0	0	0	0	0	0
1782	Human	Central	0	0	0	0	0	0
1783	Human	Central	0	0	0	0	0	0
1784	Human	Eastern	0	0	0	0	0	0
1785	Human	Central	0	0	0	0	0	0
1786	Human	Central	0	0	0	0	0	0
1787	Human	Central	0	0	0	0	0	0
1788	Human	Central	0	0	0	0	0	0
1789	Human	Eastern	0	0	0	0	0	0
1790	Human	Southern	0	0	0	0	0	0
1791	Human	Southern	0	0	0	0	0	0
1792	Human	Eastern	0	0	0	0	0	0
1793	Human	Central	0	0	0	0	0	0
1794	Human	Central	0	0	0	0	0	0
1795	Human	Northeastern	0	0	0	0	0	0
1796	Human	Central	0	0	0	0	0	0
1797	Human	Central	0	0	0	0	0	0
1798	Human	Central	0	0	0	0	0	0
1799	Human	Eastern	0	0	0	0	0	0
1800	Human	Central	0	0	0	0	0	0
1801	Human	Central	0	0	0	0	0	0
1802	Human	Central	0	0	0	0	0	0
1803	Human	Central	0	0	0	0	0	0
1804	Human	Central	0	0	0	0	0	0
1805	Human	Central	0	0	0	0	0	0
1806	Human	Central	0	0	0	0	0	0
1807	Human	Central	0	0	0	0	0	0
1808	Human	Eastern	0	0	0	0	0	0
1809	Human	Central	0	0	0	0	0	0
1810	Human	Southern	0	0	0	0	0	0
1811	Human	Southern	0	0	0	0	0	0
1812	Human	Southern	0	0	0	0	0	0
1813	Human	Northeastern	0	0	0	0	0	0
1814	Human	Central	0	0	0	0	0	0
1815	Human	Central	0	0	0	0	0	0
1816	Human	Central	0	0	0	0	0	0
1817	Human	Central	0	0	0	0	0	0
1818	Human	Central	0	0	0	0	0	0
1819	Human	Eastern	0	0	0	1	0	1
1820	Human	Central	0	0	0	1	0	1
1821	Human	Central	0	0	0	0	0	0
1822	Human	Central	0	0	0	0	0	0
1823	Human	Eastern	0	0	0	0	0	0

1824	Human	Eastern	0	0	0	0	0	0
1825	Human	Central	0	0	0	0	0	0
1826	Human	Central	0	0	0	0	0	0
1827	Human	Southern	0	0	0	0	0	0
1828	Human	Central	0	0	0	0	0	0
1829	Human	Central	0	0	0	0	0	0
1830	Human	Central	0	0	0	0	0	0
1831	Human	Central	0	0	0	0	0	0
1832	Human	Central	0	0	0	0	0	0
1833	Human	Central	0	0	0	0	0	0
1834	Human	Central	0	0	0	0	0	0
1835	Human	Central	0	0	0	0	0	0
1836	Human	Central	0	0	0	0	0	0
1837	Human	Eastern	0	0	0	0	0	0
1838	Human	Eastern	0	0	0	0	0	0
1839	Human	Central	0	0	0	0	0	0
1840	Human	Central	0	0	0	0	0	0
1841	Human	Central	0	0	0	0	0	0
1842	Human	Central	0	0	0	0	0	0
1843	Human	Central	0	0	0	0	0	0
1844	Human	Central	0	0	0	0	0	0
1845	Human	Central	0	0	0	0	0	0
1846	Human	Southern	0	0	0	0	0	0
1847	Human	Southern	0	0	0	0	0	0
1848	Human	Central	0	0	0	0	0	0
1849	Human	Eastern	0	0	0	0	0	0
1850	Human	Central	0	0	0	0	0	0
1851	Human	Central	0	0	0	0	0	0
1852	Human	Central	0	0	0	0	0	0
1853	Human	Northeastern	0	0	0	0	0	0
1854	Human	Central	0	0	0	0	0	0
1855	Human	Central	0	0	0	0	0	0
1856	Human	Central	0	0	0	0	0	0
1857	Human	Central	0	0	0	0	0	0
1858	Human	Central	0	0	0	0	0	0
1859	Human	Central	0	0	0	0	0	0
1860	Human	Eastern	0	0	0	0	0	0
1861	Human	Central	0	0	0	0	0	0
1862	Human	Central	0	0	0	0	0	0
1863	Human	Eastern	0	0	0	0	0	0
1864	Human	Central	0	0	0	0	0	0
1865	Human	Central	0	0	0	0	0	0
1866	Human	Central	0	0	0	0	0	0
1867	Human	Central	0	0	0	0	0	0
1868	Human	Central	0	0	0	0	0	0
1869	Human	Eastern	0	0	0	0	0	0
1870	Human	Central	0	0	0	0	0	0
1871	Human	Central	0	0	0	0	0	0

1872	Human	Central	0	0	0	0	0	0
1873	Human	Southern	0	0	0	1	0	1
1874	Human	Central	0	0	0	0	0	0
1875	Human	Central	0	0	0	0	0	0
1876	Human	Central	0	0	0	0	0	0
1877	Human	Central	0	0	0	0	0	0
1878	Human	Eastern	0	0	0	0	0	0
1879	Human	Central	0	0	0	0	0	0
1880	Human	Central	0	0	0	0	0	0
1881	Human	Southern	0	0	0	0	0	0
1882	Human	Central	0	0	0	0	0	0
1883	Human	Central	0	0	0	0	0	0
1884	Human	Central	0	0	0	0	0	0
1885	Human	Central	0	0	0	0	0	0
1886	Human	Northeastern	0	0	0	0	0	0
1887	Human	Eastern	1	0	0	1	0	1
1888	Human	Central	0	0	0	0	0	0
1889	Human	Central	0	0	0	0	0	0
1890	Human	Central	0	0	0	0	0	0
1891	Human	Central	0	0	0	0	0	0
1892	Human	Central	0	0	0	0	0	0
1893	Human	Central	0	0	0	0	0	0
1894	Human	Central	0	0	0	0	0	0
1895	Human	Central	0	0	0	0	0	0
1896	Human	Eastern	0	0	0	0	0	0
1897	Human	Central	0	0	0	0	0	0
1898	Human	Central	0	0	0	0	0	0
1899	Human	Central	0	0	0	0	0	0
1900	Human	Central	0	0	0	0	0	0
1901	Human	Central	0	0	0	0	0	0
1902	Human	Northeastern	0	0	0	0	0	0
1903	Human	Central	0	0	0	0	0	0
1904	Human	Central	0	0	0	0	0	0
1905	Human	Eastern	0	0	0	0	0	0
1906	Human	Eastern	0	0	0	0	0	0
1907	Human	Central	0	0	0	0	0	0
1908	Human	Central	0	0	0	0	0	0
1909	Human	Central	0	0	0	0	0	0
1910	Human	Central	0	0	0	0	0	0
1911	Human	Eastern	0	0	0	0	0	0
1912	Human	Central	0	0	0	0	0	0
1913	Human	Central	0	0	0	0	0	0
1914	Human	Central	0	0	0	0	0	0
1915	Human	Central	0	0	0	0	0	0
1916	Human	Central	0	0	0	0	0	0
1917	Human	Central	0	0	0	0	0	0
1918	Human	Central	0	0	0	0	0	0
1919	Human	Central	0	0	0	0	0	0

1920	Human	Central	0	0	0	0	0	0
1921	Human	Central	0	0	0	0	0	0
1922	Human	Central	0	0	0	0	0	0
1923	Human	Central	0	0	0	0	0	0
1924	Human	Central	0	0	0	0	0	0
1925	Human	Central	0	0	0	0	0	0
1926	Human	Northern	0	0	0	0	0	0
1927	Human	Central	0	0	0	0	0	0
1928	Human	Central	0	0	0	0	0	0
1929	Human	Central	0	0	0	0	0	0
1930	Human	Central	0	0	0	0	0	0
1931	Human	Central	0	0	0	0	0	0
1932	Human	Central	0	0	0	0	0	0
1933	Human	Central	0	0	0	0	0	0
1934	Human	Eastern	0	0	0	0	0	0
1935	Human	Central	0	0	0	0	0	0
1936	Human	Eastern	0	0	0	0	0	0
1937	Human	Central	0	0	0	0	0	0
1938	Human	Eastern	0	0	0	0	0	0
1939	Human	Eastern	0	0	0	0	0	0
1940	Human	Central	0	0	0	0	0	0
1941	Human	Central	0	0	0	0	0	0
1942	Human	Central	0	0	0	0	0	0
1943	Human	Central	0	0	0	0	0	0
1944	Human	Central	0	0	0	0	0	0
1945	Human	Central	0	0	0	0	0	0
1946	Human	Central	0	0	0	0	0	0
1947	Human	Central	0	0	0	0	0	0
1948	Human	Central	0	0	0	0	0	0
1949	Human	Central	0	0	0	0	0	0
1950	Human	Central	0	0	0	0	0	0
1951	Human	Central	0	0	0	0	0	0
1952	Human	Central	0	0	0	0	0	0
1953	Human	Central	0	0	0	0	0	0
1954	Human	Central	0	0	0	0	0	0
1955	Human	Northeastern	0	0	0	0	0	0
1956	Human	Northern	0	0	0	0	0	0
1957	Human	Central	0	0	0	0	0	0
1958	Human	Central	0	0	0	0	0	0
1959	Human	Central	0	0	0	0	0	0
1960	Human	Central	0	0	0	0	0	0
1961	Human	Central	0	0	0	0	0	0
1962	Human	Central	0	0	0	0	0	0
1963	Human	Central	0	0	0	0	0	0
1964	Human	Central	0	0	0	0	0	0
1965	Human	Central	0	0	0	0	0	0
1966	Human	Eastern	0	0	0	0	0	0
1967	Human	Central	0	0	0	0	0	0

1968	Human	Northern	0	0	0	0	0	0
1969	Human	Central	0	0	0	0	0	0
1970	Human	Central	0	0	0	0	0	0
1971	Human	Central	0	0	0	0	0	0
1972	Human	Eastern	0	0	0	0	0	0
1973	Human	Eastern	0	0	0	0	0	0
1974	Human	Central	0	0	0	0	0	0
1975	Human	Central	0	0	0	0	0	0
1976	Human	Central	0	0	0	0	0	0
1977	Human	Eastern	0	0	0	0	0	0
1978	Human	Central	0	0	0	0	0	0
1979	Human	Eastern	0	0	0	0	0	0
1980	Human	Northeastern	0	0	0	0	0	0
1981	Human	Central	0	0	0	0	0	0
1982	Human	Central	0	0	0	0	0	0
1983	Human	Central	0	0	0	0	0	0
1984	Human	Central	0	0	0	0	0	0
1985	Human	Central	0	0	0	0	0	0
1986	Human	Central	0	0	0	0	0	0
1987	Human	Central	0	0	0	0	0	0
1988	Human	Central	0	0	0	0	0	0
1989	Human	Central	0	0	0	0	0	0
1990	Human	Northeastern	0	0	0	0	0	0
1991	Cattle	Central	0	0	0	0	0	0
1992	Cattle	Central	0	0	0	0	0	0
1993	Cattle	Central	0	0	0	0	0	0
1994	Cattle	Central	0	0	0	0	0	0
1995	Cattle	Central	0	0	0	0	0	0
1996	Cattle	Central	0	0	0	0	0	0
1997	Cattle	Central	0	0	0	0	0	0
1998	Cattle	Central	0	0	0	0	0	0
1999	Cattle	Central	0	0	0	0	0	0
2000	Cattle	Central	0	0	0	0	0	0
2001	Cattle	Central	0	0	0	1	0	1
2002	Cattle	Central	0	0	0	1	0	1
2003	Cattle	Central	0	0	0	0	0	0
2004	Cattle	Central	0	0	0	0	0	0
2005	Cattle	Central	0	0	0	0	0	0
2006	Cattle	Central	0	0	0	0	0	0
2007	Cattle	Central	0	0	0	0	0	0
2008	Cattle	Central	0	0	0	1	0	1
2009	Cattle	Central	0	0	0	1	0	1
2010	Cattle	Central	0	0	0	0	0	0
2011	Cattle	Central	0	0	0	0	0	0
2012	Cattle	Central	0	0	0	0	0	0
2013	Cattle	Central	0	0	0	1	0	1
2014	Cattle	Central	0	0	0	0	0	0
2015	Cattle	Central	0	0	0	0	0	0

2016	Cattle	Central	0	0	0	0	0	0
2017	Cattle	Central	0	0	0	0	0	0
2018	Cattle	Central	0	0	0	0	0	0
2019	Cattle	Central	0	0	0	0	0	0
2020	Cattle	Central	0	0	0	0	0	0
2021	Cattle	Central	0	0	0	0	0	0
2022	Cattle	Central	0	0	1	1	0	1
2023	Cattle	Central	0	0	1	1	0	1
2024	Cattle	Central	0	0	0	0	0	0
2025	Cattle	Central	0	0	0	0	0	0
2026	Cattle	Central	0	0	1	1	0	1
2027	Cattle	Central	0	0	0	0	0	0
2028	Cattle	Central	0	0	0	0	0	0
2029	Cattle	Central	0	0	0	0	0	0
2030	Cattle	Central	0	0	0	0	0	0
2031	Cattle	Central	0	0	0	0	0	0
2032	Cattle	Central	0	0	0	0	0	0
2033	Cattle	Central	0	0	0	0	0	0
2034	Cattle	Central	0	0	0	1	0	1
2035	Cattle	Central	0	0	0	1	0	1
2036	Cattle	Central	0	0	0	0	0	0
2037	Cattle	Central	0	0	1	1	0	1
2038	Cattle	Central	0	0	0	0	0	0
2039	Cattle	Central	0	0	0	1	0	1
2040	Cattle	Central	0	0	1	1	0	1
2041	Cattle	Central	0	0	0	1	0	1
2042	Cattle	Central	0	0	0	1	0	1
2043	Cattle	Central	0	0	0	0	0	0
2044	Cattle	Central	0	0	0	0	0	0
2045	Cattle	Central	0	0	0	0	0	0
2046	Cattle	Central	0	0	0	0	0	0
2047	Cattle	Central	0	0	0	0	0	0
2048	Cattle	Central	0	0	0	0	0	0
2049	Cattle	Central	0	0	0	0	0	0
2050	Cattle	Central	0	0	0	0	0	0
2051	Cattle	Central	0	0	0	0	0	0
2052	Cattle	Central	0	0	0	0	0	0
2053	Cattle	Central	0	0	0	0	0	0
2054	Cattle	Central	0	0	0	0	0	0
2055	Cattle	Central	0	0	0	0	0	0
2056	Cattle	Central	0	0	0	0	0	0
2057	Cattle	Central	0	0	0	0	0	0
2058	Cattle	Central	0	0	0	0	0	0
2059	Cattle	Central	0	0	0	0	0	0
2060	Cattle	Central	0	0	0	0	0	0
2061	Cattle	Central	0	0	0	0	0	0
2062	Cattle	Central	0	0	0	0	0	0
2063	Cattle	Central	0	0	0	0	0	0

2064	Cattle	Central	0	0	0	0	0	0
2065	Cattle	Central	0	0	0	0	0	0
2066	Cattle	Central	0	0	0	0	0	0
2067	Cattle	Central	0	0	0	0	1	1
2068	Cattle	Central	0	0	0	0	0	0
2069	Cattle	Central	0	0	0	0	0	0
2070	Cattle	Central	0	0	0	1	0	1
2071	Cattle	Central	0	0	0	0	0	0
2072	Cattle	Central	0	0	0	0	0	0
2073	Cattle	Central	0	0	0	0	0	0
2074	Cattle	Central	0	0	0	0	0	0
2075	Cattle	Central	0	0	0	0	0	0
2076	Cattle	Central	0	0	0	0	0	0
2077	Cattle	Central	0	0	0	0	0	0
2078	Cattle	Central	0	0	0	0	0	0
2079	Cattle	Central	0	0	0	0	0	0
2080	Cattle	Central	0	0	0	0	0	0
2081	Cattle	Central	0	0	0	0	0	0
2082	Cattle	Central	0	0	0	0	0	0
2083	Cattle	Central	0	0	0	0	0	0
2084	Cattle	Central	0	0	0	0	0	0
2085	Cattle	Central	0	0	0	0	0	0
2086	Cattle	Central	0	0	0	0	0	0
2087	Cattle	Central	0	0	0	0	0	0
2088	Cattle	Central	0	0	0	0	0	0
2089	Cattle	Central	0	0	0	0	0	0
2090	Cattle	Central	0	0	0	0	0	0
2091	Cattle	Central	0	0	0	0	0	0
2092	Cattle	Central	0	0	0	0	0	0
2093	Cattle	Central	0	0	0	0	0	0
2094	Cattle	Central	0	0	0	0	0	0
2095	Cattle	Central	0	0	0	0	0	0
2096	Cattle	Central	0	0	0	0	0	0
2097	Cattle	Central	0	0	0	0	0	0
2098	Cattle	Central	0	0	0	0	0	0
2099	Cattle	Central	0	0	0	0	0	0
2100	Cattle	Central	0	0	0	0	0	0
2101	Cattle	Central	0	0	0	0	0	0
2102	Cattle	Central	0	0	0	0	0	0
2103	Cattle	Central	0	0	0	0	0	0
2104	Cattle	Central	0	0	0	0	0	0
2105	Cattle	Central	0	0	0	0	0	0
2106	Cattle	Central	0	0	0	0	0	0
2107	Cattle	Central	0	0	0	0	0	0
2108	Cattle	Central	0	0	0	0	0	0
2109	Cattle	Northeastern	0	0	0	0	0	0
2110	Cattle	Northeastern	0	0	0	0	0	0
2111	Cattle	Northeastern	0	0	0	0	0	0

2112	Cattle	Northeastern	0	0	0	0	0	0
2113	Cattle	Northeastern	0	0	0	0	0	0
2114	Cattle	Northeastern	0	0	0	0	0	0
2115	Cattle	Northeastern	0	0	0	0	0	0
2116	Cattle	Northeastern	0	0	0	0	0	0
2117	Cattle	Northeastern	0	0	0	0	0	0
2118	Cattle	Northeastern	0	0	0	0	0	0
2119	Cattle	Northeastern	0	0	0	0	0	0
2120	Cattle	Northeastern	0	0	0	0	0	0
2121	Cattle	Northeastern	0	0	0	0	0	0
2122	Cattle	Northeastern	0	0	0	0	0	0
2123	Cattle	Northeastern	0	0	0	0	0	0
2124	Cattle	Northeastern	0	0	0	0	0	0
2125	Cattle	Northeastern	0	0	0	0	0	0
2126	Cattle	Northeastern	0	0	0	0	0	0
2127	Cattle	Northeastern	0	0	0	0	0	0
2128	Cattle	Northeastern	0	0	0	0	0	0
2129	Cattle	Northeastern	0	0	0	0	0	0
2130	Cattle	Northeastern	0	0	0	0	0	0
2131	Cattle	Northeastern	0	0	0	0	0	0
2132	Cattle	Northeastern	0	0	0	0	0	0
2133	Cattle	Northeastern	0	0	0	0	0	0
2134	Cattle	Northeastern	0	0	0	0	0	0
2135	Cattle	Northeastern	0	0	0	0	0	0
2136	Cattle	Northeastern	0	0	0	0	0	0
2137	Cattle	Northeastern	0	0	0	0	0	0
2138	Cattle	Northeastern	0	0	0	0	0	0
2139	Cattle	Northeastern	0	0	0	0	0	0
2140	Cattle	Northeastern	0	0	0	0	0	0
2141	Cattle	Northeastern	0	0	0	0	0	0
2142	Cattle	Northeastern	0	0	0	0	0	0
2143	Cattle	Northeastern	0	0	0	0	0	0
2144	Cattle	Northeastern	0	0	0	0	0	0
2145	Cattle	Northeastern	0	0	0	0	0	0
2146	Cattle	Northeastern	0	0	0	0	0	0
2147	Cattle	Northeastern	0	0	0	0	0	0
2148	Cattle	Northeastern	0	0	0	0	0	0
2149	Cattle	Northeastern	0	0	0	0	0	0
2150	Cattle	Northeastern	0	0	0	0	0	0
2151	Cattle	Northeastern	0	0	0	0	0	0
2152	Cattle	Northeastern	0	0	0	0	0	0
2153	Cattle	Northeastern	0	0	0	0	0	0
2154	Cattle	Northeastern	0	0	0	0	0	0
2155	Cattle	Northeastern	0	1	0	0	0	1
2156	Cattle	Northeastern	0	0	0	0	0	0
2157	Cattle	Northeastern	0	0	0	0	0	0
2158	Cattle	Northeastern	0	0	0	0	0	0
2159	Cattle	Northeastern	0	0	0	0	0	0

2160	Cattle	Northeastern	0	0	0	0	0	0
2161	Cattle	Northeastern	0	0	0	0	0	0
2162	Cattle	Northeastern	0	0	0	0	0	0
2163	Cattle	Northeastern	0	0	0	0	0	0
2164	Cattle	Northeastern	0	0	0	0	0	0
2165	Cattle	Northeastern	0	0	0	0	0	0
2166	Cattle	Northeastern	0	0	0	0	0	0
2167	Cattle	Northeastern	0	0	0	0	0	0
2168	Cattle	Northeastern	0	0	0	0	0	0
2169	Cattle	Northeastern	0	0	0	0	0	0
2170	Cattle	Northeastern	0	0	0	0	0	0
2171	Cattle	Northeastern	0	0	0	0	0	0
2172	Cattle	Northeastern	0	0	0	0	0	0
2173	Cattle	Northeastern	0	0	0	0	0	0
2174	Cattle	Northeastern	0	0	0	0	0	0
2175	Cattle	Northeastern	0	1	0	0	0	1
2176	Cattle	Northeastern	0	0	0	1	0	1
2177	Cattle	Northeastern	0	0	0	0	0	0
2178	Cattle	Northeastern	0	0	0	0	0	0
2179	Cattle	Northeastern	0	0	0	0	0	0
2180	Cattle	Northeastern	0	0	0	0	0	0
2181	Cattle	Northeastern	0	0	0	0	0	0
2182	Cattle	Northeastern	0	0	0	0	0	0
2183	Cattle	Northeastern	0	0	0	0	0	0
2184	Cattle	Northeastern	0	0	0	0	0	0
2185	Cattle	Northeastern	0	0	0	0	0	0
2186	Cattle	Northeastern	0	0	0	0	0	0
2187	Cattle	Northeastern	0	0	0	0	0	0
2188	Cattle	Northeastern	0	0	0	0	0	0
2189	Cattle	Northeastern	0	0	0	0	0	0
2190	Cattle	Northeastern	0	0	0	0	0	0
2191	Cattle	Northeastern	0	0	0	0	0	0
2192	Cattle	Northeastern	0	0	0	0	0	0
2193	Cattle	Northeastern	0	0	0	0	0	0
2194	Cattle	Northeastern	0	0	0	0	0	0
2195	Cattle	Northeastern	0	0	0	0	0	0
2196	Cattle	Northeastern	0	0	0	0	0	0
2197	Cattle	Northeastern	0	0	0	0	0	0
2198	Cattle	Northeastern	0	0	0	0	0	0
2199	Cattle	Northeastern	0	0	0	0	0	0
2200	Cattle	Northeastern	0	0	0	0	0	0
2201	Cattle	Northeastern	0	0	0	0	0	0
2202	Cattle	Central	0	0	0	0	0	0
2203	Cattle	Central	0	0	0	0	0	0
2204	Cattle	Central	0	0	0	0	0	0
2205	Cattle	Central	0	0	0	0	0	0
2206	Cattle	Central	0	0	0	0	0	0
2207	Cattle	Central	0	0	0	0	0	0

2208	Cattle	Central	0	0	0	0	0	0
2209	Cattle	Central	0	0	0	0	0	0
2210	Cattle	Central	0	0	0	0	0	0
2211	Cattle	Central	0	0	0	0	0	0
2212	Cattle	Central	0	0	0	0	0	0
2213	Cattle	Central	0	0	0	0	0	0
2214	Cattle	Central	0	0	0	0	0	0
2215	Cattle	Central	0	0	0	0	0	0
2216	Cattle	Central	0	0	0	0	0	0
2217	Cattle	Central	0	0	0	0	0	0
2218	Cattle	Central	0	0	0	0	0	0
2219	Cattle	Central	0	0	0	0	0	0
2220	Cattle	Central	0	0	0	0	0	0
2221	Cattle	Central	0	0	0	0	0	0
2222	Cattle	Central	0	0	0	0	0	0
2223	Cattle	Central	0	0	0	0	0	0
2224	Cattle	Central	0	0	0	0	0	0
2225	Cattle	Central	0	0	0	0	0	0
2226	Cattle	Central	0	0	0	0	0	0
2227	Cattle	Central	0	0	0	0	0	0
2228	Cattle	Central	0	0	0	0	0	0
2229	Cattle	Central	0	0	0	0	0	0
2230	Cattle	Central	0	0	0	0	0	0
2231	Cattle	Central	0	0	0	0	0	0
2232	Cattle	Central	0	0	0	0	0	0
2233	Cattle	Central	0	0	0	0	1	1
2234	Cattle	Central	0	0	0	0	0	0
2235	Cattle	Central	0	0	0	0	0	0
2236	Cattle	Central	0	0	0	0	0	0
2237	Cattle	Central	0	0	0	0	0	0
2238	Cattle	Central	0	0	0	0	0	0
2239	Cattle	Central	0	0	0	0	0	0
2240	Cattle	Central	0	0	0	0	0	0
2241	Cattle	Central	0	0	0	0	0	0
2242	Cattle	Central	0	0	0	0	0	0
2243	Cattle	Central	0	0	0	0	0	0
2244	Cattle	Central	0	0	0	0	0	0
2245	Cattle	Central	0	0	0	0	0	0
2246	Cattle	Central	0	0	0	0	0	0
2247	Cattle	Central	0	0	0	0	0	0
2248	Cattle	Central	0	0	0	0	0	0
2249	Cattle	Central	0	0	0	0	0	0
2250	Cattle	Central	0	0	0	0	0	0
2251	Cattle	Central	0	0	0	0	0	0
2252	Cattle	Central	0	0	0	0	0	0
2253	Cattle	Central	0	0	0	0	0	0
2254	Cattle	Central	0	0	0	0	0	0
2255	Cattle	Central	0	0	0	0	0	0

2256	Cattle	Central	0	0	0	0	0	0
2257	Cattle	Central	0	0	0	0	0	0
2258	Cattle	Central	0	0	0	0	0	0
2259	Cattle	Central	0	0	0	0	0	0
2260	Cattle	Central	0	0	0	0	0	0
2261	Cattle	Central	0	0	0	0	0	0
2262	Cattle	Central	0	0	0	0	0	0
2263	Cattle	Central	0	0	0	0	0	0
2264	Cattle	Central	0	0	0	0	0	0
2265	Cattle	Central	0	0	0	0	0	0
2266	Cattle	Central	0	0	0	0	0	0
2267	Cattle	Central	0	0	0	0	0	0
2268	Cattle	Central	0	0	0	0	0	0
2269	Cattle	Central	0	0	0	0	0	0
2270	Cattle	Central	0	0	0	0	0	0
2271	Cattle	Central	0	0	0	0	0	0
2272	Cattle	Central	0	0	0	0	0	0
2273	Cattle	Central	0	0	0	0	0	0
2274	Cattle	Central	0	0	0	0	0	0
2275	Cattle	Central	0	0	0	0	0	0
2276	Cattle	Central	0	0	0	1	0	1
2277	Cattle	Central	0	0	0	1	1	1
2278	Cattle	Central	0	0	0	0	0	0
2279	Cattle	Central	0	0	0	0	0	0
2280	Cattle	Central	0	0	0	0	0	0
2281	Cattle	Central	0	0	0	0	0	0
2282	Cattle	Central	0	0	0	0	0	0
2283	Cattle	Central	0	0	0	0	0	0
2284	Cattle	Central	0	0	0	0	0	0
2285	Cattle	Central	0	0	0	0	0	0
2286	Cattle	Central	0	0	0	0	0	0
2287	Cattle	Central	0	0	0	0	0	0
2288	Cattle	Central	0	0	0	0	0	0
2289	Cattle	Central	0	0	0	0	0	0
2290	Cattle	Central	0	0	0	0	0	0
2291	Cattle	Central	0	0	0	0	0	0
2292	Cattle	Central	0	0	0	0	0	0
2293	Cattle	Central	0	0	0	0	0	0
2294	Cattle	Central	0	0	0	0	0	0
2295	Cattle	Central	0	0	0	0	0	0
2296	Cattle	Central	0	0	0	0	0	0
2297	Cattle	Central	0	0	0	0	0	0
2298	Cattle	Central	0	0	0	0	1	1
2299	Cattle	Central	0	0	0	0	0	0
2300	Cattle	Central	0	0	0	0	0	0
2301	Cattle	Central	0	0	0	0	0	0
2302	Cattle	Central	0	0	0	0	0	0
2303	Cattle	Central	0	0	0	1	0	1

2304	Cattle	Central	0	0	0	0	0	0
2305	Cattle	Central	0	0	0	0	0	0
2306	Cattle	Central	0	0	1	1	0	1
2307	Cattle	Central	0	0	0	1	0	1
2308	Cattle	Central	0	0	0	0	0	0
2309	Cattle	Central	0	0	0	0	0	0
2310	Cattle	Central	0	0	0	0	0	0
2311	Cattle	Central	0	0	0	0	0	0
2312	Cattle	Central	0	0	0	1	0	1
2313	Cattle	Central	0	0	0	0	0	0
2314	Cattle	Central	0	0	1	0	0	1
2315	Cattle	Central	0	0	1	1	0	1
2316	Cattle	Central	0	0	1	1	0	1
2317	Cattle	Central	0	0	1	0	0	1
2318	Cattle	Central	0	0	1	0	0	1
2319	Cattle	Central	0	0	1	0	0	1
2320	Cattle	Central	0	0	0	0	0	0
2321	Cattle	Central	0	0	0	0	0	0
2322	Cattle	Central	0	0	1	0	0	1
2323	Cattle	Central	0	0	0	0	0	0
2324	Cattle	Central	0	0	0	0	0	0
2325	Cattle	Central	0	0	0	0	0	0
2326	Cattle	Central	0	0	0	0	0	0
2327	Cattle	Central	0	0	0	0	0	0
2328	Cattle	Central	0	0	0	0	0	0
2329	Cattle	Central	0	0	0	0	0	0
2330	Cattle	Central	0	0	0	0	0	0
2331	Cattle	Central	0	0	0	0	0	0
2332	Cattle	Central	0	0	0	0	0	0
2333	Cattle	Southern	0	0	0	1	0	1
2334	Cattle	Southern	0	0	0	0	0	0
2335	Cattle	Southern	0	0	1	1	1	1
2336	Cattle	Southern	0	0	1	0	0	1
2337	Cattle	Southern	0	0	0	0	0	0
2338	Cattle	Southern	0	0	0	0	0	0
2339	Cattle	Southern	0	0	0	0	0	0
2340	Cattle	Southern	0	0	0	0	0	0
2341	Cattle	Southern	0	0	0	0	0	0
2342	Cattle	Southern	0	0	0	0	0	0
2343	Cattle	Southern	0	0	0	0	0	0
2344	Cattle	Southern	0	0	0	0	0	0
2345	Cattle	Southern	0	0	0	0	0	0
2346	Cattle	Northeastern	0	0	0	0	0	0
2347	Cattle	Northeastern	0	0	0	0	0	0
2348	Cattle	Northeastern	0	0	0	0	0	0
2349	Cattle	Northeastern	0	0	0	0	0	0
2350	Cattle	Northeastern	0	0	0	0	0	0
2351	Cattle	Northeastern	0	0	0	1	0	1

2352	Cattle	Northeastern	0	0	0	1	0	1
2353	Cattle	Northeastern	1	0	0	0	0	1
2354	Cattle	Northeastern	0	0	0	0	0	0
2355	Cattle	Northeastern	0	0	0	0	1	1
2356	Cattle	Northeastern	0	0	0	0	1	1
2357	Cattle	Northeastern	0	0	0	0	1	1
2358	Cattle	Northeastern	0	0	0	0	0	0
2359	Cattle	Northeastern	0	0	0	0	0	0
2360	Cattle	Northeastern	0	0	0	0	0	0
2361	Cattle	Northeastern	0	0	0	0	0	0
2362	Cattle	Northeastern	0	0	0	0	0	0
2363	Cattle	Northeastern	0	0	0	0	0	0
2364	Cattle	Northeastern	0	0	0	0	0	0
2365	Cattle	Central	0	0	0	0	0	0
2366	Cattle	Central	0	0	0	0	0	0
2367	Cattle	Central	0	0	0	0	0	0
2368	Cattle	Central	0	0	0	0	0	0
2369	Cattle	Central	0	0	0	0	0	0
2370	Cattle	Central	0	0	0	0	0	0
2371	Cattle	Central	0	0	0	1	0	1
2372	Cattle	Central	0	0	0	0	0	0
2373	Cattle	Central	0	0	0	0	0	0
2374	Cattle	Central	0	0	0	0	0	0
2375	Cattle	Central	0	0	0	1	0	1
2376	Cattle	Central	0	0	0	0	0	0
2377	Cattle	Central	0	0	0	0	0	0
2378	Cattle	Central	0	0	0	0	0	0
2379	Cattle	Central	0	0	0	0	0	0
2380	Cattle	Central	0	0	0	0	0	0
2381	Cattle	Central	0	0	0	0	0	0
2382	Cattle	Central	0	0	0	0	0	0
2383	Cattle	Central	0	0	0	0	0	0
2384	Cattle	Central	0	0	0	0	0	0
2385	Cattle	Central	0	0	0	0	0	0
2386	Cattle	Central	0	0	0	0	0	0
2387	Cattle	Central	0	0	0	0	0	0
2388	Cattle	Central	0	0	0	0	0	0
2389	Cattle	Central	0	0	0	0	0	0
2390	Cattle	Central	0	0	0	0	0	0
2391	Cattle	Central	0	0	0	0	0	0
2392	Cattle	Central	0	0	0	0	0	0
2393	Cattle	Central	0	0	0	0	0	0
2394	Cattle	Central	0	0	0	0	0	0
2395	Cattle	Central	0	0	0	0	0	0
2396	Cattle	Central	0	0	0	0	0	0
2397	Cattle	Central	0	0	0	0	0	0
2398	Cattle	Central	0	0	0	0	0	0
2399	Cattle	Central	0	0	0	0	0	0

2400	Cattle	Central	0	0	0	0	0	0
2401	Cattle	Central	0	0	0	0	0	0
2402	Cattle	Central	0	0	0	0	0	0
2403	Cattle	Central	0	0	0	0	0	0
2404	Cattle	Central	0	0	0	0	0	0
2405	Cattle	Central	0	0	0	0	0	0
2406	Cattle	Central	0	0	0	0	0	0
2407	Cattle	Central	0	0	0	0	0	0
2408	Cattle	Central	0	0	0	0	0	0
2409	Cattle	Central	0	0	0	0	0	0
2410	Cattle	Central	0	0	0	0	0	0
2411	Cattle	Central	0	0	0	0	0	0
2412	Cattle	Central	0	0	0	0	0	0
2413	Cattle	Central	0	0	0	0	0	0
2414	Cattle	Central	0	0	0	0	0	0
2415	Cattle	Central	0	0	0	0	0	0
2416	Cattle	Central	0	0	0	0	0	0
2417	Cattle	Central	0	0	0	0	0	0
2418	Cattle	Central	1	0	0	0	0	1
2419	Cattle	Central	0	0	0	0	0	0
2420	Cattle	Central	0	0	0	1	0	1
2421	Cattle	Central	0	0	0	0	0	0
2422	Cattle	Central	0	0	0	0	0	0
2423	Cattle	Central	0	0	0	0	0	0
2424	Cattle	Central	0	0	0	0	0	0
2425	Cattle	Central	0	0	0	0	0	0
2426	Cattle	Central	0	0	0	0	0	0
2427	Cattle	Central	0	0	0	0	0	0
2428	Cattle	Central	0	0	0	0	0	0
2429	Cattle	Central	0	0	0	0	0	0
2430	Cattle	Central	0	0	0	0	0	0
2431	Cattle	Central	0	0	0	0	0	0
2432	Cattle	Central	0	0	0	0	0	0
2433	Cattle	Central	0	0	0	1	0	1
2434	Cattle	Central	0	0	0	0	0	0
2435	Cattle	Central	0	0	0	0	0	0
2436	Cattle	Central	0	0	0	0	0	0
2437	Cattle	Central	0	0	0	0	0	0
2438	Cattle	Central	0	0	0	0	0	0
2439	Cattle	Central	0	0	0	0	0	0
2440	Cattle	Central	0	0	1	0	0	1
2441	Cattle	Central	0	0	0	0	0	0
2442	Cattle	Central	0	0	1	1	0	1
2443	Cattle	Central	0	0	0	1	0	1
2444	Cattle	Central	0	0	0	0	0	0
2445	Cattle	Central	0	0	0	0	0	0
2446	Cattle	Central	0	0	0	0	0	0
2447	Cattle	Central	0	0	0	0	0	0

2448	Cattle	Central	0	0	0	0	0	0
2449	Cattle	Central	0	0	1	0	0	1
2450	Cattle	Central	0	0	1	0	0	1
2451	Cattle	Central	0	0	1	0	0	1
2452	Cattle	Central	0	0	1	0	0	1
2453	Cattle	Central	0	0	1	0	0	1
2454	Cattle	Central	0	0	0	0	0	0
2455	Cattle	Central	0	0	0	0	0	0
2456	Cattle	Central	0	0	0	0	0	0
2457	Cattle	Central	0	0	1	1	0	1
2458	Cattle	Central	0	0	1	1	0	1
2459	Cattle	Central	0	0	0	0	0	0
2460	Cattle	Central	0	0	0	0	0	0
2461	Cattle	Central	0	0	1	0	0	1
2462	Cattle	Central	0	0	0	0	0	0
2463	Cattle	Central	0	0	0	0	0	0
2464	Cattle	Central	0	0	0	0	0	0
2465	Cattle	Central	0	0	0	0	0	0
2466	Cattle	Central	0	0	1	1	0	1
2467	Cattle	Central	0	0	1	0	0	1
2468	Cattle	Central	0	0	0	0	0	0
2469	Cattle	Central	0	0	1	0	0	1
2470	Cattle	Central	0	0	1	0	0	1
2471	Cattle	Central	0	0	0	0	0	0
2472	Cattle	Central	0	0	0	0	0	0
2473	Cattle	Central	0	0	1	0	0	1
2474	Cattle	Central	0	0	0	0	0	0
2475	Cattle	Central	0	0	1	0	0	1
2476	Cattle	Central	0	0	1	0	0	1
2477	Cattle	Central	0	0	0	0	0	0
2478	Cattle	Central	0	0	0	0	0	0
2479	Cattle	Central	0	0	1	1	0	1
2480	Cattle	Central	0	0	0	0	0	0
2481	Cattle	Central	0	0	0	0	0	0
2482	Cattle	Central	0	0	0	0	0	0
2483	Cattle	Central	0	0	0	0	0	0
2484	Cattle	Central	0	0	1	0	0	1
2485	Cattle	Central	0	0	1	0	0	1
2486	Cattle	Central	0	0	0	0	0	0
2487	Cattle	Central	0	1	0	1	0	1
2488	Cattle	Central	0	0	0	0	0	0
2489	Cattle	Central	0	0	1	1	1	1
2490	Cattle	Central	0	0	0	0	0	0
2491	Cattle	Central	0	0	0	0	0	0
2492	Cattle	Central	0	0	0	1	0	1
2493	Cattle	Central	0	0	1	1	0	1
2494	Cattle	Central	0	0	0	1	0	1
2495	Cattle	Central	0	0	0	1	0	1

2496	Cattle	Central	0	0	1	1	0	1
2497	Cattle	Central	0	0	1	0	0	1
2498	Cattle	Central	0	0	0	0	0	0
2499	Cattle	Central	0	0	0	1	1	1
2500	Cattle	Central	0	0	1	1	0	1
2501	Cattle	Central	0	0	0	0	0	0
2502	Cattle	Central	0	0	0	0	0	0
2503	Cattle	Central	0	0	0	0	0	0
2504	Cattle	Central	0	0	0	0	0	0
2505	Cattle	Central	0	0	0	0	0	0
2506	Cattle	Central	0	0	0	0	0	0
2507	Cattle	Central	0	0	0	0	0	0
2508	Cattle	Central	0	0	0	1	0	1
2509	Cattle	Central	0	0	0	1	0	1
2510	Cattle	Central	0	0	0	1	0	1
2511	Cattle	Central	0	0	0	0	0	0
2512	Cattle	Central	0	0	0	0	0	0
2513	Cattle	Central	0	0	0	0	0	0
2514	Cattle	Central	0	0	0	1	0	1
2515	Cattle	Central	0	0	0	0	0	1
2516	Cattle	Central	0	0	0	0	0	0
2517	Cattle	Central	0	0	0	0	0	0
2518	Cattle	Central	0	0	0	0	0	0
2519	Cattle	Central	0	0	0	0	0	0
2520	Cattle	Central	0	0	0	0	0	0
2521	Cattle	Central	0	0	0	0	0	0
2522	Cattle	Central	0	0	0	0	0	0
2523	Cattle	Central	0	0	0	0	0	0
2524	Cattle	Central	0	0	0	0	0	0
2525	Cattle	Central	0	0	0	0	0	0
2526	Cattle	Central	0	0	0	0	0	0
2527	Cattle	Central	0	0	1	0	0	1
2528	Cattle	Central	0	0	0	0	0	0
2529	Cattle	Central	0	0	0	0	0	0
2530	Cattle	Central	0	0	0	0	0	0
2531	Cattle	Central	0	0	0	0	0	0
2532	Cattle	Central	0	0	0	0	0	0
2533	Cattle	Central	0	0	1	0	0	1
2534	Cattle	Central	0	0	0	0	0	0
2535	Cattle	Central	0	0	0	0	0	0
2536	Cattle	Central	0	0	0	0	0	0
2537	Cattle	Central	0	0	0	1	0	1
2538	Cattle	Central	0	0	1	1	0	1
2539	Cattle	Central	0	0	1	1	0	1
2540	Cattle	Central	0	0	0	0	0	0
2541	Cattle	Central	0	0	0	0	0	0
2542	Cattle	Central	0	0	0	0	0	0
2543	Cattle	Central	0	0	0	1	0	1

2544	Cattle	Central	0	0	0	0	0	0
2545	Cattle	Central	0	0	0	0	0	0
2546	Cattle	Central	0	0	0	0	0	0
2547	Cattle	Central	0	0	0	0	0	0
2548	Cattle	Central	0	0	0	0	0	0
2549	Cattle	Central	0	0	0	0	0	0
2550	Cattle	Central	0	0	0	0	0	0
2551	Cattle	Central	0	0	0	0	0	0
2552	Cattle	Central	0	0	0	0	0	0
2553	Cattle	Central	0	0	0	0	0	0
2554	Cattle	Central	0	0	0	0	0	0
2555	Cattle	Central	0	0	0	0	0	0
2556	Cattle	Central	0	0	0	0	0	0
2557	Cattle	Central	0	0	0	0	0	0
2558	Cattle	Central	0	0	0	0	0	0
2559	Cattle	Central	0	0	0	0	0	0
2560	Cattle	Central	0	0	0	0	0	0
2561	Cattle	Central	0	0	0	0	0	0
2562	Cattle	Central	1	0	1	1	0	1
2563	Cattle	Central	0	0	0	0	0	0
2564	Cattle	Central	0	0	0	1	0	1
2565	Cattle	Central	0	0	0	1	0	1
2566	Cattle	Central	1	0	1	1	0	1
2567	Cattle	Central	0	0	0	1	0	1
2568	Cattle	Central	0	0	0	1	0	1
2569	Cattle	Central	0	0	0	0	0	0
2570	Cattle	Central	0	0	0	0	0	0
2571	Cattle	Central	0	0	0	0	0	0
2572	Cattle	Central	1	0	1	1	0	1
2573	Cattle	Central	0	0	1	0	0	1
2574	Cattle	Central	0	0	0	0	0	0
2575	Cattle	Central	0	0	0	0	0	0
2576	Cattle	Central	0	0	0	0	0	0
2577	Cattle	Central	0	0	0	0	0	0
2578	Cattle	Central	0	0	0	0	0	0
2579	Cattle	Central	0	0	1	0	0	1
2580	Cattle	Central	0	0	0	0	0	0
2581	Cattle	Central	0	0	0	0	0	0
2582	Cattle	Central	0	0	0	0	0	0
2583	Cattle	Central	0	0	0	0	0	0
2584	Cattle	Central	0	0	0	0	0	0
2585	Cattle	Central	0	0	0	0	0	0
2586	Cattle	Central	0	0	0	0	0	0
2587	Cattle	Central	0	0	0	0	0	0
2588	Cattle	Central	0	0	1	0	0	1
2589	Cattle	Central	0	0	1	0	0	1
2590	Cattle	Central	0	0	1	0	0	1
2591	Cattle	Central	0	0	0	0	0	0

2592	Cattle	Central	0	0	1	0	0	1
2593	Cattle	Central	0	0	1	0	0	1
2594	Cattle	Central	0	0	0	0	0	0
2595	Cattle	Central	0	0	0	0	0	0
2596	Cattle	Central	0	0	0	0	0	0
2597	Cattle	Central	0	0	0	0	0	0
2598	Cattle	Central	0	0	0	0	0	0
2599	Cattle	Central	0	0	0	0	0	0
2600	Cattle	Central	0	0	0	0	0	0
2601	Cattle	Central	0	0	0	0	0	0
2602	Cattle	Central	0	0	0	0	0	0
2603	Cattle	Central	0	0	0	0	0	0
2604	Cattle	Central	0	0	0	0	0	0
2605	Cattle	Central	0	0	0	0	0	0
2606	Cattle	Central	0	0	0	0	0	0
2607	Cattle	Central	0	0	0	0	0	0
2608	Cattle	Central	0	0	1	0	0	1
2609	Cattle	Central	0	0	1	0	0	1
2610	Cattle	Central	0	0	0	0	0	0
2611	Cattle	Central	0	0	0	1	0	1
2612	Cattle	Central	0	0	0	1	0	1
2613	Cattle	Central	0	0	0	1	0	1
2614	Cattle	Central	0	0	1	1	0	1
2615	Cattle	Central	0	0	1	1	0	1
2616	Cattle	Central	0	0	0	1	0	1
2617	Cattle	Central	0	0	0	0	0	0
2618	Cattle	Central	0	0	0	1	0	1
2619	Cattle	Central	0	0	1	1	0	1
2620	Cattle	Central	0	0	1	1	0	1
2621	Cattle	Central	0	0	0	1	1	1
2622	Cattle	Central	0	0	1	1	0	1
2623	Cattle	Central	0	0	0	1	1	1
2624	Cattle	Central	0	0	0	1	0	1
2625	Cattle	Central	0	0	1	1	0	1
2626	Cattle	Central	0	0	0	1	0	1
2627	Cattle	Central	0	0	0	1	0	1
2628	Cattle	Central	0	0	0	1	0	1
2629	Cattle	Central	0	0	0	1	0	1
2630	Cattle	Central	0	0	0	1	0	1
2631	Cattle	Central	0	0	0	1	0	1
2632	Cattle	Central	0	0	1	1	1	1
2633	Cattle	Central	0	0	0	1	0	1
2634	Cattle	Central	0	0	1	1	0	1
2635	Cattle	Central	0	0	0	1	1	1
2636	Cattle	Central	0	0	0	1	1	1
2637	Cattle	Central	0	0	0	1	0	1
2638	Cattle	Central	0	0	1	1	1	1
2639	Cattle	Central	0	0	1	1	0	1

2640	Cattle	Central	0	0	1	1	0	1
2641	Cattle	Central	0	0	0	1	0	1
2642	Cattle	Central	0	0	0	1	0	1
2643	Cattle	Central	0	0	0	1	0	1
2644	Cattle	Central	0	0	0	1	0	1
2645	Cattle	Central	0	0	0	1	0	1
2646	Cattle	Central	0	0	0	1	0	1
2647	Cattle	Central	0	0	0	1	0	1
2648	Cattle	Central	0	0	0	1	0	1
2649	Cattle	Central	0	0	0	1	0	1
2650	Cattle	Central	0	0	1	1	0	1
2651	Cattle	Central	0	0	1	1	0	1
2652	Cattle	Central	0	0	0	1	0	1
2653	Cattle	Central	0	0	0	1	0	1
2654	Cattle	Central	0	0	0	1	0	1
2655	Cattle	Central	0	0	1	1	0	1
2656	Cattle	Central	0	0	0	1	0	1
2657	Cattle	Central	0	0	0	1	0	1
2658	Cattle	Central	0	0	0	1	0	1
2659	Cattle	Central	0	0	0	0	0	0
2660	Cattle	Central	0	0	0	1	0	1
2661	Cattle	Central	0	0	0	0	0	0
2662	Cattle	Central	0	0	0	0	0	0
2663	Cattle	Central	0	0	0	0	0	0
2664	Cattle	Central	0	0	0	0	0	0
2665	Cattle	Central	0	0	0	0	0	0
2666	Cattle	Central	0	0	0	0	0	0
2667	Cattle	Central	0	0	0	0	0	0
2668	Cattle	Central	0	0	0	0	0	0
2669	Cattle	Central	0	0	0	0	0	0
2670	Cattle	Central	0	0	0	0	0	0
2671	Cattle	Central	0	0	0	1	0	1
2672	Cattle	Central	0	0	0	0	0	0
2673	Cattle	Central	0	0	0	1	0	1
2674	Cattle	Central	0	0	0	0	0	0
2675	Cattle	Central	0	0	0	1	0	1
2676	Cattle	Central	0	0	0	1	0	1
2677	Cattle	Central	0	0	0	0	0	0
2678	Cattle	Central	0	0	0	1	1	1
2679	Cattle	Central	0	0	0	1	0	1
2680	Cattle	Central	0	0	0	1	0	1
2681	Cattle	Central	0	0	0	0	0	0
2682	Cattle	Central	0	0	0	1	0	1
2683	Cattle	Central	0	0	0	1	0	1
2684	Cattle	Central	0	0	0	1	0	1
2685	Cattle	Central	0	0	0	1	0	1
2686	Cattle	Central	0	0	1	1	0	1
2687	Cattle	Central	0	0	0	1	0	1

2688	Cattle	Central	0	0	0	1	0	1
2689	Cattle	Central	0	0	1	1	0	1
2690	Cattle	Central	0	0	0	1	0	1
2691	Cattle	Central	0	1	0	1	0	1
2692	Cattle	Central	0	0	0	1	0	1
2693	Cattle	Central	0	0	0	1	0	1
2694	Cattle	Central	0	0	1	1	0	1
2695	Cattle	Central	0	0	0	1	0	1
2696	Cattle	Central	0	0	0	0	0	0
2697	Cattle	Central	0	0	0	1	0	1
2698	Cattle	Central	0	0	0	1	0	1
2699	Cattle	Central	0	0	0	1	0	1
2700	Cattle	Central	0	0	0	0	0	0
2701	Cattle	Central	0	0	1	1	0	1
2702	Cattle	Central	0	0	0	1	0	1
2703	Cattle	Central	0	0	0	0	0	0
2704	Cattle	Central	0	0	0	0	0	0
2705	Cattle	Central	0	0	0	0	0	0
2706	Cattle	Central	0	0	0	1	0	1
2707	Cattle	Central	0	0	0	0	0	0
2708	Cattle	Central	0	0	0	0	0	0
2709	Cattle	Central	0	0	0	0	0	0
2710	Cattle	Central	0	0	0	0	0	0
2711	Cattle	Central	0	0	1	1	0	1
2712	Cattle	Central	0	0	0	1	0	1
2713	Cattle	Central	0	0	0	1	0	1
2714	Cattle	Central	0	0	0	1	0	1
2715	Cattle	Central	0	0	0	1	0	1
2716	Cattle	Central	0	0	0	0	0	0
2717	Cattle	Central	0	0	0	1	0	1
2718	Cattle	Central	0	0	0	0	0	0
2719	Cattle	Central	0	0	0	0	0	0
2720	Cattle	Central	0	0	0	0	0	0
2721	Cattle	Central	0	0	0	1	0	1
2722	Cattle	Central	0	0	0	1	0	1
2723	Cattle	Central	0	0	0	1	0	1
2724	Cattle	Northeastern	0	0	1	1	0	1
2725	Cattle	Northeastern	0	0	0	1	0	1
2726	Cattle	Northeastern	0	0	0	1	0	1
2727	Cattle	Northeastern	0	0	0	0	0	0
2728	Cattle	Northeastern	0	0	0	0	0	0
2729	Cattle	Northeastern	0	0	0	0	0	0
2730	Cattle	Eastern	0	0	0	0	0	0
2731	Cattle	Eastern	0	0	0	0	0	1
2732	Cattle	Eastern	0	0	0	1	0	1
2733	Cattle	Eastern	0	0	0	0	0	0
2734	Cattle	Eastern	0	0	0	0	0	1
2735	Cattle	Central	0	0	0	1	0	1

2736	Cattle	Central	0	0	0	0	0	0
2737	Cattle	Central	0	0	0	1	0	1
2738	Cattle	Central	0	1	0	1	0	1
2739	Cattle	Central	0	0	0	0	0	0
2740	Cattle	Central	0	0	0	1	0	1
2741	Cattle	Central	0	1	0	0	0	1
2742	Cattle	Central	0	0	0	0	0	0
2743	Cattle	Central	0	0	0	1	0	1
2744	Cattle	Central	0	0	0	0	0	0
2745	Cattle	Central	0	0	0	0	0	0
2746	Cattle	Central	0	0	0	0	0	0
2747	Cattle	Central	0	0	0	0	0	0
2748	Cattle	Central	0	0	0	1	0	1
2749	Cattle	Central	0	0	0	1	0	1
2750	Cattle	Central	0	0	0	1	0	1
2751	Cattle	Central	0	1	0	0	0	1
2752	Cattle	Central	0	0	0	0	0	0
2753	Cattle	Central	0	0	0	0	0	0
2754	Cattle	Central	0	0	0	0	0	0
2755	Cattle	Central	0	0	0	0	0	0
2756	Cattle	Central	0	0	0	0	0	0
2757	Cattle	Central	0	0	0	0	0	0
2758	Cattle	Central	0	0	0	0	0	0
2759	Cattle	Central	0	0	0	0	0	0
2760	Cattle	Central	0	1	0	1	0	1
2761	Cattle	Central	0	0	0	0	0	0
2762	Cattle	Central	0	0	0	0	0	0
2763	Cattle	Central	0	0	0	0	0	0
2764	Cattle	Central	0	0	0	1	0	1
2765	Cattle	Central	0	0	0	0	0	0
2766	Cattle	Central	0	0	0	0	0	0
2767	Cattle	Central	0	0	0	0	0	0
2768	Cattle	Central	0	0	0	0	0	0
2769	Cattle	Central	0	0	0	0	0	0
2770	Cattle	Central	0	0	0	0	0	0
2771	Cattle	Central	0	0	0	0	0	0
2772	Cattle	Central	0	0	0	0	0	0
2773	Cattle	Southern	0	0	0	0	0	0
2774	Cattle	Southern	0	0	0	1	0	1
2775	Cattle	Southern	0	0	0	0	0	0
2776	Cattle	Southern	0	0	0	0	0	0
2777	Cattle	Southern	0	0	0	0	0	0
2778	Cattle	Southern	0	0	0	0	0	0
2779	Cattle	Southern	1	0	0	1	0	1
2780	Cattle	Southern	0	0	0	1	0	1
2781	Cattle	Southern	0	0	0	1	0	1
2782	Cattle	Southern	0	0	0	1	0	1
2783	Cattle	Southern	0	0	0	0	0	0

2784	Cattle	Southern	0	0	0	0	0	0
2785	Cattle	Southern	0	0	0	1	0	1
2786	Cattle	Southern	0	0	0	0	0	0
2787	Cattle	Southern	0	0	0	0	0	0
2788	Cattle	Southern	0	0	0	1	0	1
2789	Cattle	Southern	0	0	0	0	0	0
2790	Cattle	Southern	0	0	0	0	0	0
2791	Cattle	Southern	0	0	0	1	0	1
2792	Cattle	Southern	0	0	0	0	0	0
2793	Cattle	Southern	0	0	0	1	0	1
2794	Cattle	Southern	0	0	0	0	0	0
2795	Cattle	Southern	0	0	0	0	0	0
2796	Cattle	Southern	0	0	0	1	0	1
2797	Cattle	Southern	0	0	0	0	0	1
2798	Cattle	Southern	0	0	0	1	0	1
2799	Cattle	Southern	0	0	0	0	0	0
2800	Cattle	Southern	0	0	0	0	0	0
2801	Cattle	Southern	0	0	0	0	0	0
2802	Cattle	Southern	0	0	0	0	0	0
2803	Cattle	Central	0	0	0	1	0	1
2804	Cattle	Central	0	0	0	0	0	0
2805	Cattle	Central	0	0	0	1	0	1
2806	Cattle	Central	0	0	0	1	0	1
2807	Cattle	Central	0	0	0	1	0	1
2808	Cattle	Central	0	0	0	1	0	1
2809	Cattle	Central	0	0	0	1	1	1
2810	Cattle	Central	0	0	0	0	0	0
2811	Cattle	Central	0	0	0	1	0	1
2812	Cattle	Central	0	0	0	1	0	1
2813	Cattle	Central	0	0	0	1	0	1
2814	Cattle	Central	0	0	0	1	0	1
2815	Cattle	Central	0	0	0	1	0	1
2816	Cattle	Central	0	0	0	1	0	1
2817	Cattle	Central	0	0	0	1	1	1
2818	Cattle	Central	0	0	0	1	1	1
2819	Cattle	Central	0	0	0	1	1	1
2820	Cattle	Central	0	0	0	1	1	1
2821	Cattle	Central	0	0	1	1	1	1
2822	Cattle	Central	0	0	1	1	1	1
2823	Cattle	Central	0	0	1	1	1	1
2824	Cattle	Central	0	0	0	0	1	1
2825	Cattle	Central	0	0	1	1	0	1
2826	Cattle	Central	0	0	1	1	0	1
2827	Cattle	Central	0	0	1	1	0	1
2828	Cattle	Central	0	0	1	1	0	1
2829	Cattle	Central	0	0	1	1	0	1
2830	Cattle	Central	0	0	1	1	1	1
2831	Cattle	Central	0	0	1	1	0	1

2832	Cattle	Central	0	0	1	1	0	1
2833	Cattle	Central	0	0	1	1	0	1
2834	Cattle	Central	0	0	1	1	1	1
2835	Cattle	Central	0	1	1	1	0	1
2836	Cattle	Central	0	0	1	1	0	1
2837	Cattle	Central	0	0	1	1	0	1
2838	Cattle	Central	0	0	1	1	0	1
2839	Cattle	Central	0	0	1	1	0	1
2840	Cattle	Central	0	0	1	0	0	1
2841	Cattle	Central	0	0	0	1	0	1
2842	Cattle	Central	0	0	1	1	0	1
2843	Cattle	Central	0	0	1	1	0	1
2844	Cattle	Central	0	0	1	1	0	1
2845	Cattle	Southern	0	0	0	0	0	0
2846	Cattle	Southern	0	0	0	0	0	0
2847	Cattle	Northern	0	0	0	0	0	0
2848	Cattle	Northern	0	0	0	0	0	1
2849	Cattle	Northern	0	0	0	0	0	0
2850	Cattle	Northern	0	0	0	1	0	1
2851	Cattle	Northern	0	0	0	0	0	0
2852	Cattle	Northern	0	0	0	0	0	0
2853	Cattle	Northern	0	0	0	0	0	0
2854	Cattle	Northern	0	0	0	0	0	0
2855	Cattle	Northern	0	0	0	0	0	0
2856	Cattle	Northern	0	0	0	0	0	0
2857	Cattle	Northern	0	0	0	0	0	0
2858	Cattle	Northern	0	0	0	0	0	0
2859	Cattle	Northern	0	0	0	0	0	0
2860	Cattle	Northern	0	0	0	0	0	0
2861	Cattle	Northern	0	0	0	0	0	0
2862	Cattle	Northern	0	0	0	0	0	0
2863	Cattle	Northern	0	0	0	0	0	0
2864	Cattle	Northern	0	0	0	0	0	0
2865	Cattle	Northern	0	0	0	0	0	0
2866	Cattle	Northern	0	0	0	0	0	0
2867	Cattle	Northern	0	0	0	0	0	0
2868	Cattle	Northern	0	0	0	0	0	0
2869	Cattle	Northern	0	0	0	0	0	0
2870	Cattle	Northern	0	0	0	0	0	0
2871	Cattle	Northern	0	0	0	0	0	0
2872	Cattle	Northern	0	0	0	0	0	0
2873	Cattle	Northern	0	0	0	0	0	0
2874	Cattle	Northern	0	0	0	0	0	0
2875	Cattle	Northern	0	0	0	0	0	0
2876	Cattle	Northern	0	0	0	0	0	0
2877	Cattle	Northern	0	0	0	0	0	0
2878	Cattle	Northern	0	0	0	0	0	0
2879	Cattle	Northern	0	0	0	0	0	0

2880	Cattle	Northern	0	0	0	0	0	0
2881	Cattle	Northern	0	0	0	0	0	0
2882	Cattle	Northern	0	0	0	0	0	0
2883	Cattle	Northern	0	0	0	0	0	0
2884	Cattle	Northern	0	0	0	0	0	0
2885	Cattle	Northern	0	0	0	0	0	0
2886	Cattle	Northern	0	0	0	0	0	0
2887	Cattle	Northern	0	0	0	0	0	0
2888	Cattle	Northern	0	0	0	0	0	0
2889	Cattle	Northern	0	0	0	0	0	0
2890	Cattle	Northern	0	0	0	0	0	0
2891	Cattle	Northern	0	0	0	0	0	0
2892	Cattle	Northern	0	0	0	0	0	0
2893	Cattle	Northern	0	0	0	0	0	0
2894	Cattle	Northern	0	0	0	0	0	0
2895	Cattle	Northern	0	0	0	0	0	0
2896	Cattle	Northern	0	0	0	0	0	0
2897	Cattle	Northern	0	0	0	0	0	0
2898	Cattle	Northern	0	0	1	1	0	1
2899	Cattle	Northern	0	0	0	0	0	0
2900	Cattle	Northern	0	0	0	0	0	0
2901	Cattle	Northern	0	0	0	0	0	0
2902	Cattle	Northern	0	0	0	0	0	0
2903	Cattle	Northern	0	0	0	0	0	0
2904	Cattle	Northern	0	0	0	0	0	0
2905	Cattle	Northern	0	0	0	0	0	0
2906	Cattle	Northern	0	0	0	0	0	0
2907	Cattle	Northern	0	0	0	0	0	0
2908	Cattle	Northern	0	0	0	0	0	0
2909	Cattle	Northern	0	0	0	0	0	0
2910	Cattle	Northern	0	0	0	0	0	0
2911	Cattle	Northern	0	0	0	0	0	0
2912	Cattle	Northern	0	0	0	0	0	0
2913	Cattle	Northern	0	0	0	0	0	0
2914	Cattle	Northern	0	0	0	0	0	0
2915	Cattle	Northern	0	0	0	0	0	0
2916	Cattle	Northern	0	0	0	0	0	0
2917	Cattle	Northern	0	0	0	0	0	0
2918	Cattle	Northern	0	0	0	0	0	0
2919	Cattle	Northern	0	0	0	0	0	0
2920	Cattle	Northern	0	0	0	0	0	0
2921	Cattle	Northern	0	0	0	0	0	0
2922	Cattle	Northern	0	0	0	0	0	0
2923	Cattle	Northern	0	0	0	0	0	0
2924	Cattle	Northern	0	0	0	0	0	0
2925	Cattle	Northern	0	0	0	0	0	0
2926	Cattle	Northern	0	0	0	0	0	0
2927	Cattle	Northern	0	0	0	0	0	0

2928	Cattle	Northern	0	0	0	0	0	0
2929	Cattle	Northern	0	0	0	0	0	0
2930	Cattle	Northern	0	0	0	0	0	0
2931	Cattle	Northern	0	0	0	0	0	0
2932	Cattle	Northern	0	0	0	0	0	0
2933	Cattle	Northern	0	0	0	0	0	0
2934	Cattle	Northern	0	0	0	0	0	0
2935	Cattle	Northern	0	0	0	0	0	0
2936	Cattle	Northern	0	0	0	0	0	0
2937	Cattle	Northern	0	0	0	0	0	0
2938	Cattle	Northern	0	0	0	1	0	1
2939	Cattle	Northern	0	0	0	0	0	0
2940	Cattle	Northern	0	0	0	0	0	0
2941	Cattle	Northern	0	0	0	0	0	0
2942	Cattle	Northern	0	0	0	0	0	0
2943	Cattle	Northern	0	0	0	0	0	0
2944	Cattle	Northern	0	0	0	0	0	0
2945	Cattle	Northern	0	0	0	0	0	0
2946	Cattle	Northern	0	0	0	0	0	0
2947	Cattle	Northern	0	0	0	0	0	0
2948	Cattle	Northern	0	0	0	0	0	0
2949	Cattle	Northern	0	0	0	0	0	0
2950	Cattle	Northern	0	0	0	0	0	0
2951	Cattle	Northern	0	0	0	0	0	0
2952	Cattle	Northern	0	0	0	0	0	0
2953	Cattle	Northern	0	0	0	0	0	0
2954	Cattle	Northern	0	0	0	0	0	0
2955	Cattle	Northern	0	0	0	0	0	0
2956	Cattle	Northern	0	0	0	0	0	0
2957	Cattle	Northern	0	0	0	0	0	0
2958	Cattle	Northern	0	0	0	0	0	0
2959	Cattle	Northern	0	0	0	0	0	0
2960	Cattle	Northern	0	0	0	0	0	0
2961	Cattle	Northern	0	0	0	0	0	0
2962	Cattle	Northern	0	0	0	0	0	0
2963	Cattle	Northern	0	0	0	1	0	1
2964	Cattle	Northern	0	0	0	0	0	0
2965	Cattle	Northern	0	0	0	0	0	0
2966	Cattle	Northern	0	0	0	0	0	0
2967	Cattle	Northern	0	0	0	0	0	0
2968	Cattle	Northern	0	0	0	0	0	0
2969	Cattle	Northern	0	0	0	0	0	0
2970	Cattle	Northern	0	0	0	0	0	0
2971	Cattle	Northern	0	0	0	0	0	0
2972	Cattle	Northern	0	0	0	0	0	0
2973	Cattle	Northern	0	0	0	0	0	0
2974	Cattle	Northern	0	0	0	0	0	0
2975	Cattle	Northern	0	0	0	0	0	0

2976	Cattle	Northern	0	0	0	0	0	0
2977	Cattle	Northern	0	0	0	0	0	0
2978	Cattle	Northern	0	0	0	0	0	0
2979	Cattle	Northern	0	0	0	0	0	0
2980	Cattle	Northern	0	0	0	0	0	0
2981	Cattle	Northern	0	0	0	0	0	0
2982	Cattle	Northern	0	0	0	0	0	0
2983	Cattle	Northern	0	0	0	0	0	0
2984	Cattle	Northern	0	0	0	0	0	0
2985	Cattle	Northern	0	0	0	0	0	0
2986	Cattle	Northern	0	0	0	0	0	0
2987	Cattle	Northern	0	0	0	0	0	0
2988	Cattle	Northern	0	0	0	0	0	0
2989	Cattle	Northern	0	0	0	0	0	0
2990	Cattle	Northern	0	0	0	0	0	0
2991	Cattle	Northern	0	0	0	0	0	0
2992	Cattle	Northern	0	0	0	0	0	0
2993	Cattle	Northern	0	0	0	0	0	0
2994	Cattle	Central	0	0	0	0	0	0
2995	Cattle	Central	0	0	0	0	0	0
2996	Cattle	Central	0	0	0	0	0	0
2997	Cattle	Central	0	0	0	0	0	0
2998	Cattle	Central	0	0	0	0	0	0
2999	Cattle	Central	0	0	0	0	0	0
3000	Cattle	Central	0	0	0	1	0	1
3001	Cattle	Central	0	0	0	1	0	1
3002	Cattle	Central	0	0	0	0	0	0
3003	Cattle	Central	0	0	0	0	0	0
3004	Cattle	Central	0	0	0	0	0	0
3005	Cattle	Central	0	0	0	0	0	0
3006	Cattle	Central	0	0	0	0	0	0
3007	Cattle	Central	0	0	0	0	0	0
3008	Cattle	Central	0	0	0	0	0	0
3009	Cattle	Central	0	0	0	1	0	1
3010	Cattle	Central	0	0	0	0	0	0
3011	Cattle	Central	0	0	0	0	0	0
3012	Cattle	Central	0	0	0	0	0	0
3013	Cattle	Central	0	0	0	0	0	0
3014	Cattle	Central	0	0	0	0	0	0
3015	Cattle	Central	0	0	0	0	0	0
3016	Cattle	Central	0	0	0	0	0	0
3017	Cattle	Central	0	0	0	0	0	0
3018	Cattle	Central	0	0	0	0	0	0
3019	Cattle	Central	0	0	0	0	0	0
3020	Cattle	Central	0	0	0	0	0	0
3021	Cattle	Central	0	0	0	0	0	0
3022	Cattle	Central	0	0	0	0	0	0
3023	Cattle	Central	0	0	0	0	0	0

3024	Cattle	Central	0	0	0	1	0	1
3025	Cattle	Central	0	0	0	0	0	0
3026	Cattle	Central	0	0	0	0	0	0
3027	Cattle	Central	0	0	0	0	0	0
3028	Cattle	Central	0	0	0	0	0	0
3029	Cattle	Central	0	0	1	0	0	1
3030	Cattle	Central	0	0	0	0	0	0
3031	Cattle	Central	0	0	0	0	0	0
3032	Cattle	Central	0	0	0	0	0	0
3033	Cattle	Central	0	0	0	0	0	0
3034	Cattle	Central	0	0	0	0	0	0
3035	Cattle	Central	0	0	0	0	0	0
3036	Cattle	Central	0	0	0	0	0	0
3037	Cattle	Central	0	0	0	0	0	0
3038	Cattle	Central	0	0	0	0	0	0
3039	Cattle	Central	0	0	0	0	0	0
3040	Cattle	Central	0	0	0	0	0	0
3041	Cattle	Central	0	0	0	0	0	0
3042	Cattle	Central	0	0	0	0	0	0
3043	Cattle	Central	0	0	0	0	0	0
3044	Cattle	Central	0	0	0	0	0	0
3045	Cattle	Central	0	0	0	0	0	0
3046	Cattle	Central	0	0	0	0	0	0
3047	Cattle	Central	0	0	0	0	0	0
3048	Cattle	Central	0	0	0	0	0	0
3049	Cattle	Central	0	0	0	0	0	0
3050	Cattle	Central	0	0	0	0	0	0
3051	Cattle	Central	0	0	0	0	0	0
3052	Cattle	Central	0	0	0	0	0	0
3053	Cattle	Central	0	0	0	0	0	0
3054	Cattle	Central	0	0	0	0	0	0
3055	Cattle	Central	0	0	0	0	0	0
3056	Cattle	Central	0	0	0	0	0	0
3057	Cattle	Central	0	0	0	0	0	0
3058	Cattle	Central	0	0	0	0	0	0
3059	Cattle	Central	0	0	0	0	0	0
3060	Cattle	Central	0	0	0	0	0	0
3061	Cattle	Central	0	0	0	0	0	0
3062	Cattle	Central	0	0	0	0	0	0
3063	Cattle	Central	0	0	0	0	0	0
3064	Cattle	Central	0	0	0	0	0	0
3065	Cattle	Central	0	0	0	0	0	0
3066	Cattle	Central	0	0	0	0	0	0
3067	Cattle	Central	0	0	0	0	0	0
3068	Cattle	Central	0	0	0	0	0	0
3069	Cattle	Central	0	0	0	0	0	0
3070	Cattle	Central	0	0	0	0	0	0
3071	Cattle	Central	0	0	0	0	0	0

3072	Cattle	Central	0	0	0	0	0	0
3073	Cattle	Central	0	0	0	0	0	0
3074	Cattle	Central	0	0	0	0	0	0
3075	Cattle	Central	0	0	0	0	0	0
3076	Cattle	Central	0	0	0	0	0	0
3077	Cattle	Central	0	0	0	0	0	0
3078	Cattle	Central	0	0	0	0	0	0
3079	Cattle	Central	0	0	0	0	0	0
3080	Cattle	Central	0	0	0	0	0	0
3081	Cattle	Central	0	0	0	0	0	0
3082	Cattle	Central	0	0	0	1	0	1
3083	Cattle	Central	0	0	0	0	0	0
3084	Cattle	Central	0	0	0	1	0	1
3085	Cattle	Central	0	1	0	0	0	1
3086	Cattle	Central	0	0	0	0	0	0
3087	Cattle	Central	0	0	0	0	0	0
3088	Cattle	Central	0	0	0	0	0	1
3089	Cattle	Central	0	0	0	0	0	0
3090	Cattle	Central	1	0	0	0	0	1
3091	Cattle	Central	0	0	0	0	0	0
3092	Cattle	Central	0	0	0	0	0	0
3093	Cattle	Central	0	0	0	0	0	0
3094	Cattle	Central	0	0	0	0	0	0
3095	Cattle	Central	0	0	0	0	0	0
3096	Cattle	Central	0	0	0	0	0	1
3097	Cattle	Central	0	0	0	0	0	0
3098	Cattle	Central	0	0	0	0	0	0
3099	Cattle	Central	0	0	0	0	0	0
3100	Cattle	Central	0	0	0	0	0	1
3101	Cattle	Central	0	0	0	0	0	0
3102	Cattle	Central	0	0	0	0	0	0
3103	Cattle	Central	0	0	0	0	0	0
3104	Cattle	Central	0	0	0	0	0	1
3105	Cattle	Central	0	0	0	0	0	0
3106	Cattle	Central	0	0	0	0	0	0
3107	Cattle	Central	0	0	0	0	0	0
3108	Cattle	Central	0	0	0	0	0	0
3109	Cattle	Central	0	0	0	0	1	1
3110	Cattle	Central	0	0	0	0	0	0
3111	Cattle	Central	0	0	0	0	0	0
3112	Cattle	Central	0	0	0	0	0	0
3113	Cattle	Central	0	0	0	0	0	0
3114	Cattle	Central	0	0	0	0	0	0
3115	Cattle	Central	0	0	0	0	0	0
3116	Cattle	Central	0	0	0	1	0	1
3117	Cattle	Central	0	0	0	0	0	0
3118	Cattle	Central	0	0	0	0	0	0
3119	Cattle	Central	0	0	0	0	0	0

3120	Cattle	Central	0	0	0	0	0	0
3121	Cattle	Central	0	0	0	0	0	0
3122	Cattle	Central	0	0	0	0	0	0
3123	Cattle	Central	0	0	0	0	0	0
3124	Cattle	Central	0	0	0	0	0	0
3125	Cattle	Central	0	0	0	0	0	0
3126	Cattle	Central	0	0	0	0	0	0
3127	Cattle	Central	0	0	0	0	0	0
3128	Cattle	Central	0	0	0	0	0	0
3129	Cattle	Central	0	0	0	0	0	0
3130	Cattle	Central	0	0	0	0	0	0
3131	Cattle	Central	0	0	0	0	0	0
3132	Cattle	Central	0	0	0	0	0	0
3133	Cattle	Central	0	0	0	0	0	0
3134	Cattle	Central	0	0	0	0	0	0
3135	Cattle	Central	0	0	0	0	0	0
3136	Cattle	Central	0	0	0	0	0	0
3137	Cattle	Central	0	0	0	0	0	0
3138	Cattle	Central	0	0	0	0	0	0
3139	Cattle	Central	0	0	0	0	0	0
3140	Cattle	Central	0	0	0	0	0	1
3141	Cattle	Central	0	0	0	0	0	0
3142	Cattle	Central	0	0	0	0	0	0
3143	Cattle	Central	0	0	0	0	0	1
3144	Cattle	Central	0	0	0	0	0	0
3145	Cattle	Central	0	0	0	1	0	1
3146	Cattle	Central	0	0	0	0	0	0
3147	Cattle	Central	0	0	0	0	0	0
3148	Cattle	Central	0	0	0	0	0	0
3149	Cattle	Central	0	0	0	0	0	0
3150	Cattle	Central	0	0	0	0	0	0
3151	Cattle	Central	0	0	0	0	0	0
3152	Cattle	Central	0	0	0	0	0	0
3153	Cattle	Central	0	0	0	0	0	0
3154	Cattle	Central	0	0	0	0	0	0
3155	Cattle	Central	0	0	0	0	0	0
3156	Cattle	Central	0	0	0	0	0	0
3157	Cattle	Central	0	0	0	0	0	0
3158	Cattle	Central	0	0	0	0	0	0
3159	Cattle	Central	0	0	0	0	0	0
3160	Cattle	Central	0	0	0	0	0	0
3161	Cattle	Central	0	0	0	0	0	0
3162	Cattle	Central	0	0	0	0	0	0
3163	Cattle	Central	0	0	0	0	0	0
3164	Cattle	Central	0	0	0	0	0	0
3165	Cattle	Central	0	0	0	0	0	0
3166	Cattle	Central	0	0	0	0	0	0
3167	Cattle	Central	0	0	0	0	0	0

3168	Cattle	Central	0	0	0	0	0	0
3169	Cattle	Central	0	0	0	0	0	0
3170	Cattle	Central	0	0	0	0	0	0
3171	Cattle	Central	0	0	0	0	0	0
3172	Cattle	Central	0	0	0	0	0	0
3173	Cattle	Central	0	0	0	0	0	0
3174	Cattle	Central	0	0	0	0	0	0
3175	Cattle	Central	0	0	0	0	0	0
3176	Cattle	Central	0	0	0	0	0	0
3177	Cattle	Central	0	0	0	0	0	0
3178	Cattle	Central	0	0	0	0	0	0
3179	Cattle	Central	0	0	0	0	0	0
3180	Cattle	Central	0	0	0	0	0	0
3181	Cattle	Central	0	0	0	0	0	0
3182	Cattle	Central	0	0	0	0	0	0
3183	Cattle	Central	0	0	0	0	0	0
3184	Cattle	Central	0	0	0	0	0	0
3185	Cattle	Central	0	0	0	0	0	0
3186	Cattle	Central	0	0	0	0	0	0
3187	Cattle	Central	0	0	0	0	0	0
3188	Cattle	Central	0	0	0	0	0	0
3189	Cattle	Central	0	0	0	0	0	0
3190	Cattle	Central	0	0	0	0	0	0
3191	Cattle	Central	0	0	0	0	0	0
3192	Cattle	Central	0	0	0	0	0	0
3193	Cattle	Central	0	0	0	0	0	0
3194	Cattle	Central	0	0	0	0	0	0
3195	Cattle	Central	0	0	0	0	0	0
3196	Cattle	Central	0	0	0	0	0	0
3197	Cattle	Central	0	0	0	0	0	0
3198	Cattle	Central	0	0	0	0	0	0
3199	Cattle	Central	0	0	0	0	0	0
3200	Cattle	Central	0	0	0	0	0	0
3201	Cattle	Central	0	0	0	0	0	0
3202	Cattle	Central	0	0	0	0	0	0
3203	Cattle	Central	0	0	0	0	0	0
3204	Cattle	Central	0	0	0	0	0	0
3205	Cattle	Central	0	0	0	0	0	0
3206	Cattle	Central	0	0	0	0	0	0
3207	Cattle	Central	0	0	0	0	0	0
3208	Cattle	Central	0	0	0	0	0	0
3209	Cattle	Central	0	0	0	0	0	0
3210	Cattle	Central	0	0	0	0	0	0
3211	Cattle	Central	0	0	0	0	0	0
3212	Cattle	Central	0	0	0	0	0	0
3213	Cattle	Central	0	0	0	0	0	0
3214	Cattle	Central	0	0	0	0	0	0
3215	Cattle	Central	0	0	0	0	0	0

3216	Cattle	Central	0	0	0	0	0	0
3217	Cattle	Central	0	0	0	0	0	0
3218	Cattle	Central	0	0	0	0	0	0
3219	Cattle	Central	0	0	0	0	0	0
3220	Cattle	Central	0	0	0	0	0	0
3221	Cattle	Central	0	0	0	0	0	0
3222	Cattle	Central	0	0	0	0	0	0
3223	Cattle	Central	0	0	0	0	0	0
3224	Cattle	Central	0	0	0	0	0	0
3225	Cattle	Central	0	0	0	0	0	0
3226	Cattle	Northern	0	0	0	0	0	0
3227	Cattle	Northern	0	0	0	0	0	0
3228	Cattle	Northern	0	0	0	0	0	0
3229	Cattle	Northern	0	0	0	0	0	0
3230	Cattle	Northern	0	0	0	0	0	0
3231	Cattle	Northern	0	0	0	0	0	0
3232	Cattle	Northern	0	0	0	0	0	0
3233	Cattle	Northern	0	0	0	0	0	0
3234	Cattle	Northern	0	0	0	0	0	0
3235	Cattle	Northern	0	0	0	0	0	0
3236	Cattle	Northern	0	0	0	0	0	0
3237	Cattle	Northern	0	0	0	0	0	0
3238	Cattle	Northern	0	0	0	0	0	0
3239	Cattle	Northern	0	0	0	0	0	0
3240	Cattle	Northern	0	0	0	0	0	0
3241	Cattle	Northern	0	0	0	0	0	0
3242	Cattle	Northern	0	0	0	0	0	0
3243	Cattle	Northern	0	0	0	0	0	0
3244	Cattle	Northern	0	0	0	0	0	0
3245	Cattle	Northern	0	0	0	0	0	0
3246	Cattle	Northern	0	0	0	0	0	0
3247	Cattle	Northern	0	0	0	0	0	0
3248	Cattle	Northern	0	0	0	0	0	0
3249	Cattle	Northern	0	0	0	0	0	0
3250	Cattle	Northern	0	0	0	0	0	0
3251	Cattle	Northern	0	0	0	0	0	0
3252	Cattle	Northern	0	0	0	0	0	0
3253	Cattle	Northern	0	0	0	0	0	0
3254	Cattle	Northern	0	0	0	0	0	0
3255	Cattle	Northern	0	0	0	0	0	0
3256	Cattle	Northern	0	0	0	0	0	0
3257	Cattle	Northern	0	0	0	0	0	0
3258	Cattle	Northern	0	0	0	0	0	0
3259	Cattle	Northern	0	0	0	0	0	0
3260	Cattle	Northern	0	0	0	0	0	0
3261	Cattle	Northern	0	0	0	0	0	0
3262	Cattle	Northern	0	0	0	0	0	0
3263	Cattle	Northern	0	0	0	0	0	0

3264	Cattle	Northern	0	0	0	0	0	0
3265	Cattle	Northern	0	0	0	0	0	0
3266	Cattle	Northern	0	0	0	0	0	0
3267	Cattle	Northern	0	0	0	0	0	0
3268	Cattle	Northern	0	0	0	0	0	0
3269	Cattle	Northern	0	0	0	0	0	0
3270	Cattle	Northern	0	0	0	0	0	0
3271	Cattle	Northern	0	0	0	0	0	0
3272	Cattle	Northern	0	0	0	0	0	0
3273	Cattle	Northern	0	0	0	0	0	0
3274	Cattle	Northern	0	0	0	0	0	0
3275	Cattle	Northern	0	0	0	0	0	0
3276	Cattle	Northern	0	0	0	0	0	0
3277	Cattle	Northern	0	0	0	0	0	0
3278	Cattle	Northern	0	0	0	0	0	0
3279	Cattle	Northern	0	1	0	0	0	1
3280	Cattle	Northern	0	0	0	0	0	0
3281	Cattle	Northern	0	0	0	0	0	0
3282	Cattle	Northern	0	0	0	0	0	0
3283	Cattle	Northern	0	0	0	0	0	0
3284	Cattle	Northern	0	0	0	0	0	0
3285	Cattle	Northern	0	0	0	0	0	0
3286	Cattle	Northern	0	0	0	0	0	0
3287	Cattle	Northern	0	0	0	0	0	0
3288	Cattle	Northern	0	0	0	0	0	0
3289	Cattle	Northern	0	0	0	0	0	0
3290	Cattle	Northern	0	0	0	0	0	0
3291	Cattle	Northern	0	0	0	0	0	0
3292	Cattle	Northern	0	0	0	0	0	0
3293	Cattle	Northern	0	0	0	0	0	0
3294	Cattle	Northern	0	0	0	0	0	0
3295	Cattle	Northern	0	0	0	0	0	0
3296	Cattle	Northern	0	0	0	0	0	0
3297	Cattle	Northern	0	0	0	0	0	0
3298	Cattle	Northern	0	0	0	0	0	0
3299	Cattle	Northern	0	0	0	0	0	0
3300	Cattle	Northern	0	0	0	0	0	0
3301	Cattle	Northern	0	0	0	0	0	0
3302	Cattle	Northern	0	0	0	0	0	0
3303	Cattle	Northern	0	0	0	0	0	0
3304	Cattle	Northern	0	0	0	0	0	0
3305	Cattle	Northern	0	0	0	0	0	0
3306	Cattle	Northern	0	0	0	0	0	0
3307	Cattle	Northern	0	0	0	0	0	0
3308	Cattle	Northern	0	0	0	0	0	0
3309	Cattle	Northern	0	0	0	0	0	0
3310	Cattle	Northern	0	0	0	0	0	0
3311	Cattle	Northern	0	0	0	0	0	0

3312	Cattle	Northern	0	0	0	0	0	0
3313	Cattle	Northern	0	0	0	0	0	0
3314	Cattle	Northern	0	0	0	0	0	0
3315	Cattle	Northern	0	0	0	0	0	0
3316	Cattle	Northern	0	0	0	0	0	0
3317	Cattle	Northern	0	0	0	0	0	0
3318	Cattle	Northern	0	0	0	0	0	0
3319	Cattle	Northern	0	0	0	0	0	0
3320	Cattle	Northern	0	0	0	0	0	0
3321	Cattle	Northern	0	0	0	0	0	0
3322	Cattle	Northern	0	0	0	0	0	0
3323	Cattle	Northern	0	0	0	0	0	0
3324	Cattle	Northern	0	0	0	0	0	0
3325	Cattle	Northern	0	0	0	0	0	0
3326	Cattle	Northern	0	0	0	0	0	0
3327	Cattle	Northern	0	0	0	0	0	0
3328	Cattle	Northern	0	0	0	0	0	0
3329	Cattle	Northern	0	0	0	0	0	0
3330	Cattle	Northern	0	0	0	0	0	0
3331	Cattle	Northern	0	0	0	0	0	0
3332	Cattle	Northern	0	0	0	0	0	0
3333	Cattle	Northern	0	0	0	0	0	0
3334	Cattle	Northern	0	0	0	0	0	0
3335	Cattle	Northern	0	0	0	0	0	0
3336	Cattle	Northern	0	0	0	0	0	0
3337	Cattle	Northern	0	0	0	0	0	0
3338	Cattle	Northern	0	0	0	0	0	0
3339	Cattle	Northern	0	0	0	0	0	0
3340	Cattle	Northern	0	0	0	0	0	0
3341	Cattle	Northern	0	0	0	0	0	0
3342	Cattle	Northern	0	0	0	0	0	0
3343	Cattle	Northern	0	0	0	0	0	0
3344	Cattle	Northern	0	0	0	0	0	0
3345	Cattle	Northern	0	0	0	0	0	0
3346	Cattle	Northern	0	0	0	0	0	0
3347	Cattle	Northern	0	0	0	0	0	0
3348	Cattle	Northern	0	0	0	0	0	0
3349	Cattle	Northern	0	0	0	0	0	0
3350	Cattle	Northern	0	0	0	0	0	0
3351	Cattle	Northern	0	0	0	0	0	0
3352	Cattle	Northern	0	0	0	0	0	0
3353	Cattle	Northern	0	0	0	0	0	0
3354	Cattle	Northern	0	0	0	0	0	0
3355	Cattle	Northern	0	0	0	0	0	0
3356	Cattle	Northern	0	0	0	0	0	0
3357	Cattle	Northern	0	0	0	0	0	0
3358	Cattle	Northern	0	0	0	0	0	0
3359	Cattle	Northern	0	0	0	0	0	0

3360	Cattle	Northern	0	0	0	0	0	0
3361	Cattle	Northern	0	0	0	0	0	0
3362	Cattle	Northern	0	0	0	0	0	0
3363	Cattle	Northern	0	0	0	0	0	0
3364	Cattle	Northern	0	0	0	0	0	0
3365	Cattle	Northern	0	0	0	0	0	0
3366	Cattle	Northern	0	0	0	0	0	0
3367	Cattle	Northern	0	0	0	0	0	0
3368	Cattle	Northern	0	0	0	0	0	0
3369	Cattle	Northern	0	0	0	0	0	0
3370	Cattle	Northern	0	0	0	0	0	0
3371	Cattle	Northern	0	0	0	0	0	0
3372	Cattle	Northern	0	0	0	0	0	0
3373	Cattle	Northern	0	0	0	0	0	0
3374	Cattle	Northern	0	0	0	0	0	0
3375	Cattle	Northern	0	0	0	0	0	0
3376	Cattle	Northern	0	0	0	0	0	0
3377	Cattle	Northern	0	0	0	0	0	0
3378	Cattle	Northern	0	0	0	0	0	0
3379	Cattle	Northern	0	0	0	0	0	0
3380	Cattle	Northern	0	0	0	0	0	0
3381	Cattle	Northern	0	0	0	0	0	0
3382	Cattle	Northern	0	0	0	0	0	0
3383	Cattle	Northern	0	0	0	0	0	0
3384	Cattle	Northern	0	0	0	0	0	0
3385	Cattle	Northern	0	0	0	0	0	0
3386	Cattle	Northern	0	0	0	0	0	0
3387	Cattle	Northern	0	0	0	0	0	0
3388	Cattle	Northern	0	0	0	0	0	0
3389	Cattle	Northern	0	0	0	0	0	0
3390	Cattle	Northern	0	0	0	0	0	0
3391	Cattle	Northern	0	0	0	0	0	0
3392	Cattle	Northern	0	0	0	0	0	0
3393	Cattle	Northern	0	0	0	0	0	0
3394	Cattle	Northern	0	0	0	0	0	0
3395	Cattle	Northern	0	0	0	0	0	0
3396	Cattle	Northern	0	0	0	0	0	0
3397	Cattle	Northern	0	0	0	0	0	0
3398	Cattle	Northern	0	0	0	0	0	0
3399	Cattle	Northern	0	0	0	0	0	0
3400	Cattle	Northern	0	0	0	0	0	0
3401	Cattle	Northern	0	0	0	0	0	0
3402	Cattle	Northern	0	0	0	0	0	0
3403	Cattle	Northern	0	0	0	0	0	0
3404	Cattle	Northern	0	0	0	0	0	0
3405	Cattle	Northern	0	0	0	0	0	0
3406	Cattle	Northern	0	0	0	0	0	0
3407	Cattle	Northern	0	0	0	0	0	0

3408	Cattle	Northern	0	0	0	0	0	0
3409	Cattle	Northern	0	0	0	0	0	0
3410	Cattle	Northern	0	0	0	0	0	0
3411	Cattle	Northern	0	0	0	0	0	0
3412	Cattle	Northern	0	0	0	0	0	0
3413	Cattle	Northern	0	0	0	0	0	0
3414	Cattle	Northern	0	0	0	0	0	0
3415	Cattle	Northern	0	0	0	0	0	0
3416	Cattle	Northern	0	0	0	0	0	0
3417	Cattle	Northern	0	0	0	0	0	0
3418	Cattle	Northern	0	0	0	0	0	0
3419	Cattle	Northern	0	0	0	0	0	0
3420	Cattle	Northern	0	0	0	0	0	0
3421	Cattle	Northern	0	0	0	0	0	0
3422	Cattle	Northern	0	0	0	0	0	0
3423	Cattle	Northern	0	0	0	0	0	0
3424	Cattle	Northern	0	0	0	0	0	0
3425	Cattle	Northern	0	0	0	0	0	0
3426	Cattle	Northern	0	0	0	0	0	0
3427	Cattle	Northern	0	0	0	0	0	0
3428	Cattle	Northern	0	0	0	0	0	0
3429	Cattle	Northern	0	0	0	0	0	0
3430	Cattle	Northern	0	0	0	0	0	0
3431	Cattle	Northern	0	0	0	0	0	0
3432	Cattle	Northern	0	0	0	0	0	0
3433	Cattle	Northern	0	0	0	0	0	0
3434	Cattle	Northern	0	0	0	0	0	0
3435	Cattle	Northern	0	0	0	0	0	0
3436	Cattle	Northern	0	0	0	0	0	0
3437	Cattle	Northern	0	0	0	0	0	0
3438	Cattle	Northern	0	0	0	0	0	0
3439	Cattle	Northern	0	0	0	0	0	0
3440	Cattle	Northern	0	0	0	0	0	0
3441	Cattle	Northern	0	0	0	0	0	0
3442	Cattle	Northern	0	0	0	0	0	0
3443	Cattle	Northern	0	0	0	0	0	0
3444	Cattle	Northern	0	0	0	0	0	0
3445	Cattle	Northern	0	0	0	0	0	0
3446	Cattle	Northern	0	0	0	0	0	0
3447	Cattle	Northern	0	0	0	0	0	0
3448	Cattle	Northern	0	0	0	0	0	0
3449	Cattle	Northern	0	0	0	0	0	0
3450	Cattle	Northern	0	0	0	0	0	0
3451	Cattle	Northern	0	0	0	0	0	0
3452	Cattle	Northeastern	0	0	0	0	0	0
3453	Cattle	Northeastern	0	0	0	0	0	0
3454	Cattle	Northeastern	0	0	0	0	0	0
3455	Cattle	Southern	0	0	0	0	0	0

3456	Cattle	Northeastern	0	0	0	0	0	0
3457	Cattle	Northeastern	0	0	0	0	0	0
3458	Cattle	Northeastern	0	0	0	0	0	0
3459	Cattle	Northeastern	0	0	0	0	0	0
3460	Cattle	Northeastern	0	0	0	0	0	0
3461	Cattle	Northeastern	0	0	0	0	0	0
3462	Cattle	Northeastern	0	0	0	0	0	0
3463	Cattle	Northeastern	0	0	0	0	0	0
3464	Cattle	Northeastern	0	0	0	0	0	0
3465	Cattle	Northeastern	0	0	0	0	0	0
3466	Cattle	Northeastern	0	0	0	0	0	0
3467	Cattle	Northeastern	0	0	0	0	0	0
3468	Cattle	Northeastern	0	0	0	0	0	0
3469	Cattle	Northeastern	0	0	0	0	0	0
3470	Cattle	Northeastern	0	0	0	0	0	0
3471	Cattle	Northeastern	0	0	0	0	0	0
3472	Cattle	Northeastern	0	0	0	0	0	0
3473	Cattle	Northeastern	0	0	0	0	0	0
3474	Cattle	Northeastern	0	0	0	0	0	0
3475	Cattle	Northeastern	0	0	0	0	0	0
3476	Cattle	Northeastern	0	0	0	0	0	0
3477	Cattle	Northeastern	0	0	0	0	0	0
3478	Cattle	Central	0	0	0	0	0	0
3479	Cattle	Central	0	0	0	0	0	0
3480	Cattle	Central	0	0	0	0	0	0
3481	Cattle	Central	0	0	0	0	0	0
3482	Cattle	Central	0	0	0	0	0	0
3483	Cattle	Central	0	0	0	0	0	0
3484	Cattle	Central	0	0	0	0	0	0
3485	Cattle	Central	0	0	0	0	0	0
3486	Cattle	Central	0	0	0	0	0	0
3487	Cattle	Central	0	0	0	0	0	0
3488	Cattle	Central	0	0	0	0	0	0
3489	Cattle	Central	0	0	0	0	0	0
3490	Cattle	Central	0	0	0	0	0	0
3491	Cattle	Central	0	0	0	0	0	0
3492	Cattle	Central	0	0	0	0	0	0
3493	Cattle	Central	0	0	0	0	0	0
3494	Cattle	Central	0	0	0	0	0	0
3495	Cattle	Central	0	0	0	0	0	0
3496	Cattle	Central	0	0	0	0	0	0
3497	Cattle	Central	0	0	0	0	0	0
3498	Cattle	Central	0	0	0	0	0	0
3499	Cattle	Central	0	0	0	0	0	0
3500	Cattle	Central	0	0	0	0	0	0
3501	Cattle	Central	0	0	0	0	0	0
3502	Cattle	Central	0	0	0	0	0	0
3503	Cattle	Central	0	0	0	0	0	0

3504	Cattle	Central	0	0	0	0	0	0
3505	Cattle	Central	0	0	0	0	0	0
3506	Cattle	Central	0	0	0	0	0	0
3507	Cattle	Central	0	0	0	0	0	0
3508	Cattle	Central	0	0	0	0	0	0
3509	Cattle	Central	0	0	0	0	0	0
3510	Cattle	Central	0	0	0	0	0	0
3511	Cattle	Central	0	0	0	0	0	0
3512	Cattle	Central	0	0	0	0	0	0
3513	Cattle	Central	0	0	0	0	0	0
3514	Cattle	Central	0	0	0	0	0	0
3515	Cattle	Central	0	0	0	0	0	0
3516	Cattle	Central	0	0	0	0	0	0
3517	Cattle	Central	0	0	0	0	0	0
3518	Cattle	Central	0	0	0	0	0	0
3519	Cattle	Central	0	0	0	0	0	0
3520	Cattle	Central	0	0	0	0	0	0
3521	Cattle	Central	0	0	0	0	0	0
3522	Cattle	Central	0	0	0	0	0	0
3523	Cattle	Central	0	0	0	0	0	0
3524	Cattle	Central	0	0	0	0	0	0
3525	Cattle	Central	0	0	0	0	0	0
3526	Cattle	Central	0	0	0	0	0	0
3527	Cattle	Central	0	0	0	0	0	0
3528	Cattle	Central	0	0	0	0	0	0
3529	Cattle	Central	0	0	0	0	0	0
3530	Cattle	Central	0	0	0	0	0	0
3531	Cattle	Central	0	0	0	0	0	0
3532	Cattle	Central	0	0	0	0	0	0
3533	Cattle	Central	0	0	0	0	0	0
3534	Cattle	Central	0	0	0	0	0	0
3535	Cattle	Central	0	0	0	0	0	0
3536	Cattle	Central	0	0	0	0	0	0
3537	Cattle	Central	0	0	0	0	0	0
3538	Cattle	Central	0	0	0	0	0	0
3539	Cattle	Central	0	0	0	0	0	0
3540	Cattle	Central	0	0	0	0	0	0
3541	Cattle	Central	0	0	0	0	0	0
3542	Cattle	Central	0	0	0	0	0	0
3543	Cattle	Central	0	0	0	0	0	0
3544	Cattle	Central	0	0	0	0	0	0
3545	Cattle	Central	0	0	0	0	0	0
3546	Cattle	Central	0	0	0	0	0	0
3547	Cattle	Central	0	0	0	0	0	0
3548	Cattle	Central	0	0	0	0	0	0
3549	Cattle	Central	0	0	0	0	0	0
3550	Cattle	Central	0	0	0	0	0	0
3551	Cattle	Central	0	0	0	0	0	0

3552	Cattle	Central	0	0	0	0	0	0
3553	Cattle	Central	0	0	0	0	0	0
3554	Cattle	Central	0	0	0	0	0	0
3555	Cattle	Central	0	0	0	0	0	0
3556	Cattle	Central	0	0	0	0	0	0
3557	Cattle	Central	0	0	0	0	0	0
3558	Cattle	Central	0	0	0	0	0	0
3559	Cattle	Central	0	1	0	0	0	1
3560	Cattle	Central	0	0	0	0	0	0
3561	Cattle	Central	0	0	0	0	0	0
3562	Cattle	Central	0	0	0	0	0	0
3563	Cattle	Central	0	0	0	0	0	0
3564	Cattle	Central	0	0	0	0	0	0
3565	Cattle	Central	0	0	0	0	0	0
3566	Cattle	Northeastern	0	0	0	0	0	0
3567	Cattle	Northeastern	0	0	0	0	0	0
3568	Cattle	Northeastern	0	0	0	0	0	0
3569	Cattle	Northeastern	0	0	0	0	0	0
3570	Cattle	Northeastern	0	0	0	0	0	0
3571	Cattle	Northeastern	0	0	0	0	0	0
3572	Cattle	Northeastern	0	0	0	0	0	0
3573	Cattle	Northeastern	0	0	0	0	0	0
3574	Cattle	Northeastern	0	0	0	0	0	0
3575	Cattle	Northeastern	0	0	0	0	0	0
3576	Cattle	Northeastern	0	0	0	0	0	0
3577	Cattle	Northeastern	0	0	0	0	0	0
3578	Cattle	Northeastern	0	0	0	0	0	0
3579	Cattle	Northeastern	0	0	0	0	0	0
3580	Cattle	Northeastern	0	0	0	0	0	0
3581	Cattle	Northeastern	0	0	0	0	0	0
3582	Cattle	Northeastern	0	0	0	0	0	0
3583	Cattle	Northeastern	0	0	0	0	0	0
3584	Cattle	Northeastern	0	0	0	0	0	0
3585	Cattle	Northeastern	0	0	0	0	0	0
3586	Cattle	Northeastern	0	0	0	0	0	0
3587	Cattle	Northeastern	0	0	0	0	0	0
3588	Cattle	Northeastern	0	0	0	0	0	0
3589	Cattle	Northeastern	0	0	0	0	0	0
3590	Cattle	Northeastern	0	0	0	0	0	0
3591	Cattle	Northeastern	0	0	0	0	0	0
3592	Cattle	Northeastern	0	0	0	0	0	0
3593	Cattle	Northeastern	0	0	0	0	0	0
3594	Cattle	Northeastern	0	0	0	0	0	0
3595	Cattle	Northeastern	0	0	0	0	0	0
3596	Cattle	Northeastern	0	0	0	0	0	0
3597	Cattle	Northeastern	0	0	0	0	0	0
3598	Cattle	Northeastern	0	0	0	0	0	0
3599	Cattle	Northeastern	0	0	0	0	0	0

3600	Cattle	Northeastern	0	0	0	0	0	0
3601	Cattle	Northeastern	0	0	0	0	0	0
3602	Cattle	Northeastern	0	0	0	0	0	0
3603	Cattle	Northeastern	0	0	0	0	0	0
3604	Cattle	Northeastern	0	0	0	0	0	0
3605	Cattle	Northeastern	0	0	0	0	0	0
3606	Cattle	Northeastern	0	0	0	0	0	0
3607	Cattle	Northeastern	0	0	0	0	0	0
3608	Cattle	Northeastern	0	0	0	1	0	1
3609	Cattle	Northeastern	0	0	0	0	0	0
3610	Cattle	Northeastern	0	0	0	0	0	0
3611	Cattle	Northeastern	0	0	0	0	0	0
3612	Cattle	Northeastern	0	0	0	0	0	0
3613	Cattle	Northeastern	0	0	0	0	0	0
3614	Cattle	Northeastern	0	0	0	0	0	0
3615	Cattle	Northeastern	0	0	0	0	0	0
3616	Cattle	Northeastern	0	0	0	0	0	0
3617	Cattle	Northeastern	0	0	0	0	0	0
3618	Cattle	Northeastern	0	0	0	0	0	0
3619	Cattle	Northeastern	0	0	0	0	0	0
3620	Cattle	Northeastern	0	0	0	0	0	0
3621	Cattle	Northeastern	0	0	0	0	0	0
3622	Cattle	Northeastern	0	0	0	0	0	0
3623	Cattle	Northeastern	0	0	0	0	0	0
3624	Cattle	Northeastern	0	0	0	0	0	0
3625	Cattle	Northeastern	0	0	0	0	0	0
3626	Cattle	Northeastern	0	0	0	0	0	0
3627	Cattle	Northeastern	0	0	0	0	0	0
3628	Cattle	Northeastern	0	0	0	0	0	0
3629	Cattle	Northeastern	0	0	0	0	0	0
3630	Cattle	Northeastern	0	0	0	0	0	0
3631	Cattle	Northeastern	0	0	0	0	0	1
3632	Cattle	Northeastern	0	0	0	0	0	0
3633	Cattle	Northeastern	0	0	0	0	0	0
3634	Cattle	Northeastern	0	0	0	0	0	0
3635	Cattle	Northeastern	0	0	0	0	0	0
3636	Cattle	Northeastern	0	0	0	0	0	0
3637	Cattle	Northeastern	0	0	0	0	0	0
3638	Cattle	Northeastern	0	0	0	0	0	0
3639	Cattle	Northeastern	0	0	0	0	0	0
3640	Cattle	Northeastern	0	0	0	0	0	0
3641	Cattle	Northeastern	0	0	0	0	0	0
3642	Cattle	Northeastern	0	0	0	0	0	0
3643	Cattle	Northeastern	0	0	0	0	0	0
3644	Cattle	Northeastern	0	0	0	0	0	0
3645	Cattle	Northeastern	0	0	0	0	0	0
3646	Cattle	Northeastern	0	0	0	0	0	0
3647	Cattle	Northeastern	0	0	0	0	0	0

3648	Cattle	Northeastern	0	0	0	0	0	0
3649	Cattle	Northeastern	0	0	0	0	0	0
3650	Cattle	Northeastern	0	0	1	0	0	1
3651	Cattle	Northeastern	0	1	0	0	0	1
3652	Cattle	Northeastern	0	0	0	0	0	0
3653	Cattle	Northeastern	0	0	0	0	0	0
3654	Cattle	Northeastern	0	0	0	0	0	0
3655	Cattle	Northeastern	0	0	0	0	0	0
3656	Cattle	Northeastern	0	0	0	0	0	0
3657	Cattle	Northeastern	0	0	0	0	0	0
3658	Cattle	Northeastern	0	0	0	0	0	0
3659	Cattle	Northeastern	0	1	0	0	0	1
3660	Cattle	Northeastern	0	0	0	0	0	0
3661	Cattle	Northeastern	0	0	0	0	0	0
3662	Cattle	Northeastern	0	0	0	0	0	0
3663	Cattle	Northeastern	0	0	0	0	0	0
3664	Cattle	Northeastern	0	0	0	0	0	0
3665	Cattle	Northeastern	0	0	0	0	0	0
3666	Cattle	Northeastern	0	0	0	0	0	0
3667	Cattle	Northeastern	0	0	0	0	0	0
3668	Cattle	Northeastern	0	0	0	0	0	0
3669	Cattle	Northeastern	0	0	0	0	0	0
3670	Cattle	Northeastern	0	0	0	0	0	0
3671	Cattle	Northeastern	0	0	0	0	0	0
3672	Cattle	Northeastern	0	0	0	0	0	0
3673	Cattle	Northeastern	0	0	0	0	0	0
3674	Cattle	Northeastern	0	0	0	0	0	0
3675	Cattle	Northeastern	0	0	0	0	0	0
3676	Cattle	Northeastern	0	0	0	0	0	0
3677	Cattle	Northeastern	0	0	0	0	0	0
3678	Cattle	Northeastern	0	0	0	0	0	0
3679	Cattle	Northeastern	0	0	0	1	0	1
3680	Cattle	Northeastern	0	0	0	0	0	0
3681	Cattle	Northeastern	0	0	0	0	0	0
3682	Cattle	Northeastern	0	0	0	0	0	0
3683	Cattle	Northeastern	0	0	0	0	0	0
3684	Cattle	Northeastern	0	0	0	0	0	0
3685	Cattle	Central	0	0	0	0	0	0
3686	Cattle	Central	0	0	0	0	0	0
3687	Cattle	Central	0	0	0	0	0	0
3688	Cattle	Central	0	1	0	0	0	1
3689	Cattle	Central	0	0	0	0	0	0
3690	Cattle	Central	0	0	0	0	0	0
3691	Cattle	Central	0	0	0	0	0	0
3692	Cattle	Central	0	0	0	0	0	1
3693	Cattle	Central	0	0	0	0	0	1
3694	Cattle	Central	0	1	0	0	0	1
3695	Cattle	Central	0	1	0	0	0	1

3696	Cattle	Central	0	0	0	0	0	0
3697	Cattle	Central	0	1	0	0	0	1
3698	Cattle	Central	0	0	0	0	0	0
3699	Cattle	Central	0	0	0	0	0	0
3700	Cattle	Central	0	0	0	0	0	0
3701	Cattle	Central	0	0	0	0	0	0
3702	Cattle	Central	0	0	0	0	0	0
3703	Cattle	Eastern	0	0	0	0	0	0
3704	Cattle	Eastern	0	0	0	0	0	0
3705	Cattle	Eastern	0	0	0	0	0	0
3706	Cattle	Eastern	0	0	0	0	0	0
3707	Cattle	Eastern	0	0	1	1	0	1
3708	Cattle	Eastern	0	0	0	0	0	0
3709	Cattle	Eastern	0	0	0	0	0	0
3710	Cattle	Eastern	0	0	0	0	0	0
3711	Cattle	Eastern	0	0	0	0	0	0
3712	Cattle	Eastern	0	0	0	0	0	0
3713	Cattle	Eastern	0	0	0	0	0	0
3714	Cattle	Eastern	0	0	0	0	0	0
3715	Cattle	Eastern	0	0	0	0	0	0
3716	Cattle	Eastern	0	0	0	0	0	0
3717	Cattle	Central	0	0	0	0	0	0
3718	Cattle	Central	0	0	0	0	0	0
3719	Cattle	Central	0	0	0	0	0	0
3720	Cattle	Central	1	0	0	0	0	1
3721	Cattle	Central	0	1	0	0	0	1
3722	Cattle	Central	0	0	0	0	0	0
3723	Cattle	Central	0	0	0	0	0	0
3724	Cattle	Central	0	0	0	0	0	0
3725	Cattle	Central	0	0	0	0	0	0
3726	Cattle	Central	0	0	0	0	0	0
3727	Cattle	Central	0	0	0	0	0	0
3728	Cattle	Central	0	0	0	0	0	0
3729	Cattle	Central	0	0	0	0	0	0
3730	Cattle	Central	0	0	0	0	0	0
3731	Cattle	Central	0	0	0	0	0	0
3732	Cattle	Central	0	0	0	0	0	0
3733	Cattle	Central	0	0	0	0	0	0
3734	Cattle	Central	0	0	0	0	0	0
3735	Cattle	Central	0	0	0	0	0	0
3736	Cattle	Central	0	0	0	0	0	0
3737	Cattle	Central	0	0	0	0	0	0
3738	Cattle	Central	0	0	0	0	0	0
3739	Cattle	Central	0	0	0	0	0	0
3740	Cattle	Central	0	0	0	0	0	0
3741	Cattle	Central	0	0	0	0	0	0
3742	Cattle	Eastern	0	0	0	0	0	0
3743	Cattle	Eastern	0	0	0	0	0	0

3744	Cattle	Eastern	0	0	0	0	0	0
3745	Cattle	Eastern	0	0	0	0	0	0
3746	Cattle	Eastern	0	0	0	0	0	0
3747	Cattle	Eastern	0	0	0	0	0	0
3748	Cattle	Northeastern	0	0	0	0	0	0
3749	Cattle	Northeastern	0	0	0	0	0	0
3750	Cattle	Northeastern	0	0	0	0	0	0
3751	Cattle	Northeastern	0	0	0	0	0	0
3752	Cattle	Northeastern	0	0	0	0	0	0
3753	Cattle	Northeastern	0	1	0	0	0	1
3754	Cattle	Northeastern	0	0	0	0	0	0
3755	Cattle	Northeastern	0	0	0	0	0	0
3756	Cattle	Northeastern	0	0	0	0	0	0
3757	Cattle	Northeastern	0	0	0	0	0	0
3758	Cattle	Northeastern	0	0	0	0	0	0
3759	Cattle	Northeastern	0	0	0	0	0	0
3760	Cattle	Northeastern	0	0	0	0	0	0
3761	Cattle	Northeastern	0	0	0	0	0	0
3762	Cattle	Northeastern	0	0	0	0	0	0
3763	Cattle	Northeastern	0	0	0	0	0	0
3764	Cattle	Northeastern	0	0	0	0	0	0
3765	Cattle	Northeastern	0	0	0	0	0	0
3766	Cattle	Northeastern	0	0	0	0	0	0
3767	Cattle	Northeastern	0	0	0	0	0	0
3768	Cattle	Northeastern	0	0	0	0	0	0
3769	Cattle	Northeastern	0	0	0	0	0	0
3770	Cattle	Northeastern	0	0	0	0	0	0
3771	Cattle	Northeastern	0	0	0	0	0	0
3772	Cattle	Northeastern	0	0	0	0	0	0
3773	Cattle	Eastern	0	0	0	0	0	0
3774	Cattle	Eastern	0	0	0	0	0	0
3775	Cattle	Eastern	0	0	0	0	0	0
3776	Cattle	Northeastern	0	0	0	0	0	0
3777	Cattle	Northeastern	0	0	0	0	0	0
3778	Cattle	Northeastern	0	0	0	0	0	0
3779	Cattle	Northeastern	0	1	0	0	0	1
3780	Cattle	Northeastern	0	0	0	0	0	0
3781	Cattle	Northeastern	0	0	0	0	0	0
3782	Cattle	Northeastern	0	0	0	0	0	0
3783	Cattle	Northeastern	0	0	0	0	0	0
3784	Cattle	Northeastern	0	0	0	0	0	0
3785	Cattle	Northeastern	0	0	0	0	0	0
3786	Cattle	Northeastern	0	0	0	0	0	0
3787	Cattle	Northeastern	0	0	0	0	0	0
3788	Cattle	Central	0	0	0	0	0	0
3789	Cattle	Central	0	0	0	0	0	0
3790	Cattle	Central	0	0	0	0	0	0
3791	Cattle	Central	0	0	0	0	0	0

3792	Cattle	Central	0	0	0	0	0	0
3793	Cattle	Central	0	0	0	0	0	0
3794	Cattle	Central	0	0	0	0	0	0
3795	Cattle	Central	0	0	0	0	0	0
3796	Cattle	Central	0	0	0	0	0	0
3797	Cattle	Central	0	0	0	0	0	0
3798	Cattle	Central	0	0	0	0	0	0
3799	Cattle	Central	0	0	0	0	0	0
3800	Cattle	Central	0	0	0	0	0	0
3801	Cattle	Central	0	0	0	0	0	0
3802	Cattle	Central	0	0	0	0	0	0
3803	Cattle	Central	0	0	0	0	0	0
3804	Cattle	Central	0	0	0	0	0	0
3805	Cattle	Central	0	0	0	0	0	0
3806	Cattle	Central	0	0	0	0	0	0
3807	Cattle	Central	0	0	0	0	0	0
3808	Cattle	Central	0	0	0	0	0	0
3809	Cattle	Central	0	0	0	0	0	0
3810	Cattle	Central	0	0	0	0	0	0
3811	Cattle	Central	0	0	0	0	0	0
3812	Cattle	Central	0	0	0	0	0	0
3813	Cattle	Central	0	0	0	0	0	0
3814	Cattle	Central	0	0	0	0	0	0
3815	Cattle	Central	0	0	0	0	0	0
3816	Cattle	Central	0	0	0	0	0	0
3817	Cattle	Central	0	0	0	0	0	0
3818	Cattle	Central	0	0	0	0	0	0
3819	Cattle	Central	0	0	0	0	0	0
3820	Cattle	Central	0	0	0	0	0	0
3821	Cattle	Central	0	0	0	0	0	0
3822	Cattle	Central	0	0	0	0	0	0
3823	Cattle	Central	0	0	0	0	0	0
3824	Cattle	Central	0	0	0	0	0	0
3825	Cattle	Central	0	0	0	0	0	0
3826	Cattle	Central	0	0	0	0	0	0
3827	Cattle	Central	0	0	0	0	0	0
3828	Cattle	Central	0	0	0	0	0	0
3829	Cattle	Central	0	0	0	0	0	0
3830	Cattle	Central	0	0	0	0	0	0
3831	Cattle	Central	0	0	0	0	0	0
3832	Cattle	Central	0	0	0	0	0	0
3833	Cattle	Central	0	0	0	0	0	0
3834	Cattle	Central	0	0	0	0	0	0
3835	Cattle	Northeastern	0	0	0	0	0	0
3836	Cattle	Northeastern	0	0	0	0	0	0
3837	Cattle	Northeastern	0	0	0	0	0	0
3838	Cattle	Northeastern	0	0	0	0	0	0
3839	Cattle	Northeastern	0	0	0	1	0	1

3840	Cattle	Northeastern	0	0	0	0	0	0
3841	Cattle	Northeastern	0	0	0	0	0	0
3842	Cattle	Northeastern	0	0	0	0	0	0
3843	Cattle	Northeastern	0	0	1	1	0	1
3844	Cattle	Central	0	0	0	0	0	0
3845	Cattle	Central	0	0	0	0	0	0
3846	Cattle	Central	0	0	1	0	0	1
3847	Cattle	Central	0	0	0	0	0	0
3848	Cattle	Central	0	0	0	0	0	0
3849	Cattle	Central	0	0	0	0	0	0
3850	Cattle	Central	0	0	0	0	0	0
3851	Cattle	Central	0	0	0	0	0	0
3852	Cattle	Central	0	0	0	0	0	0
3853	Cattle	Central	0	0	0	0	0	0
3854	Cattle	Central	0	0	0	0	0	0
3855	Cattle	Central	0	0	0	0	0	0
3856	Cattle	Central	0	0	0	0	0	0
3857	Cattle	Central	0	0	0	0	0	0
3858	Cattle	Central	0	0	0	0	0	0
3859	Cattle	Central	0	0	0	0	0	0
3860	Cattle	Central	0	0	0	0	0	0
3861	Cattle	Central	0	0	0	0	0	0
3862	Cattle	Central	0	0	0	0	0	0
3863	Cattle	Central	0	0	0	0	0	0
3864	Cattle	Central	0	0	0	0	0	0
3865	Cattle	Central	0	0	0	0	0	0
3866	Cattle	Central	0	0	0	0	0	0
3867	Cattle	Central	0	0	0	0	0	0
3868	Cattle	Central	0	0	0	1	0	1
3869	Cattle	Central	0	0	0	0	0	0
3870	Cattle	Central	0	0	0	0	0	0
3871	Cattle	Central	0	0	0	0	0	0
3872	Cattle	Central	0	0	0	0	0	0
3873	Cattle	Central	0	0	0	0	0	0
3874	Cattle	Central	0	0	0	0	0	0
3875	Cattle	Central	0	0	0	0	0	0
3876	Cattle	Central	0	0	0	0	0	0
3877	Cattle	Central	0	0	0	0	0	0
3878	Cattle	Central	0	0	0	0	0	0
3879	Cattle	Central	0	0	1	1	0	1
3880	Cattle	Central	0	0	0	0	0	0
3881	Cattle	Central	0	0	0	0	0	0
3882	Cattle	Central	0	0	0	0	0	0
3883	Cattle	Central	0	0	0	1	0	1
3884	Cattle	Central	0	0	0	1	0	1
3885	Cattle	Central	0	0	0	1	0	1
3886	Cattle	Central	0	1	0	1	0	1
3887	Cattle	Central	0	1	0	0	0	1

3888	Cattle	Central	0	1	0	1	0	1
3889	Cattle	Central	0	0	0	0	0	0
3890	Cattle	Central	0	1	0	1	0	1
3891	Cattle	Central	0	0	0	0	0	0
3892	Cattle	Central	0	0	0	1	0	1
3893	Cattle	Central	0	1	0	0	0	1
3894	Cattle	Central	0	1	0	1	0	1
3895	Cattle	Central	0	1	1	1	0	1
3896	Cattle	Central	0	0	0	0	0	0
3897	Cattle	Central	0	0	0	0	0	0
3898	Cattle	Southern	0	0	0	0	0	0
3899	Cattle	Southern	0	0	0	0	0	0
3900	Cattle	Southern	0	0	1	1	0	1
3901	Cattle	Southern	0	0	0	0	0	0
3902	Cattle	Central	0	0	0	0	0	0
3903	Cattle	Central	0	0	0	1	0	1
3904	Cattle	Central	0	0	0	0	0	0
3905	Cattle	Central	0	0	0	0	0	0
3906	Cattle	Central	0	0	0	0	0	0
3907	Cattle	Central	0	0	0	0	0	0
3908	Cattle	Central	0	0	0	0	0	0
3909	Cattle	Central	0	0	0	0	0	0
3910	Cattle	Central	0	0	0	0	0	0
3911	Cattle	Central	0	0	0	0	0	0
3912	Cattle	Central	0	0	0	0	0	0
3913	Cattle	Central	0	0	0	0	0	0
3914	Cattle	Central	0	0	0	0	0	0
3915	Cattle	Central	0	0	0	0	0	0
3916	Cattle	Central	0	0	0	0	0	0
3917	Cattle	Central	0	0	1	1	0	1
3918	Cattle	Central	0	0	1	1	0	1
3919	Cattle	Central	0	0	1	1	0	1
3920	Cattle	Central	0	0	1	1	0	1
3921	Cattle	Central	0	0	0	0	0	0
3922	Cattle	Central	0	0	0	0	0	0
3923	Cattle	Central	0	0	1	1	0	1
3924	Cattle	Central	0	0	0	0	0	0
3925	Cattle	Central	0	0	0	1	0	1
3926	Cattle	Central	0	0	0	0	0	0
3927	Cattle	Northeastern	0	0	0	0	0	0
3928	Cattle	Northeastern	0	0	0	0	0	0
3929	Cattle	Northeastern	0	0	0	0	0	0
3930	Cattle	Northeastern	0	0	0	0	0	0
3931	Cattle	Northeastern	0	0	0	0	0	0
3932	Cattle	Northeastern	0	0	0	0	0	0
3933	Cattle	Northeastern	0	0	0	0	0	0
3934	Cattle	Northeastern	0	0	0	1	0	1
3935	Cattle	Northeastern	0	0	0	0	0	0

3936	Cattle	Northeastern	0	0	0	0	0	0
3937	Cattle	Northeastern	0	0	0	0	0	0
3938	Cattle	Northeastern	0	0	0	0	0	0
3939	Cattle	Northeastern	0	0	0	0	0	0
3940	Cattle	Northeastern	0	0	0	0	0	0
3941	Cattle	Northeastern	0	0	0	0	0	0
3942	Cattle	Northeastern	0	0	0	0	0	0
3943	Cattle	Northeastern	0	0	0	0	0	0
3944	Cattle	Northeastern	0	0	0	0	0	0
3945	Cattle	Northeastern	0	0	0	0	0	0
3946	Cattle	Northeastern	0	0	0	0	0	0
3947	Cattle	Northeastern	0	0	0	0	0	0
3948	Cattle	Northeastern	0	0	0	0	0	0
3949	Cattle	Northeastern	0	0	0	0	0	0
3950	Cattle	Northeastern	0	0	0	1	0	1
3951	Cattle	Northeastern	0	0	0	1	0	1
3952	Cattle	Northeastern	0	0	0	0	0	0
3953	Cattle	Northeastern	0	0	0	0	0	0
3954	Cattle	Northeastern	0	0	0	0	0	0
3955	Cattle	Northeastern	0	0	0	0	0	0
3956	Cattle	Northeastern	0	0	0	0	0	0
3957	Cattle	Northeastern	0	0	0	0	0	0
3958	Cattle	Northeastern	0	0	0	0	0	0
3959	Cattle	Northeastern	0	0	0	0	0	0
3960	Cattle	Northeastern	0	0	0	0	0	0
3961	Cattle	Northeastern	0	0	0	0	0	0
3962	Cattle	Northeastern	0	0	0	0	0	0
3963	Cattle	Northeastern	0	0	0	0	0	0
3964	Cattle	Northeastern	0	0	0	0	0	0
3965	Cattle	Northeastern	0	0	0	0	0	0
3966	Cattle	Northeastern	0	0	0	0	0	0
3967	Cattle	Northeastern	0	0	0	0	0	0
3968	Cattle	Northeastern	0	0	0	0	0	0
3969	Cattle	Northeastern	0	0	0	0	0	0
3970	Cattle	Northeastern	0	0	0	0	0	0
3971	Cattle	Northeastern	0	0	0	0	0	0
3972	Cattle	Northeastern	0	0	0	0	0	0
3973	Cattle	Northeastern	0	0	0	0	0	0
3974	Cattle	Northeastern	0	0	0	0	0	0
3975	Cattle	Northeastern	0	0	0	0	0	0
3976	Cattle	Northeastern	0	0	0	0	0	0
3977	Cattle	Northeastern	0	0	0	0	0	0
3978	Cattle	Northeastern	0	0	0	0	0	0
3979	Cattle	Northeastern	0	0	0	0	0	0
3980	Cattle	Northeastern	0	0	0	0	0	0
3981	Cattle	Northeastern	0	0	0	0	0	0
3982	Cattle	Northeastern	0	0	0	0	0	0
3983	Cattle	Northeastern	0	0	0	0	0	0

3984	Cattle	Northeastern	0	0	0	0	0	0
3985	Cattle	Northeastern	0	0	0	0	0	0
3986	Cattle	Eastern	0	0	0	0	0	0
3987	Cattle	Eastern	0	0	0	0	0	0
3988	Cattle	Eastern	0	0	0	0	0	0
3989	Cattle	Eastern	0	0	0	0	0	0
3990	Cattle	Eastern	0	0	0	0	0	0
3991	Cattle	Eastern	0	0	0	0	0	0
3992	Cattle	Eastern	0	0	0	0	0	0
3993	Cattle	Eastern	0	0	0	0	0	0
3994	Cattle	Eastern	0	0	0	0	0	0
3995	Cattle	Eastern	0	0	0	0	0	0
3996	Cattle	Eastern	0	0	0	0	0	0
3997	Cattle	Eastern	0	0	0	0	0	0
3998	Cattle	Eastern	0	0	0	0	0	0
3999	Cattle	Eastern	0	0	0	0	0	0
4000	Cattle	Eastern	0	0	0	0	0	0
4001	Cattle	Eastern	0	0	0	0	0	0
4002	Cattle	Eastern	0	0	0	0	0	0
4003	Cattle	Eastern	0	0	0	0	0	0
4004	Cattle	Eastern	0	0	0	0	0	0
4005	Cattle	Eastern	0	0	0	0	0	0
4006	Cattle	Eastern	0	0	0	0	0	0
4007	Cattle	Eastern	0	0	0	0	0	0
4008	Cattle	Eastern	0	0	0	0	0	0
4009	Cattle	Eastern	0	0	0	0	0	0
4010	Cattle	Eastern	0	0	0	0	0	0
4011	Cattle	Eastern	0	0	0	0	0	0
4012	Cattle	Eastern	0	0	0	0	0	0
4013	Cattle	Eastern	0	0	0	0	0	0
4014	Cattle	Eastern	0	0	0	0	0	0
4015	Cattle	Eastern	0	0	0	0	0	0
4016	Cattle	Northeastern	0	0	0	0	0	0
4017	Cattle	Northeastern	0	0	0	0	0	0
4018	Cattle	Northeastern	0	0	1	1	0	1
4019	Cattle	Northeastern	0	0	0	0	0	0
4020	Cattle	Northeastern	0	0	0	0	0	0
4021	Cattle	Northeastern	0	0	0	0	0	0
4022	Cattle	Northeastern	0	0	0	0	0	0
4023	Cattle	Northeastern	0	0	0	0	0	0
4024	Cattle	Northeastern	0	0	0	0	0	0
4025	Cattle	Northeastern	0	0	0	0	0	0
4026	Cattle	Northeastern	0	0	0	0	0	0
4027	Cattle	Northeastern	0	0	0	1	0	1
4028	Cattle	Northeastern	0	0	0	0	0	0
4029	Cattle	Northeastern	0	0	0	0	0	0
4030	Cattle	Northeastern	0	0	0	0	0	0
4031	Cattle	Eastern	0	0	0	0	0	0

4032	Cattle	Eastern	0	0	0	0	0	0
4033	Cattle	Eastern	0	0	1	1	0	1
4034	Cattle	Eastern	0	0	0	0	0	0
4035	Cattle	Eastern	0	0	0	0	0	0
4036	Cattle	Eastern	0	0	0	0	0	0
4037	Cattle	Eastern	0	0	0	0	0	0
4038	Cattle	Eastern	0	0	0	0	0	0
4039	Cattle	Eastern	0	0	0	0	0	0
4040	Cattle	Eastern	0	0	0	0	0	0
4041	Cattle	Eastern	0	0	0	0	0	0
4042	Cattle	Eastern	0	0	0	0	0	0
4043	Cattle	Eastern	0	0	0	0	0	0
4044	Cattle	Eastern	0	0	0	0	0	0
4045	Cattle	Eastern	0	0	0	0	0	0
4046	Cattle	Eastern	0	0	0	0	0	0
4047	Cattle	Eastern	0	0	0	0	0	0
4048	Cattle	Eastern	0	0	0	0	0	0
4049	Cattle	Eastern	0	0	0	0	0	0
4050	Cattle	Eastern	0	0	0	0	0	0
4051	Cattle	Eastern	0	0	0	0	0	0
4052	Cattle	Eastern	0	0	0	0	0	0
4053	Cattle	Eastern	0	0	0	0	0	0
4054	Cattle	Eastern	0	0	0	0	0	0
4055	Cattle	Eastern	0	0	0	0	0	0
4056	Cattle	Eastern	0	0	0	0	0	0
4057	Cattle	Eastern	0	0	0	0	0	0
4058	Cattle	Eastern	0	0	0	0	0	0
4059	Cattle	Eastern	0	0	0	0	0	0
4060	Cattle	Eastern	0	0	0	0	0	0
4061	Cattle	Eastern	0	0	0	0	0	0
4062	Cattle	Eastern	0	0	0	0	0	0
4063	Cattle	Eastern	0	0	0	0	0	0
4064	Cattle	Eastern	0	0	0	0	0	0
4065	Cattle	Eastern	0	0	1	1	0	1
4066	Cattle	Eastern	0	0	1	1	0	1
4067	Cattle	Eastern	0	0	1	1	0	1
4068	Cattle	Eastern	0	0	1	1	0	1
4069	Cattle	Eastern	0	0	0	0	0	0
4070	Cattle	Eastern	0	0	1	1	0	1
4071	Cattle	Eastern	0	0	0	0	0	0
4072	Cattle	Eastern	0	0	0	0	0	0
4073	Cattle	Eastern	0	0	0	0	0	0
4074	Cattle	Eastern	0	0	0	0	0	0
4075	Cattle	Eastern	0	0	0	0	0	0
4076	Cattle	Eastern	0	0	1	1	0	1
4077	Cattle	Eastern	0	0	1	1	0	1
4078	Cattle	Eastern	0	0	0	0	0	0
4079	Cattle	Eastern	0	0	0	0	0	0

4080	Cattle	Eastern	0	0	0	0	0	0
4081	Cattle	Eastern	0	0	0	0	0	0
4082	Cattle	Eastern	0	0	1	0	0	1
4083	Cattle	Eastern	0	0	1	0	0	1
4084	Cattle	Eastern	0	0	0	0	0	0
4085	Cattle	Eastern	0	0	1	0	0	1
4086	Cattle	Eastern	0	0	1	0	0	1
4087	Cattle	Eastern	0	0	1	0	0	1
4088	Cattle	Eastern	0	0	1	1	0	1
4089	Cattle	Eastern	0	0	0	0	0	0
4090	Cattle	Eastern	0	0	1	0	0	1
4091	Cattle	Eastern	0	0	0	0	0	0
4092	Cattle	Eastern	0	0	1	0	0	1
4093	Cattle	Eastern	0	0	0	0	0	0
4094	Cattle	Eastern	0	0	0	0	0	0
4095	Cattle	Eastern	0	0	0	0	0	0
4096	Cattle	Eastern	0	0	0	0	0	0
4097	Cattle	Central	0	0	1	1	0	1
4098	Cattle	Central	0	0	0	0	0	0
4099	Cattle	Central	0	0	1	1	0	1
4100	Cattle	Central	0	0	0	1	0	1
4101	Cattle	Central	0	0	0	0	0	0
4102	Cattle	Central	0	0	0	0	0	0
4103	Cattle	Central	0	0	0	0	0	0
4104	Cattle	Central	0	0	0	0	0	1
4105	Cattle	Central	0	0	0	1	0	1
4106	Cattle	Central	0	1	0	0	0	1
4107	Cattle	Central	0	0	0	0	0	0
4108	Cattle	Central	0	0	0	1	0	1
4109	Cattle	Central	0	0	0	0	0	0
4110	Cattle	Central	0	1	0	1	0	1
4111	Cattle	Central	0	0	0	1	0	1
4112	Cattle	Central	0	0	0	0	0	0
4113	Cattle	Central	0	0	0	0	0	0
4114	Cattle	Central	0	0	0	0	0	0
4115	Cattle	Central	0	0	0	0	0	0
4116	Cattle	Northeastern	0	0	0	0	0	0
4117	Cattle	Northeastern	0	1	0	0	0	1
4118	Cattle	Northeastern	0	0	0	0	0	0
4119	Cattle	Northeastern	0	0	0	0	0	0
4120	Cattle	Northeastern	0	0	0	0	0	0
4121	Cattle	Northeastern	0	0	0	0	0	0
4122	Cattle	Northeastern	0	0	0	0	0	0
4123	Cattle	Northeastern	0	0	0	0	0	0
4124	Cattle	Central	0	0	0	0	0	0
4125	Cattle	Northern	0	0	0	0	0	0
4126	Cattle	Northern	0	0	0	0	0	0
4127	Cattle	Northern	0	0	0	0	0	0

4128	Cattle	Northern	0	0	0	0	0	0
4129	Cattle	Northern	0	0	0	0	0	0
4130	Cattle	Northern	0	0	0	0	0	0
4131	Cattle	Northern	0	0	0	0	0	0
4132	Cattle	Northern	0	0	0	0	0	0
4133	Cattle	Northern	0	0	0	0	0	0
4134	Cattle	Northern	0	0	0	0	0	0
4135	Cattle	Northern	0	0	0	0	0	0
4136	Cattle	Northern	0	0	0	0	0	0
4137	Cattle	Northern	0	0	0	0	0	0
4138	Cattle	Northern	0	0	0	0	0	0
4139	Cattle	Northern	0	0	0	0	0	0
4140	Cattle	Northern	0	0	0	0	0	0
4141	Cattle	Northern	0	0	0	0	0	0
4142	Cattle	Central	0	0	0	0	0	0
4143	Cattle	Central	0	0	0	0	0	0
4144	Cattle	Central	0	0	0	0	0	0
4145	Cattle	Central	0	0	0	0	0	0
4146	Cattle	Central	0	0	0	0	0	0
4147	Cattle	Central	0	0	0	0	0	0
4148	Cattle	Central	0	0	0	0	0	0
4149	Cattle	Central	0	0	0	0	0	0
4150	Cattle	Central	0	0	0	0	0	0
4151	Cattle	Central	0	0	0	0	0	0
4152	Cattle	Central	0	0	0	0	0	0
4153	Cattle	Central	0	0	0	0	0	0
4154	Cattle	Central	0	0	0	0	0	0
4155	Cattle	Central	0	0	0	0	0	0
4156	Cattle	Central	0	0	0	0	0	0
4157	Cattle	Central	0	0	0	0	0	0
4158	Cattle	Central	0	0	0	0	0	0
4159	Cattle	Central	0	0	0	0	0	0
4160	Cattle	Central	0	0	0	0	0	0
4161	Cattle	Central	0	0	0	0	0	0
4162	Cattle	Central	0	0	0	0	0	0
4163	Cattle	Central	0	0	0	0	0	0
4164	Cattle	Central	0	0	0	0	0	0
4165	Cattle	Eastern	0	0	1	1	0	1
4166	Cattle	Eastern	0	0	1	0	0	1
4167	Cattle	Eastern	0	0	1	1	0	1
4168	Cattle	Eastern	0	0	1	1	0	1
4169	Cattle	Eastern	0	0	0	0	0	0
4170	Cattle	Eastern	0	0	0	0	0	0
4171	Cattle	Eastern	0	0	0	0	0	0
4172	Cattle	Northeastern	0	0	0	0	0	0
4173	Cattle	Northeastern	0	0	0	0	0	0
4174	Cattle	Northeastern	0	0	0	0	0	0
4175	Cattle	Northeastern	0	0	0	0	0	0

4176	Cattle	Central	0	1	0	0	0	1
4177	Cattle	Central	0	1	0	0	0	1
4178	Cattle	Central	0	0	0	0	0	0
4179	Cattle	Central	0	1	0	0	0	1
4180	Cattle	Central	0	0	0	0	0	0
4181	Cattle	Central	0	0	0	0	0	0
4182	Cattle	Central	0	0	0	0	0	0
4183	Cattle	Central	0	0	0	0	0	0
4184	Cattle	Central	0	1	0	0	0	1
4185	Cattle	Central	0	1	0	0	0	1
4186	Cattle	Central	0	1	0	0	0	1
4187	Cattle	Central	0	0	0	0	0	0
4188	Cattle	Central	0	0	0	0	0	0
4189	Cattle	Central	0	0	0	1	0	1
4190	Cattle	Central	0	0	0	0	0	0
4191	Cattle	Central	0	0	0	0	0	0
4192	Cattle	Central	0	0	0	0	0	0
4193	Cattle	Central	0	0	0	0	0	0
4194	Cattle	Central	0	0	0	0	0	0
4195	Cattle	Central	0	0	0	0	0	0
4196	Cattle	Central	0	0	0	0	0	0
4197	Cattle	Central	0	0	0	0	0	0
4198	Cattle	Central	0	0	0	0	0	0
4199	Cattle	Central	0	0	0	0	0	0
4200	Cattle	Central	0	0	0	0	0	0
4201	Cattle	Central	0	0	0	0	0	0
4202	Cattle	Central	0	0	0	0	0	0
4203	Cattle	Central	0	0	0	0	0	0
4204	Cattle	Central	0	0	0	0	0	0
4205	Cattle	Central	0	1	0	0	0	1
4206	Cattle	Central	0	1	0	0	0	1
4207	Cattle	Central	0	0	0	0	0	0
4208	Cattle	Central	0	1	0	0	0	1
4209	Cattle	Central	0	0	0	0	0	0
4210	Cattle	Central	0	0	0	0	0	0
4211	Cattle	Central	0	0	0	0	0	0
4212	Cattle	Central	0	0	0	0	0	0
4213	Cattle	Central	0	0	0	0	0	0
4214	Cattle	Central	0	0	0	0	0	0
4215	Cattle	Central	0	0	0	0	0	0
4216	Cattle	Central	0	0	0	0	0	0
4217	Cattle	Central	0	0	0	0	0	0
4218	Cattle	Central	0	0	0	0	0	0
4219	Cattle	Central	0	0	1	0	0	1
4220	Cattle	Central	0	0	0	0	0	0
4221	Cattle	Northeastern	0	0	0	0	0	0
4222	Cattle	Northeastern	0	0	0	0	0	0
4223	Cattle	Northeastern	0	0	0	0	0	0

4224	Cattle	Northeastern	0	0	0	0	0	0
4225	Cattle	Northeastern	0	0	0	0	0	0
4226	Cattle	Northeastern	0	0	0	0	0	0
4227	Cattle	Northeastern	0	0	0	0	0	0
4228	Cattle	Northeastern	0	0	0	0	0	0
4229	Cattle	Northeastern	0	0	0	0	0	0
4230	Cattle	Eastern	0	0	0	0	0	0
4231	Cattle	Eastern	0	0	1	1	0	1
4232	Cattle	Eastern	0	0	1	1	0	1
4233	Cattle	Eastern	0	0	1	1	0	1
4234	Cattle	Eastern	0	1	1	1	0	1
4235	Cattle	Eastern	0	0	0	0	0	0
4236	Cattle	Eastern	0	0	1	1	0	1
4237	Cattle	Eastern	0	0	0	0	0	0
4238	Cattle	Eastern	0	0	1	1	0	1
4239	Cattle	Eastern	0	1	1	1	0	1
4240	Cattle	Eastern	0	1	1	0	0	1
4241	Cattle	Eastern	0	0	1	0	0	1
4242	Cattle	Eastern	0	0	0	0	0	0
4243	Cattle	Northeastern	0	0	0	1	0	1
4244	Cattle	Northeastern	0	0	1	1	0	1
4245	Cattle	Northeastern	0	0	1	1	0	1
4246	Cattle	Northeastern	0	0	1	1	0	1
4247	Cattle	Northeastern	0	0	1	1	0	1
4248	Cattle	Northeastern	0	0	0	1	0	1
4249	Cattle	Northeastern	0	0	0	0	0	0
4250	Cattle	Northeastern	0	0	0	0	0	0
4251	Cattle	Northeastern	0	0	0	0	0	0
4252	Cattle	Northeastern	0	0	0	0	0	0
4253	Cattle	Northeastern	0	0	0	1	0	1
4254	Cattle	Northeastern	0	0	0	0	0	0
4255	Cattle	Northeastern	0	0	0	1	0	1
4256	Cattle	Northeastern	0	0	0	0	0	0
4257	Cattle	Northeastern	0	0	0	0	0	0
4258	Cattle	Northeastern	0	0	0	0	0	0
4259	Cattle	Northeastern	0	0	0	0	1	1
4260	Cattle	Central	0	0	0	0	0	0
4261	Cattle	Northern	0	0	0	0	0	0
4262	Cattle	Northern	0	0	0	0	0	0
4263	Cattle	Northeastern	0	0	0	0	0	0
4264	Cattle	Northeastern	0	0	0	0	0	0
4265	Cattle	Northeastern	0	0	0	1	0	1
4266	Cattle	Northeastern	0	0	0	0	0	0
4267	Cattle	Northeastern	0	0	0	0	0	0
4268	Cattle	Northeastern	0	0	0	1	0	1
4269	Cattle	Northeastern	0	0	0	0	0	0
4270	Cattle	Northeastern	0	0	0	0	0	0
4271	Cattle	Northeastern	0	0	0	0	0	0

4272	Cattle	Northeastern	0	0	0	0	0	0
4273	Cattle	Northeastern	0	0	0	0	0	0
4274	Cattle	Northeastern	0	0	1	1	0	1
4275	Cattle	Northeastern	0	0	0	0	0	0
4276	Cattle	Northeastern	0	0	1	1	0	1
4277	Cattle	Northeastern	0	0	0	0	0	0
4278	Cattle	Northeastern	0	0	0	0	0	0
4279	Cattle	Northeastern	0	0	0	0	0	0
4280	Cattle	Northeastern	0	0	0	0	0	0
4281	Cattle	Northeastern	0	0	0	0	0	0
4282	Cattle	Northeastern	0	0	0	0	0	0
4283	Cattle	Northeastern	0	0	0	0	0	0
4284	Cattle	Northeastern	0	0	0	0	0	0
4285	Cattle	Northeastern	0	0	0	0	0	0
4286	Cattle	Northeastern	0	0	0	0	0	0
4287	Cattle	Northeastern	0	0	0	0	0	0
4288	Cattle	Central	0	0	1	1	0	1
4289	Cattle	Central	0	0	0	0	0	0
4290	Cattle	Central	0	0	0	0	0	0
4291	Cattle	Central	0	0	0	0	0	0
4292	Cattle	Central	0	0	0	0	0	0
4293	Cattle	Central	0	0	0	0	0	0
4294	Cattle	Central	0	0	0	0	0	0
4295	Cattle	Central	0	0	0	0	0	0
4296	Cattle	Central	0	0	0	0	0	0
4297	Cattle	Central	0	0	0	0	0	0
4298	Cattle	Central	0	0	0	0	0	0
4299	Cattle	Central	0	0	0	0	0	0
4300	Cattle	Central	0	0	0	0	0	0
4301	Cattle	Central	0	0	0	0	0	0
4302	Cattle	Central	0	0	0	0	0	0
4303	Cattle	Central	0	0	0	0	0	0
4304	Cattle	Central	0	0	1	0	0	1
4305	Cattle	Central	0	0	0	0	0	0
4306	Cattle	Central	0	0	0	0	0	0
4307	Cattle	Central	0	0	0	0	0	0
4308	Cattle	Central	0	0	1	1	0	1
4309	Cattle	Central	0	0	0	0	0	0
4310	Cattle	Central	0	0	0	0	0	0
4311	Cattle	Central	0	0	0	0	0	0
4312	Cattle	Central	0	0	0	0	0	0
4313	Cattle	Central	0	0	0	0	0	0
4314	Cattle	Central	0	0	0	0	0	0
4315	Cattle	Central	0	0	1	0	0	1
4316	Cattle	Central	0	0	0	0	0	0
4317	Cattle	Central	0	0	0	0	0	0
4318	Cattle	Central	0	0	0	0	0	0
4319	Cattle	Central	0	0	0	0	0	0

4320	Cattle	Central	0	0	0	0	0	0
4321	Cattle	Central	0	0	0	0	0	0
4322	Cattle	Central	0	0	1	0	0	1
4323	Cattle	Central	0	0	0	0	0	0
4324	Cattle	Northeastern	0	0	0	0	0	0
4325	Cattle	Northeastern	0	0	0	0	0	0
4326	Cattle	Northeastern	0	0	0	0	0	0
4327	Cattle	Central	0	0	1	1	0	1
4328	Cattle	Central	0	0	0	1	0	1
4329	Cattle	Central	0	0	0	0	0	0
4330	Cattle	Central	0	0	0	0	0	0
4331	Cattle	Central	0	0	0	0	0	0
4332	Cattle	Central	0	0	1	1	0	1
4333	Cattle	Central	0	0	0	0	0	0
4334	Cattle	Central	0	0	1	1	0	1
4335	Cattle	Central	0	0	0	0	0	0
4336	Cattle	Central	0	0	0	1	0	1
4337	Cattle	Central	0	0	1	1	0	1
4338	Cattle	Central	0	0	0	0	0	0
4339	Cattle	Central	0	0	0	0	0	0
4340	Cattle	Central	0	0	0	0	0	0
4341	Cattle	Central	0	0	0	0	0	0
4342	Cattle	Central	0	0	0	0	0	0
4343	Cattle	Central	0	0	0	0	0	0
4344	Cattle	Central	0	0	0	0	0	0
4345	Cattle	Central	0	0	0	0	0	0
4346	Cattle	Central	0	0	0	0	0	0
4347	Cattle	Central	0	0	0	0	0	0
4348	Cattle	Central	0	0	0	0	0	0
4349	Cattle	Central	0	0	0	0	0	0
4350	Cattle	Central	0	0	0	0	0	0
4351	Cattle	Central	0	0	0	0	0	0
4352	Cattle	Central	0	0	0	0	0	0
4353	Cattle	Central	0	0	0	0	0	0
4354	Cattle	Central	0	0	0	0	0	0
4355	Cattle	Central	0	0	0	0	0	0
4356	Cattle	Central	0	0	0	0	0	0
4357	Cattle	Central	0	0	0	0	0	0
4358	Cattle	Central	0	0	0	0	0	0
4359	Cattle	Central	0	0	0	0	0	0
4360	Cattle	Eastern	0	0	1	1	0	1
4361	Cattle	Eastern	0	0	1	1	0	1
4362	Cattle	Eastern	0	0	1	1	0	1
4363	Cattle	Eastern	0	0	1	1	0	1
4364	Cattle	Central	0	0	1	0	0	1
4365	Cattle	Central	0	0	0	0	0	0
4366	Cattle	Central	0	0	0	1	0	1
4367	Cattle	Central	0	0	0	0	0	0

4368	Cattle	Central	0	0	0	1	0	1
4369	Cattle	Central	0	0	0	1	0	1
4370	Cattle	Central	0	0	1	1	0	1
4371	Cattle	Central	0	0	1	0	0	1
4372	Cattle	Central	0	0	0	0	0	0
4373	Cattle	Central	0	0	0	0	0	0
4374	Cattle	Central	0	0	0	1	0	1
4375	Cattle	Central	0	0	0	0	0	0
4376	Cattle	Central	0	0	1	1	0	1
4377	Cattle	Central	0	0	0	0	0	0
4378	Cattle	Central	0	0	0	0	0	0
4379	Cattle	Central	0	0	0	1	0	1
4380	Cattle	Central	0	0	1	1	0	1
4381	Cattle	Northeastern	0	0	0	0	0	0
4382	Cattle	Northeastern	0	0	0	0	0	0
4383	Cattle	Northeastern	0	0	0	0	0	0
4384	Cattle	Northeastern	0	0	0	0	0	0
4385	Cattle	Northeastern	0	0	0	0	0	0
4386	Cattle	Northeastern	0	0	0	0	0	0
4387	Cattle	Northeastern	0	0	0	0	0	0
4388	Cattle	Northeastern	0	0	0	0	0	0
4389	Cattle	Northeastern	0	1	0	0	0	1
4390	Cattle	Northeastern	0	0	0	0	0	0
4391	Cattle	Northeastern	0	0	0	0	0	0
4392	Cattle	Northeastern	0	0	0	0	0	0
4393	Cattle	Northeastern	0	0	0	0	0	0
4394	Cattle	Northeastern	0	0	0	0	0	0
4395	Cattle	Northeastern	0	0	0	0	0	0
4396	Cattle	Northeastern	0	0	0	0	0	0
4397	Cattle	Northeastern	0	0	0	0	0	0
4398	Cattle	Northeastern	0	0	0	0	0	0
4399	Cattle	Northeastern	0	0	0	0	0	0
4400	Cattle	Northeastern	0	0	0	0	0	0
4401	Cattle	Northeastern	0	0	0	0	0	0
4402	Cattle	Northeastern	0	0	0	0	0	0
4403	Cattle	Northeastern	0	0	0	0	0	0
4404	Cattle	Northeastern	0	0	0	0	0	0
4405	Cattle	Northeastern	0	0	0	0	0	0
4406	Cattle	Northeastern	0	0	0	0	0	0
4407	Cattle	Northeastern	0	0	0	0	0	0
4408	Cattle	Northeastern	0	0	0	0	0	0
4409	Cattle	Northeastern	0	0	0	0	0	0
4410	Cattle	Northeastern	0	0	0	0	0	0
4411	Cattle	Northeastern	0	0	0	0	0	0
4412	Cattle	Northeastern	0	0	0	0	0	0
4413	Cattle	Northeastern	0	0	0	0	0	0
4414	Cattle	Northeastern	0	0	0	0	0	0
4415	Cattle	Northeastern	0	0	0	0	0	0

4416	Cattle	Northeastern	0	0	0	0	0	0
4417	Cattle	Northeastern	0	0	0	0	0	0
4418	Cattle	Northeastern	0	0	0	0	0	0
4419	Cattle	Northeastern	0	0	0	0	0	0
4420	Cattle	Northeastern	0	1	0	0	0	1
4421	Cattle	Northeastern	0	0	0	0	0	0
4422	Cattle	Northeastern	0	0	0	0	0	0
4423	Cattle	Northeastern	0	0	0	0	0	0
4424	Cattle	Northeastern	0	0	0	0	0	0
4425	Cattle	Northeastern	0	0	0	0	0	0
4426	Cattle	Northeastern	0	0	0	0	0	0
4427	Cattle	Northeastern	0	0	0	0	0	0
4428	Cattle	Northeastern	0	0	0	0	0	0
4429	Cattle	Northeastern	0	0	0	0	0	0
4430	Cattle	Northeastern	0	0	0	0	0	0
4431	Cattle	Northeastern	0	0	0	0	0	0
4432	Cattle	Northeastern	0	0	0	0	0	0
4433	Cattle	Northeastern	0	0	0	0	0	0
4434	Cattle	Northeastern	0	0	0	0	0	0
4435	Cattle	Northeastern	0	0	0	0	0	0
4436	Cattle	Northeastern	0	0	0	0	0	0
4437	Cattle	Northeastern	0	0	0	0	0	0
4438	Cattle	Northeastern	0	0	0	0	0	0
4439	Cattle	Northeastern	0	0	0	0	0	0
4440	Cattle	Northeastern	0	0	0	0	0	0
4441	Cattle	Northeastern	0	0	0	0	0	0
4442	Cattle	Northeastern	0	0	0	0	0	0
4443	Cattle	Northeastern	0	0	0	0	0	0
4444	Cattle	Northeastern	0	0	0	0	0	0
4445	Cattle	Northeastern	0	0	0	0	0	0
4446	Cattle	Northeastern	0	0	0	0	0	0
4447	Cattle	Northeastern	0	0	0	0	0	0
4448	Cattle	Northeastern	0	0	0	0	0	0
4449	Cattle	Northeastern	0	0	0	0	0	0
4450	Cattle	Northeastern	0	0	0	1	0	1
4451	Cattle	Northeastern	0	0	1	1	0	1
4452	Cattle	Northeastern	0	0	0	0	0	0
4453	Cattle	Northeastern	0	0	0	0	0	0
4454	Cattle	Northeastern	0	1	0	0	0	1
4455	Cattle	Northeastern	0	0	0	0	0	0
4456	Cattle	Northeastern	0	0	0	0	0	0
4457	Cattle	Northeastern	0	0	0	0	0	0
4458	Cattle	Northeastern	0	0	0	0	0	0
4459	Cattle	Northeastern	0	0	0	0	0	0
4460	Cattle	Northeastern	0	0	0	0	0	0
4461	Cattle	Northeastern	0	0	0	0	0	0
4462	Cattle	Northeastern	0	0	0	0	0	0
4463	Cattle	Northeastern	0	0	0	0	0	0

4464	Cattle	Northeastern	0	0	0	0	0	0
4465	Cattle	Northeastern	0	1	0	0	0	1
4466	Cattle	Northeastern	0	0	0	0	0	0
4467	Cattle	Northeastern	0	0	0	0	0	0
4468	Cattle	Northeastern	0	0	0	0	0	0
4469	Cattle	Northeastern	0	0	0	0	0	0
4470	Cattle	Northeastern	0	0	0	0	0	0
4471	Cattle	Northeastern	0	0	0	0	0	0
4472	Cattle	Northeastern	0	0	0	0	0	0
4473	Cattle	Northeastern	0	0	0	0	0	0
4474	Cattle	Northeastern	0	0	0	0	0	0
4475	Cattle	Northeastern	0	0	0	0	0	0
4476	Cattle	Northeastern	0	0	0	0	0	0
4477	Cattle	Northeastern	0	0	0	0	0	0
4478	Cattle	Northeastern	0	0	0	0	0	0
4479	Cattle	Northeastern	0	0	0	0	0	0
4480	Cattle	Northeastern	0	0	0	0	0	0
4481	Cattle	Northeastern	0	0	0	0	0	0
4482	Cattle	Northeastern	0	0	0	0	0	0
4483	Cattle	Northeastern	0	0	0	0	0	0
4484	Cattle	Northeastern	0	0	0	0	0	0
4485	Cattle	Northeastern	0	0	0	0	0	0
4486	Cattle	Northeastern	0	0	0	0	0	0
4487	Cattle	Northeastern	0	0	0	0	0	0
4488	Cattle	Northeastern	0	0	0	0	0	0
4489	Cattle	Northeastern	0	0	0	0	0	0
4490	Cattle	Northeastern	0	0	0	0	0	0
4491	Cattle	Northeastern	0	0	0	0	0	0
4492	Cattle	Northeastern	0	0	0	0	0	0
4493	Cattle	Northeastern	0	0	0	0	0	0
4494	Cattle	Northeastern	0	0	0	0	0	0
4495	Cattle	Northeastern	0	0	0	0	0	0
4496	Cattle	Northeastern	0	0	0	0	0	0
4497	Cattle	Northeastern	0	0	0	0	0	0
4498	Cattle	Northeastern	0	0	0	0	0	0
4499	Cattle	Northeastern	0	0	0	0	0	0
4500	Cattle	Northeastern	0	1	0	0	0	1
4501	Cattle	Northeastern	0	0	0	0	0	0
4502	Cattle	Northeastern	0	0	0	0	0	0
4503	Cattle	Northeastern	0	0	0	0	0	0
4504	Cattle	Northeastern	0	0	0	0	0	0
4505	Cattle	Northeastern	0	0	0	0	0	0
4506	Cattle	Northeastern	0	0	0	0	0	0
4507	Cattle	Northeastern	0	0	0	0	0	0
4508	Cattle	Northeastern	0	0	0	0	0	0
4509	Cattle	Northeastern	0	0	0	0	0	0
4510	Cattle	Northeastern	0	0	0	0	0	0
4511	Cattle	Southern	0	0	0	0	0	0

4512	Cattle	Southern	0	0	0	0	0	0
4513	Cattle	Southern	0	0	0	0	0	0
4514	Cattle	Southern	0	0	0	0	0	0
4515	Cattle	Southern	0	0	0	0	0	0
4516	Cattle	Southern	0	0	0	0	0	0
4517	Cattle	Southern	0	0	0	0	0	0
4518	Cattle	Eastern	0	0	0	0	0	0
4519	Cattle	Eastern	0	0	0	0	0	0
4520	Cattle	Eastern	0	0	0	0	0	0
4521	Cattle	Eastern	0	0	0	0	0	0
4522	Cattle	Eastern	0	0	0	0	0	0
4523	Cattle	Eastern	0	0	0	0	0	0
4524	Cattle	Eastern	0	0	0	0	0	0
4525	Cattle	Eastern	0	0	0	0	0	0
4526	Cattle	Eastern	0	0	0	0	0	0
4527	Cattle	Eastern	0	0	0	0	0	0
4528	Cattle	Central	0	0	0	0	0	0
4529	Cattle	Central	0	0	0	0	0	0
4530	Cattle	Central	0	0	0	0	0	0
4531	Cattle	Central	0	0	0	0	0	0
4532	Cattle	Central	0	0	0	0	0	0
4533	Cattle	Central	0	0	0	0	0	0
4534	Cattle	Central	0	0	0	0	0	0
4535	Cattle	Central	0	0	0	0	0	0
4536	Cattle	Central	0	0	0	0	0	0
4537	Cattle	Central	0	0	0	0	0	0
4538	Cattle	Central	0	0	0	0	0	0
4539	Cattle	Central	0	0	0	0	0	0
4540	Cattle	Central	0	0	0	0	0	0
4541	Cattle	Central	0	0	0	0	0	0
4542	Cattle	Central	0	0	0	0	0	0
4543	Cattle	Central	0	0	0	0	0	0
4544	Cattle	Central	0	0	1	1	0	1
4545	Cattle	Central	0	0	0	0	0	0
4546	Cattle	Central	0	0	0	1	0	1
4547	Cattle	Central	0	0	1	1	0	1
4548	Cattle	Central	0	0	0	0	0	0
4549	Cattle	Central	0	0	0	0	0	0
4550	Cattle	Central	0	0	1	1	0	1
4551	Cattle	Central	0	0	0	0	0	0
4552	Cattle	Central	0	0	0	1	0	1
4553	Cattle	Central	0	0	1	1	0	1
4554	Cattle	Eastern	0	0	0	0	0	0
4555	Cattle	Eastern	0	0	0	0	0	0
4556	Cattle	Eastern	0	0	0	0	0	0
4557	Cattle	Eastern	0	0	0	0	0	0
4558	Cattle	Eastern	0	0	0	0	0	0
4559	Cattle	Eastern	0	0	1	1	0	1

4560	Cattle	Eastern	0	0	1	1	0	1
4561	Cattle	Eastern	0	0	0	0	0	0
4562	Cattle	Eastern	0	0	0	0	0	0
4563	Cattle	Eastern	0	0	0	0	0	0
4564	Cattle	Eastern	0	0	0	0	0	0
4565	Cattle	Eastern	0	0	1	0	0	1
4566	Cattle	Eastern	0	0	0	0	0	0
4567	Cattle	Eastern	0	0	1	1	0	1
4568	Cattle	Eastern	0	0	1	0	0	1
4569	Cattle	Eastern	0	0	1	0	0	1
4570	Cattle	Eastern	0	0	1	1	0	1
4571	Cattle	Eastern	0	0	1	0	0	1
4572	Cattle	Eastern	0	0	1	0	0	1
4573	Cattle	Eastern	0	0	0	0	0	0
4574	Cattle	Eastern	0	0	0	0	0	0
4575	Cattle	Eastern	0	1	1	1	0	1
4576	Cattle	Eastern	0	0	0	0	0	0
4577	Cattle	Eastern	0	0	0	0	0	0
4578	Cattle	Eastern	0	0	0	0	0	0
4579	Cattle	Eastern	0	0	0	0	0	0
4580	Cattle	Eastern	0	0	0	0	0	0
4581	Cattle	Eastern	0	0	0	0	0	0
4582	Cattle	Eastern	0	0	0	0	0	0
4583	Cattle	Northeastern	0	1	0	0	0	1
4584	Cattle	Northeastern	0	1	0	0	0	1
4585	Cattle	Northeastern	0	1	0	0	0	1
4586	Cattle	Northeastern	0	1	0	0	0	1
4587	Cattle	Northeastern	0	1	0	0	0	1
4588	Cattle	Northeastern	0	0	1	1	0	1
4589	Cattle	Northeastern	0	0	0	1	0	1
4590	Cattle	Northeastern	0	1	0	0	0	1
4591	Cattle	Northeastern	0	1	0	0	0	1
4592	Cattle	Northeastern	0	1	0	0	0	1
4593	Cattle	Northeastern	0	1	0	0	0	1
4594	Cattle	Northeastern	0	1	0	0	0	1
4595	Cattle	Northeastern	0	0	0	0	0	0
4596	Cattle	Northeastern	0	0	0	0	0	0
4597	Cattle	Southern	0	0	0	0	0	0
4598	Cattle	Southern	0	0	1	1	0	1
4599	Cattle	Southern	0	0	0	0	0	0
4600	Cattle	Southern	0	0	0	0	0	0
4601	Cattle	Southern	0	0	0	0	0	0
4602	Cattle	Southern	0	0	0	0	0	0
4603	Cattle	Southern	0	0	0	0	0	0
4604	Cattle	Southern	0	0	0	1	0	1
4605	Cattle	Southern	0	0	0	0	0	1
4606	Cattle	Southern	0	0	0	0	0	0
4607	Cattle	Central	0	0	0	0	0	0

4608	Cattle	Central	0	0	0	0	0	0
4609	Cattle	Central	0	0	0	0	0	0
4610	Cattle	Central	0	0	0	1	1	1
4611	Cattle	Central	0	0	0	0	0	0
4612	Cattle	Central	0	0	0	0	0	0
4613	Cattle	Central	0	0	0	1	0	1
4614	Cattle	Central	0	0	0	0	0	0
4615	Cattle	Central	0	0	0	0	0	0
4616	Cattle	Central	0	0	0	0	0	0
4617	Cattle	Central	0	0	1	1	0	1
4618	Cattle	Central	0	0	0	0	0	0
4619	Cattle	Central	0	0	0	0	0	0
4620	Cattle	Central	0	0	1	1	0	1
4621	Cattle	Central	0	0	0	1	0	1
4622	Cattle	Central	0	0	0	0	0	0
4623	Cattle	Central	0	0	0	1	0	1
4624	Cattle	Central	0	0	1	1	0	1
4625	Cattle	Central	0	0	0	1	1	1
4626	Cattle	Central	0	0	1	1	0	1
4627	Cattle	Central	0	0	0	0	0	0
4628	Cattle	Central	0	0	0	0	0	0
4629	Cattle	Central	0	0	0	0	0	0
4630	Cattle	Central	0	0	0	0	0	0
4631	Cattle	Central	0	0	0	0	0	0
4632	Cattle	Central	0	0	0	0	0	0
4633	Cattle	Central	0	0	0	0	0	0
4634	Cattle	Central	0	0	0	1	0	1
4635	Cattle	Central	0	0	0	0	0	0
4636	Cattle	Central	0	0	0	0	0	0
4637	Cattle	Central	0	0	0	0	0	0
4638	Cattle	Central	0	0	0	0	0	0
4639	Cattle	Central	0	0	0	0	0	0
4640	Cattle	Central	0	0	1	1	0	1
4641	Cattle	Central	0	0	1	1	0	1
4642	Cattle	Central	0	0	1	1	0	1
4643	Cattle	Central	0	0	1	1	0	1
4644	Cattle	Central	0	0	0	0	0	0
4645	Cattle	Central	0	0	1	1	0	1
4646	Cattle	Central	0	0	0	1	0	1
4647	Cattle	Northeastern	0	0	0	0	0	0
4648	Cattle	Northeastern	0	0	0	1	0	1
4649	Cattle	Northeastern	0	0	0	1	0	1
4650	Cattle	Northeastern	0	0	0	0	0	0
4651	Cattle	Northeastern	0	0	0	0	0	0
4652	Cattle	Northeastern	0	0	0	1	0	1
4653	Cattle	Northeastern	0	0	1	1	0	1
4654	Cattle	Northeastern	0	0	0	0	0	0
4655	Cattle	Northeastern	0	0	0	1	0	1

4656	Cattle	Northeastern	0	0	0	1	0	1
4657	Cattle	Northeastern	0	0	0	0	0	0
4658	Cattle	Northeastern	0	0	0	0	0	0
4659	Cattle	Northeastern	0	0	1	1	0	1
4660	Cattle	Northeastern	0	0	0	1	0	1
4661	Cattle	Northeastern	0	0	0	0	0	0
4662	Cattle	Northeastern	0	0	0	0	0	0
4663	Cattle	Northeastern	0	0	0	0	0	0
4664	Cattle	Northeastern	0	0	0	1	0	1
4665	Cattle	Northeastern	0	0	0	1	0	1
4666	Cattle	Northeastern	0	0	0	1	0	1
4667	Cattle	Northeastern	0	0	0	0	0	0
4668	Cattle	Northeastern	0	0	0	1	0	1
4669	Cattle	Northeastern	0	0	1	1	0	1
4670	Cattle	Northeastern	0	0	0	0	0	0
4671	Cattle	Northeastern	0	0	1	1	0	1
4672	Cattle	Northeastern	0	0	1	1	0	1
4673	Cattle	Northeastern	0	0	0	1	0	1
4674	Cattle	Northeastern	0	0	1	1	0	1
4675	Cattle	Northeastern	0	0	1	1	0	1
4676	Cattle	Northeastern	0	1	0	1	0	1
4677	Cattle	Northeastern	0	0	0	0	0	0
4678	Cattle	Central	0	0	1	1	0	1
4679	Cattle	Central	0	0	1	1	0	1
4680	Cattle	Central	0	0	1	1	0	1
4681	Cattle	Central	0	0	1	1	0	1
4682	Cattle	Central	0	0	0	0	0	0
4683	Cattle	Central	0	0	1	0	0	1
4684	Cattle	Central	0	0	0	1	0	1
4685	Cattle	Central	0	0	1	1	1	1
4686	Cattle	Central	0	0	1	1	0	1
4687	Cattle	Central	0	0	1	0	0	1
4688	Cattle	Central	0	0	1	1	0	1
4689	Cattle	Central	0	0	1	1	0	1
4690	Cattle	Central	0	0	1	0	0	1
4691	Cattle	Central	0	0	0	1	0	1
4692	Cattle	Central	0	0	0	0	0	0
4693	Cattle	Central	0	0	0	0	0	0
4694	Cattle	Central	0	0	0	1	0	1
4695	Cattle	Central	0	0	1	1	0	1
4696	Cattle	Central	0	0	1	1	0	1
4697	Cattle	Central	0	0	0	0	0	0
4698	Cattle	Central	0	0	0	0	0	0
4699	Cattle	Central	0	0	1	0	0	1
4700	Cattle	Central	0	0	1	1	0	1
4701	Cattle	Central	0	0	0	0	0	0
4702	Cattle	Central	0	0	1	0	1	1
4703	Cattle	Central	0	0	1	1	0	1

4704	Cattle	Central	0	0	0	0	0	0
4705	Cattle	Central	0	0	0	0	0	0
4706	Cattle	Central	0	0	0	0	1	1
4707	Cattle	Central	0	0	0	0	0	0
4708	Cattle	Central	0	0	0	0	0	0
4709	Cattle	Central	0	0	1	1	0	1
4710	Cattle	Central	0	0	1	0	0	1
4711	Cattle	Central	0	0	1	0	0	1
4712	Cattle	Central	0	0	1	0	0	1
4713	Cattle	Central	0	0	0	0	0	0
4714	Cattle	Central	0	0	0	0	0	0
4715	Cattle	Central	0	0	1	1	0	1
4716	Cattle	Central	0	0	1	1	0	1
4717	Cattle	Central	0	0	0	0	0	0
4718	Cattle	Central	0	0	1	0	0	1
4719	Cattle	Central	0	0	1	1	0	1
4720	Cattle	Central	0	0	0	0	0	0
4721	Cattle	Central	0	0	1	0	0	1
4722	Cattle	Central	0	0	1	1	0	1
4723	Cattle	Central	0	0	1	1	0	1
4724	Cattle	Central	0	0	1	1	0	1
4725	Cattle	Central	0	1	1	1	0	1
4726	Cattle	Central	0	0	1	1	0	1
4727	Cattle	Central	0	0	1	0	0	1
4728	Cattle	Eastern	0	0	1	1	0	1
4729	Cattle	Eastern	0	0	1	1	0	1
4730	Cattle	Eastern	0	0	1	1	0	1
4731	Cattle	Southern	0	0	0	0	0	0
4732	Cattle	Southern	0	0	0	0	0	0
4733	Cattle	Southern	0	0	1	1	0	1
4734	Cattle	Southern	0	0	0	0	0	0
4735	Cattle	Southern	0	0	0	0	0	0
4736	Cattle	Southern	0	0	1	1	0	1
4737	Cattle	Southern	0	0	1	1	0	1
4738	Cattle	Southern	0	0	0	0	0	0
4739	Cattle	Eastern	0	0	1	1	0	1
4740	Cattle	Eastern	0	0	1	1	0	1
4741	Cattle	Eastern	0	0	1	1	0	1
4742	Cattle	Eastern	0	0	1	1	0	1
4743	Cattle	Eastern	0	0	1	1	0	1
4744	Cattle	Northeastern	0	0	0	0	0	1
4745	Cattle	Northeastern	0	0	1	0	0	1
4746	Cattle	Northeastern	0	0	0	0	0	1
4747	Cattle	Northeastern	0	0	1	0	0	1
4748	Cattle	Northern	0	0	0	0	0	0
4749	Cattle	Northern	0	0	0	0	0	0
4750	Cattle	Northern	0	0	1	0	0	1
4751	Cattle	Northern	0	0	0	0	0	0

4752	Cattle	Northern	0	0	1	1	0	1
4753	Cattle	Northern	0	0	0	0	0	0
4754	Cattle	Northern	0	0	1	0	0	1
4755	Cattle	Northern	0	0	1	0	0	1
4756	Cattle	Northern	0	0	0	0	0	0
4757	Cattle	Northern	0	0	1	0	0	1
4758	Cattle	Northern	0	0	1	0	0	1
4759	Cattle	Northern	0	0	1	0	0	1
4760	Cattle	Northern	0	0	0	0	0	0
4761	Cattle	Northern	0	0	1	1	0	1
4762	Cattle	Northern	0	0	0	0	0	0
4763	Cattle	Northern	0	0	1	1	0	1
4764	Cattle	Northern	0	0	1	1	0	1
4765	Cattle	Eastern	0	0	0	0	0	0
4766	Cattle	Eastern	0	0	0	0	0	0
4767	Cattle	Eastern	0	0	0	0	0	0
4768	Cattle	Eastern	0	0	0	0	0	0
4769	Cattle	Eastern	0	0	1	1	0	1
4770	Cattle	Eastern	0	0	0	0	0	0
4771	Cattle	Eastern	0	0	0	0	0	0
4772	Cattle	Eastern	0	0	0	0	0	0
4773	Cattle	Eastern	0	0	0	0	0	0
4774	Cattle	Eastern	0	0	0	0	0	0
4775	Cattle	Eastern	0	0	1	1	0	1
4776	Cattle	Eastern	0	0	0	0	0	0
4777	Cattle	Eastern	0	0	0	0	0	0
4778	Cattle	Eastern	0	0	1	1	0	1
4779	Cattle	Eastern	0	0	0	0	0	0
4780	Cattle	Eastern	0	0	0	0	0	0
4781	Cattle	Eastern	0	0	0	0	0	0
4782	Cattle	Eastern	0	0	0	0	0	0
4783	Cattle	Eastern	0	0	1	1	0	1
4784	Cattle	Eastern	0	0	0	0	0	0
4785	Cattle	Eastern	0	0	0	0	0	0
4786	Cattle	Eastern	0	0	0	0	0	0
4787	Cattle	Eastern	0	0	0	0	0	0
4788	Cattle	Eastern	0	0	0	0	0	0
4789	Cattle	Eastern	0	0	0	0	0	0
4790	Cattle	Eastern	0	0	0	0	0	0
4791	Cattle	Eastern	0	0	0	0	0	0
4792	Cattle	Eastern	0	0	0	0	0	0
4793	Cattle	Eastern	0	0	0	0	0	0
4794	Cattle	Northeastern	0	0	0	0	0	0
4795	Cattle	Northeastern	0	0	0	0	0	0
4796	Cattle	Northeastern	0	0	0	0	0	0
4797	Cattle	Northeastern	0	0	0	1	0	1
4798	Cattle	Northeastern	0	0	0	1	0	1
4799	Cattle	Northeastern	0	0	0	0	0	0

4800	Cattle	Northeastern	0	0	0	0	0	0
4801	Cattle	Northeastern	0	0	0	0	0	0
4802	Cattle	Northeastern	0	0	0	0	0	0
4803	Cattle	Northeastern	0	0	0	0	0	0
4804	Cattle	Northeastern	0	0	0	1	0	1
4805	Cattle	Northeastern	0	0	0	1	0	1
4806	Cattle	Northeastern	0	0	0	0	0	0
4807	Cattle	Northeastern	0	0	0	0	0	0
4808	Cattle	Northeastern	0	0	0	0	0	0
4809	Cattle	Northeastern	0	0	0	0	0	0
4810	Cattle	Eastern	0	0	0	1	0	1
4811	Cattle	Eastern	0	0	0	0	0	1
4812	Cattle	Eastern	0	0	0	0	0	1
4813	Cattle	Eastern	0	0	0	0	0	0
4814	Cattle	Eastern	0	0	0	0	0	1
4815	Cattle	Eastern	0	0	0	0	0	1
4816	Cattle	Eastern	0	0	0	0	0	0
4817	Cattle	Eastern	0	0	1	0	0	1
4818	Cattle	Eastern	0	0	0	0	0	0
4819	Cattle	Eastern	0	0	0	0	0	0
4820	Cattle	Eastern	0	0	1	1	0	1
4821	Cattle	Eastern	0	0	0	0	0	0
4822	Cattle	Eastern	0	0	0	0	0	0
4823	Cattle	Eastern	0	0	0	0	0	0
4824	Cattle	Eastern	0	0	0	1	0	1
4825	Cattle	Eastern	0	0	0	0	0	0
4826	Cattle	Eastern	0	0	0	0	0	0
4827	Cattle	Eastern	0	0	0	0	0	1
4828	Cattle	Eastern	0	0	1	1	0	1
4829	Cattle	Eastern	0	0	0	0	0	0
4830	Cattle	Eastern	0	1	0	0	0	1
4831	Cattle	Eastern	0	0	0	0	0	0
4832	Cattle	Eastern	0	0	0	0	0	0
4833	Cattle	Eastern	0	0	0	0	0	0
4834	Cattle	Eastern	0	0	0	0	0	0
4835	Cattle	Eastern	0	0	0	0	0	0
4836	Cattle	Eastern	0	0	0	0	0	0
4837	Cattle	Eastern	0	0	0	0	0	1
4838	Cattle	Eastern	0	0	0	0	0	0
4839	Cattle	Eastern	0	0	0	0	0	0
4840	Cattle	Eastern	0	0	0	0	0	0
4841	Cattle	Eastern	0	1	1	1	0	1
4842	Cattle	Eastern	0	0	0	0	0	0
4843	Cattle	Eastern	0	0	0	0	0	0
4844	Cattle	Eastern	0	0	0	0	0	0
4845	Cattle	Eastern	0	0	0	0	0	0
4846	Cattle	Eastern	0	0	0	0	0	0
4847	Cattle	Eastern	0	0	0	0	0	0

4848	Cattle	Eastern	0	0	0	0	0	0
4849	Cattle	Eastern	0	0	1	1	0	1
4850	Cattle	Eastern	0	0	0	0	0	0
4851	Cattle	Eastern	0	0	0	0	0	0
4852	Cattle	Eastern	0	0	0	0	0	0
4853	Cattle	Eastern	0	0	0	0	0	0
4854	Cattle	Eastern	0	0	0	0	0	0
4855	Cattle	Eastern	0	0	0	0	0	0
4856	Cattle	Eastern	0	0	0	0	0	0
4857	Cattle	Eastern	0	0	0	0	0	0
4858	Cattle	Eastern	0	0	1	1	0	1
4859	Cattle	Eastern	0	0	0	0	0	0
4860	Cattle	Eastern	0	1	0	0	0	1
4861	Cattle	Eastern	0	0	1	1	0	1
4862	Cattle	Eastern	0	0	0	0	0	0
4863	Cattle	Eastern	0	0	0	0	0	0
4864	Cattle	Eastern	0	0	1	0	0	1
4865	Cattle	Eastern	0	0	1	1	0	1
4866	Cattle	Eastern	0	0	0	0	0	0
4867	Cattle	Eastern	0	0	0	0	0	0
4868	Cattle	Eastern	0	0	1	0	0	1
4869	Cattle	Eastern	0	0	1	0	0	1
4870	Cattle	Eastern	0	0	1	0	0	1
4871	Cattle	Eastern	0	0	0	1	0	1
4872	Cattle	Eastern	0	0	0	0	0	0
4873	Cattle	Eastern	0	0	1	0	0	1
4874	Cattle	Eastern	0	0	1	1	0	1
4875	Cattle	Eastern	0	0	1	1	0	1
4876	Cattle	Central	0	0	0	0	0	0
4877	Cattle	Central	0	0	0	0	0	0
4878	Cattle	Northeastern	0	0	1	0	0	1
4879	Cattle	Northeastern	0	0	1	0	0	1
4880	Cattle	Northeastern	0	0	1	1	0	1
4881	Cattle	Northeastern	0	0	1	0	0	1
4882	Cattle	Northeastern	0	0	0	0	0	0
4883	Cattle	Northeastern	0	0	1	1	0	1
4884	Cattle	Northeastern	0	0	1	0	0	1
4885	Cattle	Northeastern	0	0	1	1	0	1
4886	Cattle	Northeastern	0	0	1	0	0	1
4887	Cattle	Northeastern	0	0	1	0	0	1
4888	Cattle	Central	0	0	1	0	0	1
4889	Cattle	Central	0	0	1	0	0	1
4890	Cattle	Central	0	0	1	1	0	1
4891	Cattle	Central	0	0	1	0	0	1
4892	Cattle	Central	0	0	1	1	0	1
4893	Cattle	Central	0	0	0	0	0	0
4894	Cattle	Northeastern	0	0	1	0	0	1
4895	Cattle	Northeastern	0	0	0	0	0	0

4896	Cattle	Northeastern	0	0	1	1	0	1
4897	Cattle	Northeastern	0	0	1	0	0	1
4898	Cattle	Northeastern	0	0	1	0	0	1
4899	Cattle	Northeastern	0	0	0	1	0	1
4900	Cattle	Northeastern	0	0	0	0	0	0
4901	Cattle	Northeastern	0	0	0	0	0	0
4902	Cattle	Northeastern	0	0	1	1	0	1
4903	Cattle	Northeastern	0	0	1	1	0	1
4904	Cattle	Northeastern	0	0	0	0	0	0
4905	Cattle	Northeastern	0	0	0	0	0	0
4906	Cattle	Northeastern	0	0	1	0	0	1
4907	Cattle	Northeastern	0	0	1	0	0	1
4908	Cattle	Northeastern	0	0	1	0	0	1
4909	Cattle	Northeastern	0	0	1	0	0	1
4910	Cattle	Northeastern	0	0	1	1	0	1
4911	Cattle	Northeastern	0	0	1	1	0	1
4912	Cattle	Northeastern	0	0	1	1	0	1
4913	Cattle	Northeastern	0	0	1	0	0	1
4914	Cattle	Northeastern	0	0	0	0	0	0
4915	Cattle	Northeastern	0	0	1	0	0	1
4916	Cattle	Northeastern	0	0	1	1	0	1
4917	Cattle	Northeastern	0	0	1	1	0	1
4918	Cattle	Northeastern	0	0	1	0	0	1
4919	Cattle	Northeastern	0	0	0	0	0	0
4920	Cattle	Northeastern	0	0	1	0	0	1
4921	Cattle	Northeastern	0	0	1	0	0	1
4922	Cattle	Northeastern	0	0	1	0	0	1
4923	Cattle	Northeastern	0	0	1	0	0	1
4924	Cattle	Northeastern	0	0	1	0	0	1
4925	Cattle	Central	0	0	1	1	0	1
4926	Cattle	Central	0	0	1	1	0	1
4927	Cattle	Central	0	0	1	0	0	1
4928	Cattle	Central	0	0	0	0	0	0
4929	Cattle	Central	0	0	1	1	0	1
4930	Cattle	Central	0	0	1	1	0	1
4931	Cattle	Central	0	0	1	0	0	1
4932	Cattle	Central	0	0	1	1	0	1
4933	Cattle	Central	0	0	1	1	0	1
4934	Cattle	Central	0	0	1	0	0	1
4935	Cattle	Central	0	0	1	1	0	1
4936	Cattle	Central	0	0	1	1	0	1
4937	Cattle	Central	0	0	1	0	0	1
4938	Cattle	Central	0	0	1	1	0	1
4939	Cattle	Central	0	0	1	0	0	1
4940	Cattle	Central	0	0	1	0	0	1
4941	Cattle	Central	0	0	1	0	0	1
4942	Cattle	Central	0	0	1	0	0	1
4943	Cattle	Central	0	0	1	0	0	1

4944	Cattle	Central	0	0	1	1	0	1
4945	Cattle	Central	0	0	0	0	0	0
4946	Cattle	Central	0	0	1	1	0	1
4947	Cattle	Central	0	0	1	1	0	1
4948	Cattle	Central	0	0	0	0	0	0
4949	Cattle	Central	0	0	1	1	0	1
4950	Cattle	Central	0	0	0	0	0	0
4951	Cattle	Central	0	0	1	1	0	1
4952	Cattle	Central	0	0	1	1	0	1
4953	Cattle	Central	0	0	1	1	0	1
4954	Cattle	Central	0	0	0	0	0	0
4955	Cattle	Central	0	0	1	0	0	1
4956	Cattle	Central	0	0	1	1	0	1
4957	Cattle	Central	0	0	1	1	0	1
4958	Cattle	Eastern	0	0	1	1	0	1
4959	Cattle	Eastern	0	0	0	0	0	0
4960	Cattle	Eastern	0	0	0	0	0	0
4961	Cattle	Northeastern	0	0	0	0	0	0
4962	Cattle	Northeastern	0	0	1	0	0	1
4963	Cattle	Central	0	0	1	1	0	1
4964	Cattle	Central	0	0	0	0	0	0
4965	Cattle	Central	0	0	1	0	0	1
4966	Cattle	Central	0	0	1	1	0	1
4967	Cattle	Central	0	0	1	1	0	1
4968	Cattle	Central	0	0	1	1	0	1
4969	Cattle	Central	0	0	0	0	0	0
4970	Cattle	Central	0	0	1	1	0	1
4971	Cattle	Central	0	0	0	1	0	1
4972	Cattle	Central	0	0	1	1	0	1
4973	Cattle	Central	0	0	1	0	0	1
4974	Cattle	Central	0	0	1	0	0	1
4975	Cattle	Central	0	0	1	1	0	1
4976	Cattle	Central	0	0	1	0	0	1
4977	Cattle	Central	0	0	1	0	0	1
4978	Cattle	Central	0	0	1	0	0	1
4979	Cattle	Central	0	0	1	1	0	1
4980	Cattle	Central	0	0	1	1	0	1
4981	Cattle	Central	0	0	1	1	0	1
4982	Cattle	Central	0	0	1	1	0	1
4983	Cattle	Central	0	0	1	0	0	1
4984	Cattle	Central	0	0	1	0	0	1
4985	Cattle	Central	0	0	1	0	0	1
4986	Cattle	Central	0	0	1	0	0	1
4987	Cattle	Central	0	0	1	1	1	1
4988	Cattle	Central	0	0	1	0	0	1
4989	Cattle	Central	0	0	1	0	0	1
4990	Cattle	Central	0	0	1	1	0	1
4991	Cattle	Central	0	0	1	1	0	1

4992	Cattle	Central	0	0	1	1	1	1
4993	Cattle	Central	0	0	1	0	0	1
4994	Cattle	Central	0	0	0	0	0	0
4995	Cattle	Central	0	0	1	0	0	1
4996	Cattle	Central	0	0	0	0	0	0
4997	Cattle	Central	0	0	0	0	0	0
4998	Cattle	Central	0	0	1	0	0	1
4999	Cattle	Central	0	0	0	0	0	0
5000	Cattle	Central	0	0	1	0	0	1
5001	Cattle	Central	0	0	0	0	0	0
5002	Cattle	Central	0	0	0	0	0	0
5003	Cattle	Central	0	0	0	0	0	0
5004	Cattle	Central	0	0	0	0	0	0
5005	Cattle	Central	0	0	0	0	0	0
5006	Cattle	Central	0	0	0	0	0	0
5007	Cattle	Central	0	0	0	0	0	0
5008	Cattle	Central	0	0	0	0	0	0
5009	Cattle	Central	0	0	0	0	0	0
5010	Cattle	Central	0	0	0	0	0	0
5011	Cattle	Central	0	0	0	0	0	0
5012	Cattle	Central	0	0	0	0	0	0
5013	Cattle	Central	0	0	0	0	0	0
5014	Cattle	Central	0	0	0	0	0	0
5015	Cattle	Central	0	0	0	0	0	0
5016	Cattle	Central	0	0	0	0	0	0
5017	Cattle	Central	0	0	0	0	0	0
5018	Cattle	Central	0	0	1	0	0	1
5019	Cattle	Central	0	0	0	0	0	0
5020	Cattle	Central	0	0	0	0	0	0
5021	Cattle	Central	0	0	0	0	0	0
5022	Cattle	Central	0	0	0	0	0	0
5023	Cattle	Central	0	0	0	0	0	0
5024	Cattle	Central	0	0	0	0	0	0
5025	Cattle	Central	0	0	0	0	0	0
5026	Cattle	Central	0	0	0	0	0	0
5027	Cattle	Central	0	0	0	0	0	0
5028	Cattle	Central	0	0	1	1	0	1
5029	Cattle	Central	0	0	1	0	0	1
5030	Cattle	Central	0	0	1	1	0	1
5031	Cattle	Central	0	0	1	0	0	1
5032	Cattle	Central	0	0	0	1	0	1
5033	Cattle	Central	0	0	1	0	0	1
5034	Cattle	Central	0	1	1	1	0	1
5035	Cattle	Central	0	0	0	0	0	0
5036	Cattle	Central	0	0	0	1	0	1
5037	Cattle	Central	0	0	0	0	0	0
5038	Cattle	Central	0	0	0	0	0	0
5039	Cattle	Northeastern	0	0	0	0	0	0

5040	Cattle	Northeastern	0	0	0	0	0	0
5041	Cattle	Northeastern	0	0	1	1	0	1
5042	Cattle	Northeastern	0	0	0	0	0	0
5043	Cattle	Northeastern	0	0	0	0	0	0
5044	Cattle	Northeastern	0	0	1	0	0	1
5045	Cattle	Northeastern	0	0	0	0	0	0
5046	Cattle	Northeastern	0	0	0	0	0	0
5047	Cattle	Northeastern	0	0	1	0	0	1
5048	Cattle	Northeastern	0	0	0	0	0	0
5049	Cattle	Northeastern	0	0	1	0	0	1
5050	Cattle	Northeastern	0	0	0	0	0	0
5051	Cattle	Northeastern	0	0	0	0	0	0
5052	Cattle	Northeastern	0	0	0	0	0	0
5053	Cattle	Northeastern	0	0	1	0	0	1
5054	Cattle	Northeastern	0	0	1	0	0	1
5055	Cattle	Northeastern	0	0	1	0	0	1
5056	Cattle	Northeastern	0	0	1	0	0	1
5057	Cattle	Northeastern	0	0	1	1	0	1
5058	Cattle	Northeastern	0	0	1	0	0	1
5059	Cattle	Northeastern	0	0	1	0	0	1
5060	Cattle	Northeastern	0	0	0	0	0	0
5061	Cattle	Northeastern	0	0	0	0	0	0
5062	Cattle	Northeastern	0	0	1	0	0	1
5063	Cattle	Northeastern	0	0	0	0	0	0
5064	Cattle	Northeastern	0	0	0	0	0	0
5065	Cattle	Northeastern	0	0	1	0	0	1
5066	Cattle	Northeastern	0	0	1	0	0	1
5067	Cattle	Northeastern	0	0	0	0	0	0
5068	Cattle	Northeastern	0	0	0	0	0	0
5069	Cattle	Northeastern	0	0	1	0	0	1
5070	Cattle	Northeastern	0	0	1	0	0	1
5071	Cattle	Northeastern	0	0	1	0	0	1
5072	Cattle	Northeastern	0	0	1	0	0	1
5073	Cattle	Northeastern	0	0	1	1	0	1
5074	Cattle	Northeastern	0	0	1	1	0	1
5075	Cattle	Northeastern	0	0	0	0	0	0
5076	Cattle	Northeastern	0	1	1	0	0	1
5077	Cattle	Northeastern	0	0	1	0	0	1
5078	Cattle	Northeastern	0	0	1	0	0	1
5079	Cattle	Northeastern	0	0	0	0	0	0
5080	Cattle	Northeastern	0	0	0	0	0	0
5081	Cattle	Northeastern	0	0	0	0	0	0
5082	Cattle	Northeastern	0	1	1	0	0	1
5083	Cattle	Northeastern	0	0	0	0	0	0
5084	Cattle	Northeastern	0	0	0	0	0	0
5085	Cattle	Northeastern	0	0	0	0	0	0
5086	Cattle	Northeastern	0	0	1	0	0	1
5087	Cattle	Northeastern	0	0	0	0	0	0

5088	Cattle	Northeastern	0	0	0	0	0	0
5089	Cattle	Northeastern	0	0	0	0	0	0
5090	Cattle	Northeastern	0	0	1	0	0	1
5091	Cattle	Northeastern	0	0	0	0	0	0
5092	Cattle	Northeastern	0	1	1	0	0	1
5093	Cattle	Northeastern	0	0	1	0	0	1
5094	Cattle	Northeastern	0	0	1	0	0	1
5095	Cattle	Northeastern	0	0	0	0	0	0
5096	Cattle	Northeastern	0	0	0	0	0	0
5097	Cattle	Northeastern	0	0	0	0	0	0
5098	Cattle	Northeastern	0	1	0	0	0	1
5099	Cattle	Northeastern	0	0	0	0	0	0
5100	Cattle	Northeastern	0	0	0	0	0	0
5101	Cattle	Northeastern	0	0	0	0	0	0
5102	Cattle	Northeastern	0	0	1	1	0	1
5103	Cattle	Northeastern	0	0	1	0	0	1
5104	Cattle	Northeastern	0	0	1	0	0	1
5105	Cattle	Northeastern	0	0	1	0	0	1
5106	Cattle	Northeastern	0	0	1	1	0	1
5107	Cattle	Northeastern	0	0	0	0	0	0
5108	Cattle	Northeastern	0	0	1	0	0	1
5109	Cattle	Northeastern	0	0	1	1	0	1
5110	Cattle	Northeastern	0	0	1	1	1	1
5111	Cattle	Northeastern	0	0	1	0	0	1
5112	Cattle	Northeastern	0	0	1	0	0	1
5113	Cattle	Northeastern	0	0	0	0	0	0
5114	Cattle	Northeastern	0	0	0	0	0	0
5115	Cattle	Northeastern	0	0	1	0	0	1
5116	Cattle	Northeastern	0	0	1	0	0	1
5117	Cattle	Northeastern	0	0	1	0	0	1
5118	Cattle	Northeastern	0	0	0	0	0	0
5119	Cattle	Northeastern	0	0	0	0	0	0
5120	Cattle	Northeastern	0	0	1	0	0	1
5121	Cattle	Northeastern	0	0	0	0	0	0
5122	Cattle	Northeastern	0	0	0	0	0	0
5123	Cattle	Northeastern	0	0	0	0	0	0
5124	Cattle	Northeastern	0	0	1	0	0	1
5125	Cattle	Northeastern	0	0	0	0	0	0
5126	Cattle	Northeastern	0	0	0	0	0	0
5127	Cattle	Northeastern	0	0	0	0	0	0
5128	Cattle	Northeastern	0	0	0	0	0	0
5129	Cattle	Northeastern	0	0	0	0	0	0
5130	Cattle	Northeastern	0	0	0	0	0	0
5131	Cattle	Northeastern	0	0	0	0	0	0
5132	Cattle	Northeastern	0	0	1	0	0	1
5133	Cattle	Northeastern	0	0	0	0	0	0
5134	Cattle	Northeastern	0	0	0	0	0	0
5135	Cattle	Northeastern	0	0	0	0	0	0

5136	Cattle	Northeastern	0	0	0	0	0	0
5137	Cattle	Northeastern	0	0	0	0	1	1
5138	Cattle	Northeastern	0	0	0	0	0	0
5139	Cattle	Northeastern	0	0	0	0	0	0
5140	Cattle	Northeastern	0	0	0	0	0	0
5141	Cattle	Northeastern	0	0	0	0	0	0
5142	Cattle	Northeastern	0	0	0	0	0	0
5143	Cattle	Northeastern	0	0	0	0	0	0
5144	Cattle	Northeastern	0	0	0	0	0	0
5145	Cattle	Northeastern	0	0	0	0	0	0
5146	Cattle	Northeastern	0	0	1	0	0	1
5147	Cattle	Northeastern	0	0	0	0	0	0
5148	Cattle	Northeastern	0	0	0	0	0	0
5149	Cattle	Northeastern	0	0	0	0	0	0
5150	Cattle	Northeastern	0	0	0	0	0	0
5151	Cattle	Northeastern	0	0	0	0	0	0
5152	Cattle	Northeastern	0	0	1	0	0	1
5153	Cattle	Northeastern	0	0	0	0	0	0
5154	Cattle	Central	0	0	0	0	0	0
5155	Cattle	Central	0	0	1	0	0	1
5156	Cattle	Central	0	0	1	0	0	1
5157	Cattle	Central	0	0	1	0	0	1
5158	Cattle	Central	0	0	0	0	0	0
5159	Cattle	Central	0	0	1	0	0	1
5160	Cattle	Central	0	0	0	0	0	0
5161	Cattle	Central	0	0	0	0	0	0
5162	Cattle	Central	0	0	0	0	0	0
5163	Cattle	Central	0	0	0	0	0	0
5164	Cattle	Central	0	0	0	0	0	0
5165	Cattle	Central	0	0	1	0	0	1
5166	Cattle	Central	0	0	0	0	0	0
5167	Cattle	Central	0	0	1	0	0	1
5168	Cattle	Central	0	0	0	0	0	0
5169	Cattle	Central	0	0	1	1	0	1
5170	Cattle	Central	0	0	1	0	0	1
5171	Cattle	Central	0	0	0	0	0	0
5172	Cattle	Central	0	0	0	0	0	0
5173	Cattle	Central	0	0	1	0	0	1
5174	Cattle	Southern	0	0	1	0	0	1
5175	Cattle	Southern	0	0	1	0	0	1
5176	Cattle	Southern	0	0	0	0	0	0
5177	Cattle	Central	0	0	0	0	0	0
5178	Cattle	Central	0	1	1	0	0	1
5179	Cattle	Central	0	0	0	0	0	0
5180	Cattle	Central	0	0	0	0	0	0
5181	Cattle	Central	0	0	0	0	0	0
5182	Cattle	Central	0	0	0	0	0	0
5183	Cattle	Eastern	0	0	0	0	0	0

5184	Cattle	Eastern	0	0	0	0	0	0
5185	Cattle	Eastern	0	0	1	0	0	1
5186	Cattle	Eastern	0	0	1	1	0	1
5187	Cattle	Eastern	0	0	1	1	0	1
5188	Cattle	Eastern	0	0	0	0	0	0
5189	Cattle	Eastern	0	0	0	0	0	0
5190	Cattle	Eastern	0	0	1	0	0	1
5191	Cattle	Eastern	0	0	0	1	0	1
5192	Cattle	Eastern	0	0	1	0	0	1
5193	Cattle	Eastern	0	0	1	1	0	1
5194	Cattle	Eastern	0	0	1	0	0	1
5195	Cattle	Eastern	0	0	0	0	0	0
5196	Cattle	Eastern	0	0	1	0	0	1
5197	Cattle	Eastern	0	0	1	0	0	1
5198	Cattle	Eastern	0	0	1	0	0	1
5199	Cattle	Eastern	0	0	1	0	0	1
5200	Cattle	Eastern	0	0	1	0	0	1
5201	Cattle	Eastern	0	0	0	0	0	0
5202	Cattle	Eastern	0	0	0	0	0	0
5203	Cattle	Eastern	0	0	1	0	0	1
5204	Cattle	Eastern	0	0	0	0	0	0
5205	Cattle	Southern	0	0	1	1	0	1
5206	Cattle	Southern	0	0	1	0	0	1
5207	Cattle	Southern	0	0	0	0	0	0
5208	Cattle	Northeastern	0	0	0	0	0	1
5209	Cattle	Northeastern	0	0	1	1	0	1
5210	Cattle	Northeastern	0	0	1	1	0	1
5211	Cattle	Northeastern	0	0	1	1	0	1
5212	Cattle	Northeastern	0	0	0	0	0	0
5213	Cattle	Northeastern	0	0	1	1	0	1
5214	Cattle	Northeastern	0	0	1	1	0	1
5215	Cattle	Northeastern	0	0	0	1	0	1
5216	Cattle	Northeastern	0	0	0	1	0	1
5217	Cattle	Northeastern	0	0	1	1	0	1
5218	Cattle	Northeastern	0	0	1	1	0	1
5219	Cattle	Northeastern	0	0	1	1	0	1
5220	Cattle	Northeastern	0	0	1	1	0	1
5221	Cattle	Central	0	0	0	0	0	0
5222	Cattle	Central	0	0	0	0	0	0
5223	Cattle	Central	0	0	1	0	0	1
5224	Cattle	Central	0	0	1	0	0	1
5225	Cattle	Central	0	1	1	0	0	1
5226	Cattle	Central	0	0	0	0	0	0
5227	Cattle	Central	0	0	1	1	0	1
5228	Cattle	Central	0	0	1	0	0	1
5229	Cattle	Central	0	0	1	1	0	1
5230	Cattle	Central	0	0	0	0	0	0
5231	Cattle	Central	0	0	0	0	0	0

5232	Cattle	Central	0	0	1	1	0	1
5233	Cattle	Central	0	0	0	1	0	1
5234	Cattle	Central	0	0	0	0	0	0
5235	Cattle	Central	0	0	0	0	0	0
5236	Cattle	Central	0	0	1	1	0	1
5237	Cattle	Central	0	0	0	0	0	0
5238	Cattle	Central	0	0	1	0	0	1
5239	Cattle	Central	0	0	0	0	0	0
5240	Cattle	Central	0	0	0	0	0	0
5241	Cattle	Central	0	0	0	0	0	0
5242	Cattle	Central	0	0	1	1	0	1
5243	Cattle	Central	0	0	1	1	0	1
5244	Cattle	Central	0	0	0	1	0	1
5245	Cattle	Central	0	0	1	0	0	1
5246	Cattle	Central	0	0	0	1	0	1
5247	Cattle	Central	0	0	0	0	0	0
5248	Cattle	Central	0	0	0	0	0	0
5249	Cattle	Central	0	0	0	0	0	0
5250	Cattle	Central	0	0	0	1	0	1
5251	Cattle	Central	0	0	1	0	0	1
5252	Cattle	Central	0	0	0	0	0	0
5253	Cattle	Central	0	0	0	0	0	0
5254	Cattle	Central	0	0	0	0	0	0
5255	Cattle	Central	0	0	1	1	0	1
5256	Cattle	Central	0	1	1	0	0	1
5257	Cattle	Central	0	0	0	0	0	0
5258	Cattle	Northeastern	0	0	0	0	0	0
5259	Cattle	Northeastern	0	0	0	0	0	0
5260	Cattle	Northeastern	0	0	1	0	0	1
5261	Cattle	Northeastern	0	0	0	0	0	0
5262	Cattle	Northeastern	0	0	1	0	0	1
5263	Cattle	Northeastern	0	0	0	0	0	0
5264	Cattle	Northeastern	0	0	1	0	0	1
5265	Cattle	Northeastern	0	0	1	0	0	1
5266	Cattle	Northeastern	0	0	0	0	0	0
5267	Cattle	Northeastern	0	0	1	0	0	1
5268	Cattle	Northeastern	0	0	0	0	0	0
5269	Cattle	Northeastern	0	0	0	1	0	1
5270	Cattle	Northeastern	0	0	1	1	0	1
5271	Cattle	Northeastern	0	0	0	0	0	0
5272	Cattle	Northeastern	0	0	0	0	0	0
5273	Cattle	Northeastern	0	0	0	0	0	0
5274	Cattle	Northeastern	0	0	0	0	0	0
5275	Cattle	Northeastern	0	0	0	0	0	0
5276	Cattle	Northeastern	0	0	1	0	0	1
5277	Cattle	Central	0	0	0	0	0	0
5278	Cattle	Central	0	0	0	0	0	0
5279	Cattle	Central	0	0	0	0	0	0

5280	Cattle	Central	0	0	1	0	0	1
5281	Cattle	Central	0	1	1	0	0	1
5282	Cattle	Central	0	0	0	0	0	0
5283	Cattle	Central	0	0	1	1	0	1
5284	Cattle	Central	0	0	1	0	0	1
5285	Cattle	Central	0	0	1	0	0	1
5286	Cattle	Central	0	0	0	0	0	0
5287	Cattle	Central	0	0	1	0	0	1
5288	Cattle	Central	0	0	1	1	0	1
5289	Cattle	Central	0	0	0	0	0	0
5290	Cattle	Central	0	0	0	0	0	0
5291	Cattle	Central	0	0	0	0	0	0
5292	Cattle	Central	0	0	1	1	0	1
5293	Cattle	Central	0	0	0	0	0	0
5294	Cattle	Central	0	0	1	0	0	1
5295	Cattle	Central	0	0	0	0	0	0
5296	Cattle	Central	0	0	0	0	0	0
5297	Cattle	Central	0	0	0	0	0	0
5298	Cattle	Central	0	0	1	1	0	1
5299	Cattle	Central	0	0	1	1	0	1
5300	Cattle	Central	0	0	0	0	0	0
5301	Cattle	Central	0	0	1	1	0	1
5302	Cattle	Central	0	0	0	0	0	0
5303	Cattle	Central	0	0	0	0	0	0
5304	Cattle	Central	0	0	0	0	0	0
5305	Cattle	Central	0	0	1	1	0	1
5306	Cattle	Central	0	0	1	0	0	1
5307	Cattle	Central	0	0	1	0	0	1
5308	Cattle	Northern	0	0	0	0	0	0
5309	Cattle	Northern	0	0	0	0	0	0
5310	Cattle	Northern	0	0	0	0	0	0
5311	Cattle	Northern	0	0	1	0	0	1
5312	Cattle	Northern	0	0	0	0	0	0
5313	Cattle	Northern	0	0	0	0	0	0
5314	Cattle	Northern	0	0	1	1	0	1
5315	Cattle	Northern	0	0	0	0	0	1
5316	Cattle	Northern	0	0	1	1	0	1
5317	Cattle	Northern	0	0	1	1	0	1
5318	Cattle	Northern	0	0	0	0	0	0
5319	Cattle	Northern	0	0	1	1	0	1
5320	Cattle	Northern	0	0	1	0	0	1
5321	Cattle	Northern	0	0	0	1	0	1
5322	Cattle	Northern	0	0	1	0	0	1
5323	Cattle	Northern	0	0	1	1	0	1
5324	Cattle	Central	0	0	0	0	0	0
5325	Cattle	Central	0	0	0	0	0	0
5326	Cattle	Central	0	0	0	0	0	0
5327	Cattle	Central	0	0	0	0	0	0

5328	Cattle	Central	0	1	1	0	0	1
5329	Cattle	Central	0	0	0	0	0	0
5330	Cattle	Eastern	0	0	1	1	0	1
5331	Cattle	Eastern	0	0	1	0	0	1
5332	Cattle	Eastern	0	0	0	0	0	0
5333	Cattle	Eastern	0	0	1	1	0	1
5334	Cattle	Eastern	0	0	0	0	0	0
5335	Cattle	Eastern	0	0	1	1	0	1
5336	Cattle	Eastern	0	0	0	1	0	1
5337	Cattle	Eastern	0	0	1	1	0	1
5338	Cattle	Eastern	0	0	1	1	0	1
5339	Cattle	Eastern	0	0	0	0	0	0
5340	Cattle	Eastern	0	0	1	1	0	1
5341	Cattle	Eastern	0	0	1	1	0	1
5342	Cattle	Eastern	0	0	0	0	0	0
5343	Cattle	Eastern	0	0	1	0	0	1
5344	Cattle	Eastern	0	0	1	1	0	1
5345	Cattle	Eastern	0	0	1	1	0	1
5346	Cattle	Eastern	0	0	1	0	0	1
5347	Cattle	Eastern	0	0	1	1	0	1
5348	Cattle	Eastern	0	0	1	1	0	1
5349	Cattle	Eastern	0	0	0	0	0	0
5350	Cattle	Eastern	0	0	0	0	0	0
5351	Cattle	Eastern	0	0	0	0	0	0
5352	Cattle	Eastern	0	0	1	1	0	1
5353	Cattle	Eastern	0	0	1	1	0	1
5354	Cattle	Eastern	0	0	0	0	0	0
5355	Cattle	Eastern	0	0	1	0	0	1
5356	Cattle	Southern	0	0	1	1	0	1
5357	Cattle	Southern	0	0	1	1	1	1
5358	Cattle	Southern	0	0	1	1	0	1
5359	Cattle	Southern	0	0	1	0	0	1
5360	Cattle	Southern	0	0	1	1	0	1
5361	Cattle	Southern	0	0	1	0	0	1
5362	Cattle	Southern	0	0	1	0	0	1
5363	Cattle	Northeastern	0	0	1	0	0	1
5364	Cattle	Northeastern	0	0	1	1	0	1
5365	Cattle	Northeastern	0	0	1	0	0	1
5366	Cattle	Northeastern	0	0	0	0	0	0
5367	Cattle	Northeastern	0	0	0	0	0	0
5368	Cattle	Northeastern	0	0	1	0	0	1
5369	Cattle	Northeastern	0	0	1	1	0	1
5370	Cattle	Northeastern	0	0	0	0	0	0
5371	Cattle	Northeastern	0	0	1	1	0	1
5372	Cattle	Northeastern	0	0	1	1	0	1
5373	Cattle	Northeastern	0	0	1	1	0	1
5374	Cattle	Northeastern	0	0	1	1	0	1
5375	Cattle	Central	0	0	1	1	0	1

5376	Cattle	Central	0	0	1	0	0	1
5377	Cattle	Central	0	0	1	0	0	1
5378	Cattle	Central	0	0	0	0	0	0
5379	Cattle	Central	0	0	0	0	0	0
5380	Cattle	Central	0	0	1	0	0	1
5381	Cattle	Central	0	0	0	0	0	0
5382	Cattle	Central	0	0	0	0	0	0
5383	Cattle	Central	0	0	0	0	0	0
5384	Cattle	Central	0	0	0	0	0	0
5385	Cattle	Central	0	0	0	0	0	0
5386	Cattle	Central	0	0	0	0	0	0
5387	Cattle	Central	0	0	0	0	0	0
5388	Cattle	Central	0	0	0	0	0	0
5389	Cattle	Central	0	0	0	0	0	0
5390	Cattle	Central	0	0	1	0	0	1
5391	Cattle	Central	0	0	0	0	0	0
5392	Cattle	Central	0	0	0	0	0	0
5393	Cattle	Central	0	0	0	0	0	0
5394	Cattle	Central	0	0	0	0	0	0
5395	Cattle	Central	0	0	0	0	0	0
5396	Cattle	Central	0	0	0	0	0	0
5397	Cattle	Central	0	0	0	0	0	0
5398	Cattle	Northeastern	0	0	0	0	0	0
5399	Cattle	Northeastern	0	0	1	0	0	1
5400	Cattle	Northeastern	0	0	0	0	0	0
5401	Cattle	Northeastern	0	0	0	0	0	0
5402	Cattle	Northeastern	0	0	0	0	0	0
5403	Cattle	Eastern	0	1	1	1	0	1
5404	Cattle	Eastern	0	1	1	1	0	1
5405	Cattle	Eastern	0	0	0	0	0	0
5406	Cattle	Eastern	0	0	1	1	0	1
5407	Cattle	Eastern	0	0	1	1	0	1
5408	Cattle	Eastern	0	0	0	0	0	0
5409	Cattle	Eastern	0	1	0	0	0	1
5410	Cattle	Eastern	0	0	0	0	0	0
5411	Cattle	Eastern	0	0	1	1	0	1
5412	Cattle	Eastern	0	1	1	1	0	1
5413	Cattle	Eastern	0	0	0	1	0	1
5414	Cattle	Eastern	0	1	1	1	0	1
5415	Cattle	Eastern	0	0	1	1	1	1
5416	Cattle	Eastern	0	0	0	1	0	1
5417	Cattle	Eastern	0	0	1	1	0	1
5418	Cattle	Eastern	0	0	1	1	0	1
5419	Cattle	Eastern	0	0	0	0	0	0
5420	Cattle	Eastern	0	0	1	1	0	1
5421	Cattle	Eastern	0	1	0	1	0	1
5422	Cattle	Eastern	0	0	0	1	0	1
5423	Cattle	Eastern	0	1	0	1	0	1

5424	Cattle	Eastern	0	0	1	1	0	1
5425	Cattle	Eastern	0	1	1	1	1	1
5426	Cattle	Southern	0	0	1	1	0	1
5427	Cattle	Southern	0	0	0	0	0	0
5428	Cattle	Southern	0	0	1	1	0	1
5429	Cattle	Southern	0	0	0	0	0	0
5430	Cattle	Southern	0	0	1	1	0	1
5431	Cattle	Southern	0	0	0	0	0	0
5432	Cattle	Southern	0	0	0	1	0	1
5433	Cattle	Southern	0	0	1	1	0	1
5434	Cattle	Southern	0	0	0	0	0	0
5435	Cattle	Southern	0	0	0	0	0	0
5436	Cattle	Southern	0	0	1	1	0	1
5437	Cattle	Southern	0	0	0	0	0	0
5438	Cattle	Southern	0	0	0	0	0	0
5439	Cattle	Southern	0	0	1	1	0	1
5440	Cattle	Southern	0	0	0	0	0	0
5441	Cattle	Southern	0	0	1	1	0	1
5442	Cattle	Southern	0	0	1	1	0	1
5443	Cattle	Southern	0	1	1	1	0	1
5444	Cattle	Southern	0	1	1	1	0	1
5445	Cattle	Southern	0	1	1	1	0	1
5446	Cattle	Southern	0	0	0	0	0	0
5447	Cattle	Southern	0	0	1	1	0	1
5448	Cattle	Southern	0	0	0	0	0	0
5449	Cattle	Southern	0	0	1	1	0	1
5450	Cattle	Southern	0	0	1	1	0	1
5451	Cattle	Southern	0	1	1	1	0	1
5452	Cattle	Southern	0	0	1	1	0	1
5453	Cattle	Southern	0	0	1	1	1	1
5454	Cattle	Southern	0	0	1	1	1	1
5455	Cattle	Southern	0	0	1	1	0	1
5456	Cattle	Southern	0	0	1	1	1	1
5457	Cattle	Southern	0	0	0	1	0	1
5458	Cattle	Southern	0	0	0	1	0	1
5459	Cattle	Southern	0	0	1	1	0	1
5460	Cattle	Southern	0	0	0	1	0	1
5461	Cattle	Southern	0	0	1	1	0	1
5462	Cattle	Southern	0	0	0	1	0	1
5463	Cattle	Southern	0	0	0	1	0	1
5464	Cattle	Southern	0	0	1	1	0	1
5465	Cattle	Southern	0	0	0	0	0	0
5466	Cattle	Southern	0	0	1	1	0	1
5467	Cattle	Southern	0	0	0	1	0	1
5468	Cattle	Southern	0	0	0	0	0	0
5469	Cattle	Southern	0	0	0	1	0	1
5470	Cattle	Southern	0	0	0	1	0	1
5471	Cattle	Southern	0	0	1	1	0	1

5472	Cattle	Southern	0	0	0	0	0	0
5473	Cattle	Northeastern	0	0	0	0	0	0
5474	Cattle	Northeastern	0	0	0	0	0	0
5475	Cattle	Northeastern	0	0	0	0	0	0
5476	Cattle	Northeastern	0	0	0	0	0	0
5477	Cattle	Northeastern	0	0	0	0	0	0
5478	Cattle	Northeastern	0	0	0	0	0	0
5479	Cattle	Northeastern	0	0	0	0	0	0
5480	Cattle	Northeastern	0	0	0	0	0	0
5481	Cattle	Northeastern	0	0	0	0	0	0
5482	Cattle	Northeastern	0	0	1	1	0	1
5483	Cattle	Northeastern	0	0	1	1	0	1
5484	Cattle	Northeastern	0	0	0	0	0	0
5485	Cattle	Northeastern	0	0	0	0	0	0
5486	Cattle	Northeastern	0	0	0	1	0	1
5487	Cattle	Northeastern	0	0	0	1	0	1
5488	Cattle	Northeastern	0	0	1	1	0	1
5489	Cattle	Northeastern	0	0	1	1	0	1
5490	Cattle	Northeastern	0	0	1	1	0	1
5491	Cattle	Northeastern	0	0	0	0	0	0
5492	Cattle	Northeastern	0	0	0	0	0	0
5493	Cattle	Northeastern	0	0	0	0	0	0
5494	Cattle	Northeastern	0	0	1	1	1	1
5495	Cattle	Northeastern	0	0	0	1	0	1
5496	Cattle	Northeastern	0	0	1	1	0	1
5497	Cattle	Northeastern	0	0	1	1	0	1
5498	Cattle	Northeastern	0	0	1	1	0	1
5499	Cattle	Northeastern	0	0	0	0	0	0
5500	Cattle	Northeastern	0	0	0	1	0	1
5501	Cattle	Northeastern	0	0	1	1	0	1
5502	Cattle	Northeastern	0	0	1	1	0	1
5503	Cattle	Northeastern	0	0	0	0	0	0
5504	Cattle	Northeastern	0	0	1	1	0	1
5505	Cattle	Northeastern	0	0	1	1	0	1
5506	Cattle	Northeastern	0	0	1	1	0	1
5507	Cattle	Northeastern	0	0	1	1	0	1
5508	Cattle	Northeastern	0	0	1	1	0	1
5509	Cattle	Northeastern	0	0	1	1	0	1
5510	Cattle	Northeastern	0	0	0	1	0	1
5511	Cattle	Northeastern	0	0	0	0	0	0
5512	Cattle	Northeastern	0	0	1	1	0	1
5513	Cattle	Northeastern	0	0	1	1	0	1
5514	Cattle	Northeastern	0	0	1	1	0	1
5515	Cattle	Northeastern	0	0	1	1	0	1
5516	Cattle	Northeastern	0	0	0	0	0	0
5517	Cattle	Northeastern	0	0	0	0	0	0
5518	Cattle	Northeastern	0	0	1	1	0	1
5519	Cattle	Northeastern	0	0	1	1	0	1

5520	Cattle	Northeastern	0	0	0	0	0	0
5521	Cattle	Northeastern	0	0	0	1	0	1
5522	Cattle	Northeastern	0	0	0	1	0	1
5523	Cattle	Northeastern	0	0	0	0	0	0
5524	Cattle	Northeastern	0	0	1	1	0	1
5525	Cattle	Northeastern	0	0	1	1	1	1
5526	Cattle	Northeastern	0	0	1	1	0	1
5527	Cattle	Northeastern	0	0	1	1	0	1
5528	Cattle	Northeastern	0	0	1	1	0	1
5529	Cattle	Northeastern	0	0	1	1	0	1
5530	Cattle	Northeastern	0	0	1	1	1	1
5531	Cattle	Northeastern	0	0	0	0	0	0
5532	Cattle	Northeastern	0	0	0	0	0	0
5533	Cattle	Northeastern	0	0	1	1	0	1
5534	Cattle	Northeastern	0	0	1	1	0	1
5535	Cattle	Northeastern	0	0	0	0	0	0
5536	Cattle	Northeastern	0	0	0	1	0	1
5537	Cattle	Northeastern	0	0	0	0	0	0
5538	Cattle	Northeastern	0	0	1	1	0	1
5539	Cattle	Northeastern	0	0	1	1	0	1
5540	Cattle	Northeastern	0	0	0	1	0	1
5541	Cattle	Northeastern	0	0	0	0	0	0
5542	Cattle	Northeastern	0	0	0	0	0	0
5543	Cattle	Northeastern	0	0	1	1	0	1
5544	Cattle	Northeastern	0	1	1	1	0	1
5545	Cattle	Northeastern	0	0	0	1	0	1
5546	Cattle	Northeastern	0	0	0	0	1	1
5547	Cattle	Northeastern	0	0	0	0	0	0
5548	Cattle	Northeastern	0	0	0	0	0	0
5549	Cattle	Northeastern	0	0	0	0	0	0
5550	Cattle	Northeastern	0	0	0	0	0	0
5551	Cattle	Northeastern	0	0	0	0	0	1
5552	Cattle	Northeastern	0	0	0	0	0	0
5553	Cattle	Northeastern	0	0	1	1	0	1
5554	Cattle	Northeastern	0	0	1	1	0	1
5555	Cattle	Northeastern	0	0	0	0	0	0
5556	Cattle	Northeastern	0	0	0	0	0	0
5557	Cattle	Northeastern	0	0	1	1	0	1
5558	Cattle	Northeastern	0	0	0	0	0	0
5559	Cattle	Northeastern	0	0	0	0	0	0
5560	Cattle	Northeastern	0	0	0	0	0	0
5561	Cattle	Northeastern	0	0	1	1	0	1
5562	Cattle	Northeastern	0	0	0	0	0	0
5563	Cattle	Northeastern	0	0	0	0	0	0
5564	Cattle	Northeastern	0	0	0	0	0	0
5565	Cattle	Northeastern	0	0	1	1	0	1
5566	Cattle	Northeastern	0	0	0	0	0	0
5567	Cattle	Northeastern	0	0	0	0	0	0

5568	Cattle	Northeastern	0	0	0	0	0	0
5569	Cattle	Northeastern	0	0	1	1	0	1
5570	Cattle	Northeastern	0	0	1	1	0	1
5571	Cattle	Northeastern	0	0	0	0	0	0
5572	Cattle	Northeastern	0	0	0	0	0	0
5573	Cattle	Northeastern	0	0	1	1	0	1
5574	Cattle	Northeastern	0	0	1	1	0	1
5575	Cattle	Northeastern	0	0	0	0	0	0
5576	Cattle	Northeastern	0	0	0	0	0	0
5577	Cattle	Northeastern	0	0	1	1	0	1
5578	Cattle	Northeastern	0	0	0	0	0	0
5579	Cattle	Northeastern	0	0	0	0	0	0
5580	Cattle	Northeastern	0	0	0	0	0	0
5581	Cattle	Northeastern	0	0	0	0	0	0
5582	Cattle	Northeastern	0	0	0	0	0	0
5583	Cattle	Northeastern	0	0	0	0	0	0
5584	Cattle	Northeastern	0	0	0	0	0	0
5585	Cattle	Northeastern	0	0	0	0	0	0
5586	Cattle	Northeastern	0	0	0	0	0	0
5587	Cattle	Northeastern	0	0	1	1	0	1
5588	Cattle	Northeastern	0	0	0	0	0	0
5589	Cattle	Northeastern	0	0	1	1	0	1
5590	Cattle	Northeastern	0	0	1	1	0	1
5591	Cattle	Central	0	0	0	0	0	0
5592	Cattle	Central	0	0	0	0	0	0
5593	Cattle	Central	0	0	0	0	0	0
5594	Cattle	Central	0	1	1	0	0	1
5595	Cattle	Central	0	0	0	0	0	0
5596	Cattle	Eastern	0	0	0	0	0	0
5597	Cattle	Eastern	0	0	0	0	0	0
5598	Cattle	Eastern	0	0	0	0	0	0
5599	Cattle	Eastern	0	0	1	1	1	1
5600	Cattle	Eastern	0	0	1	1	0	1
5601	Cattle	Eastern	0	1	1	1	0	1
5602	Cattle	Eastern	0	0	1	1	1	1
5603	Cattle	Eastern	0	0	0	0	0	0
5604	Cattle	Eastern	0	0	1	1	0	1
5605	Cattle	Eastern	0	0	0	0	0	0
5606	Cattle	Eastern	0	0	1	1	0	1
5607	Cattle	Eastern	0	1	1	1	0	1
5608	Cattle	Eastern	0	0	1	1	0	1
5609	Cattle	Eastern	0	0	1	1	0	1
5610	Cattle	Eastern	0	0	1	1	1	1
5611	Cattle	Central	0	0	1	1	0	1
5612	Cattle	Central	0	0	1	1	0	1
5613	Cattle	Central	0	0	1	1	0	1
5614	Cattle	Central	0	0	1	1	0	1
5615	Cattle	Central	0	0	1	1	0	1

5616	Cattle	Eastern	0	0	1	1	0	1
5617	Cattle	Eastern	0	0	0	0	0	0
5618	Cattle	Central	0	0	1	1	0	1
5619	Cattle	Central	0	0	0	0	0	0
5620	Cattle	Central	0	0	1	1	0	1
5621	Cattle	Central	0	0	1	1	0	1
5622	Cattle	Southern	0	0	1	1	0	1
5623	Cattle	Southern	0	0	0	0	0	0
5624	Cattle	Southern	0	0	1	1	0	1
5625	Cattle	Southern	0	0	1	1	0	1
5626	Cattle	Southern	0	0	1	1	0	1
5627	Cattle	Southern	0	0	1	1	0	1
5628	Cattle	Southern	0	0	1	1	0	1
5629	Cattle	Southern	0	0	1	1	0	1
5630	Cattle	Central	0	0	1	1	0	1
5631	Cattle	Central	0	0	0	0	0	0
5632	Cattle	Central	0	0	1	0	0	1
5633	Cattle	Central	0	0	1	0	0	1
5634	Cattle	Southern	0	0	1	1	0	1
5635	Cattle	Southern	0	0	1	1	0	1
5636	Cattle	Southern	0	0	0	0	0	0
5637	Cattle	Southern	0	0	0	0	0	0
5638	Cattle	Southern	0	0	0	1	0	1
5639	Pig	Northern	0	0	0	0	0	0
5640	Pig	Northern	0	0	0	0	0	0
5641	Pig	Northern	0	0	0	0	0	0
5642	Pig	Northern	0	0	0	0	0	0
5643	Pig	Central	0	0	0	0	0	0
5644	Pig	Central	0	0	0	0	0	0
5645	Pig	Central	0	0	0	0	0	0
5646	Pig	Central	0	0	0	0	0	0
5647	Pig	Central	0	0	0	0	0	0
5648	Pig	Central	0	0	0	0	0	0
5649	Pig	Central	0	0	0	0	0	0
5650	Pig	Central	0	0	0	0	0	0
5651	Pig	Central	0	0	0	0	0	0
5652	Pig	Central	0	0	0	0	0	0
5653	Pig	Central	0	0	0	0	0	0
5654	Pig	Central	0	0	0	0	0	0
5655	Pig	Central	0	0	0	0	0	0
5656	Pig	Central	0	0	0	0	0	0
5657	Pig	Central	0	0	0	0	0	0
5658	Pig	Central	0	0	0	0	0	0
5659	Pig	Central	0	0	0	0	0	0
5660	Pig	Central	0	0	0	0	0	0
5661	Pig	Central	0	0	0	0	0	0
5662	Pig	Central	0	0	0	0	0	0
5663	Pig	Central	0	0	0	0	0	0

5664	Pig	Central	0	0	0	0	0	0
5665	Pig	Central	0	0	0	0	0	0
5666	Pig	Central	0	0	0	0	0	0
5667	Pig	Central	0	0	0	0	0	0
5668	Pig	Central	0	0	0	0	0	0
5669	Pig	Central	0	0	0	0	0	0
5670	Pig	Central	0	0	0	0	0	0
5671	Pig	Central	0	0	0	0	0	0
5672	Pig	Central	0	0	0	0	0	0
5673	Pig	Northern	0	0	0	0	0	0
5674	Pig	Northern	0	0	0	0	0	0
5675	Pig	Northern	0	0	0	0	0	0
5676	Pig	Northern	0	0	0	0	0	0
5677	Pig	Southern	0	0	0	0	0	0
5678	Pig	Southern	0	0	0	0	0	0
5679	Pig	Southern	0	0	0	0	0	0
5680	Pig	Southern	0	0	1	0	0	1
5681	Pig	Central	0	0	0	0	0	0
5682	Pig	Central	0	0	0	0	0	0
5683	Pig	Central	0	0	0	0	0	0
5684	Pig	Central	0	0	0	0	0	0
5685	Pig	Central	0	0	0	0	0	0
5686	Pig	Central	0	0	0	0	0	0
5687	Pig	Central	0	0	0	0	0	0
5688	Pig	Central	0	0	0	0	0	0
5689	Pig	Central	0	0	0	0	0	0
5690	Pig	Central	0	0	0	0	0	0
5691	Pig	Central	0	0	0	0	0	0
5692	Pig	Central	0	0	0	0	0	0
5693	Pig	Central	0	0	0	0	0	0
5694	Pig	Central	0	0	0	0	0	0
5695	Pig	Central	0	0	0	0	0	0
5696	Pig	Central	0	0	0	0	0	0
5697	Pig	Southern	0	0	0	0	0	0
5698	Pig	Southern	0	0	0	0	0	0
5699	Pig	Southern	0	0	0	1	0	1
5700	Pig	Southern	0	0	0	0	0	0
5701	Pig	Central	0	0	0	0	0	0
5702	Pig	Central	0	0	0	0	0	0
5703	Pig	Central	0	0	0	0	0	0
5704	Pig	Central	0	0	0	0	0	0
5705	Pig	Central	0	0	0	0	0	0
5706	Pig	Central	0	0	0	0	0	0
5707	Pig	Central	0	0	0	0	0	0
5708	Pig	Central	0	0	0	0	0	0
5709	Pig	Central	0	0	0	0	0	0
5710	Pig	Central	0	0	0	0	0	0
5711	Pig	Central	0	0	0	0	0	0

5712	Pig	Central	0	0	0	0	0	0
5713	Pig	Central	0	0	0	0	0	0
5714	Pig	Central	0	0	0	0	0	0
5715	Pig	Central	0	0	0	0	0	0
5716	Pig	Central	0	0	0	0	0	0
5717	Pig	Central	0	0	0	0	0	0
5718	Pig	Central	0	0	0	0	0	0
5719	Pig	Central	0	0	0	0	0	0
5720	Pig	Central	0	0	0	0	0	0
5721	Pig	Central	0	0	0	0	0	0
5722	Pig	Central	0	0	0	0	0	0
5723	Pig	Central	0	0	0	0	0	0
5724	Pig	Central	0	0	0	0	0	0
5725	Pig	Central	0	0	0	0	0	0
5726	Pig	Central	0	0	0	0	0	0
5727	Pig	Central	0	0	0	0	0	0
5728	Pig	Central	0	0	0	0	0	0
5729	Pig	Central	0	0	0	0	0	0
5730	Pig	Central	0	0	0	0	0	0
5731	Pig	Central	0	0	0	0	0	0
5732	Pig	Central	0	0	0	0	0	0
5733	Pig	Central	0	0	0	0	0	0
5734	Pig	Central	0	0	0	0	0	0
5735	Pig	Central	0	0	0	0	0	0
5736	Pig	Central	0	0	0	0	0	0
5737	Pig	Central	0	0	0	0	0	0
5738	Pig	Central	0	0	0	0	0	0
5739	Pig	Central	0	0	0	0	0	0
5740	Pig	Central	0	0	0	0	0	0
5741	Pig	Central	0	0	0	0	0	0
5742	Pig	Central	0	0	0	0	0	0
5743	Pig	Central	0	0	0	0	0	0
5744	Pig	Central	0	0	0	0	0	0
5745	Pig	Central	0	0	0	0	0	0
5746	Pig	Central	0	0	0	0	0	0
5747	Pig	Central	0	0	0	0	0	0
5748	Pig	Central	0	0	0	0	0	0
5749	Pig	Central	0	0	0	0	0	0
5750	Pig	Central	0	0	0	0	0	0
5751	Pig	Central	0	0	0	0	0	0
5752	Pig	Central	0	0	0	0	0	0
5753	Pig	Central	0	0	0	1	0	1
5754	Pig	Central	0	0	0	0	0	0
5755	Pig	Central	0	0	0	0	0	0
5756	Pig	Central	0	0	0	0	0	0
5757	Pig	Central	0	0	0	0	0	0
5758	Pig	Central	0	0	0	0	0	0
5759	Pig	Central	0	0	0	0	0	0

5760	Pig	Central	0	0	0	0	0	0
5761	Pig	Central	0	0	0	0	0	0
5762	Pig	Central	0	0	0	0	0	0
5763	Pig	Central	0	0	1	0	0	1
5764	Pig	Central	0	0	0	0	0	0
5765	Pig	Central	0	0	0	0	0	0
5766	Pig	Central	0	0	0	0	0	0
5767	Pig	Central	0	0	0	0	0	0
5768	Pig	Central	0	0	0	0	0	0
5769	Pig	Central	0	0	0	0	0	0
5770	Pig	Central	0	0	1	0	0	1
5771	Pig	Central	0	0	0	0	0	0
5772	Pig	Central	0	0	1	0	0	1
5773	Pig	Central	0	0	0	0	0	0
5774	Pig	Central	0	0	0	0	0	0
5775	Pig	Central	0	0	0	0	0	0
5776	Pig	Central	0	0	0	0	0	0
5777	Pig	Central	0	0	0	0	0	0
5778	Pig	Central	0	0	0	0	0	0
5779	Pig	Central	0	0	0	0	0	0
5780	Pig	Central	0	0	0	0	0	0
5781	Pig	Central	0	0	0	0	0	0
5782	Pig	Central	0	0	0	0	0	0
5783	Pig	Central	0	0	0	0	0	0
5784	Pig	Central	0	0	0	0	0	0
5785	Pig	Central	0	0	0	0	0	0
5786	Pig	Central	0	0	0	0	0	0
5787	Pig	Central	0	0	0	0	0	0
5788	Pig	Central	0	0	0	0	0	0
5789	Pig	Central	0	0	0	0	0	0
5790	Pig	Central	0	0	0	0	0	0
5791	Pig	Central	0	0	0	0	0	0
5792	Pig	Central	0	0	0	0	0	0
5793	Pig	Central	0	0	0	0	0	0
5794	Pig	Central	0	0	0	0	0	0
5795	Pig	Central	0	0	0	0	0	0
5796	Pig	Central	0	0	0	0	0	0
5797	Pig	Central	0	0	0	0	0	0
5798	Pig	Central	0	0	0	0	0	0
5799	Pig	Central	0	0	0	0	0	0
5800	Pig	Central	0	0	0	0	0	0
5801	Pig	Central	0	0	0	0	0	0
5802	Pig	Central	0	0	0	0	0	0
5803	Pig	Central	0	0	0	0	0	0
5804	Pig	Central	0	0	0	0	0	0
5805	Pig	Central	0	0	0	0	0	0
5806	Pig	Central	0	0	0	0	0	0
5807	Pig	Central	0	0	0	0	0	0

5808	Pig	Central	0	0	0	0	0	0
5809	Pig	Central	0	0	0	0	0	0
5810	Pig	Central	0	0	0	0	0	0
5811	Pig	Central	0	0	0	0	0	0
5812	Pig	Central	0	0	0	0	0	0
5813	Pig	Central	0	0	0	0	0	0
5814	Pig	Central	0	0	0	0	0	0
5815	Pig	Central	0	0	0	0	0	0
5816	Pig	Central	0	0	0	0	0	0
5817	Pig	Central	0	0	0	0	0	0
5818	Pig	Central	0	0	0	0	0	0
5819	Pig	Central	0	0	0	0	0	0
5820	Pig	Central	0	0	0	0	0	0
5821	Pig	Central	0	0	0	0	0	0
5822	Pig	Central	0	0	0	0	0	0
5823	Pig	Central	0	0	0	0	0	0
5824	Pig	Central	0	0	0	0	0	0
5825	Pig	Central	0	0	0	0	0	0
5826	Pig	Central	0	0	0	0	0	0
5827	Pig	Central	0	0	0	0	0	0
5828	Pig	Central	0	0	0	0	0	0
5829	Pig	Central	0	0	0	0	0	0
5830	Pig	Central	0	0	0	0	0	0
5831	Pig	Central	0	0	0	0	0	0
5832	Pig	Central	0	0	1	0	0	1
5833	Pig	Central	0	0	0	0	0	0
5834	Pig	Central	0	0	1	0	0	1
5835	Pig	Central	0	0	0	0	0	0
5836	Pig	Central	0	0	0	0	0	0
5837	Pig	Central	0	0	1	0	0	1
5838	Pig	Central	0	0	0	0	0	0
5839	Pig	Central	0	0	1	0	0	1
5840	Pig	Central	0	0	0	0	0	0
5841	Pig	Central	0	0	0	0	0	0
5842	Pig	Central	0	0	0	0	0	0
5843	Pig	Central	0	0	0	0	0	0
5844	Pig	Central	0	0	0	0	0	0
5845	Pig	Central	0	0	0	0	0	0
5846	Pig	Central	0	0	0	0	0	0
5847	Pig	Central	0	0	0	0	0	0
5848	Pig	Central	0	0	0	0	0	0
5849	Pig	Central	0	0	0	0	0	0
5850	Pig	Central	0	0	0	0	0	0
5851	Pig	Central	0	0	0	0	0	0
5852	Pig	Central	0	0	0	0	0	0
5853	Pig	Central	0	0	0	0	0	0
5854	Pig	Central	0	0	0	0	0	0
5855	Pig	Central	0	0	0	0	0	0

5856	Pig	Central	0	0	0	0	0	0
5857	Pig	Central	0	0	0	0	0	0
5858	Pig	Central	0	0	0	0	0	0
5859	Pig	Central	0	0	0	0	0	0
5860	Pig	Central	0	0	1	0	0	1
5861	Pig	Central	0	0	0	0	0	0
5862	Pig	Central	0	0	0	0	0	0
5863	Pig	Central	0	0	0	0	0	0
5864	Pig	Central	0	0	1	0	0	1
5865	Pig	Central	0	0	0	0	0	0
5866	Pig	Central	0	0	0	0	0	0
5867	Pig	Central	0	0	1	0	0	1
5868	Pig	Central	0	0	0	0	0	0
5869	Pig	Central	0	0	0	0	0	0
5870	Pig	Central	0	0	0	0	0	0
5871	Pig	Central	0	0	0	0	0	0
5872	Pig	Central	0	0	0	0	0	0
5873	Pig	Central	0	0	0	0	0	0
5874	Pig	Central	0	0	1	0	0	1
5875	Pig	Central	0	0	0	0	0	0
5876	Pig	Central	0	0	1	0	0	1
5877	Pig	Central	0	0	1	0	0	1
5878	Pig	Central	0	0	0	0	0	0
5879	Pig	Central	0	0	0	0	0	0
5880	Pig	Central	0	0	0	0	0	0
5881	Pig	Central	0	0	1	0	0	1
5882	Pig	Central	0	0	0	0	0	0
5883	Pig	Central	0	0	0	0	0	0
5884	Pig	Central	0	0	0	0	0	0
5885	Pig	Central	0	0	0	0	0	0
5886	Pig	Central	0	0	0	0	0	0
5887	Pig	Central	0	0	0	0	0	0
5888	Pig	Central	0	0	0	0	0	0
5889	Pig	Central	0	0	0	0	0	0
5890	Pig	Central	0	0	0	0	0	0
5891	Pig	Central	0	0	0	0	0	0
5892	Pig	Central	0	0	0	0	0	0
5893	Pig	Central	0	0	0	0	0	0
5894	Pig	Central	0	0	0	0	0	0
5895	Pig	Central	0	0	0	0	0	0
5896	Pig	Central	0	0	0	0	0	0
5897	Pig	Central	0	0	0	0	0	0
5898	Pig	Central	0	0	0	0	0	0
5899	Pig	Central	0	0	0	0	0	0
5900	Pig	Central	0	0	0	0	0	0
5901	Pig	Central	0	0	0	0	0	0
5902	Pig	Central	0	0	0	0	0	0
5903	Pig	Central	0	0	0	0	0	0

5904	Pig	Central	0	0	0	0	0	0
5905	Pig	Central	0	0	0	0	0	0
5906	Pig	Central	0	0	0	0	0	0
5907	Pig	Central	0	0	0	0	0	0
5908	Pig	Central	0	0	0	0	0	0
5909	Pig	Central	0	0	0	0	0	0
5910	Pig	Central	0	0	0	0	0	0
5911	Pig	Central	0	0	0	0	0	0
5912	Pig	Central	0	0	0	0	0	0
5913	Pig	Central	0	0	0	0	0	0
5914	Pig	Central	0	0	0	0	0	0
5915	Pig	Central	0	0	1	0	0	1
5916	Pig	Central	0	0	0	0	0	0
5917	Pig	Central	0	0	0	0	0	0
5918	Pig	Central	0	0	0	0	0	0
5919	Pig	Central	0	0	0	0	0	0
5920	Pig	Central	0	0	0	0	0	0
5921	Pig	Central	0	0	0	0	0	0
5922	Pig	Central	0	0	0	0	0	0
5923	Pig	Central	0	0	0	0	0	0
5924	Pig	Central	0	0	0	0	0	0
5925	Pig	Central	0	0	0	0	0	0
5926	Pig	Central	0	0	0	0	0	0
5927	Pig	Central	0	0	0	0	0	0
5928	Pig	Central	0	0	0	0	0	0
5929	Pig	Central	0	0	0	0	0	0
5930	Pig	Central	0	0	0	0	0	0
5931	Pig	Central	0	0	0	0	0	0
5932	Pig	Central	0	0	0	0	0	0
5933	Pig	Central	0	0	0	0	0	0
5934	Pig	Central	0	0	0	0	0	0
5935	Pig	Central	0	0	0	0	0	0
5936	Pig	Central	0	0	0	0	0	0
5937	Pig	Central	0	0	0	0	0	0
5938	Pig	Central	0	0	0	0	0	0
5939	Pig	Central	0	0	0	0	0	0
5940	Pig	Central	0	0	0	0	0	0
5941	Pig	Central	0	0	0	0	0	0
5942	Pig	Central	0	0	0	0	0	0
5943	Pig	Central	0	0	0	0	0	0
5944	Pig	Central	0	0	0	0	0	0
5945	Pig	Central	0	0	0	0	0	0
5946	Pig	Central	0	0	0	0	0	0
5947	Pig	Central	0	0	0	0	0	0
5948	Pig	Central	0	0	0	0	0	0
5949	Pig	Central	1	0	0	1	0	1
5950	Pig	Central	0	0	0	0	0	0
5951	Pig	Central	0	0	0	0	0	0

5952	Pig	Central	0	0	0	0	0	0
5953	Pig	Central	0	0	0	0	0	0
5954	Pig	Central	0	0	0	0	0	0
5955	Pig	Central	0	0	0	0	0	0
5956	Pig	Central	0	0	0	0	0	0
5957	Pig	Central	0	0	0	0	0	0
5958	Pig	Central	0	0	0	0	0	0
5959	Pig	Central	0	0	0	0	0	0
5960	Pig	Central	0	0	0	0	0	0
5961	Pig	Central	0	0	0	1	0	1
5962	Pig	Central	0	0	0	0	0	0
5963	Pig	Central	0	0	0	0	0	0
5964	Pig	Central	0	0	0	0	0	0
5965	Pig	Central	0	0	0	0	0	0
5966	Pig	Central	0	0	0	0	0	0
5967	Pig	Central	0	0	0	1	0	1
5968	Pig	Central	0	0	0	0	0	0
5969	Pig	Central	0	0	0	0	0	0
5970	Pig	Central	0	0	0	0	0	0
5971	Pig	Central	0	0	0	0	0	0
5972	Pig	Central	0	0	0	0	0	0
5973	Pig	Central	0	0	0	0	0	0
5974	Pig	Central	0	0	0	0	0	0
5975	Pig	Central	0	0	0	0	0	0
5976	Pig	Central	0	0	0	1	0	1
5977	Pig	Central	0	0	0	1	0	1
5978	Pig	Central	0	0	0	0	0	0
5979	Pig	Central	0	0	0	0	0	0
5980	Pig	Central	0	0	0	0	0	0
5981	Pig	Central	0	0	0	0	0	0
5982	Pig	Central	0	0	0	0	0	0
5983	Pig	Central	0	0	0	0	0	0
5984	Pig	Central	0	0	0	0	0	0
5985	Pig	Central	0	0	0	0	0	0
5986	Pig	Central	0	0	0	0	0	0
5987	Pig	Central	0	0	0	0	0	0
5988	Pig	Central	0	0	0	0	0	0
5989	Pig	Central	0	0	0	0	0	0
5990	Pig	Central	0	0	0	0	0	0
5991	Pig	Central	0	0	0	0	0	0
5992	Pig	Central	0	0	0	0	0	0
5993	Pig	Central	0	0	0	0	0	0
5994	Pig	Central	0	0	0	0	0	0
5995	Pig	Central	0	0	0	0	0	0
5996	Pig	Central	0	0	0	0	0	0
5997	Pig	Central	0	0	0	0	0	0
5998	Pig	Central	0	0	0	0	0	0
5999	Pig	Central	0	0	0	0	0	0

6000	Pig	Central	0	0	0	0	0	0
6001	Pig	Central	0	0	0	0	0	0
6002	Pig	Central	0	0	0	0	0	0
6003	Pig	Central	0	0	0	0	0	0
6004	Pig	Central	0	0	0	0	0	0
6005	Pig	Central	0	0	0	1	0	1
6006	Pig	Central	0	0	0	0	0	0
6007	Pig	Central	0	0	0	0	0	0
6008	Pig	Central	0	0	0	0	0	0
6009	Pig	Central	0	0	0	0	0	0
6010	Pig	Central	0	0	0	0	0	0
6011	Pig	Central	0	0	0	0	0	0
6012	Pig	Central	0	0	0	0	0	0
6013	Pig	Central	0	0	0	0	0	0
6014	Pig	Central	0	0	0	0	0	0
6015	Pig	Central	0	0	0	0	0	0
6016	Pig	Central	0	0	0	0	0	0
6017	Pig	Central	0	0	0	0	0	0
6018	Pig	Central	0	0	0	0	0	0
6019	Pig	Central	0	0	0	0	0	0
6020	Pig	Central	0	0	0	0	0	0
6021	Pig	Central	0	0	0	0	0	0
6022	Pig	Central	0	0	0	0	0	0
6023	Pig	Central	0	0	0	0	0	0
6024	Pig	Central	0	0	0	0	0	0
6025	Pig	Central	0	0	0	0	0	0
6026	Pig	Central	0	0	0	0	0	0
6027	Pig	Central	0	0	0	0	0	0
6028	Pig	Central	0	0	0	0	0	0
6029	Pig	Central	0	0	0	0	0	0
6030	Pig	Central	0	0	0	0	0	0
6031	Pig	Central	0	0	0	0	0	0
6032	Pig	Central	0	0	0	0	0	0
6033	Pig	Central	0	0	0	0	0	0
6034	Pig	Central	0	0	0	0	0	0
6035	Pig	Central	0	0	0	0	0	0
6036	Pig	Central	0	0	0	0	0	0
6037	Pig	Central	0	0	0	0	0	0
6038	Pig	Central	0	0	0	0	0	0
6039	Pig	Central	0	0	0	0	0	0
6040	Pig	Central	0	0	0	0	0	0
6041	Pig	Central	0	0	0	0	0	0
6042	Pig	Central	0	0	0	0	0	0
6043	Pig	Central	0	0	0	0	0	0
6044	Pig	Central	0	0	0	0	0	0
6045	Pig	Central	0	0	0	0	0	0
6046	Pig	Central	0	0	0	0	0	0
6047	Pig	Central	0	0	0	0	0	0

6048	Pig	Central	0	0	0	0	0	0
6049	Pig	Central	0	0	0	0	0	0
6050	Pig	Central	0	0	0	0	0	0
6051	Pig	Central	0	0	0	0	0	0
6052	Pig	Central	0	0	0	0	0	0
6053	Pig	Central	0	0	0	0	0	0
6054	Pig	Central	0	0	0	0	0	0
6055	Pig	Central	0	0	0	0	0	0
6056	Pig	Central	0	0	0	0	0	0
6057	Pig	Central	0	0	0	0	0	0
6058	Pig	Central	0	0	0	0	0	0
6059	Pig	Central	0	0	0	0	0	0
6060	Pig	Central	0	0	0	0	0	0
6061	Pig	Central	0	0	0	0	0	0
6062	Pig	Central	0	0	0	0	0	0
6063	Pig	Central	0	0	0	0	0	0
6064	Pig	Central	0	0	0	0	0	0
6065	Pig	Central	0	0	0	0	0	0
6066	Pig	Central	0	0	0	0	0	0
6067	Pig	Central	0	0	0	0	0	0
6068	Pig	Central	0	0	0	0	0	0
6069	Pig	Central	0	0	0	0	0	0
6070	Pig	Central	0	0	0	0	0	0
6071	Pig	Central	0	0	0	0	0	0
6072	Pig	Central	0	0	0	0	0	0
6073	Pig	Central	0	0	0	0	0	0
6074	Pig	Central	0	0	0	0	0	0
6075	Pig	Central	0	0	0	0	0	0
6076	Pig	Central	0	0	0	0	0	0
6077	Pig	Central	0	0	0	0	0	0
6078	Pig	Central	0	0	0	0	0	0
6079	Pig	Central	0	0	0	0	0	0
6080	Pig	Central	0	0	0	0	0	0
6081	Pig	Central	0	0	0	0	0	0
6082	Pig	Central	0	0	0	0	0	0
6083	Pig	Central	0	0	0	0	0	0
6084	Pig	Central	0	0	0	0	0	0
6085	Pig	Central	0	0	0	0	0	0
6086	Pig	Central	0	0	0	0	0	0
6087	Pig	Central	0	0	0	0	0	0
6088	Pig	Central	0	0	0	0	0	0
6089	Pig	Central	0	0	0	0	0	0
6090	Pig	Central	0	0	0	0	0	0
6091	Pig	Central	0	0	0	0	0	0
6092	Pig	Central	0	0	0	0	0	0
6093	Pig	Central	0	0	0	1	0	1
6094	Pig	Central	0	0	0	0	0	0
6095	Pig	Central	0	0	0	0	0	0

6096	Pig	Central	0	0	0	1	0	1
6097	Pig	Central	0	0	0	0	0	0
6098	Pig	Central	0	0	0	0	0	0
6099	Pig	Central	0	0	1	1	0	1
6100	Pig	Central	0	0	0	0	0	0
6101	Pig	Central	0	0	0	0	0	0
6102	Pig	Central	0	0	0	0	0	0
6103	Pig	Central	0	0	0	0	0	0
6104	Pig	Central	0	0	0	0	0	0
6105	Pig	Central	0	0	0	0	0	0
6106	Pig	Central	0	0	0	0	0	0
6107	Pig	Central	0	0	0	0	0	0
6108	Pig	Central	0	0	0	0	0	0
6109	Pig	Central	0	0	0	0	0	0
6110	Pig	Central	0	0	0	0	0	0
6111	Pig	Central	0	0	0	0	0	0
6112	Pig	Central	0	0	0	0	0	0
6113	Pig	Central	0	0	0	0	0	0
6114	Pig	Central	0	0	0	0	0	0
6115	Pig	Central	0	0	0	0	0	0
6116	Pig	Central	0	0	0	0	0	0
6117	Pig	Central	0	0	0	0	0	0
6118	Pig	Central	0	0	0	0	0	0
6119	Pig	Central	0	0	0	0	0	0
6120	Pig	Central	0	0	0	0	0	0
6121	Pig	Central	0	0	0	0	0	0
6122	Pig	Central	0	0	0	0	0	0
6123	Pig	Central	0	0	0	0	0	0
6124	Pig	Central	0	0	0	0	0	0
6125	Pig	Central	0	0	0	0	0	0
6126	Pig	Central	0	0	0	0	0	0
6127	Pig	Central	0	0	0	0	0	0
6128	Pig	Central	0	0	0	0	0	0
6129	Pig	Central	0	0	0	1	0	1
6130	Pig	Central	0	0	0	0	0	0
6131	Pig	Central	0	0	0	0	0	0
6132	Pig	Central	0	0	0	0	0	0
6133	Pig	Central	0	0	0	0	0	0
6134	Pig	Central	0	0	0	0	0	0
6135	Pig	Central	0	0	0	0	0	0
6136	Pig	Central	0	0	0	0	0	0
6137	Pig	Central	0	0	0	0	0	0
6138	Pig	Central	0	0	0	0	0	0
6139	Pig	Central	0	0	0	0	0	0
6140	Pig	Central	0	0	0	0	0	0
6141	Pig	Central	1	0	0	0	0	1
6142	Pig	Central	0	0	0	0	0	0
6143	Pig	Central	0	0	0	0	0	0

6144	Pig	Central	0	0	0	0	0	0
6145	Pig	Central	0	0	0	0	0	0
6146	Pig	Central	0	0	0	0	0	0
6147	Pig	Central	0	0	0	0	0	0
6148	Pig	Central	0	0	0	0	0	0
6149	Pig	Central	0	0	0	0	0	0
6150	Pig	Central	0	0	0	0	0	0
6151	Pig	Central	0	0	0	0	0	0
6152	Pig	Central	0	0	0	1	0	1
6153	Pig	Central	0	0	0	0	0	0
6154	Pig	Central	0	0	0	0	0	0
6155	Pig	Central	0	0	0	0	0	0
6156	Pig	Central	0	0	0	0	0	0
6157	Pig	Central	0	0	0	0	0	0
6158	Pig	Central	0	0	0	0	0	0
6159	Pig	Central	0	0	0	0	0	0
6160	Pig	Central	0	0	0	0	0	0
6161	Pig	Central	0	0	0	0	0	0
6162	Pig	Central	0	0	0	0	0	0
6163	Pig	Central	0	0	0	0	0	0
6164	Pig	Central	0	0	0	0	0	0
6165	Pig	Central	0	0	0	0	0	0
6166	Pig	Central	0	0	0	0	0	0
6167	Pig	Central	0	0	0	0	0	0
6168	Pig	Central	0	0	0	0	0	0
6169	Pig	Central	0	0	0	0	0	0
6170	Pig	Central	0	0	0	0	0	0
6171	Pig	Central	0	0	0	0	0	0
6172	Pig	Central	0	0	0	0	0	0
6173	Pig	Central	0	0	0	0	0	0
6174	Pig	Central	0	0	0	0	0	0
6175	Pig	Central	0	0	0	0	0	0
6176	Pig	Central	0	0	0	0	0	0
6177	Pig	Central	0	0	0	0	0	0
6178	Pig	Central	0	0	0	0	0	0
6179	Pig	Central	0	0	0	0	0	0
6180	Pig	Central	0	0	0	0	0	0
6181	Pig	Central	0	0	0	0	0	0
6182	Pig	Central	0	0	0	0	0	0
6183	Pig	Central	0	0	0	0	0	0
6184	Pig	Central	0	0	0	0	0	0
6185	Pig	Central	0	0	0	0	0	0
6186	Pig	Central	0	0	0	0	0	0
6187	Pig	Central	0	0	0	0	0	0
6188	Pig	Central	0	0	0	0	0	0
6189	Pig	Central	0	0	0	0	0	0
6190	Pig	Central	0	0	0	0	0	0
6191	Pig	Central	0	0	0	0	0	0

6192	Pig	Central	0	0	0	0	0	0
6193	Pig	Central	0	0	0	0	0	0
6194	Pig	Central	0	0	0	0	0	0
6195	Pig	Central	0	0	0	0	0	0
6196	Pig	Central	0	0	0	0	0	0
6197	Pig	Central	0	0	0	0	0	0
6198	Pig	Central	0	0	0	0	0	0
6199	Pig	Central	0	0	0	0	0	0
6200	Pig	Central	0	0	0	0	0	0
6201	Pig	Central	0	0	0	0	0	0
6202	Pig	Central	0	0	0	0	0	0
6203	Pig	Central	0	0	0	0	0	0
6204	Pig	Central	0	0	0	0	0	0
6205	Pig	Central	0	0	0	0	0	0
6206	Pig	Central	0	0	0	0	0	0
6207	Pig	Central	0	0	0	0	0	0
6208	Pig	Central	0	0	0	1	0	1
6209	Pig	Central	0	0	0	0	0	0
6210	Pig	Central	0	0	0	1	0	1
6211	Pig	Central	0	0	0	1	0	1
6212	Pig	Central	0	0	0	0	0	0
6213	Pig	Central	0	0	0	0	0	0
6214	Pig	Central	0	0	0	1	0	1
6215	Pig	Central	0	0	0	1	0	1
6216	Pig	Central	0	0	0	0	0	0
6217	Pig	Central	0	0	0	0	0	0
6218	Pig	Central	0	0	0	0	0	0
6219	Pig	Central	0	0	0	0	0	0
6220	Pig	Central	0	0	0	0	0	0
6221	Pig	Central	0	0	0	0	0	0
6222	Pig	Central	0	0	0	0	0	0
6223	Pig	Central	0	0	0	0	0	0
6224	Pig	Central	0	0	0	1	0	1
6225	Pig	Central	0	0	0	0	0	0
6226	Pig	Central	0	0	0	0	0	0
6227	Pig	Central	0	0	0	1	0	1
6228	Pig	Central	0	0	0	0	0	0
6229	Pig	Central	0	0	0	0	0	0
6230	Pig	Central	0	0	0	0	0	0
6231	Pig	Central	0	0	0	0	0	0
6232	Pig	Central	0	0	0	0	0	0
6233	Pig	Central	0	0	0	0	0	0
6234	Pig	Central	0	0	0	0	0	0
6235	Pig	Central	0	0	0	0	0	0
6236	Pig	Central	0	0	0	1	0	1
6237	Pig	Central	0	0	0	1	0	1
6238	Pig	Central	0	0	0	0	0	0
6239	Pig	Central	0	0	0	0	0	0

6240	Pig	Central	0	0	0	0	0	0
6241	Pig	Central	0	0	0	0	0	0
6242	Pig	Central	0	0	0	0	0	0
6243	Pig	Central	0	0	0	0	0	0
6244	Pig	Central	0	0	0	0	0	0
6245	Pig	Central	0	0	0	1	0	1
6246	Pig	Central	0	0	0	0	0	0
6247	Pig	Central	0	0	0	1	0	1
6248	Pig	Central	0	0	0	0	0	0
6249	Pig	Central	0	0	0	1	0	1
6250	Pig	Central	0	0	0	0	0	0
6251	Pig	Central	0	0	0	0	0	0
6252	Pig	Central	0	0	0	0	0	0
6253	Pig	Central	0	0	0	1	0	1
6254	Pig	Central	0	0	0	0	0	0
6255	Pig	Central	0	0	0	0	0	0
6256	Pig	Central	0	0	0	1	0	1
6257	Pig	Central	0	0	0	1	0	1
6258	Pig	Central	0	0	0	1	0	1
6259	Pig	Central	0	0	0	0	0	0
6260	Pig	Central	0	0	0	1	0	1
6261	Pig	Central	0	0	0	1	0	1
6262	Pig	Central	0	0	0	0	0	0
6263	Pig	Central	0	0	0	0	0	0
6264	Pig	Central	0	0	1	1	0	1
6265	Pig	Central	0	0	0	1	0	1
6266	Pig	Central	0	0	0	1	0	1
6267	Pig	Central	0	0	0	0	0	0
6268	Pig	Central	0	0	0	1	0	1
6269	Pig	Central	0	0	0	0	0	0
6270	Pig	Central	0	0	0	0	0	0
6271	Pig	Central	0	0	0	1	0	1
6272	Pig	Central	0	0	0	0	0	0
6273	Pig	Central	0	0	0	1	0	1
6274	Pig	Central	0	0	0	1	0	1
6275	Pig	Central	0	0	0	0	0	0
6276	Pig	Central	0	0	0	0	0	0
6277	Pig	Central	0	0	0	0	0	0
6278	Pig	Central	0	0	0	0	0	1
6279	Pig	Central	0	0	0	0	0	0
6280	Pig	Central	0	0	0	0	0	0
6281	Pig	Central	0	0	0	1	0	1
6282	Pig	Central	0	0	0	1	0	1
6283	Pig	Central	0	0	0	0	0	0
6284	Pig	Central	0	0	0	1	0	1
6285	Pig	Central	0	0	0	1	0	1
6286	Pig	Central	0	0	0	1	0	1
6287	Pig	Central	0	0	0	1	0	1

6288	Pig	Central	0	0	0	0	0	0
6289	Pig	Central	0	0	0	0	0	0
6290	Pig	Central	0	0	0	1	0	1
6291	Pig	Central	0	0	0	0	0	0
6292	Pig	Central	0	0	0	0	0	0
6293	Pig	Central	0	0	0	0	0	0
6294	Pig	Central	0	0	0	0	0	0
6295	Pig	Central	0	0	0	0	0	1
6296	Pig	Central	0	0	0	0	0	0
6297	Pig	Central	0	0	0	0	0	0
6298	Pig	Central	0	0	0	0	0	0
6299	Pig	Central	0	0	0	0	0	0
6300	Pig	Central	0	0	0	0	0	0
6301	Pig	Central	0	0	0	0	0	0
6302	Pig	Central	0	0	0	0	0	0
6303	Pig	Central	0	0	0	0	0	0
6304	Pig	Central	0	0	0	0	0	0
6305	Pig	Central	0	0	0	0	0	0
6306	Pig	Central	0	0	0	0	0	0
6307	Pig	Central	0	0	0	0	0	0
6308	Pig	Central	0	0	0	0	0	0
6309	Pig	Central	0	0	0	0	0	0
6310	Pig	Central	0	0	0	0	0	0
6311	Pig	Central	0	0	0	0	0	0
6312	Pig	Central	0	0	0	0	0	0
6313	Pig	Central	0	0	0	0	0	0
6314	Pig	Central	0	0	0	0	0	0
6315	Pig	Central	0	0	0	0	0	0
6316	Pig	Central	0	0	0	0	0	0
6317	Pig	Central	0	0	0	0	0	0
6318	Pig	Central	0	0	0	0	0	0
6319	Pig	Central	0	0	0	0	0	0
6320	Pig	Central	0	0	0	0	0	0
6321	Pig	Central	0	0	0	0	0	0
6322	Pig	Central	0	0	0	0	0	0
6323	Pig	Central	0	0	0	0	0	0
6324	Pig	Central	0	0	0	0	0	0
6325	Pig	Central	0	0	0	0	0	0
6326	Pig	Central	0	0	0	0	0	0
6327	Pig	Central	0	0	0	0	0	0
6328	Pig	Central	0	0	0	0	0	0
6329	Pig	Central	0	0	0	0	0	0
6330	Pig	Central	0	0	0	0	0	0
6331	Pig	Central	0	0	0	0	0	0
6332	Pig	Central	0	0	0	0	0	0
6333	Pig	Central	0	0	0	0	0	0
6334	Pig	Central	0	0	0	0	0	0
6335	Pig	Central	0	0	0	0	0	0

6336	Pig	Central	0	0	0	0	0	0
6337	Pig	Central	0	0	0	0	0	0
6338	Pig	Central	0	0	0	0	0	0
6339	Pig	Central	0	0	0	0	0	0
6340	Pig	Central	0	0	0	0	0	0
6341	Pig	Central	0	0	0	0	0	0
6342	Pig	Central	0	0	0	0	0	0
6343	Pig	Central	0	0	0	0	0	0
6344	Pig	Central	0	0	0	0	0	0
6345	Pig	Central	0	0	0	0	0	0
6346	Pig	Central	0	0	0	0	0	0
6347	Pig	Central	0	0	0	0	0	0
6348	Pig	Central	0	0	0	0	0	0
6349	Pig	Central	0	0	0	0	0	0
6350	Pig	Central	0	0	0	0	0	0
6351	Pig	Central	0	0	0	0	0	0
6352	Pig	Central	0	0	0	0	0	0
6353	Pig	Central	0	0	0	0	0	0
6354	Pig	Central	0	0	0	0	0	0
6355	Pig	Central	0	0	0	0	0	0
6356	Pig	Central	0	0	0	0	0	0
6357	Pig	Central	0	0	0	0	0	0
6358	Pig	Central	0	0	0	0	0	0
6359	Pig	Central	0	0	0	0	0	0
6360	Pig	Central	0	0	0	0	0	0
6361	Pig	Central	0	0	0	0	0	0
6362	Pig	Central	0	0	0	0	0	0
6363	Pig	Central	0	0	0	0	0	0
6364	Pig	Central	0	0	0	0	0	0
6365	Pig	Central	0	0	0	0	0	0
6366	Pig	Central	0	0	0	0	0	0
6367	Pig	Central	0	0	0	0	0	0
6368	Pig	Central	0	0	0	0	0	0
6369	Pig	Central	0	0	0	0	0	0
6370	Pig	Central	0	0	0	0	0	0
6371	Pig	Central	0	0	0	0	0	0
6372	Pig	Central	0	0	0	0	0	0
6373	Pig	Central	0	0	0	0	0	0
6374	Pig	Central	0	0	0	0	0	0
6375	Pig	Central	0	0	0	0	0	0
6376	Pig	Central	0	0	0	0	0	0
6377	Pig	Central	0	0	0	0	0	0
6378	Pig	Central	0	0	0	0	0	0
6379	Pig	Central	0	0	0	0	0	0
6380	Pig	Central	0	0	0	0	0	0
6381	Pig	Central	0	0	0	0	0	0
6382	Pig	Central	0	0	0	0	0	0
6383	Pig	Central	0	0	0	0	0	0

6384	Pig	Central	0	0	0	0	0	0
6385	Pig	Central	0	0	0	0	0	0
6386	Pig	Central	0	0	0	0	0	0
6387	Pig	Central	0	0	0	0	0	0
6388	Pig	Central	0	0	0	0	0	0
6389	Pig	Central	0	0	0	0	0	0
6390	Pig	Central	0	0	0	0	0	0
6391	Pig	Central	0	0	0	0	0	0
6392	Pig	Central	0	0	0	0	0	0
6393	Pig	Central	0	0	0	0	0	0
6394	Pig	Central	0	0	0	0	0	0
6395	Pig	Central	0	0	0	0	0	0
6396	Pig	Central	0	0	0	0	0	0
6397	Pig	Central	0	0	0	0	0	0
6398	Pig	Central	0	0	0	0	0	0
6399	Pig	Central	0	0	0	0	0	0
6400	Pig	Central	0	0	0	0	0	0
6401	Pig	Central	0	0	0	0	0	0
6402	Pig	Central	0	0	0	0	0	0
6403	Pig	Central	0	0	0	0	0	0
6404	Pig	Central	0	0	0	0	0	0
6405	Pig	Central	0	0	0	0	0	0
6406	Pig	Central	0	0	0	0	0	0
6407	Pig	Central	0	0	0	0	0	0
6408	Pig	Central	0	0	0	0	0	0
6409	Pig	Central	0	0	0	0	0	0
6410	Pig	Central	0	0	0	1	0	1
6411	Pig	Central	0	0	0	0	0	0
6412	Pig	Central	0	0	0	0	0	0
6413	Pig	Central	0	0	0	0	0	0
6414	Pig	Central	0	0	0	1	0	1
6415	Pig	Central	0	0	0	0	0	0
6416	Pig	Central	0	0	0	0	0	0
6417	Pig	Central	0	0	0	0	0	0
6418	Pig	Central	0	0	0	0	0	0
6419	Pig	Central	0	0	0	1	0	1
6420	Pig	Central	0	0	0	0	0	0
6421	Pig	Central	0	0	0	0	0	0
6422	Pig	Central	0	0	0	1	0	1
6423	Pig	Central	0	0	0	0	0	0
6424	Pig	Central	0	0	0	1	0	1
6425	Pig	Central	0	0	0	0	0	0
6426	Pig	Central	0	0	0	0	0	0
6427	Pig	Central	0	0	0	0	0	0
6428	Pig	Central	0	0	0	0	0	0
6429	Pig	Central	0	0	0	0	0	0
6430	Pig	Central	0	0	0	0	0	0
6431	Pig	Central	0	0	0	0	0	0

6432	Pig	Central	0	0	0	0	0	0
6433	Pig	Central	0	0	0	0	0	0
6434	Pig	Central	0	0	0	0	0	0
6435	Pig	Central	0	0	0	0	0	0
6436	Pig	Central	0	0	0	0	0	0
6437	Pig	Central	0	0	0	0	0	0
6438	Pig	Central	0	0	0	0	0	0
6439	Pig	Central	0	0	0	0	0	0
6440	Pig	Central	0	0	0	0	0	0
6441	Pig	Central	0	0	0	0	0	0
6442	Pig	Central	0	0	0	0	0	0
6443	Pig	Central	0	0	0	0	0	0
6444	Pig	Central	0	0	0	0	0	0
6445	Pig	Central	0	0	0	0	0	0
6446	Pig	Central	0	0	0	0	0	0
6447	Pig	Central	0	0	0	0	0	0
6448	Pig	Central	0	0	0	0	0	0
6449	Pig	Central	0	0	0	0	0	0
6450	Pig	Central	0	0	0	0	0	0
6451	Pig	Central	0	0	0	0	0	0
6452	Pig	Central	0	0	0	0	0	0
6453	Pig	Central	0	0	0	0	0	0
6454	Pig	Central	0	0	0	0	0	0
6455	Pig	Central	0	0	0	0	0	0
6456	Pig	Central	0	0	0	0	0	0
6457	Pig	Central	0	0	0	0	0	0
6458	Pig	Central	0	0	0	0	0	0
6459	Pig	Central	0	0	0	0	0	0
6460	Pig	Central	0	0	0	0	0	0
6461	Pig	Central	0	0	0	0	0	0
6462	Pig	Central	0	0	0	0	0	0
6463	Pig	Central	0	0	0	0	0	0
6464	Pig	Central	0	0	0	0	0	0
6465	Pig	Central	0	0	0	0	0	0
6466	Pig	Central	0	0	0	0	0	0
6467	Pig	Central	0	0	0	0	0	0
6468	Pig	Central	0	0	0	0	0	0
6469	Pig	Central	0	0	0	0	0	0
6470	Pig	Central	0	0	0	0	0	0
6471	Pig	Central	0	0	0	0	0	0
6472	Pig	Central	0	0	0	0	0	0
6473	Pig	Central	0	0	0	0	0	0
6474	Pig	Central	0	0	0	0	0	0
6475	Pig	Central	0	0	0	0	0	0
6476	Pig	Central	0	0	0	0	0	0
6477	Pig	Central	0	0	0	0	0	0
6478	Pig	Central	0	0	0	0	0	0
6479	Pig	Central	0	0	0	0	0	0

6480	Pig	Central	0	0	0	0	0	0
6481	Pig	Central	0	0	0	0	0	0
6482	Pig	Central	0	0	0	0	0	0
6483	Pig	Central	0	0	0	0	0	0
6484	Pig	Central	0	0	0	0	0	0
6485	Pig	Central	0	0	0	0	0	0
6486	Pig	Central	0	0	0	0	0	0
6487	Pig	Central	0	0	0	0	0	0
6488	Pig	Central	0	0	0	0	0	0
6489	Pig	Central	0	0	0	0	0	0
6490	Pig	Central	0	0	0	0	0	0
6491	Pig	Central	0	0	0	0	0	0
6492	Pig	Central	0	0	0	0	0	0
6493	Pig	Central	0	0	0	0	0	0
6494	Pig	Eastern	0	0	0	0	0	0
6495	Pig	Eastern	0	0	0	0	0	0
6496	Pig	Eastern	0	0	0	0	0	0
6497	Pig	Eastern	0	0	0	0	0	0
6498	Pig	Eastern	0	0	0	0	0	0
6499	Pig	Eastern	0	0	0	0	0	0
6500	Pig	Eastern	0	0	0	0	0	0
6501	Pig	Eastern	0	0	0	0	0	0
6502	Pig	Eastern	0	0	0	0	0	0
6503	Pig	Eastern	0	0	0	0	0	0
6504	Pig	Eastern	0	0	0	0	0	0
6505	Pig	Eastern	0	0	0	0	0	0
6506	Pig	Eastern	0	0	0	0	0	0
6507	Pig	Eastern	0	0	0	0	0	0
6508	Pig	Eastern	0	0	0	0	0	0
6509	Pig	Eastern	0	0	0	0	0	0
6510	Pig	Eastern	0	0	0	0	0	0
6511	Pig	Eastern	0	0	0	0	0	0
6512	Pig	Eastern	0	0	0	0	0	0
6513	Pig	Eastern	0	0	0	0	0	0
6514	Pig	Central	0	0	0	0	0	0
6515	Pig	Central	0	0	0	0	0	0
6516	Pig	Central	0	0	0	1	0	1
6517	Pig	Central	0	0	0	0	0	0
6518	Pig	Central	0	0	0	0	0	0
6519	Pig	Central	0	0	0	0	0	0
6520	Pig	Central	0	0	0	0	0	0
6521	Pig	Central	0	0	0	1	0	1
6522	Pig	Central	0	0	0	0	0	0
6523	Pig	Central	0	0	0	0	0	0
6524	Pig	Central	0	0	0	0	0	0
6525	Pig	Central	0	0	0	0	0	0
6526	Pig	Central	0	0	0	0	0	0
6527	Pig	Central	0	0	0	0	0	0

6528	Pig	Central	0	0	0	0	0	0
6529	Pig	Central	0	0	0	1	0	1
6530	Pig	Central	0	0	0	0	0	0
6531	Pig	Central	0	0	0	0	0	0
6532	Pig	Central	0	0	0	0	0	0
6533	Pig	Central	0	0	0	0	0	0
6534	Pig	Central	0	0	0	0	0	0
6535	Pig	Central	0	0	0	0	0	0
6536	Pig	Central	0	0	0	0	0	0
6537	Pig	Central	0	0	0	0	0	0
6538	Pig	Central	0	0	0	0	0	0
6539	Pig	Central	0	0	0	0	0	0
6540	Pig	Central	0	0	0	0	0	0
6541	Pig	Central	0	0	0	0	0	0
6542	Pig	Central	0	0	0	0	0	0
6543	Pig	Central	0	0	0	0	0	0
6544	Pig	Central	0	0	0	1	0	1
6545	Pig	Central	0	0	0	0	0	0
6546	Pig	Central	0	0	0	0	0	0
6547	Pig	Central	0	0	0	1	0	1
6548	Pig	Central	0	0	0	0	0	0
6549	Pig	Central	0	0	0	0	0	0
6550	Pig	Central	0	0	0	0	0	0
6551	Pig	Central	0	0	0	0	0	0
6552	Pig	Central	0	0	0	0	0	0
6553	Pig	Central	0	0	0	0	0	0
6554	Pig	Central	0	0	0	0	0	0
6555	Pig	Central	0	0	0	1	0	1
6556	Pig	Central	0	0	0	1	0	1
6557	Pig	Central	0	0	0	0	0	0
6558	Pig	Central	0	0	0	1	0	1
6559	Pig	Central	0	0	0	0	0	0
6560	Pig	Central	0	0	0	0	0	0
6561	Pig	Central	0	0	0	0	0	0
6562	Pig	Central	0	0	0	0	0	0
6563	Pig	Central	0	0	0	0	0	0
6564	Pig	Central	0	0	0	0	0	0
6565	Pig	Central	0	0	0	1	0	1
6566	Pig	Central	0	0	0	1	0	1
6567	Pig	Central	0	0	0	1	0	1
6568	Pig	Central	0	0	0	1	0	1
6569	Pig	Central	0	0	0	0	0	0
6570	Pig	Central	0	0	0	0	0	0
6571	Pig	Central	0	0	0	0	0	0
6572	Pig	Central	0	0	0	0	0	0
6573	Pig	Central	0	0	0	0	0	0
6574	Pig	Central	0	0	0	0	0	0
6575	Pig	Central	0	0	0	0	0	0

6576	Pig	Central	0	0	0	0	0	0
6577	Pig	Central	0	0	0	0	0	0
6578	Pig	Central	0	0	0	0	0	0
6579	Pig	Central	0	0	0	1	0	1
6580	Pig	Central	0	0	0	1	0	1
6581	Pig	Central	0	0	0	1	0	1
6582	Pig	Central	0	0	0	0	0	0
6583	Pig	Central	0	0	0	1	0	1
6584	Pig	Central	0	0	0	1	0	1
6585	Pig	Central	0	0	0	0	0	0
6586	Pig	Central	0	0	0	1	0	1
6587	Pig	Central	0	0	0	1	0	1
6588	Pig	Central	0	0	0	1	0	1
6589	Pig	Central	0	0	0	1	0	1
6590	Pig	Central	0	0	0	0	0	0
6591	Pig	Central	0	0	0	0	0	0
6592	Pig	Central	0	0	0	1	0	1
6593	Pig	Central	0	0	0	0	0	0
6594	Pig	Central	0	0	0	0	0	0
6595	Pig	Central	0	0	0	0	0	0
6596	Pig	Central	0	0	0	0	0	0
6597	Pig	Central	0	0	0	0	0	0
6598	Pig	Central	0	0	0	0	0	0
6599	Pig	Central	0	0	0	0	0	0
6600	Pig	Central	0	0	0	0	0	0
6601	Pig	Central	0	0	0	0	0	0
6602	Pig	Central	0	0	0	0	0	0
6603	Pig	Central	0	0	0	0	0	0
6604	Pig	Central	0	0	0	0	0	0
6605	Pig	Central	0	0	0	0	0	0
6606	Pig	Central	0	0	0	0	0	0
6607	Pig	Central	0	0	0	0	0	0
6608	Pig	Central	0	0	0	0	0	0
6609	Pig	Central	0	0	0	0	0	0
6610	Pig	Central	0	0	0	0	0	0
6611	Pig	Central	0	0	0	0	0	0
6612	Pig	Central	0	0	0	0	0	0
6613	Pig	Central	0	0	0	0	0	0
6614	Pig	Central	0	0	0	1	0	1
6615	Pig	Central	0	0	0	0	0	0
6616	Pig	Central	0	0	0	0	0	0
6617	Pig	Central	0	0	0	0	0	0
6618	Pig	Central	0	0	0	0	0	0
6619	Pig	Central	0	0	0	0	0	0
6620	Pig	Central	0	0	0	0	0	0
6621	Pig	Central	0	0	0	0	0	0
6622	Pig	Central	0	0	0	0	0	0
6623	Pig	Central	0	0	0	0	0	0

6624	Pig	Central	0	0	0	0	0	0
6625	Pig	Central	0	0	0	0	0	0
6626	Pig	Central	0	0	0	0	0	0
6627	Pig	Central	0	0	0	0	0	0
6628	Pig	Central	0	0	0	0	0	0
6629	Pig	Central	0	0	0	0	0	0
6630	Pig	Central	0	0	0	0	0	0
6631	Pig	Central	0	0	0	0	0	0
6632	Pig	Central	0	0	0	0	0	0
6633	Pig	Central	0	0	0	0	0	0
6634	Pig	Central	0	0	0	1	0	1
6635	Pig	Central	0	0	0	0	0	0
6636	Pig	Central	0	0	0	1	0	1
6637	Pig	Central	0	0	0	0	0	0
6638	Pig	Central	0	0	0	1	0	1
6639	Pig	Central	0	0	0	1	0	1
6640	Pig	Central	0	0	0	1	0	1
6641	Pig	Central	0	0	0	0	0	0
6642	Pig	Central	0	0	0	0	0	0
6643	Pig	Central	0	0	0	1	0	1
6644	Pig	Central	0	0	0	0	0	0
6645	Pig	Central	0	0	0	0	0	0
6646	Pig	Central	0	0	0	0	0	0
6647	Pig	Central	0	0	0	0	0	0
6648	Pig	Central	0	0	0	0	0	0
6649	Pig	Central	0	0	0	0	0	0
6650	Pig	Central	0	0	0	0	0	0
6651	Pig	Central	0	0	0	0	0	0
6652	Pig	Central	0	0	0	0	0	0
6653	Pig	Central	0	0	0	0	0	0
6654	Pig	Central	0	0	0	0	0	0
6655	Pig	Central	0	0	0	0	0	0
6656	Pig	Central	0	0	0	0	0	0
6657	Pig	Central	0	0	0	0	0	0
6658	Pig	Central	0	0	0	0	0	0
6659	Pig	Central	0	0	0	0	0	0
6660	Pig	Central	0	0	0	0	0	0
6661	Pig	Central	0	0	0	0	0	0
6662	Pig	Central	0	0	0	0	0	0
6663	Pig	Central	0	0	0	0	0	0
6664	Pig	Central	0	0	0	0	0	0
6665	Pig	Central	0	0	0	0	0	0
6666	Pig	Central	0	0	0	0	0	0
6667	Pig	Central	0	0	0	0	0	0
6668	Pig	Central	0	0	0	0	0	0
6669	Pig	Central	0	0	0	0	0	0
6670	Pig	Central	0	0	0	0	0	0
6671	Pig	Central	0	0	0	0	0	0

6672	Pig	Central	0	0	0	0	0	0
6673	Pig	Central	0	0	0	0	0	0
6674	Pig	Central	0	0	0	0	0	0
6675	Pig	Central	0	0	0	0	0	0
6676	Pig	Central	0	0	0	0	0	0
6677	Pig	Central	0	0	0	0	0	0
6678	Pig	Central	0	0	0	0	0	0
6679	Pig	Central	0	0	0	0	0	0
6680	Pig	Central	0	0	0	0	0	0
6681	Pig	Central	0	0	0	0	0	0
6682	Pig	Central	0	0	0	0	0	0
6683	Pig	Central	0	0	0	0	0	0
6684	Pig	Central	0	0	0	0	0	0
6685	Pig	Central	0	0	0	0	0	0
6686	Pig	Central	0	0	0	0	0	0
6687	Pig	Central	0	0	0	0	0	0
6688	Pig	Central	0	0	0	0	0	0
6689	Pig	Central	0	0	0	0	0	0
6690	Pig	Central	0	0	0	0	0	0
6691	Pig	Central	0	0	0	0	0	0
6692	Pig	Central	0	0	0	0	0	0
6693	Pig	Central	0	0	0	0	0	0
6694	Pig	Central	0	0	0	0	0	0
6695	Pig	Central	0	0	0	0	0	0
6696	Pig	Central	0	0	0	1	0	1
6697	Pig	Central	0	0	0	1	0	1
6698	Pig	Central	0	0	0	0	0	0
6699	Pig	Central	0	0	0	0	0	0
6700	Pig	Central	0	0	0	0	0	0
6701	Pig	Central	0	0	0	0	0	0
6702	Pig	Central	0	0	0	0	0	0
6703	Pig	Central	0	0	0	0	0	0
6704	Pig	Central	0	0	0	0	0	0
6705	Pig	Central	0	0	0	0	0	0
6706	Pig	Central	0	0	0	0	0	0
6707	Pig	Central	0	0	0	0	0	0
6708	Pig	Central	0	0	0	0	0	0
6709	Pig	Central	0	0	0	0	0	0
6710	Pig	Central	0	0	0	0	0	0
6711	Pig	Central	0	0	0	0	0	0
6712	Pig	Central	0	0	0	1	0	1
6713	Pig	Central	0	0	0	0	0	0
6714	Pig	Central	0	0	0	0	0	0
6715	Pig	Central	0	0	0	0	0	0
6716	Pig	Central	0	0	0	0	0	0
6717	Pig	Central	0	0	0	0	0	0
6718	Pig	Central	0	0	0	0	0	0
6719	Pig	Central	0	0	0	0	0	0

6720	Pig	Central	0	0	0	0	0	0
6721	Pig	Central	0	0	0	0	0	0
6722	Pig	Central	0	0	0	0	0	0
6723	Pig	Central	0	0	0	0	0	0
6724	Pig	Central	0	0	0	0	0	0
6725	Pig	Central	0	0	0	0	0	0
6726	Pig	Central	0	0	0	0	0	0
6727	Pig	Central	0	0	0	0	0	0
6728	Pig	Central	0	0	0	1	0	1
6729	Pig	Central	0	0	0	0	0	0
6730	Pig	Central	0	0	0	0	0	0
6731	Pig	Central	0	0	0	1	0	1
6732	Pig	Central	0	0	1	1	0	1
6733	Pig	Central	0	0	0	0	0	0
6734	Pig	Central	0	0	0	0	0	0
6735	Pig	Central	0	0	0	1	0	1
6736	Pig	Central	0	0	0	0	0	0
6737	Pig	Central	0	0	0	0	0	0
6738	Pig	Central	0	0	0	1	0	1
6739	Pig	Central	0	0	0	1	0	1
6740	Pig	Central	0	0	0	0	0	0
6741	Pig	Central	0	0	0	0	0	0
6742	Pig	Central	0	0	0	0	0	0
6743	Pig	Central	0	0	0	0	0	0
6744	Pig	Central	0	0	0	0	0	0
6745	Pig	Central	0	0	0	0	0	0
6746	Pig	Central	0	0	0	0	0	0
6747	Pig	Central	0	0	0	0	0	0
6748	Pig	Central	0	0	0	1	0	1
6749	Pig	Central	0	0	0	0	0	0
6750	Pig	Central	0	0	0	0	0	0
6751	Pig	Central	0	0	0	1	0	1
6752	Pig	Central	0	0	0	0	0	0
6753	Pig	Northeastern	0	0	0	0	0	0
6754	Pig	Northeastern	0	0	0	0	0	0
6755	Pig	Northeastern	0	0	0	0	0	0
6756	Pig	Northeastern	0	0	0	0	0	0
6757	Pig	Northeastern	0	0	0	0	0	0
6758	Pig	Northeastern	0	0	0	0	0	0
6759	Pig	Northeastern	0	0	0	0	0	0
6760	Pig	Northeastern	0	0	0	0	0	0
6761	Pig	Northeastern	0	0	0	0	0	0
6762	Pig	Northeastern	0	0	0	0	0	0
6763	Pig	Northeastern	0	0	0	0	0	0
6764	Pig	Northeastern	0	0	0	0	0	0
6765	Pig	Northeastern	0	0	0	0	0	0
6766	Pig	Northeastern	0	0	0	0	0	0
6767	Pig	Northeastern	0	0	0	0	0	0

6768	Pig	Northeastern	0	0	0	1	0	1
6769	Pig	Northeastern	0	0	0	0	0	0
6770	Pig	Northeastern	0	0	0	0	0	0
6771	Pig	Northeastern	0	0	0	0	0	0
6772	Pig	Northeastern	0	0	0	0	0	0
6773	Pig	Northeastern	0	0	0	0	0	0
6774	Pig	Northeastern	0	0	0	0	0	0
6775	Pig	Northeastern	0	0	0	0	0	0
6776	Pig	Northeastern	0	0	0	0	0	0
6777	Pig	Northeastern	0	0	0	0	0	0
6778	Pig	Northeastern	0	0	0	0	0	0
6779	Pig	Northeastern	0	0	0	0	0	0
6780	Pig	Northeastern	0	0	0	0	0	0
6781	Pig	Northeastern	0	0	0	0	0	0
6782	Pig	Northeastern	0	0	0	0	0	0
6783	Pig	Northeastern	0	0	0	0	0	0
6784	Pig	Northeastern	0	0	0	0	0	0
6785	Pig	Northeastern	0	0	0	0	0	0
6786	Pig	Northeastern	0	0	0	0	0	0
6787	Pig	Northeastern	0	0	0	0	0	0
6788	Pig	Northeastern	0	0	0	0	0	0
6789	Pig	Northeastern	0	0	0	0	0	0
6790	Pig	Northeastern	0	0	0	0	0	0
6791	Pig	Northeastern	0	0	0	0	0	0
6792	Pig	Northeastern	0	0	0	0	0	0
6793	Pig	Central	0	0	0	0	0	0
6794	Pig	Central	0	0	0	0	0	0
6795	Pig	Central	0	0	0	0	0	0
6796	Pig	Central	0	0	0	0	0	0
6797	Pig	Central	0	0	0	0	0	0
6798	Pig	Central	0	0	0	0	0	0
6799	Pig	Central	0	0	0	0	0	0
6800	Pig	Central	0	0	0	0	0	0
6801	Pig	Central	0	0	0	0	0	0
6802	Pig	Central	0	0	0	0	0	0
6803	Pig	Central	0	0	0	0	0	0
6804	Pig	Central	0	0	0	0	0	0
6805	Pig	Central	0	0	0	0	0	0
6806	Pig	Central	0	0	0	0	0	0
6807	Pig	Central	0	0	0	0	0	0
6808	Pig	Central	0	0	0	0	0	0
6809	Pig	Central	0	0	0	0	0	0
6810	Pig	Central	0	0	0	0	0	0
6811	Pig	Central	0	0	0	0	0	0
6812	Pig	Central	0	0	0	0	0	0
6813	Pig	Central	0	0	0	0	0	0
6814	Pig	Central	0	0	0	0	0	0
6815	Pig	Central	0	0	0	0	0	0

6816	Pig	Central	0	0	0	0	0	0
6817	Pig	Central	0	0	0	0	0	0
6818	Pig	Central	0	0	0	0	0	0
6819	Pig	Central	0	0	0	0	0	0
6820	Pig	Central	0	0	0	0	0	0
6821	Pig	Central	0	0	0	0	0	0
6822	Pig	Central	0	0	0	0	0	0
6823	Pig	Central	0	0	0	0	0	0
6824	Pig	Central	0	0	0	0	0	0
6825	Pig	Central	0	0	0	0	0	0
6826	Pig	Central	0	0	0	0	0	0
6827	Pig	Central	0	0	0	1	0	1
6828	Pig	Central	0	0	0	0	0	0
6829	Pig	Central	0	0	0	0	0	0
6830	Pig	Central	0	0	0	0	0	0
6831	Pig	Central	0	0	0	0	0	0
6832	Pig	Central	0	0	0	0	0	0
6833	Pig	Central	0	0	0	0	0	0
6834	Pig	Central	0	0	0	0	0	0
6835	Pig	Central	0	0	0	0	0	0
6836	Pig	Central	0	0	0	0	0	0
6837	Pig	Central	0	0	0	0	0	0
6838	Pig	Central	0	0	0	0	0	0
6839	Pig	Central	0	0	0	0	0	0
6840	Pig	Central	0	0	0	0	0	0
6841	Pig	Central	0	0	0	0	0	0
6842	Pig	Central	0	0	0	1	0	1
6843	Pig	Central	0	0	0	0	0	0
6844	Pig	Central	0	0	0	0	0	0
6845	Pig	Central	0	0	0	0	0	0
6846	Pig	Central	0	0	0	0	0	0
6847	Pig	Central	0	0	0	0	0	0
6848	Pig	Central	0	0	0	0	0	0
6849	Pig	Central	0	0	0	1	0	1
6850	Pig	Central	0	0	0	0	0	0
6851	Pig	Central	0	0	0	0	0	0
6852	Pig	Central	0	0	0	0	0	0
6853	Pig	Central	0	0	0	0	0	0
6854	Pig	Central	0	0	0	0	0	0
6855	Pig	Central	0	0	0	0	0	0
6856	Pig	Central	0	0	0	0	0	0
6857	Pig	Central	0	0	0	0	0	0
6858	Pig	Central	0	0	0	0	0	0
6859	Pig	Central	0	0	0	1	0	1
6860	Pig	Central	0	0	0	0	0	0
6861	Pig	Central	0	0	0	0	0	0
6862	Pig	Central	0	0	0	0	0	0
6863	Pig	Central	0	0	0	0	0	0

6864	Pig	Central	0	0	0	0	0	0
6865	Pig	Central	0	0	0	0	0	0
6866	Pig	Central	0	0	0	0	0	0
6867	Pig	Central	0	0	0	0	0	0
6868	Pig	Central	0	0	0	1	0	1
6869	Pig	Central	0	0	0	0	0	0
6870	Pig	Central	0	0	0	0	0	0
6871	Pig	Central	0	0	0	0	0	0
6872	Pig	Central	0	0	0	0	0	0
6873	Pig	Central	0	0	0	0	0	0
6874	Pig	Central	0	0	0	0	0	0
6875	Pig	Central	0	0	0	0	0	0
6876	Pig	Central	0	0	0	0	0	0
6877	Pig	Central	0	0	0	0	0	0
6878	Pig	Central	0	0	0	0	0	0
6879	Pig	Central	0	0	0	0	0	0
6880	Pig	Central	0	0	0	0	0	0
6881	Pig	Central	0	0	0	0	0	0
6882	Pig	Central	0	0	0	0	0	0
6883	Pig	Central	0	0	0	0	0	0
6884	Pig	Central	0	0	0	0	0	0
6885	Pig	Central	0	0	0	0	0	0
6886	Pig	Central	0	0	0	0	0	0
6887	Pig	Central	0	0	0	0	0	0
6888	Pig	Central	0	0	0	0	0	0
6889	Pig	Central	0	0	0	0	0	0
6890	Pig	Central	0	0	0	0	0	0
6891	Pig	Central	0	0	0	0	0	0
6892	Pig	Central	0	0	0	0	0	0
6893	Pig	Central	0	0	0	0	0	0
6894	Pig	Central	0	0	0	0	0	0
6895	Pig	Central	0	0	0	0	0	0
6896	Pig	Central	0	0	0	0	0	0
6897	Pig	Central	0	0	0	0	0	0
6898	Pig	Central	0	0	0	0	0	0
6899	Pig	Central	0	0	0	0	0	0
6900	Pig	Central	0	0	0	0	0	0
6901	Pig	Central	0	0	0	0	0	0
6902	Pig	Central	0	0	0	0	0	0
6903	Pig	Central	0	0	0	0	0	0
6904	Pig	Central	0	0	0	0	0	0
6905	Pig	Central	0	0	0	0	0	0
6906	Pig	Central	0	0	0	0	0	0
6907	Pig	Central	0	0	0	0	0	0
6908	Pig	Central	0	0	0	0	0	0
6909	Pig	Central	0	0	0	0	0	0
6910	Pig	Central	0	0	0	0	0	0
6911	Pig	Central	0	0	0	0	0	0

6912	Pig	Central	0	0	0	0	0	0
6913	Pig	Central	0	0	0	0	0	0
6914	Pig	Central	0	0	0	0	0	0
6915	Pig	Central	0	0	0	0	0	0
6916	Pig	Central	0	0	0	0	0	0
6917	Pig	Central	0	0	0	0	0	0
6918	Pig	Central	0	0	0	0	0	0
6919	Pig	Central	0	0	0	0	0	0
6920	Pig	Central	0	0	0	1	0	1
6921	Pig	Central	0	0	0	0	0	0
6922	Pig	Central	0	0	0	0	0	0
6923	Pig	Central	0	0	0	0	0	0
6924	Pig	Central	0	0	0	0	0	0
6925	Pig	Central	1	1	0	1	0	1
6926	Pig	Central	0	0	0	0	0	0
6927	Pig	Central	0	0	0	0	0	0
6928	Pig	Central	0	0	0	0	0	0
6929	Pig	Central	0	0	0	0	0	0
6930	Pig	Central	0	0	0	0	0	0
6931	Pig	Central	0	0	0	0	0	0
6932	Pig	Central	0	0	0	0	0	0
6933	Pig	Central	0	0	0	0	0	0
6934	Pig	Central	0	0	0	0	0	0
6935	Pig	Central	0	0	0	0	0	0
6936	Pig	Central	0	0	0	0	0	0
6937	Pig	Central	0	0	0	0	0	0
6938	Pig	Central	0	0	0	0	0	0
6939	Pig	Central	0	0	0	0	0	0
6940	Pig	Central	0	0	0	0	0	0
6941	Pig	Central	0	0	0	0	0	0
6942	Pig	Central	0	0	0	0	0	0
6943	Pig	Central	0	0	0	0	0	0
6944	Pig	Central	0	0	0	1	0	1
6945	Pig	Central	0	0	0	0	0	0
6946	Pig	Central	0	0	0	0	0	0
6947	Pig	Central	0	0	0	0	0	0
6948	Pig	Central	0	0	0	0	0	0
6949	Pig	Central	0	0	0	0	0	0
6950	Pig	Central	0	0	0	0	0	0
6951	Pig	Central	0	0	0	1	0	1
6952	Pig	Central	0	0	0	0	0	0
6953	Pig	Central	0	0	0	0	0	0
6954	Pig	Central	0	0	0	0	0	0
6955	Pig	Central	0	0	0	0	0	0
6956	Pig	Central	0	0	0	1	0	1
6957	Pig	Central	0	0	0	0	0	0
6958	Pig	Central	0	0	0	0	0	0
6959	Pig	Central	0	0	0	0	0	0

6960	Pig	Central	0	0	0	0	0	0
6961	Pig	Central	0	0	0	0	0	0
6962	Pig	Central	0	0	0	0	0	0
6963	Pig	Central	0	0	0	0	0	0
6964	Pig	Central	0	0	0	0	0	0
6965	Pig	Central	0	0	0	0	0	0
6966	Pig	Central	0	0	0	0	0	0
6967	Pig	Central	0	0	0	0	0	0
6968	Pig	Central	0	0	0	0	0	0
6969	Pig	Central	0	0	0	0	0	0
6970	Pig	Central	0	0	0	0	0	0
6971	Pig	Central	0	0	0	0	0	0
6972	Pig	Central	0	0	0	0	0	0
6973	Pig	Central	0	0	0	0	0	0
6974	Pig	Central	0	0	0	0	0	0
6975	Pig	Central	0	0	0	0	0	0
6976	Pig	Central	0	0	0	0	0	0
6977	Pig	Central	0	0	0	0	0	0
6978	Pig	Central	0	0	0	0	0	0
6979	Pig	Central	0	0	0	0	0	0
6980	Pig	Central	0	0	0	0	0	0
6981	Pig	Central	0	0	0	0	0	0
6982	Pig	Central	0	0	0	0	0	0
6983	Pig	Central	0	0	0	1	0	1
6984	Pig	Central	0	0	0	0	0	0
6985	Pig	Central	0	0	0	0	0	0
6986	Pig	Central	0	0	0	0	0	0
6987	Pig	Central	0	0	0	0	0	0
6988	Pig	Central	0	0	0	0	0	0
6989	Pig	Central	0	0	0	0	0	0
6990	Pig	Central	0	0	0	0	0	0
6991	Pig	Central	0	0	0	0	0	0
6992	Pig	Central	0	0	0	1	0	1
6993	Pig	Central	0	0	0	1	0	1
6994	Pig	Central	0	0	0	0	0	0
6995	Pig	Central	0	0	0	0	0	0
6996	Pig	Central	0	0	0	0	0	0
6997	Pig	Central	0	0	0	0	0	0
6998	Pig	Central	0	0	0	0	0	0
6999	Pig	Central	0	0	0	0	0	0
7000	Pig	Central	0	0	0	0	0	0
7001	Pig	Central	0	0	0	0	0	0
7002	Pig	Central	0	0	0	0	0	0
7003	Pig	Central	0	0	0	0	0	0
7004	Pig	Central	0	0	0	0	0	0
7005	Pig	Central	0	0	0	0	0	0
7006	Pig	Central	0	0	0	0	0	0
7007	Pig	Central	0	0	0	0	0	0

7008	Pig	Central	0	0	0	0	0	0
7009	Pig	Central	0	0	0	0	0	0
7010	Pig	Central	0	0	0	0	0	0
7011	Pig	Central	0	0	0	0	0	0
7012	Pig	Central	0	0	0	0	0	0
7013	Pig	Central	0	0	0	1	0	1
7014	Pig	Central	0	0	0	1	0	1
7015	Pig	Central	0	0	0	0	0	0
7016	Pig	Central	0	0	0	0	0	0
7017	Pig	Central	0	0	0	0	0	0
7018	Pig	Central	0	0	0	1	0	1
7019	Pig	Central	0	0	0	0	0	0
7020	Pig	Central	0	0	0	0	0	0
7021	Pig	Central	0	0	0	0	0	0
7022	Pig	Central	0	0	0	0	0	0
7023	Pig	Central	0	0	0	0	0	0
7024	Pig	Central	0	0	0	0	0	0
7025	Pig	Central	0	0	0	0	0	0
7026	Pig	Central	0	0	0	0	0	0
7027	Pig	Central	0	0	0	0	0	0
7028	Pig	Central	0	0	0	0	0	0
7029	Pig	Central	0	0	0	0	0	0
7030	Pig	Central	0	0	0	0	0	0
7031	Pig	Central	0	0	0	0	0	0
7032	Pig	Northeastern	0	0	0	0	0	0
7033	Pig	Northeastern	0	0	0	0	0	0
7034	Pig	Northeastern	0	0	0	0	0	0
7035	Pig	Northeastern	0	0	0	0	0	0
7036	Pig	Northeastern	0	0	0	1	0	1
7037	Pig	Northeastern	0	0	0	0	0	0
7038	Pig	Northeastern	0	0	0	0	0	0
7039	Pig	Northeastern	0	0	0	0	0	0
7040	Pig	Northeastern	0	0	0	0	0	0
7041	Pig	Northeastern	0	0	0	0	0	0
7042	Pig	Northeastern	0	0	0	0	0	0
7043	Pig	Northeastern	0	0	0	1	0	1
7044	Pig	Northeastern	0	0	0	1	0	1
7045	Pig	Northeastern	0	0	0	0	0	0
7046	Pig	Northeastern	0	0	0	0	0	0
7047	Pig	Northeastern	0	0	0	0	0	0
7048	Pig	Northeastern	0	0	0	0	0	0
7049	Pig	Northeastern	0	0	0	0	0	0
7050	Pig	Northeastern	0	0	0	0	0	0
7051	Pig	Northeastern	0	0	0	0	0	0
7052	Pig	Northeastern	0	0	0	0	0	0
7053	Pig	Northeastern	0	0	0	0	0	0
7054	Pig	Northeastern	0	0	0	0	0	0
7055	Pig	Northeastern	0	0	0	0	0	0

7056	Pig	Northeastern	0	0	0	0	0	0
7057	Pig	Northeastern	0	0	0	0	0	0
7058	Pig	Northeastern	0	0	0	0	0	0
7059	Pig	Northeastern	0	0	0	0	0	0
7060	Pig	Northeastern	0	0	0	0	0	0
7061	Pig	Northeastern	0	0	0	0	0	0
7062	Pig	Northeastern	0	0	0	0	0	0
7063	Pig	Northeastern	0	0	0	0	0	0
7064	Pig	Northeastern	0	0	0	1	0	1
7065	Pig	Northeastern	0	0	0	1	0	1
7066	Pig	Northeastern	0	0	0	1	0	1
7067	Pig	Northeastern	0	0	0	0	0	0
7068	Pig	Northeastern	0	0	0	1	0	1
7069	Pig	Northeastern	0	0	0	0	0	0
7070	Pig	Northeastern	0	0	0	0	0	0
7071	Pig	Northeastern	0	0	0	0	0	0
7072	Pig	Northeastern	0	0	0	1	0	1
7073	Pig	Northeastern	0	0	0	1	0	1
7074	Pig	Northeastern	0	0	0	1	0	1
7075	Pig	Northeastern	0	0	0	0	0	0
7076	Pig	Northeastern	0	0	1	1	0	1
7077	Pig	Northeastern	0	0	0	0	0	0
7078	Pig	Northeastern	0	0	0	0	0	0
7079	Pig	Northeastern	0	0	0	0	0	0
7080	Pig	Northeastern	0	0	0	0	0	0
7081	Pig	Northeastern	0	0	1	1	0	1
7082	Pig	Northeastern	0	0	0	0	0	0
7083	Pig	Northeastern	0	0	0	0	0	0
7084	Pig	Northeastern	0	0	0	0	0	0
7085	Pig	Northeastern	0	0	0	0	0	0
7086	Pig	Northeastern	0	0	0	0	0	0
7087	Pig	Northeastern	0	0	0	0	0	0
7088	Pig	Northeastern	0	0	0	1	0	1
7089	Pig	Northeastern	0	0	0	0	0	0
7090	Pig	Central	0	0	0	0	0	0
7091	Pig	Central	0	0	0	0	0	0
7092	Pig	Central	0	0	0	0	0	0
7093	Pig	Central	0	0	0	0	0	0
7094	Pig	Central	0	0	0	0	0	0
7095	Pig	Central	0	0	0	0	0	0
7096	Pig	Central	0	0	0	0	0	0
7097	Pig	Central	0	0	0	0	0	0
7098	Pig	Central	0	0	0	0	0	0
7099	Pig	Central	0	0	0	0	0	0
7100	Pig	Central	0	0	0	0	0	0
7101	Pig	Central	0	0	0	0	0	0
7102	Pig	Central	0	0	0	0	0	0
7103	Pig	Central	0	0	0	0	0	0

7104	Pig	Central	0	0	0	0	0	0
7105	Pig	Central	0	0	0	0	0	0
7106	Pig	Central	0	0	0	0	0	0
7107	Pig	Central	0	0	0	0	0	0
7108	Pig	Central	0	0	0	0	0	0
7109	Pig	Central	0	0	0	0	0	0
7110	Pig	Central	0	0	0	0	0	0
7111	Pig	Central	0	0	0	0	0	0
7112	Pig	Central	0	0	0	0	0	0
7113	Pig	Central	0	0	0	0	0	0
7114	Pig	Central	0	0	0	0	0	0
7115	Pig	Central	0	0	0	0	0	0
7116	Pig	Central	0	0	0	0	0	0
7117	Pig	Central	0	0	0	0	0	0
7118	Pig	Central	0	0	0	0	0	0
7119	Pig	Central	0	0	0	0	0	0
7120	Pig	Eastern	0	0	0	0	0	0
7121	Pig	Eastern	0	0	0	0	0	0
7122	Pig	Eastern	0	0	0	0	0	0
7123	Pig	Eastern	0	0	0	0	0	0
7124	Pig	Eastern	0	0	0	0	0	0
7125	Pig	Eastern	0	0	0	0	0	0
7126	Pig	Eastern	0	0	0	0	0	0
7127	Pig	Eastern	0	0	0	0	0	0
7128	Pig	Eastern	0	0	0	0	0	0
7129	Pig	Eastern	0	0	0	0	0	0
7130	Pig	Eastern	0	0	0	0	0	0
7131	Pig	Eastern	0	0	0	0	0	0
7132	Pig	Eastern	0	0	0	0	0	0
7133	Pig	Eastern	0	0	0	0	0	0
7134	Pig	Eastern	0	0	0	0	0	0
7135	Pig	Eastern	0	0	0	0	0	0
7136	Pig	Eastern	0	0	0	0	0	0
7137	Pig	Eastern	0	0	0	0	0	0
7138	Pig	Eastern	0	0	0	0	0	0
7139	Pig	Eastern	0	0	0	0	0	0
7140	Pig	Eastern	0	0	0	0	0	0
7141	Pig	Eastern	0	0	0	0	0	0
7142	Pig	Eastern	0	0	0	0	0	0
7143	Pig	Eastern	0	0	0	0	0	0
7144	Pig	Eastern	0	0	0	0	0	0
7145	Pig	Eastern	0	0	0	0	0	0
7146	Pig	Eastern	0	0	0	0	0	0
7147	Pig	Eastern	0	0	0	0	0	0
7148	Pig	Eastern	0	0	0	0	0	0
7149	Pig	Eastern	0	0	0	0	0	0
7150	Pig	Eastern	0	0	0	0	0	0
7151	Pig	Eastern	0	0	0	0	0	0

7152	Pig	Eastern	0	0	0	0	0	0
7153	Pig	Eastern	0	0	0	0	0	0
7154	Pig	Eastern	0	0	0	0	0	0
7155	Pig	Eastern	0	0	0	0	0	0
7156	Pig	Eastern	0	0	0	0	0	0
7157	Pig	Eastern	0	0	0	0	0	0
7158	Pig	Eastern	0	0	0	0	0	0
7159	Pig	Eastern	0	0	0	0	0	0
7160	Pig	Eastern	0	0	0	0	0	0
7161	Pig	Eastern	0	0	0	0	0	0
7162	Pig	Eastern	0	0	0	0	0	0
7163	Pig	Eastern	0	0	0	0	0	0
7164	Pig	Central	0	0	0	0	0	0
7165	Pig	Central	0	0	0	0	0	0
7166	Pig	Central	0	0	0	0	0	0
7167	Pig	Central	0	0	0	0	0	1
7168	Pig	Central	1	0	0	0	0	1
7169	Pig	Central	0	0	0	0	0	0
7170	Pig	Central	0	0	0	0	0	1
7171	Pig	Central	0	0	0	0	0	0
7172	Pig	Central	0	0	0	0	0	0
7173	Pig	Central	0	0	0	0	0	0
7174	Pig	Central	0	0	0	0	0	0
7175	Pig	Central	0	0	0	0	0	0
7176	Pig	Central	0	0	0	0	0	0
7177	Pig	Central	0	0	0	0	0	0
7178	Pig	Central	0	0	0	0	0	0
7179	Pig	Central	0	0	0	0	0	0
7180	Pig	Central	0	0	0	0	0	0
7181	Pig	Central	0	0	0	0	0	0
7182	Pig	Central	0	0	0	0	0	0
7183	Pig	Central	0	0	0	0	0	0
7184	Pig	Central	0	0	0	0	0	0
7185	Pig	Central	0	0	0	0	0	0
7186	Pig	Central	0	0	0	0	0	0
7187	Pig	Central	0	0	0	0	0	0
7188	Pig	Central	0	0	0	0	0	0
7189	Pig	Central	0	0	0	0	0	0
7190	Pig	Central	0	0	0	0	0	0
7191	Pig	Central	0	0	0	0	0	0
7192	Pig	Central	0	0	0	0	0	0
7193	Pig	Central	0	0	0	0	0	0
7194	Pig	Central	0	0	0	0	0	0
7195	Pig	Central	0	0	0	0	0	0
7196	Pig	Central	0	0	0	0	0	0
7197	Pig	Central	0	0	0	0	0	0
7198	Pig	Central	0	0	0	0	0	0
7199	Pig	Central	0	0	0	0	0	0

7200	Pig	Central	0	0	0	0	0	0
7201	Pig	Central	0	0	0	0	0	0
7202	Pig	Central	0	0	0	0	0	0
7203	Pig	Central	0	0	0	0	0	0
7204	Pig	Central	0	0	0	0	0	0
7205	Pig	Central	0	0	0	0	0	0
7206	Pig	Central	0	0	0	0	0	0
7207	Pig	Central	0	0	0	0	0	0
7208	Pig	Central	0	0	0	0	0	0
7209	Pig	Central	0	0	0	0	0	0
7210	Pig	Central	0	0	0	0	0	0
7211	Pig	Central	0	0	0	0	0	0
7212	Pig	Central	0	0	0	0	0	0
7213	Pig	Central	0	0	0	0	0	0
7214	Pig	Central	0	0	0	0	0	0
7215	Pig	Central	0	0	0	0	0	0
7216	Pig	Central	0	0	0	0	0	0
7217	Pig	Central	0	0	0	0	0	0
7218	Pig	Central	0	0	0	0	0	0
7219	Pig	Central	0	0	0	0	0	0
7220	Pig	Central	0	0	0	0	0	0
7221	Pig	Central	0	0	0	0	0	0
7222	Pig	Central	0	0	0	0	0	0
7223	Pig	Central	0	0	0	0	0	0
7224	Pig	Central	0	0	0	0	0	0
7225	Pig	Central	0	0	0	0	0	0
7226	Pig	Central	0	0	0	0	0	0
7227	Pig	Central	0	0	0	0	0	0
7228	Pig	Central	0	0	0	0	0	0
7229	Pig	Central	0	0	0	0	0	0
7230	Pig	Central	0	0	0	0	0	0
7231	Pig	Central	0	0	0	0	0	0
7232	Pig	Central	0	0	0	0	0	0
7233	Pig	Central	0	0	0	0	0	0
7234	Pig	Central	0	0	0	0	0	0
7235	Pig	Central	0	0	0	0	0	0
7236	Pig	Central	0	0	0	0	0	0
7237	Pig	Central	0	0	0	0	0	0
7238	Pig	Central	0	0	0	0	0	0
7239	Pig	Central	0	0	0	0	0	0
7240	Pig	Central	0	0	0	0	0	0
7241	Pig	Central	0	0	0	0	0	0
7242	Pig	Southern	0	0	0	0	0	0
7243	Pig	Southern	0	0	0	0	0	0
7244	Pig	Southern	0	0	0	0	0	0
7245	Pig	Southern	0	0	0	0	0	0
7246	Pig	Southern	0	0	0	0	0	0
7247	Pig	Southern	0	0	0	0	0	0

7248	Pig	Southern	0	0	0	0	0	0
7249	Pig	Southern	0	0	0	0	0	0
7250	Pig	Southern	0	0	0	0	0	0
7251	Pig	Southern	0	0	0	0	0	0
7252	Pig	Southern	0	0	0	0	0	0
7253	Pig	Southern	0	0	0	0	0	0
7254	Pig	Southern	0	0	0	0	0	0
7255	Pig	Southern	0	0	0	0	0	0
7256	Pig	Southern	0	0	0	0	0	0
7257	Pig	Southern	0	0	0	0	0	0
7258	Pig	Southern	0	0	0	0	0	0
7259	Pig	Southern	0	0	0	0	0	0
7260	Pig	Southern	0	0	0	0	0	0
7261	Pig	Southern	0	0	0	0	0	0
7262	Pig	Southern	0	0	0	0	0	0
7263	Pig	Southern	0	0	0	0	0	0
7264	Pig	Southern	0	0	0	0	0	0
7265	Pig	Southern	0	0	0	0	0	0
7266	Pig	Southern	0	0	0	0	0	0
7267	Pig	Southern	0	0	0	0	0	0
7268	Pig	Southern	0	0	0	0	0	0
7269	Pig	Southern	0	0	0	0	0	0
7270	Pig	Southern	0	0	0	0	0	0
7271	Pig	Southern	0	0	0	0	0	0
7272	Pig	Eastern	0	0	1	1	0	1
7273	Pig	Eastern	0	0	0	1	0	1
7274	Pig	Eastern	0	0	0	1	0	1
7275	Pig	Eastern	0	0	0	0	0	0
7276	Pig	Eastern	0	0	0	1	0	1
7277	Pig	Eastern	0	0	0	0	0	0
7278	Pig	Eastern	0	0	0	0	0	0
7279	Pig	Eastern	0	0	0	0	0	0
7280	Pig	Eastern	0	0	0	0	0	0
7281	Pig	Eastern	0	0	0	1	0	1
7282	Pig	Eastern	0	0	0	0	0	0
7283	Pig	Eastern	0	0	0	1	0	1
7284	Pig	Eastern	0	0	0	0	0	0
7285	Pig	Eastern	0	0	0	0	0	0
7286	Pig	Eastern	0	0	0	0	0	0
7287	Pig	Eastern	0	0	0	0	0	0
7288	Pig	Eastern	0	0	0	0	0	0
7289	Pig	Eastern	0	0	0	0	0	0
7290	Pig	Eastern	0	0	0	0	0	0
7291	Pig	Eastern	0	0	0	0	0	0
7292	Pig	Eastern	0	0	0	0	0	0
7293	Pig	Eastern	0	0	0	0	0	0
7294	Pig	Eastern	0	0	0	0	0	0
7295	Pig	Eastern	0	0	0	0	0	0

7296	Pig	Eastern	0	0	0	0	0	0
7297	Pig	Eastern	0	0	0	0	0	0
7298	Pig	Eastern	0	0	0	0	0	0
7299	Pig	Eastern	0	0	0	0	0	0
7300	Pig	Eastern	0	0	0	0	0	0
7301	Pig	Eastern	0	0	0	0	0	0
7302	Pig	Central	0	0	0	0	0	0
7303	Pig	Central	0	0	0	0	0	0
7304	Pig	Central	0	0	0	0	0	0
7305	Pig	Central	0	0	0	0	0	0
7306	Pig	Central	0	0	0	0	0	0
7307	Pig	Central	0	0	0	0	0	0
7308	Pig	Central	0	0	0	0	0	0
7309	Pig	Central	0	0	0	0	0	0
7310	Pig	Central	0	0	0	0	0	0
7311	Pig	Central	0	0	0	0	0	0
7312	Pig	Central	0	0	0	0	0	0
7313	Pig	Central	0	0	0	0	0	0
7314	Pig	Central	0	0	0	0	0	0
7315	Pig	Central	0	0	0	0	0	0
7316	Pig	Central	0	0	0	0	0	0
7317	Pig	Central	0	0	0	0	0	0
7318	Pig	Central	0	0	0	0	0	0
7319	Pig	Central	0	0	1	1	0	1
7320	Pig	Central	0	0	0	0	0	0
7321	Pig	Central	0	0	0	0	0	0
7322	Pig	Central	0	0	0	1	0	1
7323	Pig	Central	0	0	0	0	0	0
7324	Pig	Central	0	0	0	0	0	0
7325	Pig	Central	0	0	0	0	0	0
7326	Pig	Central	0	0	0	0	0	0
7327	Pig	Central	0	0	0	0	0	0
7328	Pig	Central	0	0	0	0	0	0
7329	Pig	Central	0	0	0	1	0	1
7330	Pig	Central	0	0	0	0	0	0
7331	Pig	Central	0	0	0	0	0	0
7332	Pig	Central	0	0	0	0	0	0
7333	Pig	Central	0	0	0	0	0	0
7334	Pig	Central	0	0	0	0	0	0
7335	Pig	Central	0	0	0	0	0	0
7336	Pig	Central	0	0	0	0	0	0
7337	Pig	Central	0	0	0	0	0	0
7338	Pig	Central	1	0	0	0	0	1
7339	Pig	Central	0	0	0	0	0	0
7340	Pig	Central	1	0	0	0	0	1
7341	Pig	Central	0	0	0	0	0	0
7342	Pig	Central	0	0	0	0	0	0
7343	Pig	Central	0	0	0	1	0	1

7344	Pig	Central	0	0	0	0	0	0
7345	Pig	Central	0	0	0	0	0	0
7346	Pig	Central	0	0	0	1	0	1
7347	Pig	Central	0	0	0	0	0	0
7348	Pig	Central	0	0	0	0	0	0
7349	Pig	Central	0	0	0	0	0	0
7350	Pig	Central	0	0	0	0	0	0
7351	Pig	Central	0	0	0	0	0	0
7352	Pig	Central	0	0	0	0	0	0
7353	Pig	Central	0	0	0	1	0	1
7354	Pig	Central	0	0	0	0	0	0
7355	Pig	Central	0	0	0	0	0	0
7356	Pig	Central	0	0	0	0	0	0
7357	Pig	Central	0	0	0	0	0	0
7358	Pig	Central	0	0	0	0	0	0
7359	Pig	Central	0	0	0	0	0	0
7360	Pig	Central	0	0	0	0	0	0
7361	Pig	Central	0	0	0	0	0	0
7362	Pig	Central	0	0	0	0	0	0
7363	Pig	Central	0	0	0	0	0	0
7364	Pig	Central	0	0	0	0	0	0
7365	Pig	Central	0	0	0	0	0	0
7366	Pig	Central	0	0	0	0	0	0
7367	Pig	Central	0	0	0	1	0	1
7368	Pig	Central	0	0	0	0	0	0
7369	Pig	Central	0	0	0	0	0	0
7370	Pig	Central	0	0	0	0	0	0
7371	Pig	Central	0	0	0	0	0	0
7372	Pig	Central	0	0	0	0	0	1
7373	Pig	Central	0	0	0	0	0	0
7374	Pig	Central	0	0	0	0	0	0
7375	Pig	Central	0	0	0	0	0	0
7376	Pig	Central	0	0	0	0	0	0
7377	Pig	Central	0	0	0	1	0	1
7378	Pig	Central	0	0	0	1	0	1
7379	Pig	Central	0	0	0	0	0	0
7380	Pig	Central	0	0	0	1	0	1
7381	Pig	Central	0	0	0	0	0	0
7382	Pig	Central	0	0	0	0	0	0
7383	Pig	Central	0	0	0	1	0	1
7384	Pig	Central	0	0	0	0	0	0
7385	Pig	Central	0	0	0	0	0	0
7386	Pig	Central	0	0	0	0	0	0
7387	Pig	Central	1	0	0	1	0	1
7388	Pig	Central	0	0	0	0	0	0
7389	Pig	Central	0	0	0	0	0	0
7390	Pig	Central	0	0	0	0	0	0
7391	Pig	Central	0	0	0	1	0	1

7392	Pig	Central	0	0	0	0	0	0
7393	Pig	Central	0	0	0	1	0	1
7394	Pig	Central	0	0	0	0	0	0
7395	Pig	Central	0	0	0	0	0	0
7396	Pig	Central	0	0	0	0	0	0
7397	Pig	Central	1	0	0	1	0	1
7398	Pig	Central	0	0	0	0	0	0
7399	Pig	Central	0	0	0	0	0	0
7400	Pig	Central	0	0	0	1	0	1
7401	Pig	Central	0	0	0	0	0	0
7402	Pig	Central	0	0	0	0	0	0
7403	Pig	Central	0	0	0	1	0	1
7404	Pig	Central	0	0	0	0	0	0
7405	Pig	Central	0	0	0	0	0	0
7406	Pig	Central	0	0	0	1	0	1
7407	Pig	Central	0	0	0	0	0	0
7408	Pig	Central	0	0	0	0	0	0
7409	Pig	Central	0	0	0	0	0	0
7410	Pig	Central	0	0	0	1	0	1
7411	Pig	Central	0	0	0	1	0	1
7412	Pig	Central	0	0	0	0	0	0
7413	Pig	Central	0	0	0	1	0	1
7414	Pig	Central	0	0	0	1	0	1
7415	Pig	Central	0	0	0	1	0	1
7416	Pig	Central	0	0	0	1	0	1
7417	Pig	Central	0	0	0	1	0	1
7418	Pig	Central	0	0	0	0	0	0
7419	Pig	Central	0	0	0	0	0	0
7420	Pig	Central	0	0	0	1	0	1
7421	Pig	Central	0	0	0	0	0	0
7422	Pig	Central	0	0	0	0	0	0
7423	Pig	Central	0	0	0	0	0	0
7424	Pig	Central	0	0	0	0	0	0
7425	Pig	Central	0	0	0	0	0	0
7426	Pig	Central	0	0	0	0	0	0
7427	Pig	Central	0	0	0	0	0	0
7428	Pig	Central	0	0	0	0	0	0
7429	Pig	Central	0	0	0	0	0	0
7430	Pig	Central	0	0	0	0	0	0
7431	Pig	Central	0	0	0	0	0	0
7432	Pig	Central	0	0	0	0	0	0
7433	Pig	Central	0	0	0	0	0	0
7434	Pig	Central	0	0	0	0	0	0
7435	Pig	Central	0	0	0	0	0	0
7436	Pig	Central	0	0	0	0	0	0
7437	Pig	Central	0	0	0	0	0	0
7438	Pig	Central	0	0	0	0	0	0
7439	Pig	Central	0	0	0	0	0	0

7440	Pig	Central	0	0	0	0	0	0
7441	Pig	Central	0	0	0	0	0	0
7442	Pig	Central	0	0	0	0	0	0
7443	Pig	Central	0	0	0	0	0	0
7444	Pig	Central	0	0	0	0	0	0
7445	Pig	Central	0	0	0	0	0	0
7446	Pig	Central	0	0	0	0	0	0
7447	Pig	Central	0	0	0	1	0	1
7448	Pig	Central	0	0	0	0	0	0
7449	Pig	Central	0	0	0	0	0	0
7450	Pig	Central	0	0	0	0	0	0
7451	Pig	Central	0	0	0	1	0	1
7452	Pig	Central	0	0	0	0	0	0
7453	Pig	Central	0	0	0	1	0	1
7454	Pig	Central	0	0	0	1	0	1
7455	Pig	Central	0	0	0	0	0	0
7456	Pig	Central	0	0	0	1	0	1
7457	Pig	Central	0	0	0	1	0	1
7458	Pig	Central	0	0	0	0	0	0
7459	Pig	Central	0	0	0	0	0	0
7460	Pig	Central	0	0	0	1	0	1
7461	Pig	Central	0	0	1	1	0	1
7462	Pig	Central	0	0	0	0	0	0
7463	Pig	Central	0	0	0	1	0	1
7464	Pig	Central	0	0	0	0	0	0
7465	Pig	Central	1	0	1	1	0	1
7466	Pig	Central	0	0	0	0	0	0
7467	Pig	Central	0	0	0	1	0	1
7468	Pig	Central	0	0	0	1	0	1
7469	Pig	Central	0	0	0	1	0	1
7470	Pig	Central	0	0	0	0	0	0
7471	Pig	Central	0	0	0	0	0	0
7472	Pig	Central	0	0	1	1	0	1
7473	Pig	Central	0	0	0	1	0	1
7474	Pig	Central	0	0	0	0	0	0
7475	Pig	Central	0	0	0	0	0	0
7476	Pig	Central	0	0	1	1	0	1
7477	Pig	Central	0	0	0	1	0	1
7478	Pig	Central	0	0	0	1	0	1
7479	Pig	Central	0	0	0	1	0	1
7480	Pig	Central	0	0	0	1	0	1
7481	Pig	Central	0	0	1	1	0	1
7482	Pig	Central	0	0	1	1	0	1
7483	Pig	Central	0	0	0	0	0	0
7484	Pig	Central	0	0	0	0	0	0
7485	Pig	Central	0	0	0	0	0	0
7486	Pig	Central	0	0	0	0	0	0
7487	Pig	Central	0	0	0	1	0	1

7488	Pig	Central	0	0	0	1	0	1
7489	Pig	Central	0	0	0	1	0	1
7490	Pig	Central	0	0	0	0	0	0
7491	Pig	Central	0	0	1	1	0	1
7492	Pig	Central	0	0	1	1	0	1
7493	Pig	Central	0	0	1	1	0	1
7494	Pig	Central	0	0	1	1	0	1
7495	Pig	Central	0	0	0	1	0	1
7496	Pig	Central	0	0	0	1	0	1
7497	Pig	Central	0	0	0	1	0	1
7498	Pig	Central	0	0	1	1	0	1
7499	Pig	Central	0	0	0	0	0	0
7500	Pig	Central	0	0	0	0	0	0
7501	Pig	Central	0	0	0	0	0	0
7502	Pig	Central	0	0	0	0	0	0
7503	Pig	Central	0	0	0	0	0	0
7504	Pig	Central	0	0	0	0	0	0
7505	Pig	Central	0	0	0	0	0	0
7506	Pig	Central	0	0	1	1	0	1
7507	Pig	Central	0	0	0	0	0	0
7508	Pig	Central	0	0	1	1	0	1
7509	Pig	Central	0	0	0	1	0	1
7510	Pig	Central	0	0	1	1	0	1
7511	Pig	Central	0	0	1	1	0	1
7512	Pig	Central	0	0	1	1	0	1
7513	Pig	Central	0	0	1	1	0	1
7514	Pig	Central	0	0	0	0	0	0
7515	Pig	Central	0	0	1	1	0	1
7516	Pig	Central	0	0	0	0	0	0
7517	Pig	Central	0	0	0	1	0	1
7518	Pig	Central	0	0	1	1	0	1
7519	Pig	Central	0	0	1	1	0	1
7520	Pig	Central	0	0	1	0	0	1
7521	Pig	Central	0	0	1	1	0	1
7522	Pig	Central	0	0	0	0	0	0
7523	Pig	Central	0	0	0	0	0	0
7524	Pig	Central	0	0	0	0	0	0
7525	Pig	Central	0	0	0	1	0	1
7526	Pig	Central	0	0	1	1	0	1
7527	Pig	Central	0	0	0	1	0	1
7528	Pig	Central	0	0	0	1	0	1
7529	Pig	Central	0	0	0	0	0	0
7530	Pig	Central	0	0	0	0	0	0
7531	Pig	Central	0	0	0	1	0	1
7532	Pig	Central	0	0	0	0	0	0
7533	Pig	Central	0	0	0	0	0	0
7534	Pig	Central	0	0	0	1	0	1
7535	Pig	Central	0	0	0	0	0	0

7536	Pig	Central	0	0	0	1	0	1
7537	Pig	Central	0	0	0	1	0	1
7538	Pig	Central	0	0	0	1	0	1
7539	Pig	Central	0	0	0	0	0	0
7540	Pig	Central	0	0	0	0	0	0
7541	Pig	Central	0	0	0	1	0	1
7542	Pig	Central	0	0	0	0	0	0
7543	Pig	Central	0	0	0	0	0	0
7544	Pig	Central	0	0	0	0	0	0
7545	Pig	Central	0	0	0	0	0	0
7546	Pig	Central	0	0	0	0	0	0
7547	Pig	Central	0	0	0	0	0	1
7548	Pig	Central	0	0	0	0	0	0
7549	Pig	Central	0	0	0	0	0	0
7550	Pig	Central	0	0	0	0	0	0
7551	Pig	Central	0	0	0	1	0	1
7552	Pig	Central	0	0	0	0	0	0
7553	Pig	Central	0	0	0	0	0	0
7554	Pig	Central	0	0	0	0	0	0
7555	Pig	Central	0	0	0	0	0	0
7556	Pig	Central	0	0	0	0	0	0
7557	Pig	Central	0	0	1	1	0	1
7558	Pig	Central	0	0	0	0	0	0
7559	Pig	Central	0	0	1	1	0	1
7560	Pig	Central	0	0	0	1	0	1
7561	Pig	Central	0	0	0	1	0	1
7562	Pig	Central	0	0	1	1	0	1
7563	Pig	Central	0	0	0	1	0	1
7564	Pig	Central	0	0	0	1	0	1
7565	Pig	Central	0	0	0	0	0	0
7566	Pig	Central	0	0	0	0	0	0
7567	Pig	Central	0	0	0	1	0	1
7568	Pig	Central	0	0	0	0	0	0
7569	Pig	Central	0	0	0	0	0	0
7570	Pig	Central	0	0	0	0	0	0
7571	Pig	Central	0	0	0	0	0	0
7572	Pig	Central	0	0	0	0	0	0
7573	Pig	Central	0	0	0	0	0	0
7574	Pig	Central	0	0	0	1	0	1
7575	Pig	Central	0	0	0	1	0	1
7576	Pig	Central	0	0	0	0	0	0
7577	Pig	Central	0	0	0	1	0	1
7578	Pig	Central	0	0	0	0	0	0
7579	Pig	Central	0	0	0	1	0	1
7580	Pig	Central	0	0	0	1	0	1
7581	Pig	Central	0	0	0	1	0	1
7582	Pig	Central	0	0	0	1	0	1
7583	Pig	Central	0	0	0	0	0	0

7584	Pig	Central	0	0	1	1	0	1
7585	Pig	Central	0	0	0	0	0	0
7586	Pig	Central	0	0	0	0	0	0
7587	Pig	Central	0	0	0	0	0	0
7588	Pig	Central	0	0	0	0	0	0
7589	Pig	Central	0	0	0	0	0	0
7590	Pig	Central	0	0	0	1	0	1
7591	Pig	Central	0	0	0	0	0	0
7592	Pig	Central	0	0	0	0	0	0
7593	Pig	Central	0	0	0	1	0	1
7594	Pig	Central	0	0	0	0	0	0
7595	Pig	Central	0	0	0	0	0	0
7596	Pig	Central	0	0	0	0	0	0
7597	Pig	Central	0	0	0	0	0	0
7598	Pig	Central	0	0	1	1	0	1
7599	Pig	Central	0	0	0	0	0	0
7600	Pig	Central	0	0	0	1	0	1
7601	Pig	Central	0	0	0	0	0	0
7602	Pig	Central	0	0	0	0	0	0
7603	Pig	Central	0	0	0	0	0	0
7604	Pig	Central	0	0	0	0	0	0
7605	Pig	Central	0	0	0	0	0	0
7606	Pig	Central	0	0	0	0	0	0
7607	Pig	Central	0	0	0	0	0	0
7608	Pig	Central	0	0	0	0	0	0
7609	Pig	Central	0	0	0	0	0	0
7610	Pig	Central	0	0	0	0	0	0
7611	Pig	Central	0	0	0	0	0	0
7612	Pig	Central	0	0	0	0	0	0
7613	Pig	Central	0	0	0	0	0	0
7614	Pig	Central	0	0	0	0	0	0
7615	Pig	Central	0	0	0	0	0	0
7616	Pig	Central	0	0	0	0	0	0
7617	Pig	Central	0	0	0	0	0	0
7618	Pig	Central	0	0	0	1	0	1
7619	Pig	Central	0	0	0	1	0	1
7620	Pig	Central	0	0	0	1	0	1
7621	Pig	Central	0	0	0	0	0	0
7622	Pig	Central	0	0	0	1	0	1
7623	Pig	Central	0	0	0	0	0	0
7624	Pig	Central	0	0	1	1	0	1
7625	Pig	Central	0	0	0	0	0	0
7626	Pig	Central	0	0	0	0	0	0
7627	Pig	Central	0	0	0	1	0	1
7628	Pig	Central	0	0	0	0	0	0
7629	Pig	Central	0	0	0	0	0	0
7630	Pig	Central	0	0	0	1	0	1
7631	Pig	Central	0	0	0	1	0	1

7632	Pig	Central	0	0	0	1	0	1
7633	Pig	Central	0	0	0	0	0	0
7634	Pig	Central	0	0	1	1	0	1
7635	Pig	Central	0	0	0	0	0	0
7636	Pig	Central	0	0	0	0	0	0
7637	Pig	Central	0	0	0	1	0	1
7638	Pig	Central	0	0	0	1	0	1
7639	Pig	Central	0	0	0	1	0	1
7640	Pig	Central	0	0	0	1	0	1
7641	Pig	Central	0	0	0	0	0	0
7642	Pig	Central	0	0	0	0	0	0
7643	Pig	Central	0	0	0	0	0	0
7644	Pig	Central	0	0	0	0	0	0
7645	Pig	Central	0	0	0	0	0	0
7646	Pig	Central	0	0	0	0	0	0
7647	Pig	Central	0	0	0	0	0	0
7648	Pig	Central	0	0	0	1	0	1
7649	Pig	Central	0	0	0	0	0	0
7650	Pig	Central	0	0	0	0	0	0
7651	Pig	Central	0	0	0	0	0	0
7652	Pig	Central	0	0	0	0	0	0
7653	Pig	Central	0	0	0	0	0	0
7654	Pig	Central	0	0	0	0	0	0
7655	Pig	Central	0	0	0	0	0	0
7656	Pig	Central	0	0	0	0	0	0
7657	Pig	Central	0	0	0	0	0	0
7658	Pig	Central	0	0	0	1	0	1
7659	Pig	Central	0	0	0	1	0	1
7660	Pig	Central	0	0	1	1	0	1
7661	Pig	Central	0	0	0	1	0	1
7662	Pig	Central	0	0	0	0	0	0
7663	Pig	Central	0	0	0	0	0	0
7664	Pig	Central	0	0	1	0	0	1
7665	Pig	Central	0	1	1	0	0	1
7666	Pig	Central	0	0	0	0	0	0
7667	Pig	Central	1	0	1	0	0	1
7668	Pig	Central	0	0	1	0	0	1
7669	Pig	Central	0	0	1	0	0	1
7670	Pig	Central	0	0	0	0	0	0
7671	Pig	Central	0	0	0	0	0	0
7672	Pig	Central	0	0	0	0	0	0
7673	Pig	Central	0	0	0	0	0	0
7674	Pig	Central	0	0	0	0	0	0
7675	Pig	Central	0	0	0	0	0	0
7676	Pig	Central	0	0	0	0	0	0
7677	Pig	Central	0	0	0	0	0	0
7678	Pig	Central	0	0	0	0	0	0
7679	Pig	Central	0	0	0	0	0	0

7680	Pig	Central	0	0	0	0	0	0
7681	Pig	Central	0	0	0	0	0	0
7682	Pig	Central	0	0	0	0	0	0
7683	Pig	Central	0	0	0	0	0	0
7684	Pig	Central	0	0	0	0	0	0
7685	Pig	Central	0	0	0	0	0	0
7686	Pig	Southern	1	0	0	0	0	1
7687	Pig	Southern	1	0	1	1	0	1
7688	Pig	Southern	0	0	1	1	0	1
7689	Pig	Southern	0	0	0	1	0	1
7690	Pig	Southern	0	0	0	0	0	0
7691	Pig	Southern	0	0	0	1	0	1
7692	Pig	Southern	0	0	0	0	0	0
7693	Pig	Southern	0	0	1	0	0	1
7694	Pig	Southern	0	0	1	0	0	1
7695	Pig	Southern	0	0	0	0	0	0
7696	Pig	Southern	0	0	1	1	0	1
7697	Pig	Southern	0	0	1	1	0	1
7698	Pig	Southern	0	0	1	1	0	1
7699	Pig	Southern	0	0	0	0	0	0
7700	Pig	Southern	0	0	1	1	0	1
7701	Pig	Southern	0	0	1	1	0	1
7702	Pig	Southern	0	0	1	1	0	1
7703	Pig	Southern	0	0	0	0	0	0
7704	Pig	Southern	0	0	0	0	0	0
7705	Pig	Southern	0	0	0	0	0	0
7706	Pig	Southern	0	0	0	1	0	1
7707	Pig	Southern	0	0	0	1	0	1
7708	Pig	Southern	0	0	0	0	0	0
7709	Pig	Southern	1	0	0	0	0	1
7710	Pig	Southern	0	0	0	0	0	0
7711	Pig	Southern	0	0	0	1	0	1
7712	Pig	Southern	0	0	0	1	0	1
7713	Pig	Southern	0	0	1	1	0	1
7714	Pig	Southern	1	0	1	1	0	1
7715	Pig	Southern	0	0	0	1	0	1
7716	Pig	Southern	0	0	0	0	0	0
7717	Pig	Southern	0	0	1	1	0	1
7718	Pig	Southern	0	0	0	0	0	0
7719	Pig	Southern	0	0	0	0	0	0
7720	Pig	Southern	0	0	0	0	0	0
7721	Pig	Southern	0	0	0	0	0	0
7722	Pig	Southern	0	0	0	1	0	1
7723	Pig	Southern	0	0	0	0	0	0
7724	Pig	Southern	0	0	0	0	0	0
7725	Pig	Southern	0	0	0	0	0	0
7726	Pig	Southern	0	0	0	0	0	0
7727	Pig	Southern	0	0	0	0	0	0

7728	Pig	Southern	0	0	0	1	0	1
7729	Pig	Southern	1	0	1	1	0	1
7730	Pig	Southern	0	0	0	0	0	0
7731	Pig	Southern	0	0	0	0	0	0
7732	Pig	Southern	0	0	0	0	0	0
7733	Pig	Southern	0	0	0	0	0	0
7734	Pig	Southern	0	0	0	1	0	1
7735	Pig	Southern	0	0	0	0	0	0
7736	Pig	Southern	0	0	0	0	0	0
7737	Pig	Southern	0	0	0	0	0	0
7738	Pig	Southern	0	0	0	0	0	0
7739	Pig	Southern	0	0	0	0	0	0
7740	Pig	Southern	0	0	0	0	0	0
7741	Pig	Southern	0	0	0	0	0	0
7742	Pig	Southern	0	0	0	0	0	0
7743	Pig	Southern	0	0	0	0	0	0
7744	Pig	Southern	0	0	0	0	0	0
7745	Pig	Southern	0	0	0	0	0	0
7746	Pig	Southern	0	0	1	1	0	1
7747	Pig	Southern	0	0	0	0	0	0
7748	Pig	Southern	0	0	0	0	0	0
7749	Pig	Southern	0	0	0	1	0	1
7750	Pig	Southern	0	0	0	1	0	1
7751	Pig	Southern	0	0	0	0	0	0
7752	Pig	Southern	0	0	0	0	0	0
7753	Pig	Southern	0	0	0	1	0	1
7754	Pig	Southern	0	0	0	0	0	0
7755	Pig	Southern	0	0	0	0	0	0
7756	Pig	Southern	0	0	0	0	0	0
7757	Pig	Southern	0	0	0	0	0	0
7758	Pig	Southern	0	0	0	0	0	0
7759	Pig	Southern	0	0	0	0	0	1
7760	Pig	Southern	0	0	0	0	0	0
7761	Pig	Southern	0	0	0	0	0	0
7762	Pig	Southern	0	0	0	0	0	0
7763	Pig	Southern	0	0	0	0	0	0
7764	Pig	Southern	0	0	0	0	0	0
7765	Pig	Southern	0	0	0	0	0	0
7766	Pig	Southern	0	0	0	0	0	0
7767	Pig	Southern	0	0	0	0	0	0
7768	Pig	Southern	0	0	0	0	0	0
7769	Pig	Southern	0	0	0	0	0	0
7770	Pig	Southern	0	0	0	0	0	0
7771	Pig	Southern	0	0	0	0	0	0
7772	Pig	Southern	0	0	0	0	0	0
7773	Pig	Southern	0	0	0	0	0	0
7774	Pig	Southern	0	0	0	0	0	0
7775	Pig	Southern	1	0	0	0	0	1

7776	Pig	Southern	0	0	0	0	0	0
7777	Pig	Southern	0	0	0	0	0	0
7778	Pig	Southern	0	0	0	0	0	0
7779	Pig	Southern	0	0	0	0	0	0
7780	Pig	Southern	0	0	0	0	0	0
7781	Pig	Southern	0	0	0	0	0	0
7782	Pig	Southern	0	0	0	0	0	0
7783	Pig	Southern	0	0	0	0	0	0
7784	Pig	Southern	0	0	0	0	0	0
7785	Pig	Southern	0	0	0	0	0	0
7786	Pig	Southern	0	0	0	0	0	0
7787	Pig	Southern	0	0	0	0	0	0
7788	Pig	Southern	0	0	0	0	0	0
7789	Pig	Southern	0	0	0	0	0	0
7790	Pig	Southern	0	0	0	0	0	0
7791	Pig	Southern	0	0	0	0	0	0
7792	Pig	Southern	0	0	0	0	0	0
7793	Pig	Southern	0	0	0	0	0	0
7794	Pig	Southern	0	0	0	0	0	0
7795	Pig	Southern	0	0	0	0	0	0
7796	Pig	Southern	0	0	0	0	0	0
7797	Pig	Southern	0	0	0	0	0	0
7798	Pig	Southern	0	0	0	0	0	0
7799	Pig	Southern	0	0	0	0	0	0
7800	Pig	Southern	0	0	0	0	0	0
7801	Pig	Southern	0	0	0	0	0	0
7802	Pig	Southern	0	0	0	0	0	0
7803	Pig	Southern	0	0	1	1	0	1
7804	Pig	Southern	0	0	0	0	0	0
7805	Pig	Southern	0	0	0	0	0	0
7806	Pig	Southern	1	0	0	1	0	1
7807	Pig	Southern	0	0	0	0	0	0
7808	Pig	Southern	0	0	0	0	0	0
7809	Pig	Southern	0	0	0	0	0	0
7810	Pig	Southern	0	0	0	0	0	0
7811	Pig	Southern	0	0	0	0	0	0
7812	Pig	Southern	0	0	0	0	0	0
7813	Pig	Southern	0	0	0	0	0	0
7814	Pig	Southern	0	0	0	0	0	0
7815	Pig	Southern	0	0	0	0	0	0
7816	Pig	Southern	0	0	0	0	0	0
7817	Pig	Southern	0	0	0	1	0	1
7818	Pig	Southern	1	0	0	0	0	1
7819	Pig	Southern	0	0	0	0	0	0
7820	Pig	Southern	0	0	1	0	0	1
7821	Pig	Southern	0	0	0	0	0	0
7822	Pig	Southern	0	0	1	1	0	1
7823	Pig	Southern	0	0	0	0	0	0

7824	Pig	Southern	0	0	0	1	0	1
7825	Pig	Eastern	0	0	0	0	0	0
7826	Pig	Eastern	0	0	0	0	0	0
7827	Pig	Eastern	0	0	0	0	0	0
7828	Pig	Eastern	0	0	0	0	0	0
7829	Pig	Eastern	0	0	0	0	0	0
7830	Pig	Eastern	0	0	0	0	0	0
7831	Pig	Eastern	0	0	0	0	0	0
7832	Pig	Eastern	0	0	0	0	0	0
7833	Pig	Eastern	0	0	0	0	0	0
7834	Pig	Eastern	0	0	0	0	0	0
7835	Pig	Eastern	0	0	0	0	0	0
7836	Pig	Eastern	0	0	0	0	0	0
7837	Pig	Eastern	0	0	0	0	0	0
7838	Pig	Eastern	0	0	0	0	0	0
7839	Pig	Eastern	0	0	0	0	0	0
7840	Pig	Eastern	0	0	0	0	0	0
7841	Pig	Eastern	0	0	0	0	0	0
7842	Pig	Eastern	0	0	0	0	0	0
7843	Pig	Eastern	0	0	0	0	0	0
7844	Pig	Eastern	0	0	0	0	0	0
7845	Pig	Eastern	0	0	0	0	0	0
7846	Pig	Eastern	0	0	0	0	0	0
7847	Pig	Eastern	0	0	0	0	0	0
7848	Pig	Eastern	0	0	0	0	0	0
7849	Pig	Eastern	0	0	0	0	0	0
7850	Pig	Eastern	0	0	0	0	0	0
7851	Pig	Eastern	0	0	0	0	0	0
7852	Pig	Eastern	0	0	0	0	0	0
7853	Pig	Eastern	0	0	0	0	0	0
7854	Pig	Eastern	0	0	0	0	0	0
7855	Pig	Eastern	0	0	0	0	0	0
7856	Pig	Eastern	0	0	0	0	0	0
7857	Pig	Eastern	0	0	0	0	0	0
7858	Pig	Eastern	0	0	0	0	0	0
7859	Pig	Eastern	0	0	0	0	0	0
7860	Pig	Eastern	0	0	0	0	0	0
7861	Pig	Eastern	0	0	0	0	0	0
7862	Pig	Eastern	0	0	0	0	0	0
7863	Pig	Eastern	0	0	0	0	0	0
7864	Pig	Eastern	0	0	0	0	0	0
7865	Pig	Eastern	0	0	0	0	0	0
7866	Pig	Eastern	0	0	0	0	0	0
7867	Pig	Eastern	0	0	0	0	0	0
7868	Pig	Eastern	0	0	0	0	0	0
7869	Pig	Eastern	0	0	0	0	0	0
7870	Pig	Eastern	0	0	0	0	0	0
7871	Pig	Eastern	0	0	0	0	0	0

7872	Pig	Eastern	0	0	0	0	0	0
7873	Pig	Eastern	0	0	0	0	0	0
7874	Pig	Eastern	0	0	0	0	0	0
7875	Pig	Eastern	0	0	0	0	0	0
7876	Pig	Eastern	0	0	0	0	0	0
7877	Pig	Eastern	0	0	0	0	0	0
7878	Pig	Eastern	0	0	0	0	0	0
7879	Pig	Eastern	0	0	0	0	0	0
7880	Pig	Eastern	0	0	0	0	0	0
7881	Pig	Eastern	0	0	0	0	0	0
7882	Pig	Eastern	0	0	0	0	0	0
7883	Pig	Eastern	0	0	0	0	0	0
7884	Pig	Eastern	0	0	0	0	0	0
7885	Pig	Eastern	0	0	0	0	0	0
7886	Pig	Eastern	0	0	0	0	0	0
7887	Pig	Eastern	0	0	0	0	0	0
7888	Pig	Eastern	0	0	0	0	0	0
7889	Pig	Eastern	0	0	0	0	0	0
7890	Pig	Eastern	0	0	0	0	0	0
7891	Pig	Eastern	0	0	0	0	0	0
7892	Pig	Eastern	0	0	0	0	0	0
7893	Pig	Eastern	0	0	0	0	0	0
7894	Pig	Eastern	0	0	0	0	0	0
7895	Pig	Eastern	0	0	0	0	0	0
7896	Pig	Eastern	0	0	0	0	0	0
7897	Pig	Eastern	0	0	0	0	0	0
7898	Pig	Eastern	0	0	0	0	0	0
7899	Pig	Eastern	0	0	0	0	0	0
7900	Pig	Eastern	0	0	0	0	0	0
7901	Pig	Eastern	0	0	0	0	0	0
7902	Pig	Eastern	0	0	0	0	0	0
7903	Pig	Eastern	0	0	0	0	0	0
7904	Pig	Eastern	0	0	0	0	0	0
7905	Pig	Eastern	0	0	0	0	0	0
7906	Pig	Eastern	0	0	0	0	0	0
7907	Pig	Eastern	0	0	0	0	0	0
7908	Pig	Eastern	0	0	0	0	0	0
7909	Pig	Eastern	0	0	0	0	0	0
7910	Pig	Eastern	0	0	0	0	0	0
7911	Pig	Eastern	0	0	0	0	0	0
7912	Pig	Eastern	0	0	0	0	0	0
7913	Pig	Eastern	0	0	0	0	0	0
7914	Pig	Eastern	0	0	0	0	0	0
7915	Pig	Eastern	0	0	0	0	0	0
7916	Pig	Eastern	0	0	0	0	0	0
7917	Pig	Eastern	0	0	0	0	0	0
7918	Pig	Eastern	0	0	0	0	0	0
7919	Pig	Eastern	0	0	0	0	0	0

7920	Pig	Eastern	0	0	0	0	0	0
7921	Pig	Eastern	0	0	0	0	0	0
7922	Pig	Eastern	0	0	0	0	0	0
7923	Pig	Eastern	0	0	0	0	0	0
7924	Pig	Eastern	0	0	0	0	0	0
7925	Pig	Eastern	0	0	0	0	0	0
7926	Pig	Eastern	0	0	0	0	0	0
7927	Pig	Eastern	0	0	0	0	0	0
7928	Pig	Eastern	0	0	0	0	0	0
7929	Pig	Eastern	0	0	0	0	0	0
7930	Pig	Eastern	0	0	0	0	0	0
7931	Pig	Eastern	0	0	0	0	0	0
7932	Pig	Eastern	0	0	0	0	0	0
7933	Pig	Eastern	0	0	0	0	0	0
7934	Pig	Eastern	0	0	0	0	0	0
7935	Pig	Eastern	0	0	0	0	0	0
7936	Pig	Eastern	0	0	0	0	0	0
7937	Pig	Eastern	0	0	0	0	0	0
7938	Pig	Eastern	0	0	0	0	0	0
7939	Pig	Eastern	0	0	0	0	0	0
7940	Pig	Eastern	0	0	0	0	0	0
7941	Pig	Eastern	0	0	0	0	0	0
7942	Pig	Eastern	0	0	0	0	0	0
7943	Pig	Eastern	0	0	0	0	0	0
7944	Pig	Eastern	0	0	0	0	0	0
7945	Pig	Eastern	0	0	0	0	0	0
7946	Pig	Eastern	0	0	0	0	0	0
7947	Pig	Eastern	0	0	0	0	0	0
7948	Pig	Eastern	0	0	0	0	0	0
7949	Pig	Eastern	0	0	0	0	0	0
7950	Pig	Eastern	0	0	0	0	0	0
7951	Pig	Eastern	0	0	0	0	0	0
7952	Pig	Eastern	0	0	0	0	0	0
7953	Pig	Eastern	0	0	0	0	0	0
7954	Pig	Eastern	0	0	0	0	0	0
7955	Pig	Eastern	0	0	0	0	0	0
7956	Pig	Eastern	0	0	0	0	0	0
7957	Pig	Eastern	0	0	0	0	0	0
7958	Pig	Eastern	0	0	0	0	0	0
7959	Pig	Eastern	0	0	0	0	0	0
7960	Pig	Eastern	0	0	0	0	0	0
7961	Pig	Eastern	0	0	0	0	0	0
7962	Pig	Eastern	0	0	0	0	0	0
7963	Pig	Eastern	0	0	0	0	0	0
7964	Pig	Eastern	0	0	0	0	0	0
7965	Pig	Eastern	0	0	0	0	0	0
7966	Pig	Eastern	0	0	0	0	0	0
7967	Pig	Eastern	0	0	0	0	0	0

7968	Pig	Eastern	0	0	0	0	0	0
7969	Pig	Eastern	0	0	0	0	0	0
7970	Pig	Eastern	0	0	0	0	0	0
7971	Pig	Eastern	0	0	0	0	0	0
7972	Pig	Eastern	0	0	0	0	0	0
7973	Pig	Eastern	0	0	0	0	0	0
7974	Pig	Eastern	0	0	0	0	0	0
7975	Pig	Eastern	0	0	0	0	0	0
7976	Pig	Eastern	0	0	0	0	0	0
7977	Pig	Eastern	0	0	0	0	0	0
7978	Pig	Eastern	0	0	0	0	0	0
7979	Pig	Eastern	0	0	0	0	0	0
7980	Pig	Eastern	0	0	0	0	0	0
7981	Pig	Eastern	0	0	0	0	0	0
7982	Pig	Eastern	0	0	0	0	0	0
7983	Pig	Eastern	0	0	0	0	0	0
7984	Pig	Eastern	0	0	0	0	0	0
7985	Pig	Eastern	0	0	0	0	0	0
7986	Pig	Eastern	0	0	0	0	0	0
7987	Pig	Eastern	0	0	0	0	0	0
7988	Pig	Eastern	0	0	0	0	0	0
7989	Pig	Eastern	0	0	0	0	0	0
7990	Pig	Eastern	0	0	0	0	0	0
7991	Pig	Eastern	0	0	0	0	0	0
7992	Pig	Eastern	0	0	0	0	0	0
7993	Pig	Eastern	0	0	0	0	0	0
7994	Pig	Eastern	0	0	0	0	0	0
7995	Pig	Eastern	0	0	0	0	0	0
7996	Pig	Eastern	0	0	0	0	0	0
7997	Pig	Eastern	0	0	0	0	0	0
7998	Pig	Eastern	0	0	0	0	0	0
7999	Pig	Eastern	0	0	0	0	0	0
8000	Pig	Eastern	0	0	0	0	0	0
8001	Pig	Eastern	0	0	0	0	0	0
8002	Pig	Eastern	0	0	0	0	0	0
8003	Pig	Eastern	0	0	0	0	0	0
8004	Pig	Eastern	0	0	0	0	0	0
8005	Pig	Southern	1	0	0	0	0	1
8006	Pig	Southern	0	0	0	0	0	0
8007	Pig	Southern	0	0	0	0	0	0
8008	Pig	Southern	0	0	0	0	0	0
8009	Pig	Southern	0	0	0	0	0	0
8010	Pig	Southern	0	0	0	0	0	0
8011	Pig	Southern	0	0	0	0	0	0
8012	Pig	Southern	0	0	0	0	0	0
8013	Pig	Southern	0	0	0	0	0	0
8014	Pig	Southern	0	0	1	1	0	1
8015	Pig	Southern	0	0	1	0	0	1

8016	Pig	Southern	0	0	0	0	0	0
8017	Pig	Eastern	0	0	0	0	0	0
8018	Pig	Eastern	0	0	0	0	0	0
8019	Pig	Eastern	0	0	0	0	0	0
8020	Pig	Eastern	0	0	0	0	0	0
8021	Pig	Eastern	0	0	0	0	0	0
8022	Pig	Eastern	0	0	0	0	0	0
8023	Pig	Eastern	0	0	0	0	0	0
8024	Pig	Eastern	0	0	0	0	0	0
8025	Pig	Eastern	0	0	0	0	0	0
8026	Pig	Eastern	0	0	0	0	0	0
8027	Pig	Eastern	0	0	0	0	0	0
8028	Pig	Eastern	0	0	0	0	0	0
8029	Pig	Eastern	0	0	0	0	0	0
8030	Pig	Eastern	0	0	0	0	0	0
8031	Pig	Eastern	0	0	0	0	0	0
8032	Pig	Eastern	0	0	0	0	0	0
8033	Pig	Eastern	0	0	0	0	0	0
8034	Pig	Eastern	0	0	0	0	0	0
8035	Pig	Eastern	0	0	0	0	0	0
8036	Pig	Eastern	0	0	0	0	0	0
8037	Pig	Eastern	0	0	0	0	0	0
8038	Pig	Eastern	0	0	0	0	0	0
8039	Pig	Eastern	0	0	0	0	0	0
8040	Pig	Eastern	0	0	0	0	0	0
8041	Pig	Eastern	0	0	0	0	0	0
8042	Pig	Eastern	0	0	0	0	0	0
8043	Pig	Eastern	0	0	0	0	0	0
8044	Pig	Eastern	0	0	0	0	0	0
8045	Pig	Eastern	0	0	0	0	0	0
8046	Pig	Eastern	0	0	0	0	0	0
8047	Pig	Central	0	0	0	0	0	0
8048	Pig	Central	0	0	0	0	0	0
8049	Pig	Central	0	0	0	0	0	0
8050	Pig	Central	0	0	0	0	0	0
8051	Pig	Central	0	0	0	0	0	0
8052	Pig	Central	0	0	0	0	0	0
8053	Pig	Central	0	0	0	0	0	0
8054	Pig	Central	0	0	0	0	0	0
8055	Pig	Central	0	0	0	0	0	0
8056	Pig	Central	0	0	0	0	0	0
8057	Pig	Central	0	0	0	0	0	0
8058	Pig	Central	0	0	0	0	0	0
8059	Pig	Central	0	0	0	0	0	0
8060	Pig	Central	0	0	0	0	0	0
8061	Pig	Central	0	0	0	0	0	0
8062	Pig	Central	0	0	0	0	0	0
8063	Pig	Central	0	0	0	0	0	0

8064	Pig	Central	0	0	0	0	0	0
8065	Pig	Central	0	0	0	0	0	0
8066	Pig	Central	0	0	0	0	0	0
8067	Pig	Central	0	0	0	0	0	0
8068	Pig	Central	0	0	0	0	0	0
8069	Pig	Central	0	0	0	0	0	0
8070	Pig	Central	0	0	0	0	0	0
8071	Pig	Central	0	0	0	0	0	0
8072	Pig	Central	0	0	0	0	0	0
8073	Pig	Central	0	0	0	0	0	0
8074	Pig	Central	0	0	0	0	0	0
8075	Pig	Central	0	0	0	0	0	0
8076	Pig	Central	0	0	0	0	0	0
8077	Pig	Central	0	0	0	0	0	0
8078	Pig	Central	0	0	0	0	0	0
8079	Pig	Central	0	0	0	0	0	0
8080	Pig	Central	0	0	0	0	0	0
8081	Pig	Central	0	0	0	0	0	0
8082	Pig	Central	0	0	0	0	0	0
8083	Pig	Central	0	0	0	0	0	0
8084	Pig	Central	0	0	0	0	0	0
8085	Pig	Central	0	0	0	0	0	0
8086	Pig	Central	0	0	0	0	0	0
8087	Pig	Central	0	0	0	0	0	0
8088	Pig	Central	0	0	0	0	0	0
8089	Pig	Central	0	0	0	0	0	0
8090	Pig	Central	0	0	0	0	0	0
8091	Pig	Central	0	0	0	0	0	0
8092	Pig	Central	0	0	0	0	0	0
8093	Pig	Central	0	0	0	0	0	0
8094	Pig	Central	0	0	0	0	0	0
8095	Pig	Central	0	0	0	0	0	0
8096	Pig	Central	0	0	0	0	0	0
8097	Pig	Central	0	0	0	0	0	0
8098	Pig	Central	0	0	0	0	0	0
8099	Pig	Central	0	0	0	0	0	0
8100	Pig	Central	0	0	0	0	0	0
8101	Pig	Central	0	0	0	0	0	0
8102	Pig	Central	0	0	0	0	0	0
8103	Pig	Central	0	0	0	0	0	0
8104	Pig	Central	0	0	0	0	0	0
8105	Pig	Central	0	0	0	0	0	0
8106	Pig	Central	0	0	0	0	0	0
8107	Pig	Central	0	0	0	0	0	0
8108	Pig	Central	0	0	0	0	0	0
8109	Pig	Central	0	0	0	0	0	0
8110	Pig	Central	0	0	0	0	0	0
8111	Pig	Central	0	0	0	0	0	0

8112	Pig	Central	0	0	0	0	0	0
8113	Pig	Central	0	0	0	0	0	0
8114	Pig	Central	0	0	0	0	0	0
8115	Pig	Central	0	0	0	0	0	0
8116	Pig	Central	0	0	0	0	0	0
8117	Pig	Central	0	0	0	0	0	0
8118	Pig	Eastern	0	0	0	0	0	0
8119	Pig	Eastern	0	0	0	0	0	0
8120	Pig	Eastern	0	0	0	0	0	0
8121	Pig	Eastern	0	0	0	0	0	0
8122	Pig	Eastern	0	0	0	0	0	0
8123	Pig	Eastern	0	0	0	0	0	0
8124	Pig	Eastern	0	0	0	0	0	0
8125	Pig	Eastern	0	0	0	0	0	0
8126	Pig	Central	0	0	0	0	0	0
8127	Pig	Central	0	0	0	0	0	0
8128	Pig	Central	0	0	1	1	0	1
8129	Pig	Central	0	0	0	0	0	0
8130	Pig	Central	0	0	0	0	0	0
8131	Pig	Central	0	0	1	1	0	1
8132	Pig	Central	0	0	0	0	0	0
8133	Pig	Central	0	0	0	0	0	0
8134	Pig	Central	0	0	0	0	0	0
8135	Pig	Central	0	0	0	0	0	0
8136	Pig	Central	0	0	0	0	0	0
8137	Pig	Central	0	0	0	0	0	0
8138	Pig	Central	0	0	0	0	0	0
8139	Pig	Central	0	0	1	0	0	1
8140	Pig	Central	0	0	0	0	0	0
8141	Pig	Central	0	0	0	0	0	0
8142	Pig	Central	0	0	0	0	0	0
8143	Pig	Central	0	0	0	0	0	0
8144	Pig	Central	0	0	0	0	0	1
8145	Pig	Central	0	0	0	0	0	0
8146	Pig	Central	0	0	0	0	0	0
8147	Pig	Central	0	0	0	0	0	0
8148	Pig	Central	1	0	0	0	0	1
8149	Pig	Central	0	0	0	0	0	0
8150	Pig	Central	0	0	0	1	0	1
8151	Pig	Central	0	0	0	0	0	0
8152	Pig	Central	0	0	0	0	0	0
8153	Pig	Central	0	0	0	0	0	0
8154	Pig	Central	0	0	0	0	0	0
8155	Pig	Central	0	0	0	0	0	0
8156	Pig	Central	0	0	0	0	0	0
8157	Pig	Central	0	0	0	0	0	0
8158	Pig	Central	0	0	0	0	0	0
8159	Pig	Central	0	0	0	0	0	0

8160	Pig	Central	0	0	0	0	0	0
8161	Pig	Central	0	0	0	0	0	0
8162	Pig	Central	0	0	0	0	0	0
8163	Pig	Central	0	0	0	0	0	0
8164	Pig	Central	0	0	0	0	0	0
8165	Pig	Central	0	0	0	0	0	0
8166	Pig	Central	0	0	0	0	0	0
8167	Pig	Central	0	0	0	0	0	0
8168	Pig	Central	0	0	0	0	0	0
8169	Pig	Central	0	0	0	0	0	0
8170	Pig	Central	0	0	0	0	0	0
8171	Pig	Central	0	0	0	0	0	0
8172	Pig	Central	0	0	0	0	0	0
8173	Pig	Central	0	0	0	0	0	0
8174	Pig	Central	0	0	0	0	0	0
8175	Pig	Central	0	0	0	0	0	0
8176	Pig	Central	0	0	0	0	0	0
8177	Pig	Central	0	0	0	0	0	0
8178	Pig	Central	0	0	0	0	0	0
8179	Pig	Central	0	0	0	0	0	0
8180	Pig	Central	0	0	0	0	0	0
8181	Pig	Central	0	0	0	0	0	0
8182	Pig	Central	0	0	0	0	0	0
8183	Pig	Central	0	0	0	0	0	0
8184	Pig	Central	0	0	0	0	0	0
8185	Pig	Central	0	0	0	0	0	0
8186	Pig	Central	0	0	0	0	0	0
8187	Pig	Central	0	0	0	0	0	0
8188	Pig	Central	0	0	0	0	0	0
8189	Pig	Central	0	0	0	0	0	0
8190	Pig	Central	0	0	0	0	0	0
8191	Pig	Central	0	0	0	0	0	0
8192	Pig	Central	0	0	0	0	0	0
8193	Pig	Central	0	0	0	0	0	0
8194	Pig	Central	0	0	0	0	0	0
8195	Pig	Central	0	0	0	0	0	0
8196	Pig	Central	0	0	0	0	0	0
8197	Pig	Central	0	0	0	0	0	0
8198	Pig	Central	0	0	1	0	0	1
8199	Pig	Central	0	0	0	0	0	0
8200	Pig	Central	0	0	1	0	0	1
8201	Pig	Central	0	0	0	0	0	0
8202	Pig	Central	0	0	0	0	0	0
8203	Pig	Central	0	0	0	0	0	0
8204	Pig	Central	0	0	0	0	0	0
8205	Pig	Central	0	0	0	0	0	0
8206	Pig	Central	0	0	0	0	0	0
8207	Pig	Central	0	0	0	0	0	0

8208	Pig	Central	0	0	0	0	0	0
8209	Pig	Central	0	0	0	0	0	0
8210	Pig	Central	0	0	0	0	0	0
8211	Pig	Central	0	0	0	0	0	0
8212	Pig	Central	0	0	0	0	0	0
8213	Pig	Central	0	0	0	0	0	0
8214	Pig	Central	0	0	0	0	0	0
8215	Pig	Central	0	0	0	0	0	0
8216	Pig	Central	0	0	0	0	0	0
8217	Pig	Central	0	0	0	0	0	0
8218	Pig	Central	0	0	0	0	0	0
8219	Pig	Central	0	0	0	0	0	0
8220	Pig	Central	0	0	0	0	0	0
8221	Pig	Central	0	0	0	0	0	0
8222	Pig	Central	0	0	0	0	0	0
8223	Pig	Central	0	0	0	0	0	0
8224	Pig	Central	0	0	0	0	0	0
8225	Pig	Central	0	0	0	0	0	0
8226	Pig	Central	0	0	0	0	0	0
8227	Pig	Central	0	0	0	0	0	0
8228	Pig	Central	0	0	0	0	0	0
8229	Pig	Central	0	0	0	0	0	0
8230	Pig	Central	0	0	0	0	0	0
8231	Pig	Central	0	0	0	0	0	0
8232	Pig	Central	0	0	0	0	0	0
8233	Pig	Central	0	0	0	0	0	0
8234	Pig	Central	0	0	0	0	0	0
8235	Pig	Central	0	0	0	0	0	0
8236	Pig	Central	0	0	0	0	0	0
8237	Pig	Central	0	0	0	0	0	0
8238	Pig	Central	0	0	0	0	0	0
8239	Pig	Central	0	0	0	0	0	0
8240	Pig	Central	0	0	0	0	0	0
8241	Pig	Central	0	0	0	0	0	0
8242	Pig	Central	0	0	0	0	0	0
8243	Pig	Central	0	0	0	0	0	0
8244	Pig	Central	0	0	0	0	0	0
8245	Pig	Central	0	0	0	0	0	0
8246	Pig	Central	1	0	0	0	0	1
8247	Pig	Central	0	0	1	0	0	1
8248	Pig	Central	0	0	0	0	0	0
8249	Pig	Central	0	0	1	1	0	1
8250	Pig	Central	0	0	0	0	0	0
8251	Pig	Central	0	0	0	0	0	0
8252	Pig	Central	0	0	1	1	0	1
8253	Pig	Central	0	0	0	0	0	0
8254	Pig	Central	0	0	0	0	0	0
8255	Pig	Central	0	0	0	0	0	0

8256	Pig	Central	0	0	0	0	0	0
8257	Pig	Central	0	0	0	0	0	0
8258	Pig	Central	0	0	0	0	0	0
8259	Pig	Central	0	0	0	0	0	0
8260	Pig	Central	0	0	0	0	0	0
8261	Pig	Central	0	0	0	0	0	0
8262	Pig	Central	0	0	0	0	0	0
8263	Pig	Central	0	0	0	0	0	0
8264	Pig	Central	0	0	0	0	0	0
8265	Pig	Central	0	0	0	0	0	0
8266	Pig	Central	0	0	0	0	0	0
8267	Pig	Central	0	0	0	0	0	0
8268	Pig	Central	0	0	0	0	0	0
8269	Pig	Central	0	0	0	0	0	0
8270	Pig	Central	0	0	0	0	0	0
8271	Pig	Central	0	0	0	0	0	0
8272	Pig	Central	0	0	0	0	0	0
8273	Pig	Central	0	0	0	0	0	0
8274	Pig	Central	0	0	0	0	0	0
8275	Pig	Central	0	0	0	0	0	0
8276	Pig	Central	0	0	0	0	0	0
8277	Pig	Central	0	0	0	0	0	0
8278	Pig	Central	0	0	0	0	0	0
8279	Pig	Central	0	0	0	0	0	0
8280	Pig	Central	0	0	0	0	0	0
8281	Pig	Central	0	0	0	0	0	0
8282	Pig	Central	0	0	0	0	0	0
8283	Pig	Central	0	0	0	0	0	0
8284	Pig	Central	0	0	0	0	0	0
8285	Pig	Central	0	0	0	0	0	0
8286	Pig	Central	0	0	0	0	0	0
8287	Pig	Central	0	0	0	0	0	0
8288	Pig	Central	0	0	0	0	0	0
8289	Pig	Central	0	0	0	0	0	0
8290	Pig	Central	0	0	0	0	0	0
8291	Pig	Central	0	0	0	0	0	0
8292	Pig	Central	0	0	0	0	0	0
8293	Pig	Central	0	0	0	0	0	0
8294	Pig	Central	0	0	0	0	0	0
8295	Pig	Central	0	0	0	0	0	0
8296	Pig	Central	0	0	0	0	0	0
8297	Pig	Central	0	0	0	0	0	0
8298	Pig	Central	0	0	0	0	0	0
8299	Pig	Central	0	0	0	0	0	0
8300	Pig	Central	0	0	0	0	0	0
8301	Pig	Central	0	0	0	0	0	0
8302	Pig	Central	0	0	0	0	0	0
8303	Pig	Central	0	0	0	0	0	0

8304	Pig	Central	0	0	0	0	0	0
8305	Pig	Central	0	0	0	0	0	0
8306	Pig	Central	0	0	0	0	0	0
8307	Pig	Central	0	0	0	0	0	0
8308	Pig	Central	0	0	0	0	0	0
8309	Pig	Central	0	0	0	0	0	0
8310	Pig	Central	0	0	0	0	0	0
8311	Pig	Central	0	0	0	0	0	0
8312	Pig	Central	0	0	0	0	0	0
8313	Pig	Central	0	0	0	0	0	0
8314	Pig	Central	0	0	0	0	0	0
8315	Pig	Central	0	0	0	0	0	0
8316	Pig	Central	0	0	0	0	0	0
8317	Pig	Central	0	0	0	0	0	0
8318	Pig	Central	0	0	0	0	0	0
8319	Pig	Central	0	0	0	0	0	0
8320	Pig	Central	0	0	0	0	0	0
8321	Pig	Central	0	0	0	0	0	0
8322	Pig	Central	0	0	0	0	0	0
8323	Pig	Central	0	0	0	0	0	0
8324	Pig	Central	0	0	0	0	0	0
8325	Pig	Central	0	0	0	0	0	0
8326	Pig	Central	0	0	0	0	0	0
8327	Pig	Central	0	0	0	0	0	0
8328	Pig	Central	0	0	0	0	0	0
8329	Pig	Central	0	0	1	0	0	1
8330	Pig	Central	0	0	0	0	0	0
8331	Pig	Central	0	0	0	0	0	0
8332	Pig	Central	0	0	0	0	0	0
8333	Pig	Central	0	0	0	0	0	0
8334	Pig	Central	0	0	0	0	0	0
8335	Pig	Central	0	0	0	0	0	0
8336	Pig	Central	0	0	0	0	0	0
8337	Pig	Central	0	0	0	0	0	0
8338	Pig	Central	0	0	0	0	0	0
8339	Pig	Central	0	0	0	0	0	0
8340	Pig	Central	0	0	0	0	0	0
8341	Pig	Central	0	0	0	0	0	0
8342	Pig	Central	0	0	0	0	0	0
8343	Pig	Central	0	0	0	0	0	0
8344	Pig	Central	0	0	1	0	0	1
8345	Pig	Central	0	0	1	0	0	1
8346	Pig	Central	0	0	0	0	0	0
8347	Pig	Central	0	0	0	0	0	0
8348	Pig	Central	0	0	0	0	0	0
8349	Pig	Central	0	0	0	0	0	0
8350	Pig	Central	0	0	0	0	0	0
8351	Pig	Central	0	0	0	0	0	0

8352	Pig	Central	0	0	0	0	0	0
8353	Pig	Central	0	0	0	0	0	0
8354	Pig	Central	0	0	0	0	0	0
8355	Pig	Central	0	0	0	0	0	0
8356	Pig	Central	0	0	0	0	0	0
8357	Pig	Central	0	0	0	0	0	0
8358	Pig	Central	0	0	1	0	0	1
8359	Pig	Central	0	0	0	0	0	0
8360	Pig	Central	0	0	0	0	0	0
8361	Pig	Central	0	0	0	0	0	0
8362	Pig	Central	0	0	0	0	0	0
8363	Pig	Central	0	0	0	0	0	0
8364	Pig	Central	0	0	0	0	0	0
8365	Pig	Central	0	0	0	0	0	0
8366	Pig	Central	0	0	0	0	0	0
8367	Pig	Central	0	0	0	0	0	0
8368	Pig	Central	0	0	0	0	0	0
8369	Pig	Central	0	0	0	0	0	0
8370	Pig	Central	0	0	1	0	0	1
8371	Pig	Central	0	0	0	0	0	0
8372	Pig	Central	0	0	0	0	0	0
8373	Pig	Central	0	0	0	0	0	0
8374	Pig	Central	0	0	0	0	0	0
8375	Pig	Central	0	0	0	0	0	0
8376	Pig	Central	0	0	0	0	0	0
8377	Pig	Central	0	0	0	0	0	0
8378	Pig	Central	0	0	0	0	0	0
8379	Pig	Central	0	0	0	0	0	0
8380	Pig	Central	0	0	0	0	0	0
8381	Pig	Central	0	0	0	0	0	0
8382	Pig	Central	0	0	0	0	0	0
8383	Pig	Central	0	0	0	0	0	0
8384	Pig	Central	0	0	0	0	0	0
8385	Pig	Central	0	0	0	0	0	0
8386	Pig	Central	0	0	0	0	0	0
8387	Pig	Central	0	0	0	0	0	0
8388	Pig	Central	0	0	0	0	0	0
8389	Pig	Central	0	0	0	0	0	0
8390	Pig	Central	0	0	0	0	0	0
8391	Pig	Central	0	0	0	0	0	0
8392	Pig	Central	0	0	0	0	0	0
8393	Pig	Central	0	0	0	0	0	0
8394	Pig	Central	0	0	0	0	0	0
8395	Pig	Central	0	0	0	0	0	0
8396	Pig	Central	0	0	0	0	0	0
8397	Pig	Northeastern	0	0	0	0	0	0
8398	Pig	Northeastern	0	0	0	0	0	0
8399	Pig	Northeastern	0	0	0	0	0	0

8400	Pig	Northeastern	0	0	0	0	0	0
8401	Pig	Northeastern	0	0	0	0	0	0
8402	Pig	Northeastern	0	0	0	0	0	0
8403	Pig	Northeastern	0	0	0	0	0	0
8404	Pig	Northeastern	0	0	0	0	0	0
8405	Pig	Northeastern	0	0	0	0	0	0
8406	Pig	Northeastern	0	0	0	0	0	0
8407	Pig	Northeastern	0	0	0	0	0	0
8408	Pig	Northeastern	0	0	0	0	0	0
8409	Pig	Northeastern	0	0	0	0	0	0
8410	Pig	Northeastern	0	0	0	0	0	0
8411	Pig	Northeastern	0	0	0	0	0	0
8412	Pig	Northeastern	0	0	0	0	0	0
8413	Pig	Northeastern	0	0	0	0	0	0
8414	Pig	Northeastern	0	0	0	0	0	0
8415	Pig	Northeastern	0	0	0	0	0	0
8416	Pig	Northeastern	0	0	0	0	0	0
8417	Pig	Northeastern	0	0	0	0	0	0
8418	Pig	Northeastern	0	0	0	0	0	0
8419	Pig	Northeastern	0	0	0	0	0	0
8420	Pig	Northeastern	0	0	0	0	0	0
8421	Pig	Northeastern	0	0	0	0	0	0
8422	Pig	Northeastern	0	0	0	0	0	0
8423	Pig	Northeastern	0	0	0	0	0	0
8424	Pig	Northeastern	0	0	0	0	0	0
8425	Pig	Northeastern	0	0	0	0	0	0
8426	Pig	Northeastern	0	0	0	0	0	0
8427	Pig	Northeastern	0	0	0	0	0	0
8428	Pig	Northeastern	0	0	0	0	0	0
8429	Pig	Northeastern	0	0	0	0	0	0
8430	Pig	Northeastern	0	0	0	0	0	0
8431	Pig	Northeastern	0	0	0	0	0	0
8432	Pig	Northeastern	0	0	0	0	0	0
8433	Pig	Northeastern	0	0	0	0	0	0
8434	Pig	Northeastern	0	0	0	0	0	0
8435	Pig	Northeastern	0	0	0	0	0	0
8436	Pig	Northeastern	0	0	0	0	0	0
8437	Pig	Northeastern	0	0	0	0	0	0
8438	Pig	Northeastern	0	0	0	0	0	0
8439	Pig	Northeastern	0	0	0	0	0	0
8440	Pig	Northeastern	0	0	0	0	0	0
8441	Pig	Northeastern	0	0	0	0	0	0
8442	Pig	Northeastern	0	0	0	0	0	0
8443	Pig	Northeastern	0	0	0	0	0	0
8444	Pig	Northeastern	0	0	0	0	0	0
8445	Pig	Northeastern	0	0	0	0	0	0
8446	Pig	Northeastern	0	0	0	0	0	0
8447	Pig	Northeastern	0	0	0	0	0	0

8448	Pig	Northeastern	0	0	0	0	0	0
8449	Pig	Northeastern	0	0	0	0	0	0
8450	Pig	Northeastern	0	0	0	0	0	0
8451	Pig	Northeastern	0	0	0	0	0	0
8452	Pig	Northeastern	0	0	0	0	0	0
8453	Pig	Northeastern	0	0	0	0	0	0
8454	Pig	Northeastern	0	0	0	0	0	0
8455	Pig	Northeastern	0	0	0	0	0	0
8456	Pig	Northeastern	0	0	0	0	0	0
8457	Pig	Northeastern	0	0	0	0	0	0
8458	Pig	Northeastern	0	0	0	0	0	0
8459	Pig	Northeastern	0	0	0	0	0	0
8460	Pig	Northeastern	0	0	0	0	0	0
8461	Pig	Northeastern	0	0	0	0	0	0
8462	Pig	Northeastern	0	0	0	0	0	0
8463	Pig	Northeastern	0	0	0	0	0	0
8464	Pig	Northeastern	0	0	0	0	0	0
8465	Pig	Northeastern	0	0	0	0	0	0
8466	Pig	Northeastern	0	0	0	0	0	0
8467	Pig	Northeastern	0	0	0	0	0	0
8468	Pig	Northeastern	0	0	1	1	0	1
8469	Pig	Northeastern	0	0	0	0	0	0
8470	Pig	Northeastern	0	0	0	0	0	0
8471	Pig	Northeastern	0	0	1	1	0	1
8472	Pig	Northeastern	0	0	0	0	0	0
8473	Pig	Northeastern	0	0	1	1	0	1
8474	Pig	Northeastern	0	0	0	0	0	0
8475	Pig	Northeastern	0	0	0	0	0	0
8476	Pig	Northeastern	0	0	0	0	0	0
8477	Pig	Northeastern	0	0	0	0	0	0
8478	Pig	Northeastern	0	0	1	0	0	1
8479	Pig	Northeastern	0	0	1	1	0	1
8480	Pig	Northeastern	0	0	0	0	0	0
8481	Pig	Northeastern	0	0	0	0	0	0
8482	Pig	Northeastern	0	0	0	0	0	0
8483	Pig	Northeastern	0	0	0	0	0	0
8484	Pig	Northeastern	0	0	0	0	0	0
8485	Pig	Northeastern	0	0	0	0	0	0
8486	Pig	Northeastern	0	0	0	0	0	0
8487	Pig	Northeastern	0	0	0	0	0	0
8488	Pig	Northeastern	0	0	0	0	0	0
8489	Pig	Northeastern	0	0	0	0	0	0
8490	Pig	Northeastern	0	0	0	0	0	0
8491	Pig	Northeastern	0	0	0	0	0	0
8492	Pig	Northeastern	0	0	0	0	0	0
8493	Pig	Northeastern	0	0	0	0	0	0
8494	Pig	Northeastern	0	0	0	0	0	0
8495	Pig	Northeastern	0	0	0	0	0	0

8496	Pig	Northeastern	0	0	0	0	0	0
8497	Pig	Northeastern	0	0	0	0	0	0
8498	Pig	Northeastern	0	0	0	0	0	0
8499	Pig	Northeastern	0	0	0	0	0	0
8500	Pig	Northeastern	0	0	0	0	0	0
8501	Pig	Northeastern	0	0	0	0	0	0
8502	Pig	Northeastern	0	0	0	0	0	0
8503	Pig	Northeastern	0	0	0	0	0	0
8504	Pig	Northeastern	0	0	0	0	0	0
8505	Pig	Northeastern	0	0	0	0	0	0
8506	Pig	Northeastern	0	0	0	0	0	0
8507	Pig	Northeastern	0	0	0	0	0	0
8508	Pig	Northeastern	0	0	0	0	0	0
8509	Pig	Northeastern	0	0	0	0	0	0
8510	Pig	Northeastern	0	0	0	0	0	0
8511	Pig	Northeastern	0	0	0	0	0	0
8512	Pig	Northeastern	0	0	0	0	0	0
8513	Pig	Northeastern	0	0	0	0	0	0
8514	Pig	Northeastern	0	0	0	0	0	0
8515	Pig	Northeastern	0	0	0	0	0	0
8516	Pig	Northeastern	0	0	0	0	0	0
8517	Pig	Northeastern	0	0	0	0	0	0
8518	Pig	Northeastern	0	0	0	0	0	0
8519	Pig	Northeastern	0	0	0	0	0	0
8520	Pig	Northeastern	0	0	0	0	0	0
8521	Pig	Northeastern	0	0	0	0	0	0
8522	Pig	Northeastern	0	0	0	0	0	0
8523	Pig	Northeastern	0	0	0	0	0	0
8524	Pig	Northeastern	0	0	0	0	0	0
8525	Pig	Northeastern	0	0	0	0	0	0
8526	Pig	Northeastern	0	0	0	0	0	0
8527	Pig	Northeastern	0	0	0	0	0	0
8528	Pig	Northeastern	0	0	0	0	0	0
8529	Pig	Northeastern	0	0	0	0	0	0
8530	Pig	Central	0	0	0	0	0	0
8531	Pig	Central	0	0	0	0	0	0
8532	Pig	Central	0	0	0	0	0	0
8533	Pig	Central	0	0	0	0	0	0
8534	Pig	Central	0	0	0	0	0	0
8535	Pig	Central	0	0	0	0	0	0
8536	Pig	Central	0	0	0	0	0	0
8537	Pig	Central	0	0	0	0	0	0
8538	Pig	Central	0	0	0	0	0	0
8539	Pig	Central	0	0	1	0	0	1
8540	Pig	Central	0	0	0	0	0	0
8541	Pig	Central	0	0	0	0	0	0
8542	Pig	Central	0	0	0	0	0	0
8543	Pig	Central	0	0	0	0	0	0

8544	Pig	Central	0	0	0	0	0	0
8545	Pig	Central	0	0	0	0	0	0
8546	Pig	Central	0	0	0	0	0	0
8547	Pig	Central	0	0	0	0	0	0
8548	Pig	Central	0	0	0	0	0	0
8549	Pig	Central	0	0	0	0	0	0
8550	Pig	Central	0	0	0	0	0	0
8551	Pig	Central	0	0	0	0	0	0
8552	Pig	Central	0	0	0	0	0	0
8553	Pig	Central	0	0	0	0	0	0
8554	Pig	Central	0	0	0	0	0	0
8555	Pig	Central	0	0	0	0	0	0
8556	Pig	Central	0	0	0	0	0	0
8557	Pig	Central	0	0	0	0	0	0
8558	Pig	Central	0	0	0	0	0	0
8559	Pig	Central	1	0	0	0	0	1
8560	Pig	Central	0	0	0	0	0	0
8561	Pig	Central	0	0	1	0	0	1
8562	Pig	Central	0	0	0	0	0	0
8563	Pig	Central	0	0	1	0	0	1
8564	Pig	Central	0	0	0	0	0	0
8565	Pig	Central	0	0	0	0	0	0
8566	Pig	Central	0	0	1	0	0	1
8567	Pig	Central	0	0	0	0	0	0
8568	Pig	Central	0	0	0	0	0	0
8569	Pig	Central	0	0	0	0	0	0
8570	Pig	Central	0	0	0	0	0	0
8571	Pig	Central	0	0	0	0	0	0
8572	Pig	Central	0	0	0	0	0	0
8573	Pig	Central	0	0	0	0	0	0
8574	Pig	Central	0	0	0	0	0	0
8575	Pig	Central	0	0	1	0	0	1
8576	Pig	Central	0	0	0	0	0	0
8577	Pig	Central	0	0	1	0	0	1
8578	Pig	Central	0	0	1	0	0	1
8579	Pig	Central	0	0	0	0	0	0
8580	Pig	Central	0	0	0	0	0	0
8581	Pig	Central	0	0	0	0	0	0
8582	Pig	Central	0	0	0	0	0	0
8583	Pig	Central	0	0	1	0	0	1
8584	Pig	Central	0	0	0	0	0	0
8585	Pig	Central	0	0	1	1	0	1
8586	Pig	Central	0	0	0	0	0	0
8587	Pig	Central	0	0	0	0	0	0
8588	Pig	Central	0	0	1	0	0	1
8589	Pig	Central	0	0	0	0	0	0
8590	Pig	Central	0	0	0	0	0	0
8591	Pig	Central	0	0	0	0	0	0

8592	Pig	Central	0	0	0	0	0	0
8593	Pig	Central	0	0	0	0	0	0
8594	Pig	Central	0	0	0	0	0	0
8595	Pig	Central	0	0	0	0	0	0
8596	Pig	Central	0	0	0	0	0	0
8597	Pig	Central	0	0	0	0	0	0
8598	Pig	Central	0	0	0	0	0	0
8599	Pig	Central	0	0	0	0	0	0
8600	Pig	Central	0	0	0	0	0	0
8601	Pig	Central	0	0	0	0	0	0
8602	Pig	Central	0	0	0	0	0	0
8603	Pig	Central	0	0	0	0	0	0
8604	Pig	Central	0	0	0	0	0	0
8605	Pig	Central	0	0	0	0	0	0
8606	Pig	Central	0	0	0	0	0	0
8607	Pig	Central	0	0	0	0	0	0
8608	Pig	Central	0	0	0	0	0	0
8609	Pig	Central	0	0	0	0	0	0
8610	Pig	Southern	0	0	0	0	0	0
8611	Pig	Central	1	0	0	0	0	1
8612	Pig	Central	0	0	0	0	0	0
8613	Pig	Central	0	0	0	0	0	0
8614	Pig	Central	0	0	0	0	0	0
8615	Pig	Central	0	0	0	0	0	0
8616	Pig	Central	0	0	0	0	0	0
8617	Pig	Central	0	0	0	0	0	0
8618	Pig	Central	0	0	0	0	0	0
8619	Pig	Central	0	0	0	0	0	0
8620	Pig	Central	0	0	0	0	0	0
8621	Pig	Central	0	0	0	0	0	0
8622	Pig	Central	0	0	0	0	0	0
8623	Pig	Central	0	0	0	0	0	0
8624	Pig	Central	0	0	0	0	0	0
8625	Pig	Central	0	0	0	0	0	0
8626	Pig	Central	0	0	0	0	0	0
8627	Pig	Central	0	0	0	0	0	0
8628	Pig	Central	0	0	0	0	0	0
8629	Pig	Central	0	0	0	0	0	0
8630	Pig	Central	0	0	0	0	0	0
8631	Pig	Central	0	0	0	0	0	0
8632	Pig	Central	0	0	0	0	0	0
8633	Pig	Central	0	0	0	0	0	0
8634	Pig	Central	0	0	0	0	0	0
8635	Pig	Central	0	0	0	0	0	0
8636	Pig	Central	0	0	0	0	0	0
8637	Pig	Central	0	0	0	0	0	0
8638	Pig	Central	0	0	0	0	0	0
8639	Pig	Central	0	0	0	0	0	0

8640	Pig	Central	0	0	0	0	0	0
8641	Pig	Central	0	0	0	0	0	0
8642	Pig	Central	0	0	0	0	0	0
8643	Pig	Central	0	0	0	0	0	0
8644	Pig	Central	0	0	0	0	0	0
8645	Pig	Central	0	0	0	0	0	0
8646	Pig	Central	0	0	0	0	0	0
8647	Pig	Central	0	0	0	0	0	0
8648	Pig	Central	0	0	0	0	0	0
8649	Pig	Central	0	0	0	0	0	0
8650	Pig	Central	0	0	0	0	0	0
8651	Pig	Central	0	0	0	0	0	0
8652	Pig	Central	0	0	0	0	0	0
8653	Pig	Central	0	0	0	0	0	0
8654	Pig	Central	0	0	0	0	0	0
8655	Pig	Central	0	0	0	0	0	0
8656	Pig	Central	0	0	0	0	0	0
8657	Pig	Central	0	0	0	0	0	0
8658	Pig	Central	0	0	0	0	0	0
8659	Pig	Central	0	0	0	0	0	0
8660	Pig	Central	0	0	0	0	0	0
8661	Pig	Central	0	0	0	0	0	0
8662	Pig	Central	0	0	0	0	0	0
8663	Pig	Central	0	0	0	0	0	0
8664	Pig	Central	0	0	0	0	0	0
8665	Pig	Central	1	0	0	1	0	1
8666	Pig	Central	0	0	0	0	0	0
8667	Pig	Central	0	0	0	0	0	0
8668	Pig	Central	0	0	0	0	0	0
8669	Pig	Central	0	0	0	0	0	0
8670	Pig	Central	0	0	0	0	0	0
8671	Pig	Central	0	0	0	0	0	0
8672	Pig	Central	0	0	0	0	0	0
8673	Pig	Central	0	0	0	0	0	0
8674	Pig	Central	0	0	0	0	0	0
8675	Pig	Central	0	0	0	0	0	0
8676	Pig	Central	0	0	0	0	0	0
8677	Pig	Central	0	0	0	0	0	0
8678	Pig	Central	0	0	0	0	0	0
8679	Pig	Central	0	0	0	0	0	0
8680	Pig	Central	0	0	0	0	0	0
8681	Pig	Central	0	0	0	0	0	0
8682	Pig	Central	0	0	0	0	0	0
8683	Pig	Central	0	0	0	0	0	0
8684	Pig	Central	0	0	0	0	0	0
8685	Pig	Central	0	0	0	0	0	0
8686	Pig	Central	0	0	0	0	0	0
8687	Pig	Central	0	0	0	0	0	0

8688	Pig	Central	0	0	0	0	0	0
8689	Pig	Central	0	0	0	0	0	0
8690	Pig	Central	0	0	0	0	0	0
8691	Pig	Central	0	0	0	0	0	0
8692	Pig	Central	0	0	0	0	0	0
8693	Pig	Central	0	0	0	0	0	0
8694	Pig	Central	0	0	0	0	0	0
8695	Pig	Central	0	0	0	0	0	0
8696	Pig	Central	0	0	0	0	0	0
8697	Pig	Central	0	0	0	0	0	0
8698	Pig	Central	0	0	0	0	0	0
8699	Pig	Central	0	0	0	0	0	0
8700	Pig	Central	0	0	0	0	0	0
8701	Pig	Central	0	0	0	0	0	0
8702	Pig	Central	0	0	0	0	0	0
8703	Pig	Central	0	0	0	0	0	0
8704	Pig	Central	0	0	0	0	0	0
8705	Pig	Central	0	0	0	0	0	0
8706	Pig	Central	0	0	0	0	0	0
8707	Pig	Central	0	0	0	0	0	0
8708	Pig	Central	0	0	0	0	0	0
8709	Pig	Central	0	0	0	0	0	0
8710	Pig	Central	0	0	0	0	0	0
8711	Pig	Central	0	0	0	0	0	0
8712	Pig	Central	0	0	0	0	0	0
8713	Pig	Central	0	0	0	0	0	0
8714	Pig	Central	0	0	0	0	0	0
8715	Pig	Central	0	0	0	0	0	0
8716	Pig	Central	0	0	0	0	0	0
8717	Pig	Central	0	0	0	0	0	0
8718	Pig	Central	0	0	0	0	0	0
8719	Pig	Central	0	0	0	0	0	0
8720	Pig	Central	0	0	0	0	0	0
8721	Pig	Central	0	0	0	0	0	0
8722	Pig	Central	0	0	0	0	0	0
8723	Pig	Central	0	0	0	0	0	0
8724	Pig	Central	0	0	0	0	0	0
8725	Pig	Central	0	0	0	0	0	0
8726	Pig	Central	0	0	0	0	0	0
8727	Pig	Central	0	0	0	0	0	0
8728	Pig	Central	0	0	0	0	0	0
8729	Pig	Central	0	0	0	0	0	0
8730	Pig	Central	0	0	0	0	0	0
8731	Pig	Central	0	0	0	0	0	0
8732	Pig	Central	0	0	0	0	0	0
8733	Pig	Central	0	0	0	0	0	0
8734	Pig	Central	0	0	0	0	0	0
8735	Pig	Central	0	0	0	0	0	0

8736	Pig	Central	0	0	0	0	0	0
8737	Pig	Central	0	0	0	0	0	0
8738	Pig	Central	0	0	0	0	0	0
8739	Pig	Central	0	0	0	0	0	0
8740	Pig	Central	0	0	0	0	0	0
8741	Pig	Central	0	0	0	0	0	0
8742	Pig	Central	0	0	0	0	0	0
8743	Pig	Central	0	0	0	0	0	0
8744	Pig	Central	0	0	0	0	0	0
8745	Pig	Central	0	0	0	0	0	0
8746	Pig	Central	0	0	0	0	0	0
8747	Pig	Central	0	0	0	0	0	0
8748	Pig	Central	0	0	0	0	0	0
8749	Pig	Central	0	0	0	0	0	0
8750	Pig	Central	0	0	0	0	0	0
8751	Pig	Central	0	0	0	0	0	0
8752	Pig	Central	0	0	0	0	0	0
8753	Pig	Central	0	0	0	0	0	0
8754	Pig	Central	0	0	0	0	0	0
8755	Pig	Central	0	0	0	0	0	0
8756	Pig	Central	0	0	0	0	0	0
8757	Pig	Central	0	0	0	0	0	0
8758	Pig	Central	0	0	0	0	0	0
8759	Pig	Central	0	0	0	0	0	0
8760	Pig	Central	0	0	0	0	0	0
8761	Pig	Central	0	0	0	0	0	0
8762	Pig	Central	0	0	0	0	0	0
8763	Pig	Central	0	0	0	0	0	0
8764	Pig	Central	0	0	0	0	0	0
8765	Pig	Central	0	0	0	0	0	0
8766	Pig	Central	0	0	0	0	0	0
8767	Pig	Central	0	0	0	0	0	0
8768	Pig	Central	0	0	0	0	0	0
8769	Pig	Central	0	0	0	0	0	0
8770	Pig	Central	0	0	0	0	0	0
8771	Pig	Central	0	0	0	0	0	0
8772	Pig	Central	0	0	0	0	0	0
8773	Pig	Central	0	0	0	0	0	0
8774	Pig	Central	0	0	0	0	0	0
8775	Pig	Central	0	0	0	0	0	0
8776	Pig	Central	0	0	0	0	0	0
8777	Buffalo	Northeastern	0	0	0	0	0	0
8778	Buffalo	Northeastern	0	0	0	0	0	0
8779	Buffalo	Northeastern	0	0	0	0	0	0
8780	Buffalo	Northeastern	0	0	0	0	0	0
8781	Buffalo	Northeastern	0	0	0	0	0	0
8782	Buffalo	Northeastern	0	0	0	0	1	1
8783	Buffalo	Northeastern	0	0	0	0	1	1

8784	Buffalo	Northeastern	0	0	0	0	0	0
8785	Buffalo	Northeastern	0	0	0	0	1	1
8786	Buffalo	Northeastern	0	0	0	0	1	1
8787	Buffalo	Northeastern	0	0	0	1	0	1
8788	Buffalo	Northeastern	0	0	0	0	1	1
8789	Buffalo	Northeastern	0	0	0	0	1	1
8790	Buffalo	Northeastern	0	0	0	0	0	0
8791	Buffalo	Northeastern	0	0	0	1	1	1
8792	Buffalo	Northeastern	0	0	0	0	0	0
8793	Buffalo	Northeastern	0	0	0	0	1	1
8794	Buffalo	Northeastern	0	0	0	0	1	1
8795	Buffalo	Northeastern	0	0	0	0	1	1
8796	Buffalo	Northeastern	0	0	0	0	0	0
8797	Buffalo	Northeastern	0	0	0	0	1	1
8798	Buffalo	Northeastern	0	0	0	0	0	0
8799	Buffalo	Northeastern	0	0	0	0	0	0
8800	Buffalo	Northeastern	0	1	0	0	0	1
8801	Buffalo	Northeastern	0	0	0	0	0	0
8802	Buffalo	Northeastern	0	0	0	0	0	0
8803	Buffalo	Northeastern	0	0	0	0	0	0
8804	Buffalo	Northeastern	0	0	0	0	0	0
8805	Buffalo	Northeastern	0	0	0	0	0	0
8806	Buffalo	Northeastern	0	0	0	0	0	0
8807	Buffalo	Northeastern	0	0	0	0	0	0
8808	Buffalo	Northeastern	0	0	0	0	0	0
8809	Buffalo	Northeastern	0	0	0	0	0	0
8810	Buffalo	Northeastern	0	0	0	0	0	0
8811	Buffalo	Northeastern	0	0	0	0	0	0
8812	Buffalo	Northeastern	0	0	0	1	0	1
8813	Buffalo	Northeastern	0	0	0	1	0	1
8814	Buffalo	Northeastern	0	0	0	0	0	0
8815	Buffalo	Northeastern	0	0	0	0	1	1
8816	Buffalo	Northeastern	0	0	0	0	0	0
8817	Buffalo	Northeastern	0	0	0	0	0	0
8818	Buffalo	Northeastern	0	0	0	0	0	0
8819	Buffalo	Northeastern	0	0	0	0	0	0
8820	Buffalo	Northeastern	0	0	0	0	0	0
8821	Buffalo	Northeastern	0	0	0	1	0	1
8822	Buffalo	Northeastern	0	0	0	0	0	0
8823	Buffalo	Northeastern	0	0	0	0	0	1
8824	Buffalo	Northeastern	0	0	0	0	0	0
8825	Buffalo	Northeastern	0	0	0	1	0	1
8826	Buffalo	Northeastern	0	0	0	0	0	0
8827	Buffalo	Northeastern	0	0	0	1	0	1
8828	Buffalo	Northeastern	0	0	0	1	0	1
8829	Buffalo	Northeastern	0	0	0	0	0	0
8830	Buffalo	Northeastern	0	0	0	0	0	0
8831	Buffalo	Northeastern	0	0	0	0	0	0

8832	Buffalo	Northeastern	0	0	0	1	0	1
8833	Buffalo	Northeastern	0	0	0	0	0	0
8834	Buffalo	Northeastern	0	0	0	0	0	0
8835	Buffalo	Northeastern	0	0	0	0	0	0
8836	Buffalo	Northeastern	0	0	0	0	0	0
8837	Buffalo	Northeastern	0	0	0	0	1	1
8838	Buffalo	Northeastern	0	0	0	0	0	0
8839	Buffalo	Northeastern	0	0	0	0	0	0
8840	Buffalo	Northeastern	0	0	0	0	0	0
8841	Buffalo	Northeastern	0	0	0	0	0	0
8842	Buffalo	Northeastern	0	0	0	0	0	0
8843	Buffalo	Northeastern	0	0	0	0	0	0
8844	Buffalo	Northeastern	0	0	0	0	0	0
8845	Buffalo	Northeastern	0	1	0	0	0	1
8846	Buffalo	Northeastern	0	0	0	0	0	0
8847	Buffalo	Northeastern	0	0	0	0	0	0
8848	Buffalo	Northeastern	0	0	0	0	1	1
8849	Buffalo	Northeastern	0	0	0	0	0	0
8850	Buffalo	Northeastern	0	1	0	0	0	1
8851	Buffalo	Northeastern	0	0	0	0	0	0
8852	Buffalo	Northeastern	0	0	0	0	0	0
8853	Buffalo	Northeastern	0	0	0	0	0	0
8854	Buffalo	Northeastern	0	0	0	0	0	0
8855	Buffalo	Northeastern	0	0	0	0	0	0
8856	Buffalo	Northeastern	0	0	0	0	0	0
8857	Buffalo	Northeastern	0	0	0	0	0	0
8858	Buffalo	Northeastern	0	0	0	0	0	0
8859	Buffalo	Northeastern	0	0	0	0	0	0
8860	Buffalo	Northeastern	0	1	0	0	0	1
8861	Buffalo	Northeastern	0	0	0	0	0	0
8862	Buffalo	Northeastern	0	0	0	0	0	0
8863	Buffalo	Northeastern	0	1	0	0	0	1
8864	Buffalo	Northeastern	0	1	0	0	0	1
8865	Buffalo	Northeastern	0	0	0	0	0	0
8866	Buffalo	Northeastern	0	0	0	0	0	0
8867	Buffalo	Northeastern	0	1	0	0	0	1
8868	Buffalo	Northeastern	0	0	0	0	0	0
8869	Buffalo	Northeastern	0	0	0	0	0	0
8870	Buffalo	Northeastern	0	0	0	1	0	1
8871	Buffalo	Northeastern	0	0	0	0	0	0
8872	Buffalo	Northeastern	0	0	0	1	0	1
8873	Buffalo	Northeastern	0	0	0	0	0	0
8874	Buffalo	Northeastern	0	0	0	0	0	0
8875	Buffalo	Northeastern	0	1	0	0	0	1
8876	Buffalo	Northeastern	0	0	0	0	0	0
8877	Buffalo	Northeastern	0	1	0	0	0	1
8878	Buffalo	Northeastern	0	0	0	0	0	0
8879	Buffalo	Northeastern	0	0	0	0	0	0

8880	Buffalo	Northeastern	0	0	0	0	0	0
8881	Buffalo	Northeastern	0	0	0	0	0	0
8882	Buffalo	Northeastern	0	0	0	0	0	0
8883	Buffalo	Northeastern	0	0	0	0	0	0
8884	Buffalo	Northeastern	0	0	0	0	0	0
8885	Buffalo	Northeastern	0	0	0	0	0	0
8886	Buffalo	Northeastern	0	0	0	0	0	0
8887	Buffalo	Northeastern	0	0	0	0	0	0
8888	Buffalo	Northeastern	0	0	0	0	0	0
8889	Buffalo	Northeastern	0	0	0	0	0	0
8890	Buffalo	Northeastern	0	0	0	0	0	0
8891	Buffalo	Northeastern	0	0	0	0	0	0
8892	Buffalo	Northeastern	0	0	0	0	0	0
8893	Buffalo	Northeastern	0	0	0	0	0	0
8894	Buffalo	Northeastern	0	0	0	0	0	0
8895	Buffalo	Northeastern	0	0	0	0	0	0
8896	Buffalo	Northeastern	0	0	0	0	0	0
8897	Buffalo	Northeastern	0	0	0	0	0	0
8898	Buffalo	Northeastern	0	0	0	0	0	0
8899	Buffalo	Northeastern	0	0	0	0	0	0
8900	Buffalo	Northeastern	0	0	0	0	0	0
8901	Buffalo	Northeastern	0	0	0	0	0	0
8902	Buffalo	Northeastern	0	0	0	0	0	0
8903	Buffalo	Northeastern	0	0	0	0	0	0
8904	Buffalo	Northeastern	0	0	0	0	0	0
8905	Buffalo	Northeastern	0	0	0	0	0	0
8906	Buffalo	Northeastern	0	0	0	0	0	0
8907	Buffalo	Northeastern	0	0	0	0	0	0
8908	Buffalo	Northeastern	0	0	0	0	0	0
8909	Buffalo	Northeastern	0	0	0	0	0	0
8910	Buffalo	Northeastern	0	0	0	0	0	0
8911	Buffalo	Northeastern	0	0	0	0	0	0
8912	Buffalo	Northeastern	0	0	0	0	0	0
8913	Buffalo	Northeastern	0	0	0	0	0	0
8914	Buffalo	Northeastern	0	0	0	0	0	0
8915	Buffalo	Northeastern	0	0	0	0	0	0
8916	Buffalo	Northeastern	0	0	0	0	0	0
8917	Buffalo	Northeastern	0	0	0	0	0	0
8918	Buffalo	Northeastern	0	0	0	0	0	0
8919	Buffalo	Northeastern	0	0	0	0	0	0
8920	Buffalo	Northeastern	0	0	0	0	0	0
8921	Buffalo	Northeastern	0	0	0	0	0	0
8922	Buffalo	Northeastern	0	0	0	0	0	0
8923	Buffalo	Northeastern	0	0	0	0	0	0
8924	Buffalo	Northeastern	0	0	0	0	0	0
8925	Buffalo	Northeastern	0	0	0	0	0	0
8926	Buffalo	Northeastern	0	0	0	0	0	0
8927	Buffalo	Northeastern	0	0	0	0	0	0

8928	Buffalo	Northeastern	0	0	0	0	0	0
8929	Buffalo	Northeastern	0	0	0	0	0	0
8930	Buffalo	Northeastern	0	0	0	0	0	0
8931	Buffalo	Northeastern	0	0	0	0	0	0
8932	Buffalo	Northeastern	0	0	0	0	0	0
8933	Buffalo	Northeastern	0	0	0	0	0	0
8934	Buffalo	Northeastern	0	0	0	0	0	0
8935	Buffalo	Northeastern	0	0	0	0	0	0
8936	Buffalo	Northeastern	0	0	0	0	0	0
8937	Buffalo	Northeastern	0	0	0	0	0	0
8938	Buffalo	Northeastern	0	0	0	0	0	0
8939	Buffalo	Northeastern	0	0	0	0	0	0
8940	Buffalo	Northeastern	0	0	0	0	0	0
8941	Buffalo	Northeastern	0	0	0	0	0	0
8942	Buffalo	Northeastern	0	0	0	0	0	0
8943	Buffalo	Northeastern	0	0	0	0	0	0
8944	Buffalo	Northeastern	0	0	0	0	0	0
8945	Buffalo	Northeastern	0	0	0	0	0	0
8946	Buffalo	Northeastern	0	0	0	0	0	0
8947	Buffalo	Northeastern	0	0	0	0	0	0
8948	Buffalo	Northeastern	0	0	0	0	0	0
8949	Buffalo	Northeastern	0	0	0	0	0	0
8950	Buffalo	Northeastern	0	0	0	0	0	0
8951	Buffalo	Northeastern	0	0	0	0	0	0
8952	Buffalo	Northeastern	0	0	0	0	0	0
8953	Buffalo	Northeastern	0	0	0	0	0	0
8954	Buffalo	Northeastern	0	0	0	0	0	0
8955	Buffalo	Northeastern	0	0	0	0	0	0
8956	Buffalo	Northeastern	0	0	0	0	0	0
8957	Buffalo	Northeastern	0	0	0	0	0	0
8958	Buffalo	Northeastern	0	0	0	0	0	0
8959	Buffalo	Northeastern	0	0	0	0	0	0
8960	Buffalo	Northeastern	0	0	0	0	0	0
8961	Buffalo	Northeastern	0	0	0	0	0	0
8962	Buffalo	Northeastern	0	0	0	0	0	0
8963	Buffalo	Northeastern	0	0	0	0	0	0
8964	Buffalo	Northeastern	0	0	0	0	0	0
8965	Buffalo	Northeastern	0	0	0	0	0	0
8966	Buffalo	Northeastern	0	0	0	0	0	0
8967	Buffalo	Northeastern	0	0	0	0	0	0
8968	Buffalo	Northeastern	0	0	0	0	0	0
8969	Buffalo	Northeastern	0	0	0	0	0	0
8970	Buffalo	Northeastern	0	0	0	0	0	0
8971	Buffalo	Northeastern	0	0	0	0	0	0
8972	Buffalo	Northeastern	0	0	0	0	0	0
8973	Buffalo	Northeastern	0	0	0	0	0	0
8974	Buffalo	Northeastern	0	0	0	0	0	0
8975	Buffalo	Northeastern	0	0	0	0	0	0

8976	Buffalo	Northeastern	0	0	0	0	0	0
8977	Buffalo	Northeastern	0	0	0	0	0	0
8978	Buffalo	Northeastern	0	0	0	0	0	0
8979	Buffalo	Northeastern	0	0	0	0	0	0
8980	Buffalo	Northeastern	0	0	0	0	0	0
8981	Buffalo	Northeastern	0	0	0	0	0	0
8982	Buffalo	Northeastern	0	0	0	0	0	0
8983	Buffalo	Central	0	0	0	0	0	0
8984	Buffalo	Central	0	0	0	0	0	0
8985	Buffalo	Central	0	0	0	0	0	0
8986	Buffalo	Eastern	0	0	0	0	1	1
8987	Buffalo	Eastern	0	0	0	0	0	0
8988	Buffalo	Eastern	0	0	0	0	0	0
8989	Buffalo	Eastern	0	0	0	0	0	0
8990	Buffalo	Eastern	0	0	1	0	0	1
8991	Buffalo	Eastern	0	0	0	0	0	1
8992	Buffalo	Eastern	0	0	0	0	0	0
8993	Buffalo	Eastern	0	0	0	0	1	1
8994	Buffalo	Eastern	0	0	0	0	0	0
8995	Buffalo	Eastern	0	0	0	1	1	1
8996	Buffalo	Eastern	0	1	0	0	0	1
8997	Buffalo	Eastern	0	0	0	0	0	0
8998	Buffalo	Eastern	0	0	0	0	0	0
8999	Buffalo	Eastern	0	0	0	0	1	1
9000	Buffalo	Eastern	0	0	0	0	1	1
9001	Buffalo	Eastern	0	0	0	0	0	0
9002	Buffalo	Eastern	0	0	0	0	0	0
9003	Buffalo	Eastern	0	0	0	1	1	1
9004	Buffalo	Eastern	0	0	0	0	1	1
9005	Buffalo	Eastern	0	0	0	0	0	0
9006	Buffalo	Eastern	0	0	0	0	1	1
9007	Buffalo	Eastern	0	0	0	0	0	0
9008	Buffalo	Eastern	0	0	0	0	0	0
9009	Buffalo	Eastern	0	0	0	0	1	1
9010	Buffalo	Eastern	0	0	0	0	0	0
9011	Buffalo	Eastern	0	0	0	0	0	0
9012	Buffalo	Eastern	0	0	0	0	0	0
9013	Buffalo	Eastern	0	0	0	0	0	0
9014	Buffalo	Eastern	0	0	0	0	1	1
9015	Buffalo	Eastern	0	0	0	0	0	0
9016	Buffalo	Eastern	0	0	0	0	0	0
9017	Buffalo	Eastern	0	0	0	0	0	0
9018	Buffalo	Eastern	0	1	0	0	0	1
9019	Buffalo	Eastern	0	0	0	0	0	0
9020	Buffalo	Eastern	0	0	0	1	1	1
9021	Buffalo	Eastern	0	0	0	0	0	0
9022	Buffalo	Eastern	0	0	0	0	0	0
9023	Buffalo	Eastern	0	0	0	0	0	0

9024	Buffalo	Northeastern	0	0	0	0	0	0
9025	Buffalo	Central	0	0	0	0	0	0
9026	Buffalo	Central	0	0	0	1	0	1
9027	Buffalo	Central	0	0	0	1	0	1
9028	Buffalo	Northern	0	0	1	1	1	1
9029	Buffalo	Northern	0	0	1	1	1	1
9030	Buffalo	Northern	0	0	1	1	0	1
9031	Buffalo	Northern	0	0	0	1	0	1
9032	Buffalo	Northern	0	0	1	0	0	1
9033	Buffalo	Northern	0	0	1	1	0	1
9034	Buffalo	Northern	0	0	0	1	1	1
9035	Buffalo	Northern	0	0	1	1	0	1
9036	Buffalo	Northern	0	0	1	1	0	1
9037	Buffalo	Northern	0	0	1	1	0	1
9038	Buffalo	Northern	0	0	1	1	0	1
9039	Buffalo	Northern	0	0	1	1	0	1
9040	Buffalo	Northern	0	0	1	1	0	1
9041	Buffalo	Northern	0	0	1	1	0	1
9042	Buffalo	Northern	0	0	1	1	0	1
9043	Buffalo	Northern	0	0	1	1	0	1
9044	Buffalo	Northern	0	0	1	1	0	1
9045	Buffalo	Northern	0	0	0	0	0	0
9046	Buffalo	Northern	0	0	0	0	0	0
9047	Buffalo	Northern	0	0	0	0	0	0
9048	Buffalo	Northern	0	0	0	0	0	0
9049	Buffalo	Northern	0	0	0	0	0	0
9050	Buffalo	Northern	0	0	0	0	0	0
9051	Buffalo	Northern	0	0	0	0	0	0
9052	Buffalo	Northern	0	0	0	0	0	0
9053	Buffalo	Northern	0	0	0	0	0	0
9054	Buffalo	Northern	0	0	0	0	0	0
9055	Buffalo	Northern	0	0	0	0	0	0
9056	Buffalo	Northern	0	0	0	0	0	0
9057	Buffalo	Northern	0	0	0	0	0	0
9058	Buffalo	Northern	0	0	0	0	0	0
9059	Buffalo	Northern	0	0	0	0	0	0
9060	Buffalo	Northern	0	0	0	0	0	0
9061	Buffalo	Northern	0	0	0	0	0	0
9062	Buffalo	Northern	0	0	0	0	0	0
9063	Buffalo	Northern	0	0	0	0	0	0
9064	Buffalo	Northern	0	0	0	0	0	0
9065	Buffalo	Northern	0	0	0	0	0	0
9066	Buffalo	Northern	0	0	0	0	0	0
9067	Buffalo	Northern	0	0	0	0	0	0
9068	Buffalo	Northern	0	0	0	0	0	0
9069	Buffalo	Northern	0	0	0	0	0	0
9070	Buffalo	Northern	0	0	0	0	0	0
9071	Buffalo	Northern	0	0	0	0	0	1

9072	Buffalo	Northern	0	0	0	0	0	0
9073	Buffalo	Northern	0	0	0	0	0	0
9074	Buffalo	Northern	0	0	0	0	0	1
9075	Buffalo	Northern	0	0	0	0	0	1
9076	Buffalo	Northern	0	0	0	0	0	0
9077	Buffalo	Northern	0	0	0	0	0	0
9078	Buffalo	Northern	0	0	0	0	0	0
9079	Buffalo	Northern	0	0	0	0	0	0
9080	Buffalo	Northern	0	0	0	0	0	0
9081	Buffalo	Northern	0	0	0	0	0	0
9082	Buffalo	Northern	0	0	0	0	0	0
9083	Buffalo	Northern	0	0	0	0	0	0
9084	Buffalo	Northern	0	0	0	0	0	0
9085	Buffalo	Northern	0	0	0	0	0	0
9086	Buffalo	Northern	0	0	0	0	0	0
9087	Buffalo	Northern	0	0	0	0	0	0
9088	Buffalo	Northern	0	0	0	0	0	0
9089	Buffalo	Northern	0	0	0	0	0	0
9090	Buffalo	Northern	0	0	0	0	0	0
9091	Buffalo	Northern	0	0	0	0	0	0
9092	Buffalo	Northern	0	0	0	0	0	0
9093	Buffalo	Northern	0	0	0	0	0	0
9094	Buffalo	Central	0	0	0	1	0	1
9095	Buffalo	Central	0	0	0	0	0	0
9096	Buffalo	Central	0	0	0	0	0	0
9097	Buffalo	Central	0	0	0	0	0	0
9098	Buffalo	Central	0	0	0	0	0	0
9099	Buffalo	Central	0	0	0	0	0	0
9100	Buffalo	Central	0	0	0	0	0	0
9101	Buffalo	Central	0	0	0	0	0	0
9102	Buffalo	Central	0	0	0	0	0	1
9103	Buffalo	Central	0	0	0	0	0	0
9104	Buffalo	Central	0	0	0	0	0	0
9105	Buffalo	Central	0	0	0	0	0	0
9106	Buffalo	Central	0	0	0	0	0	0
9107	Buffalo	Central	0	0	0	0	0	0
9108	Buffalo	Southern	0	0	0	0	0	0
9109	Buffalo	Southern	0	0	0	0	0	0
9110	Buffalo	Southern	0	0	0	0	0	0
9111	Buffalo	Southern	0	0	0	0	0	0
9112	Buffalo	Southern	0	0	0	0	0	0
9113	Buffalo	Southern	0	0	0	0	0	0
9114	Buffalo	Southern	0	0	0	0	0	0
9115	Buffalo	Southern	0	0	0	0	0	0
9116	Buffalo	Southern	0	0	0	0	0	0
9117	Buffalo	Southern	0	0	0	0	0	0
9118	Buffalo	Southern	0	0	0	0	0	0
9119	Buffalo	Southern	0	0	0	0	0	0

9120	Buffalo	Southern	0	0	0	0	0	0
9121	Buffalo	Southern	0	0	0	0	0	0
9122	Buffalo	Southern	0	0	0	0	0	0
9123	Buffalo	Southern	0	0	0	0	0	0
9124	Buffalo	Southern	0	0	0	0	0	0
9125	Buffalo	Southern	0	0	0	0	0	0
9126	Buffalo	Southern	0	0	0	0	0	0
9127	Buffalo	Southern	0	0	0	0	0	0
9128	Buffalo	Southern	0	0	0	0	0	0
9129	Buffalo	Southern	0	0	0	0	0	0
9130	Buffalo	Southern	0	0	0	0	0	0
9131	Buffalo	Southern	0	0	0	0	0	0
9132	Buffalo	Southern	1	0	0	0	0	1
9133	Buffalo	Southern	0	0	0	0	0	0
9134	Buffalo	Southern	0	0	0	0	0	0
9135	Buffalo	Southern	0	0	0	0	0	0
9136	Buffalo	Southern	0	0	0	0	0	0
9137	Buffalo	Southern	0	0	0	0	0	0
9138	Buffalo	Eastern	0	0	0	0	0	0
9139	Buffalo	Eastern	0	0	0	0	0	0
9140	Buffalo	Eastern	0	0	0	0	0	0
9141	Buffalo	Eastern	0	0	0	0	0	0
9142	Buffalo	Eastern	0	0	0	0	0	0
9143	Buffalo	Eastern	0	0	0	0	0	0
9144	Buffalo	Eastern	0	0	0	0	0	0
9145	Buffalo	Eastern	0	0	0	0	0	0
9146	Buffalo	Eastern	0	0	0	0	0	0
9147	Buffalo	Northern	0	0	0	0	0	0
9148	Buffalo	Eastern	0	0	0	0	0	0
9149	Buffalo	Eastern	0	0	0	0	0	0
9150	Buffalo	Eastern	0	0	0	0	0	0
9151	Buffalo	Northern	0	0	0	0	0	0
9152	Buffalo	Central	0	0	1	1	0	1
9153	Buffalo	Central	0	0	0	0	0	0
9154	Buffalo	Central	0	0	0	1	0	1
9155	Buffalo	Central	0	0	0	1	0	1
9156	Buffalo	Eastern	0	0	1	0	0	1
9157	Buffalo	Eastern	0	0	1	1	0	1
9158	Buffalo	Eastern	0	0	0	0	0	0
9159	Buffalo	Eastern	0	0	1	1	0	1
9160	Buffalo	Eastern	0	0	0	1	0	1
9161	Buffalo	Eastern	0	0	0	0	0	0
9162	Buffalo	Eastern	0	0	0	0	0	0
9163	Buffalo	Eastern	0	0	0	0	0	0
9164	Buffalo	Eastern	0	0	1	1	0	1
9165	Buffalo	Eastern	0	0	1	1	0	1
9166	Buffalo	Central	0	0	0	0	0	0
9167	Buffalo	Central	0	0	0	0	0	0

9168	Buffalo	Central	0	0	0	0	0	0
9169	Buffalo	Central	0	0	0	0	0	0
9170	Buffalo	Central	0	0	0	0	0	0
9171	Buffalo	Central	0	0	0	0	0	0
9172	Buffalo	Northern	0	0	0	0	0	0
9173	Buffalo	Central	0	0	0	0	0	0
9174	Buffalo	Northern	0	0	0	0	0	0
9175	Buffalo	Central	0	0	0	0	0	0
9176	Buffalo	Central	0	0	0	0	0	0
9177	Buffalo	Central	0	0	1	1	0	1
9178	Buffalo	Central	0	0	0	0	0	0
9179	Buffalo	Central	0	0	0	0	0	0
9180	Buffalo	Central	0	0	0	0	0	0
9181	Buffalo	Eastern	0	0	0	0	0	0
9182	Buffalo	Eastern	0	0	0	0	0	0
9183	Buffalo	Eastern	0	0	1	1	0	1
9184	Buffalo	Eastern	0	0	1	1	0	1
9185	Buffalo	Eastern	0	0	1	1	0	1
9186	Buffalo	Eastern	0	0	0	1	0	1
9187	Buffalo	Eastern	0	0	1	0	0	1
9188	Buffalo	Eastern	0	0	0	0	0	0
9189	Buffalo	Eastern	0	0	0	0	0	0
9190	Buffalo	Eastern	0	0	1	0	0	1
9191	Buffalo	Eastern	0	0	1	0	0	1
9192	Buffalo	Eastern	0	0	1	0	0	1
9193	Buffalo	Eastern	0	0	1	0	0	1
9194	Buffalo	Central	0	0	0	0	0	0
9195	Buffalo	Central	0	0	1	1	0	1
9196	Buffalo	Central	0	0	1	0	0	1
9197	Buffalo	Central	0	1	1	1	0	1
9198	Buffalo	Central	0	1	1	1	0	1
9199	Buffalo	Central	1	0	1	1	0	1
9200	Buffalo	Central	0	0	1	1	0	1
9201	Buffalo	Central	0	0	1	1	0	1
9202	Buffalo	Central	0	1	1	1	0	1
9203	Buffalo	Central	0	0	1	1	0	1
9204	Buffalo	Central	0	0	1	0	0	1
9205	Buffalo	Central	0	0	1	0	0	1
9206	Buffalo	Central	0	0	1	0	0	1
9207	Buffalo	Central	0	1	0	0	0	1
9208	Buffalo	Northern	0	1	0	0	0	1

1 = Seropositive

0 = Seronagative
